# Supplementary material for: Multifunctional sequence-defined macromolecules for chemical data storage
Source: Nat Commun. 2018 Oct 26;9:4451. doi: 10.1038/s41467-018-06926-3 (PMC6203848; doi:10.1038/s41467-018-06926-3)
Supplement: Supplementary file 1 — Supplementary Information [file 41467_2018_6926_MOESM1_ESM.pdf]

Supplementary Information

Multifunctional sequence-defined macromolecules for chemical data storage

Martens *et al.*

Supplementary Methods

Materials

DMSO-d_6 ([2206-27-1], ≥ 99.8 %) and $CHCl_3$-*d* ([865-49-6], ≥ 99.8 %) were purchased from Euriso-top. Acryloyl chloride ([814-68-6], 96 %) was purchased from abcr GmbH. Acetonitrile ([75-05-8], HPLC grade), 1,4-Dioxane ([123-91-1], HPLC grade), and Triethylamine ([121-44-8], 99 %) were purchased from Acros Organics. DL-Homocysteinethiolactone hydrochloride ([6038-19-3], 99 %) was purchased from Haihang industry (Jinan City, China). Magnesium sulphate hydrate [22189-08-8], ≥ 99 %), Potassium carbonate ([584-08-7], ≥ 99 %) and Sodium bicarbonate ([144-55-8], ≥ 99.5 %) were purchased from Carl Roth. Trifluoroacetic acid ([76-05-1], Peptide grade) and 2-Chlorotrityl chloride resin ([42074-68-0], 100-200 mesh, 1% DVB, 1.6 mmol/g) were purchased from Iris Biotech GmbH. Acetyl chloride ([75-36-5], ≥ 99 %), Bromoacetyl bromide ([598-21-0], ≥ 98 %), Butyl acrylate ([141-32-2], ≥ 99 %), Chloroform ([865-49-6], ≥ 99.8 %), Citronellol ([106-22-9], ≥ 95 %), Dichloromethane ([75-09-2], ≥ 99.8 %), Diethylether ([60-29-7], ≥ 99.9 %), *N*,*N*-Diisopropylethylamine (DIPEA, [7087-68-5], 99 %), *N*,*N*-Dimethylformamide ([68-12-2], anhydrous, 99.8 %), Ethanolamine ([141-43-5], ≥ 99%), Ethyl acrylate ([140-88-5], 99 %), Glutaric anhydride ([108-55-4], 95 %), 1-Heptanol ([111-70-6], 98 %), 2-Hydroxyethyl acrylate ([818-61-1], 96 %), Isobornyl acrylate ([5888-33-5], technical grade), 2-Mercaptoethanol ([60-24-2], ≥ 99 %), Methanol ([67-56-1], ≥ 99.9 %), Methyl acrylate ([96-33-3], 99 %), Phenothiazine ([92-84-2], ≥ 98 %), 1-Propanol ([71-23-8], 99.7 %), Propargyl acrylate ([10477-47-1], 98 %), Pyridine ([110-86-1], ≥ 99 %), Tetrahydrofuran ([109-99-9], ≥ 99 %) were purchased from Sigma-Aldrich and used without purification, except isobornyl acrylate which was distilled. Benzyl acrylate ([2495-35-4], > 97 %), 2-Cyanoethyl Acrylate ([106-71-8], > 95 %), Cyclohexyl Acrylate ([3066-71-5], > 98 %), Dibutyltin dilaurate ([77-58-7], > 95 %), N,N-Diethylacrylamide ([2675-94-7], > 98 %), 2-(Dimethylamino)ethyl Acrylate ([2439-35-2], > 98 %), 2-Ethoxyethanol ([110-80-5], > 99 %), 2-(2-Ethoxyethoxy)ethyl Acrylate ([7328-17-8], > 98 %), 2-Ethylhexyl Acrylate ([103-11-7], > 99 %), Isoamyl Acrylate ([4245-35-6], > 98 %), 2-Methoxyethyl Acrylate ([3121-61-7], > 98 %), 1-Nonanol ([143-08-8], > 99 %) and Triphosgene ([32315-10-9], > 98 %) were purchased from TCI and used without purification. Tetrahydrofurfuryl acrylate ([2399-48-6]) was purchased from Polysciences and used without purification. Hydrochloric acid 36 % p. (HCl, [7647-01-0]) was purchased from Chem-Lab and used without purification. Solvents (CH_2Cl_2, $CHCl_3$, DIPEA and pyridine) for the chain extension of sequences, the synthesis of α-isocyanato-γ-thiolactone or the immobilization of functionalized thiolactone linkers were

distilled from CaH_2 prior to use. Silicagel (ROCC, SI 1721, 60 Å, 40 – 63 μm) was used to perform preparative column chromatography, eluting with technical solvents. The collected fractions were analyzed by thin layer chromatography (TLC-plates, Macherey-Nagel, SIL G-25 UV254). The α-isocyanato-γ-thiolactone, the acid-functionalized and hydroxyl-functionalized thiolactone linker and 3,7-dimethyloct-6-en-1-yl acrylate (citronellyl acrylate) were prepared as previously described.[1-4]

Instrumentation

^{1}H- and ^{13}C-NMR (Attached Proton Test, APT) spectra were recorded on a Bruker Avance 300 at 300 MHz and a Bruker Avance 500 at 500 MHz. Chemical shifts are presented in parts per million (δ) relative to DMSO-d_6 or $CHCl_3$-*d* (2.50 ppm or 7.27 ppm in ^{1}H- and 39.51 ppm or 77.24 ppm in ^{13}C-NMR respectively) as internal standard. All samples were analyzed with 2D-NMR techniques (COSY, HSQC and HMBC), which provided a full assignment of the structures. All measurements were performed at 25°C and ACD/NMR Processor was used for the processing of all data. All spectra including 1D ^{1}H and ^{13}C, 2D COSY, ^{1}H-^{13}C HSQC and ^{1}H-^{13}C HMBC were recorded in a standard fashion with pulse programs available in the Bruker library.

An Agilent technologies 1100 series LC/MSD system equipped with a diode array detector and single quad MS detector (VL) with an electrospray source (ESI-MS) was used for classic reversed phase LC-MS (liquid chromatography mass spectroscopy) and MS analysis. Analytic reversed phase HPLC was performed with a Phenomenex C18 (2) column (5 µ, 250 x 4.6 mm) using a solvent gradient (0 → 100% acetonitrile in H_2O in 15 min) and the eluting compounds were detected via UV-detection (λ = 214 nm). High resolution mass spectra (HRMS) were collected using an Agilent 6220 Accurate-Mass time-of-flight (TOF) equipped with a multimode ionization (MMI) source.

Infrared spectra were recorded with Attenuated Total Reflection (ATR) with a PIKE Miracle ATR unit and a Perkin Elmer FTIR SPECTRUM 1000 spectrometer. IR-software of Perkin Elmer was used for the analysis of the spectra.

Automated syntheses were performed on a 72-reactor block INTAVIS MultiPep CF Synthesizer with open 5 mL reaction columns equipped with a vortexing unit (Supplementary Figure 1). The speed of vortexing is 550 rpm.

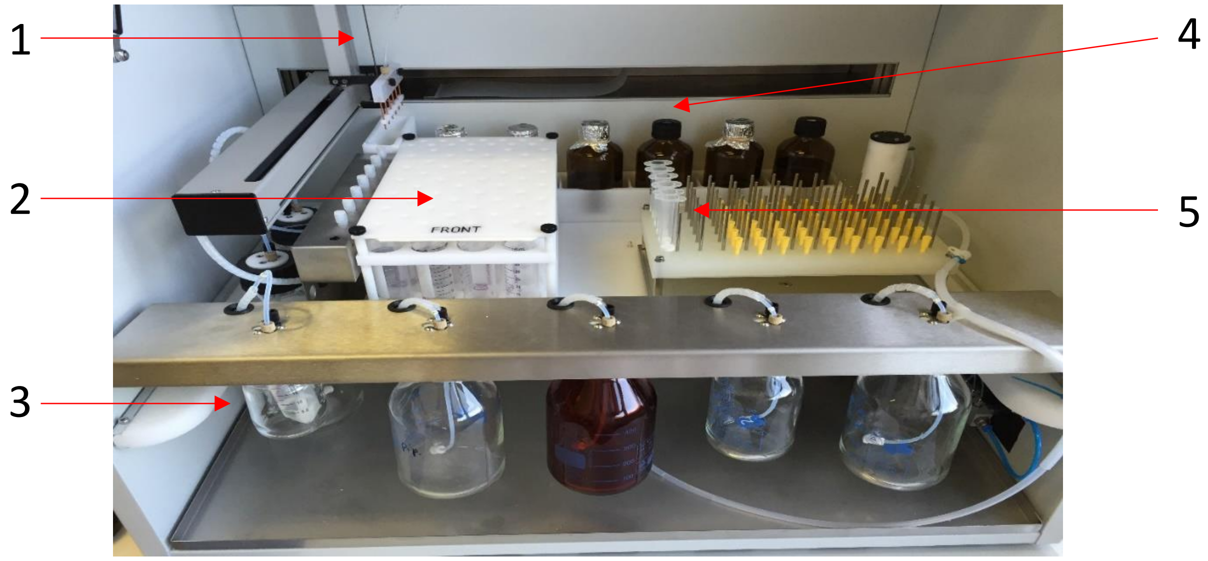

Supplementary Figure 1 | Synthesizer lay-out: needle that dispenses reagents (1), storage rack for acrylate solutions (2), reservoir for the thiolactone isocyanate protected under a nitrogen flow (3), rack with solutions, from left to right: catalyst solution, ethanolamine solution, $CHCl_3$ for washing, dry $CHCl_3$ for reaction with the thiolactone isocyanate and methanol for washing (4) and vortexing unit with reactors (5).

ESI mass spectrometry analysis was performed on a Synapt G1 HDMS mass spectrometer (Waters). Samples were diluted in 50% acetonitrile/0.1% formic acid in water and transferred into a 96-well plate. This plate was loaded into an Advion Triversa Nanomate source. From each sample 3 microliter was picked with a conductive peptide tip and moved towards the D-chip plate. Typically, 1.3 V was applied on the chip, spraying the sample in the source area of the mass spectrometer which was used in the Q-TOF mode. Tandem mass spectra were generated by collision induced dissociation using Ar as collision gas at 30eV collision energy.
For MALDI analysis, measurements were performed with trans-2-[3-(4-tert-butylphenyl)-2-methyl-2-propenylidene]malonitrile (DCTB, 30 mg/mL in dichloromethane) as a matrix, Sodium trifluoroacetate (19 mg/mL in acetone) as a cationizing agent, and oligomer samples were dissolved in THF (4 mg/mL). Oligomer solutions were prepared by mixing 10 µL of the oligomer, 1 µL of the salt, and 10 µL of the matrix solution. Subsequently, 0.5 µL of this mixture was spotted on the sample plate, and the spots were dried in air at room temperature. 0.5 microliter was spotted on a MALDI plate and loaded into the Sciex 4800 MALDI-TOF/TOF MS instrument equipped with an Nd:YAG laser (200 Hz, 355 nm) controlled by 4000 Series Explorer software version 3.5.3 (Applied Biosystems, Germany). The instrument was operated in positive ion mode with delayed extraction and an acceleration voltage of 20 kV with a grid of 15.6 kV. Fragmentation (MS/MS) was performed in positive ion mode at 1 kV using the no gas option. The 4700 Proteomics Analyser Mass Standard kit (Applied Biosystems, Germany) was prepared according to the manufacturers' recommendation and

used for external calibration before analysis (mass to charge range from 800 to 4,000 Da). MS/MS calibration was based on the precursor mass of 1,570.677 Da of Glu-fibrinopeptide B. Signals were considered as interpretable if the error in m/z was not higher than 0.02 and the signal-to-noise had to be higher than 5.

Algorithm: Chemreader

The Chemreader algorithm determines the composition of a sequence-defined polymer starting from an experimental MS/MS-spectrum of the polymer, an upper limit for the length of the polymer and a list of possible functionalities in the polymer. It contains three subscripts: a first script in which all possible theoretical fragments are generated, a second script that searches for matches between the theoretical fragments and the experimental peaks in the MS/MS spectrum and a third script in which the matched fragments are combined in order to determine the sequence. The error in m/z, as a parameter of the software, can be adjusted at will. We set the value to 0.02 for our measurements, because this is the typical error in m/z for the used MS equipment. A dimer is used as an example to give a step-by step explanation of the algorithm.

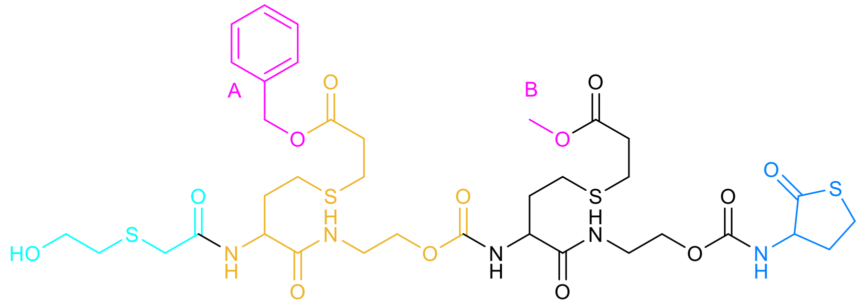

Supplementary Figure 2 | Dimer with two different functionalities. The different building blocks are indicated in different colours: start fragment (cyan); backbone (brown), stop fragment (blue) and the functionalities (magenta).

The first script generates all possible fragments starting from a SMILES representation of the primary building blocks (can be obtained via Chemdraw software for example): the backbone, the functionalities (A and B, Supplementary Figure 2), the start fragment and the stop fragment. In case of dimers with two possible functionalities A and B, the possible fragments are: A, B, {AA}, {AB}, {BB}, *A, *B, *{AB}, *{AA}, *{BB}, A*, B*, {AA}*,{AB}*, {BB}*, *{AB}*, *{AA}* and *{BB}*. The asterisk denotes the presence of a start and/or a stop fragment, while the brackets indicate that the actual order of the functionalities is unknown. The mass of all generated fragments is calculated.

The second script searches for all possible matches between the generated fragments and the masses in the MS/MS spectrum. For the given dimer, the only possible matches are: *{AB}*,

*{AB}, *A, B*, {AB}*, A, B and {AB}. In practice it is not necessarily the case that all possible fragments are observed in the measured spectrum. Furthermore, there will also be a lot of peaks in the experimental spectrum coming from fragments generated by other fragmentation patterns than the main fragmentation pattern on the urethane bond (secondary fragmentations, loss of side chain…). These phenomena might prevent an unambiguous determination of the sequence in the third part of the algorithm.

The third script reconstructs the original sequence-defined polymer from the identified fragments using Dijkstra's shortest path algorithm[5]. In order to do this, a distinction is made between fragments containing the start fragment (*{AB}*, *{AB} and *A) and fragments containing the stop fragment (*{AB}*,{AB}* and B*). Fragments without a start or a stop fragment are not used by the algorithm for now, although they could also provide useful information that is taken into account in future versions of the algorithm. With a perfect spectrum in which all fragment peaks are present, the sequence can be recovered using only the start-containing or stop-containing fragments. However, in practice, spectra are rarely perfect and there is a considerable chance that one or more peaks will be missing due to incomplete fragmentation or because a non-ionized fragment is generated. Combining the information from the start-containing fragments (reading from left to right) with the information of the stop-containing fragments (reading from right to left) increases the chance to unambiguously determine the sequence. The start fragment (alcohol or acid linker) could be easily modified in the algorithm. We provide a reference implementation of the Chemreader algorithm in Python using the RDKit module (http://www.rdkit.org). RDKit supports all necessary functions to work with molecules, e.g. calculation of the mass, bond formation and bond fragmentation. The algorithm uses a SMILES representation of molecules instead of their 2D-representation.

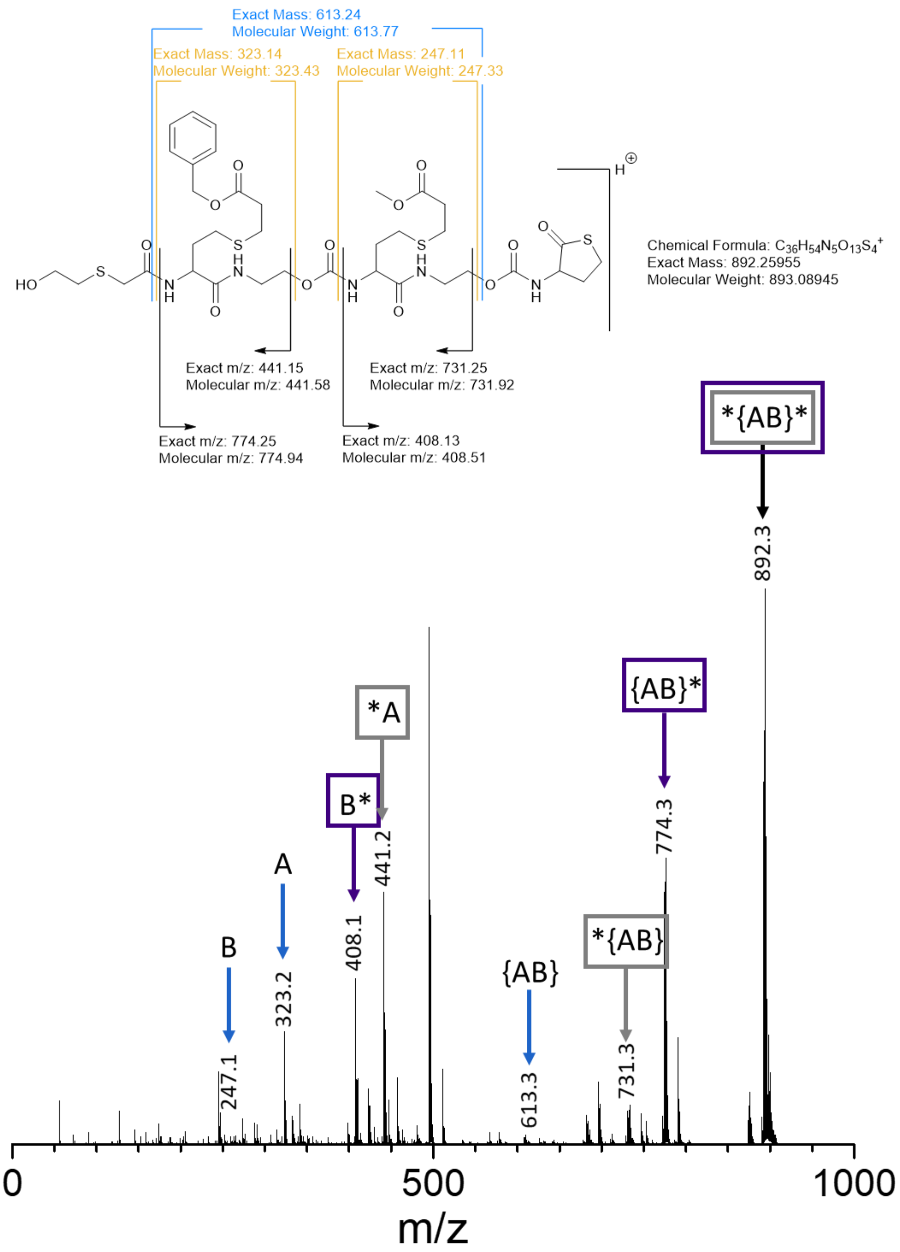

Supplementary Figure 3 | ESI-MS/MS spectrum of dimer: benzyl is A, methyl is B, the asterisk indicates the presence of a start or stop fragment and the brackets express the unknown order of functionalities in the sequence.

Synthesis of different acrylates

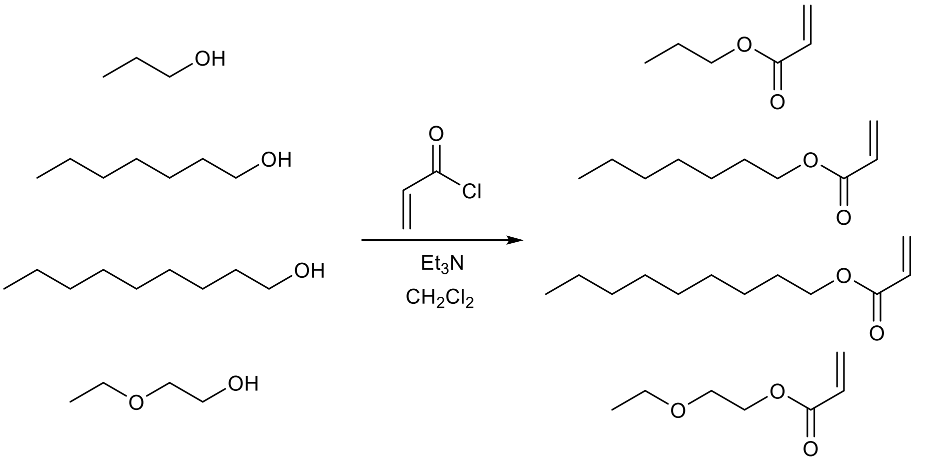

Supplementary Figure 4 | Synthesis of different acrylates starting from different alcohols.

Starting from an alcohol: The procedure was the same for every alcohol. The alcohol (100 mmol) in an ice-cooled mixture of anhydrous CH_2Cl_2 (200 ml) and anhydrous Et_3N (20.9 ml ,150 mmol) was treated with acryloyl chloride (9.8 ml, 120 mmol) by dropwise addition and stirred for 1 hour at 0°C. The reaction mixture was allowed to reach room temperature overnight. The reaction was washed with water (200 ml) and brine (200 ml). The organic fraction was dried ($MgSO_4$) and concentrated. The crude residue was purified by distillation yielding a colorless oil. Phenothiazine was added as an inhibitor and the product was stored in the fridge.

Propyl acrylate

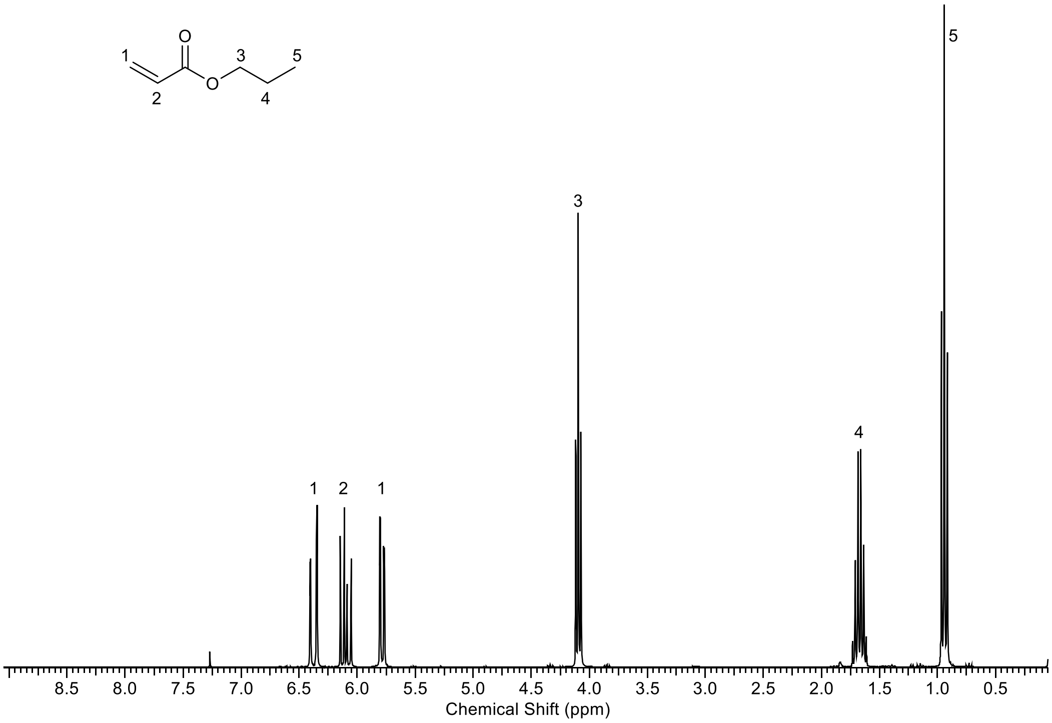

Supplementary Figure 5 | ^{1}H-NMR spectrum (300 MHz, $CHCl_3$-d) with peak assignment of purified propyl acrylate.

^{1}H-NMR (300 MHz, $CDCl_3$, ppm) δ 6.39 (1H, dd, 1), 6.12 (1H, dd, 2), 5.81 (1H, dd, 1), 4.11 (2H, t, 3), 1.69 (2H, m, 4), 0.96 (3H, t, 5)
^{13}C-NMR (75 MHz, $CDCl_3$, ppm) δ 166.3 (ester), 130.4 (1), 128.6 (2), 66.1 (3), 21.9 (4), 10.3 (5)
Boiling point: 30°C at 25 mbar

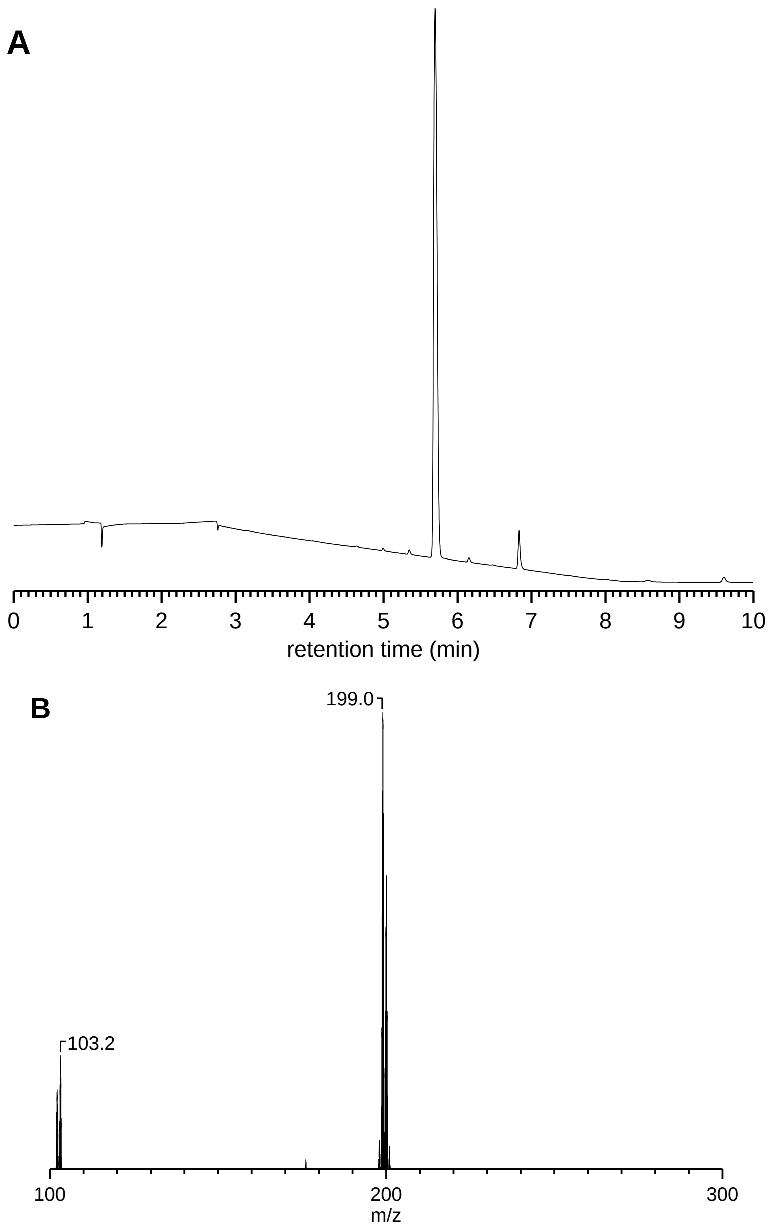

Supplementary Figure 6 | LC analysis of propyl acrylate (5.69 min) and Phenothiazine (6.83 min) (**A**). ESI-MS-spectrum of Phenothiazine (positive mode) (**B**).

Heptyl acrylate

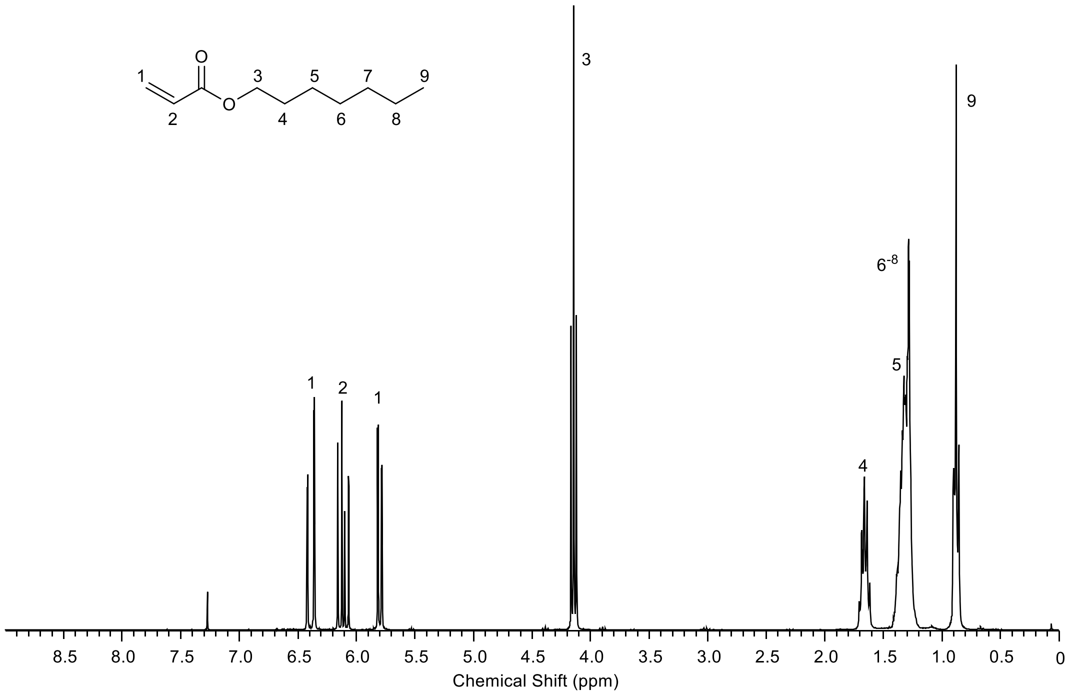

Supplementary Figure 7 | ^{1}H-NMR spectrum (300 MHz, $CHCl_3$-d) with peak assignment of purified heptyl acrylate.

^{1}H-NMR (300 MHz, $CDCl_3$, ppm) δ 6.39 (1H, dd, 1), 6.11 (1H, dd, 2), 5.80 (1H, dd, 1), 4.14 (2H, t, 3), 1.66 (2H, quint, 4), 1.33 (2H, quint, 5), 1.29 (6H, m, 6-8), 0.88 (3H, t, 9)
^{13}C-NMR (75 MHz, $CDCl_3$, ppm) δ 166.3 (ester), 130.3 (1), 128.6 (2), 64.7 (3), 31.7 (7), 28.9 (6), 28.6 (4), 25.8 (5), 22.5 (8), 14.0 (9)
Boiling point: 30°C at 1,05 mbar

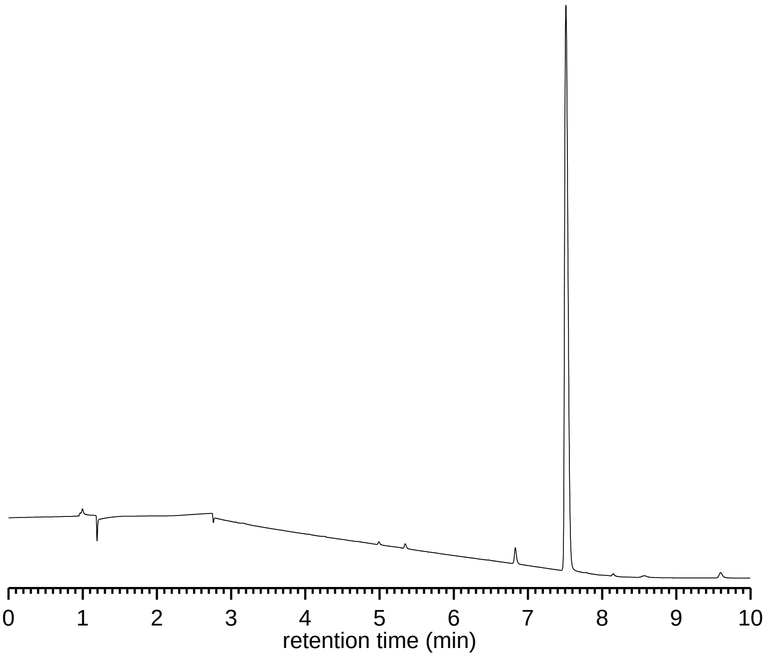

Supplementary Figure 8 | LC analysis of heptyl acrylate (7.51 min) and Phenothiazine (6.83 min).

Nonyl acrylate

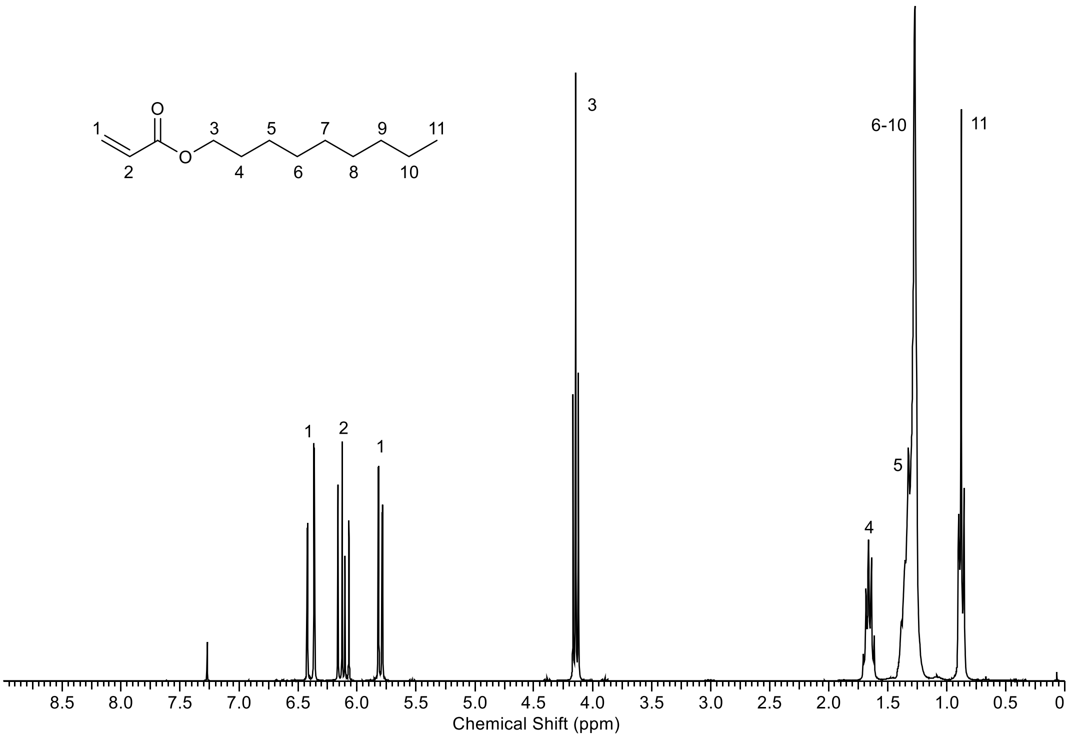

Supplementary Figure 9 | ^{1}H-NMR spectrum (300 MHz, CHCl3-d) with peak assignment of purified nonyl acrylate.

^{1}H-NMR (300 MHz, $CDCl_3$, ppm) δ 6.40 (1H, dd, 1), 6.12 (1H, dd, 2), 5.81 (1H, dd, 1), 4.15 (2H, t, 3), 1.67 (2H, quint, 4), 1.33 (2H, quint, 5), 1.28 (8H, m, 6-10), 0.88 (3H, t, 11)
^{13}C-NMR (75 MHz, $CDCl_3$, ppm) δ 166.3 (ester), 130.4 (1), 128.7 (2), 64.7 (3), 31.8 (9), 29.4, 29.3, 29.2 (6-8), 28.6 (4), 25.9 (5), 22.6 (10), 14.1 (11)
Boiling point: 45°C at 1,05 mbar

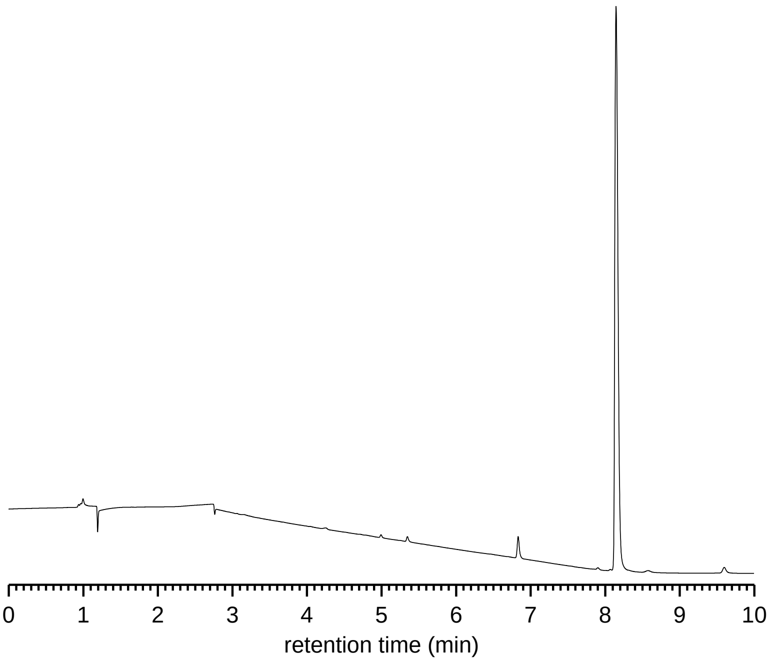

Supplementary Figure 10 | LC analysis of nonyl acrylate (8.15 min) and Phenothiazine (6.83 min).

2-ethoxyethyl acrylate

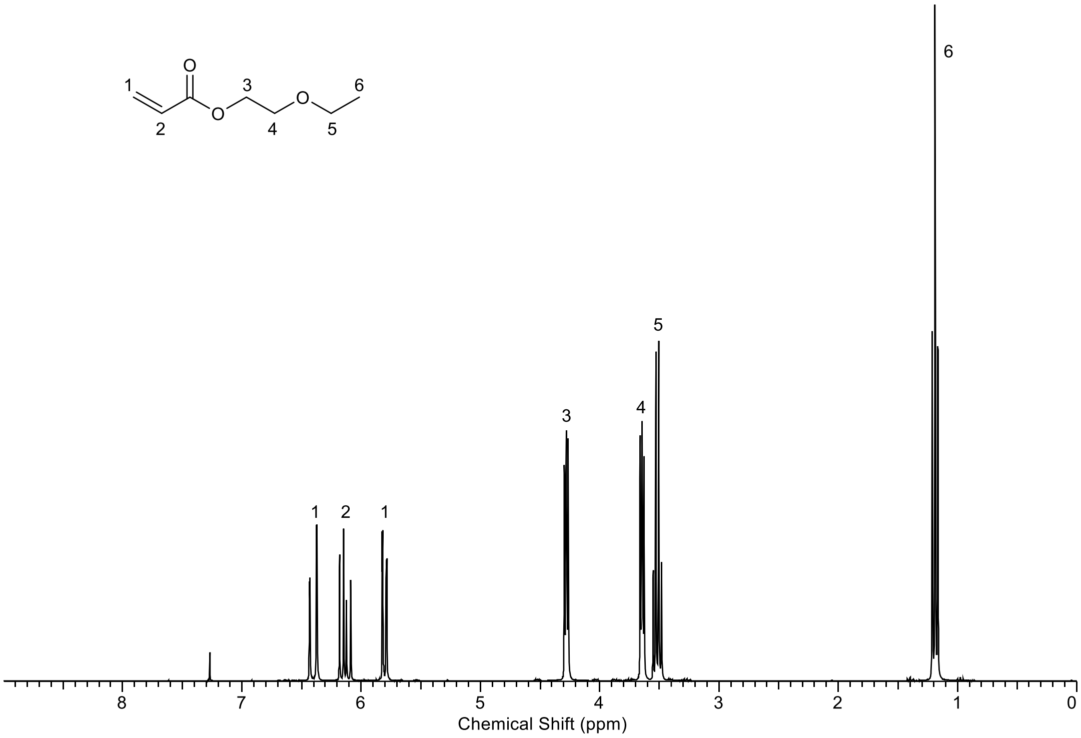

Supplementary Figure 11 | ^{1}H-NMR spectrum (300 MHz, $CHCl_3$-d) with peak assignment of purified 2-ethoxyethyl acrylate.

^{1}H-NMR (300 MHz, $CDCl_3$, ppm) δ 6.41 (1H, dd, 1), 6.14 (1H, dd, 2), 5.81 (1H, dd, 1), 4.29 (2H, t, 3), 3.65 (2H, t, 4), 3.52 (2H, q, 5), 1.20 (3H, t, 6)
^{13}C-NMR (75 MHz, $CDCl_3$, ppm) δ 166.1 (ester), 130.9 (1), 128.2 (2), 68.2 (4), 66.6 (5), 63.7 (3), 15.0 (6)
Boiling point: 25°C at 0,7 mbar
HR-MS (m/z for $[MH]^+$): *calculated m/z* $[M+H^+]$: 145.0859; *experimental m/z* $[M+H^+]$: 145.0859

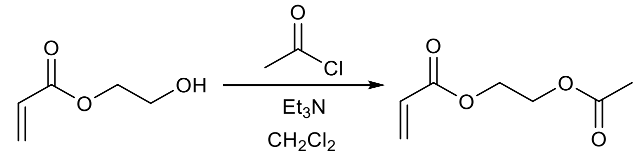

Supplementary Figure 12 | Synthesis of 2-acethoxyethyl acrylate starting from 2-hydroxyethyl acrylate.

Starting from 2-hydroxyethyl acrylate: 2-hydroxyethyl acrylate (100 mmol) in an ice-cooled mixture of anhydrous CH_2Cl_2 (200 ml) and anhydrous Et_3N (20.9 ml ,150 mmol) was treated with acetyl chloride (9.8 ml, 120 mmol) by dropwise addition and stirred for 1 hour at 0°C. The reaction mixture was allowed to reach room temperature overnight. The reaction was washed with water (200 ml) and brine (200 ml). The organic fraction was dried ($MgSO_4$) and concentrated. The crude residue was purified by distillation yielding a colorless oil. Phenothiazine was added as an inhibitor and the product was stored in the fridge.

2-acethoxyethyl acrylate

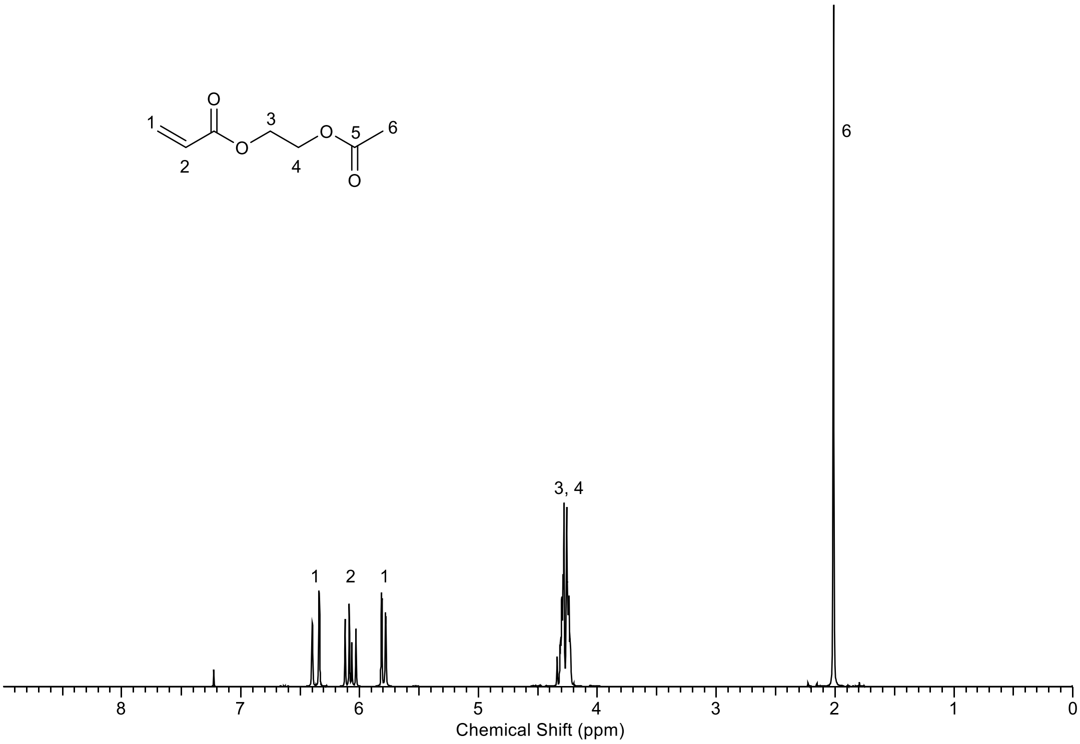

Supplementary Figure 13 | ^{1}H-NMR spectrum (300 MHz, $CHCl_3$-d) with peak assignment of purified 2-acethoxyethyl acrylate.

^{1}H-NMR (300 MHz, $CDCl_3$, ppm) δ 6.41 (1H, dd, 1), 6.12 (1H, dd, 2), 5.84 (1H, dd, 1), 4.31 (4H, m, 3 and 4), 2.05 (3H, s, 6)
^{13}C-NMR (75 MHz, $CDCl_3$, ppm) δ 170.7 (5), 165.8 (ester), 131.3 (1), 127.9 (2), 62.2 (4), 62.1 (3), 20.8 (6)
Boiling point: 42°C at 0,7 mbar
HR-MS (m/z for $[MH]^+$): *calculated m/z* $[M+NH_4^+]$: 176.0917; *experimental m/z* $[M+NH_4^+]$: 176.0922

Manipulation of the solid support

Swelling

The solid phase was swollen in the reaction solvent for at least 10 minutes.

Washing

The solid phase was washed with DMF (4x), methanol (4x), $CHCl_3$ (4x) and diethyl ether (4x) and dried after each reaction.

Loading

The loading and calibration curve of the thiolactone linkers were according to literature procedures.[1, 3]

Storing of the samples

Samples, oligomers on the solid support, are stored in the fridge if not needed for reactions or cleavage.

Cleavage

For cleavage, 2 mg of solid support was treated with 0.5 mL of 1% trifluoroacetic acid (TFA) in CH_2Cl_2 for 5 minutes. During cleavage, the color of the beads turns from yellow to red because of the formation of a trityl cation. The solid support was then filtered, the sample concentrated by evaporation and dissolved in MeCN for LC-ESI-MS analysis. For the tandem MS analysis, samples were dissolved in THF.

Aminolysis and incorporation of functionalities

$CHCl_3$ is added to the solid support (1 mL reaction mixture per 100 mg resin). Next, the acrylic (30 equivalents relative to the thiolactone unit) and ethanolamine (15 equivalents) were added. This mixture is shaken for 15 minutes at room temperature. This procedure is repeated once more to be sure of the full conversion of the thiolactone. After this second procedure, the resin is washed with DMF (4x), methanol (4x), $CHCl_3$ (4x) and diethyl ether (4x) and dried.

Exceptions: propargyl acrylate and 2-cyanoethyl acrylate required longer reaction times to fully open the thiolactone ring. This mixture is shaken for 15 minutes at room temperature. This procedure is repeated three times: once for 15 minutes and two times overnight.

Chain extension

Dried $CHCl_3$ is added to the solid support (1 mL reaction mixture per 100 mg resin), then the α-isocyanato-γ-thiolactone (10 eq. relative to the resin-bound alcohol-units) and dibutyltin dilaurate (0,025 eq. relative to the resin-bound alcohol-units) are added. This mixture is shaken for 1 hour. Afterwards, the resin is washed with DMF (4x), MeOH (4x), $CHCl_3$ (4x) and diethyl ether (4x) and dried.

Automated protocol

The protocol was adapted from the conventional approach with some slight modifications.[3] Stable stock solutions were prepared in $CHCl_3$ with either (i) the ethanolamine (7M), (ii) an acrylate (4M), (iii) the α-isocyanato-γ-thiolactone (2M) or (iv) the dibutyltin dilaurate (0.15M). 50 mg of resin was weighed in each reactor.

The resin was swollen for 5 minutes in $CHCl_3$ and afterwards the solvent was removed. 480 µL of the acrylate/$CHCl_3$ solution and 180 µL of the ethanolamine/$CHCl_3$ solution were added in this order and shaken for 15 minutes. This step was repeated for a second time like in the manual approach. Then the resin was washed with DMF (2x), MeOH (2x) and $CHCl_3$ (2x).

Subsequently, the solid support is suspended for 5 minutes in $CHCl_3$ and afterwards the solvent removed. 480 µL of the α-isocyanato-γ-thiolactone/$CHCl_3$ solution and 120 µL of the dibutyltin dilaurate/$CHCl_3$ solution were added in this order and shaken for 2 hours. During this reaction a white precipitate is formed[3]. Next, 1 mL of DMF is added and shaken for 1 minute. This allows for the white precipitate that is formed, to dissolve and to prevent the tubings of the robot from clogging. The resin was then washed with DMF (2x), MeOH (2x) and $CHCl_3$ (2x). After completion of the automated procedure, the resin was washed manually with DMF (4x), MeOH (4x), $CHCl_3$ (4x) and diethyl ether (4x), and dried.

Supplementary Tables and Figures

Supplementary Table 1 | HRMS data of the different sequences made with different functionalities: benzyl (ben), methyl (met), tetrahydrofurfuryl (thf), butyl (but), isobornyl (isob), nonyl (non), propargyl (pgyl), cyclohexyl (che), ethyl (eth), methoxyethyl (moe), isoamyl (iam), ethoxyethyl (eoe), 2-acetoxyethyl (ace), heptyl (hep), propyl (pro), citronellyl (cit), 2-cyanoethyl (cyn), 2-ethylhexyl (ehe), dimethylaminoethyl (dmae), diethylacrylamide (deaa) and 2-(2-ethoxyethoxy)ethyl (eee). The letters indicate the different oligomers: Z = started with the acid linker, H = hexamer made with the robot and the alcohol linker, A = started with the alcohol linker and QR = part of the QR code and started with the acid linker.

	Sequence	m/z_{theo}	m/z_{exp}	Estimation of purity (%)*
**Z5**	ben met thf but isob	1992.7617	1992.7591	97
**H1**	ben thf ben ben thf ben	1211.4112	1211.4083	88
**H2**	ben thf but ben but ben	1180.4216	1180.4199	90
**H3**	ben thf thf ben thf thf	1205.4218	1205.4195	89
**H4**	ben but ben thf thf ben	1194.4190	1194.4154	89
**H5**	ben but ben thf ben but	1180.4216	1180.4184	85
**H6**	ben ben but ben ben thf	1197.4137	1197.4099	86
**A1TO**	cyn ben met	1221.3641	1221.3616	85
**A2WRITE**	eee but thf eth ben pro	2308.8380	2308.8348	89
**A3OR**	ehe met thf	1274.4733	1274.4737	98
**A4NOT**	non isob met ben	1706.6816	1706.6842	89
**A5TO**	cit ben met	1306.4784	1306.4769	95
**A6WRITE**	hep but thf eth ben pro	2290.8638	2290.8690	89
**A7ON**	ace met isob	1300.4526	1300.4515	82
**A8OLIGOS?**	pgyl met che eth moe met iam eoe	2820.9990	2820.0042	75
**Z1TO**	cyn ben met	1217.3869	1217.3810	84
**Z2WRITE**	eee but thf eth ben pro	2304.8608	2304.8570	97
**Z3OR**	ehe met thf	1270.4962	1270.4947	97
**Z4NOT**	non isob met ben	1702.7044	1702.7096	94

**Z5TO**	cit ben met	1302.5012	1302.5003	91
**Z6WRITE**	hep but thf eth ben pro	2286.8866	2286.8906	92
**Z7ON**	ace met isob	1296.4754	1296.4751	86
**Z8OLIGOS?**	pgyl met che eth moe met iam eoe	2817.0219	2817.0180	75
**QR1**	che	590.2201	590.2196	97
**QR2**	ben met pro ben eee	1964.6940	1964.6981	94
**QR3**	but ehe ben dmae ben	2031.8090	2031.8152	94
**QR4**	but ehe eth but deaa	1919.8141	1919.8106	93
**QR5**	iam thf eth thf ehe	1990.8036	1990.8007	93
**QR6**	iam deaa dmae dmae ben	1969.8046	1969.8022	92
**QR7**	dmae met ehe iam pro	1921.7933	1921.8019	90
**QR8**	eee che isob eth ehe	2086.8975	2086.9046	92
**QR9**	pro met but pro ehe	1878.7511	1878.7495	95
**QR10**	hep thf ben met moe	1956.7253	1956.7251	97
**QR11**	moe che hep isob iam	2056.8869	2056.8865	93
**QR12**	moe ehe iam moe ben	2000.7879	2000.7878	91
**QR13**	ben ben eee ehe ehe ben	2499.0067	2499.0062	89
**QR14**	ben ben hep deaa eee che	2419.9758	2419.9843	87
**QR15**	ben but thf iam ehe ben	2390.9492	2390.9464	90
**QR16**	ben eth met but but eee	2248.8346	2248.8416	88
**QR17**	ben iam moe moe eth che	2274.8502	2274.8401	86
**QR18**	ben deaa che ben isob thf	2425.9652	2425.9539	86
**QR19**	ben dmae met dmae moe moe	2250.8251	2250.8350	96
**QR20**	ben eee dmae deaa dmae hep	2389.9976	2390.0029	91
**QR21**	ben pro met hep met eee	2262.8502	2262.8509	90
**QR22**	ben hep iam iam met pro	2272.9074	2272.9142	91
**QR23**	ben ehe deaa met moe eth	2245.8713	2245.8801	90
**QR24**	thf che deaa isob thf thf	2413.9863	2413.9872	88
**QR25**	thf ben pro isob che but	2378.9420	2378.9443	88
**QR26**	thf thf iam thf isob but	2402.9704	2402.9750	89
**QR27**	thf met che iam iam met	2222.8553	2222.8560	92
**QR28**	thf eth hep iam eee moe	2342.9340	2342.9398	91
**QR29**	thf deaa che che moe pro	2291.9132	2291.9159	87
**QR30**	thf deaa ehe met hep che	2333.9965	2333.9919	90
**QR31**	thf isob eth dmae but deaa	2318.9605	2318.9507	88
**QR32**	thf eee thf ehe isob iam	2491.0592	2491.0697	89
**QR33**	thf pro deaa hep dmae moe	2296.9397	2296.9483	92
**QR34**	thf ehe che hep met ehe	2391.0431	2391.0468	93
**QR35**	thf moe che hep iam thf	2364.9547	2364.9560	87
**QR36**	but che pro dmae thf dmae	2294.9241	2294.9137	92
**QR37**	but ben isob iam met thf	2338.9179	2338.9255	93
**QR38**	but but but eee che isob	2391.0067	2391.0082	82
**QR39**	but met but ben ben met	2208.7822	2208.7721	83
**QR40**	but iam isob moe dmae dmae	2350.9867	2350.9978	93
**QR41**	but iam pro hep thf isob	2375.0118	2375.0224	94
**QR42**	but dmae iam isob ben pro	2353.9652	2353.9723	92
**QR43**	but isob eth eee met isob	2374.9754	2374.9671	91
**QR44**	but eee moe hep eee thf	2416.9708	2416.9639	91

**QR45**	but hep isob but met iam	2318.9856	2318.9828	92
**QR46**	but moe che but eee moe	2314.9027	2314.8942	89
**QR47**	met che ben moe moe isob	2326.8815	2326.8758	84
**QR48**	met ben ben thf ben hep	2354.8553	2354.8499	86
**QR49**	met thf hep but deaa pro	2237.9026	2237.9063	87
**QR50**	met met che eth dmae but	2153.8087	2153.8031	89
**QR51**	met eth che pro iam eth	2152.8135	2152.8103	90
**QR52**	met iam thf deaa but moe	2225.8662	2225.8567	85
**QR53**	met deaa deaa thf deaa ben	2241.8876	2241.8805	84
**QR54**	met isob ehe che ben isob	2459.0482	2459.0371	86
**QR55**	met eee but iam iam ehe	2326.9754	2326.9646	84
**QR56**	met pro dmae iam but ehe	2253.9339	2253.9401	95
**QR57**	met ehe eth pro dmae eee	2271.9081	2271.9082	94
**QR58**	met moe met moe dmae met	2117.7359	2117.7336	95
**QR59**	eth che iam but ehe eth	2264.9387	2264.9363	88
**QR60**	eth ben pro ehe ben moe	2308.8710	2308.8759	93
**QR61**	eth but deaa moe thf ehe	2281.9288	2281.9260	90
**QR62**	eth eth thf eee ben moe	2292.8244	2292.8222	92
**QR63**	eth iam eth che but eee	2268.8972	2268.8946	90
**QR64**	eth deaa dmae eee moe met	2230.8564	2230.8531	95
**QR65**	eth isob che deaa ehe che	2384.0486	2384.0449	96
**QR66**	eth isob moe hep iam eth	2306.9492	2306.9517	93
**QR67**	eth eee deaa che eth but	2253.8975	2253.8951	91
**QR68**	eth hep pro moe but eth	2198.8553	2198.8655	89
**QR69**	eth moe met pro che che	2194.8240	2194.8321	90
**QR70**	eth moe isob pro met thf	2250.8502	2250.8590	91
**QR71**	iam che isob but moe eee	2407.0017	2407.0087	86

* Estimation of purity determined with LC analysis.

*Characterization of **Z5** using mass spectrometry (Supplementary Figure 14), NMR spectroscopy (Supplementary Figure 15) and tandem mass analysis (Supplementary Figures 16-17).*

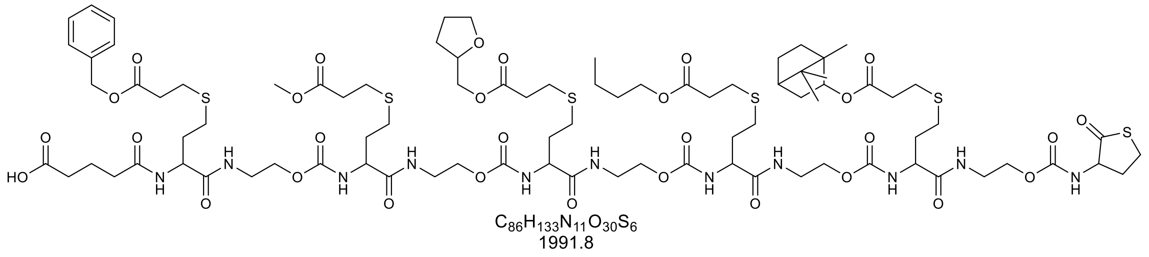

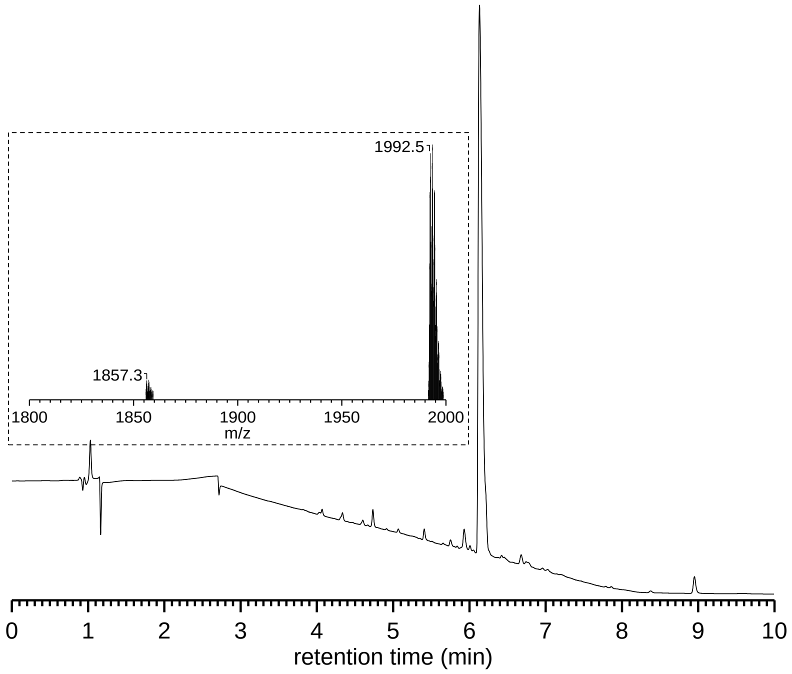

Supplementary Figure 14 | LC-ESI-MS analysis of **Z5**. Insert: ESI-MS-spectrum of dominant species (positive mode).

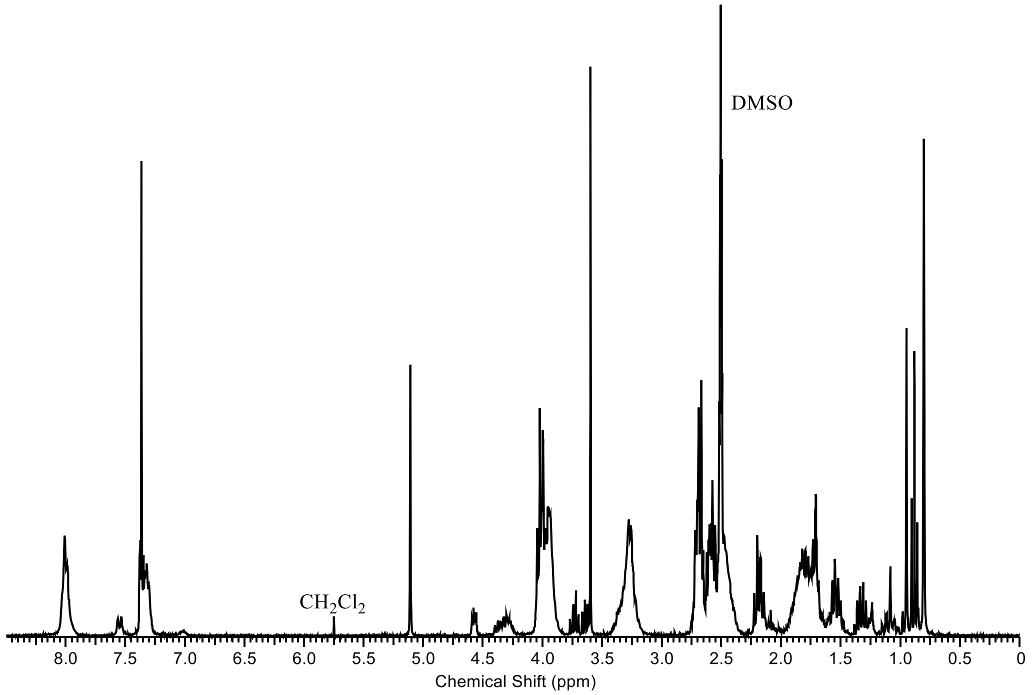

Supplementary Figure 15 | ^{1}H-NMR spectrum (500 MHz, DMSO-d$_6$) with peak assignment of **Z5**.

Supplementary Table 2 | determination of the [1]H- and ^{13}C-chemical shift values of **Z5**

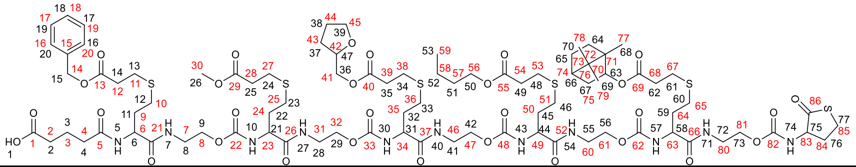

δ (ppm)	H	δ (ppm)	C
13.25	1	174.24	1
8.01	5, 7, 27, 40, 54 and 71	171.69	5, 21, 26, 37, 52 and 66
7.55	82	155.88	74
7.36	16-20	127.9, 127.99 and 128.41	16-20
7.33	10, 30, 43 and 57	155.88	22, 33, 48 and 62
5.11	15	65.59	14
4.58	63	80.3	70
4.35	75	59.88	83
4.31	6	51.81	6
4.02	50	63.72	56
4.01 and 4	36 and 47	66 and 75.75	41 and 42
3.97	73, 56, 42, 29, 9 and 21, 31, 44, 58	62.50 and 53.90	81, 61, 47, 32, 8 and 23, 34, 49, 63
3.73/3.63	39	67.42	45
3.6	26	51.41	30

δ (ppm)	H	δ (ppm)	C
3.3	77	26.36	85
3.27	8, 28, 41, 55 and 72	38.15 /33.86	7, 31, 46, 60 and 80
2.72	13	26.11	11
2.69	24, 34, 48 and 61	26.01/26.20	27, 38, 53 and 67
2.64	14, 35, 49 and 62	34.26/34.30/ 34.60	12, 39, 54 and 68
2.6	25	34.11	28
2.46	12, 23, 33, 46 and 60	27.36/ 27.49	10, 25, 36, 51 and 65
2.43	76	29.8	84
2.18	2 and 4	33.03/ 34.25	2 and 4
2.09	76	29.8	84
1.81	11, 22, 32, 45 and 59; 37and 38	32.05; 27.46 and 25.17	9, 24, 35, 50 and 64; 43 and 44
1.71	67	38.32	75

δ (ppm)	H	δ (ppm)	C
1.7	3 and 66	20.61/44.37	3 and 74
1.63	64	26.6	72
1.55	37 and 51	27.46 and 30.16	43 and 57
1.5	65	33.24	73
1.33	52	18.59	58
1.09	65	33.24	73
1.05	64	26.6	72
0.95	68	19.73	77
0.88	53	13.53	59
0.8	69 and 70	11.39 and 19.93	78 and 79
**δ (ppm)**	**C**	**δ (ppm)**	**C**
171.34	13	171.49	55
136.06	15	170.73	69
205.55	86	48.21	76
171.93	29	46.53	71
171.4	40		

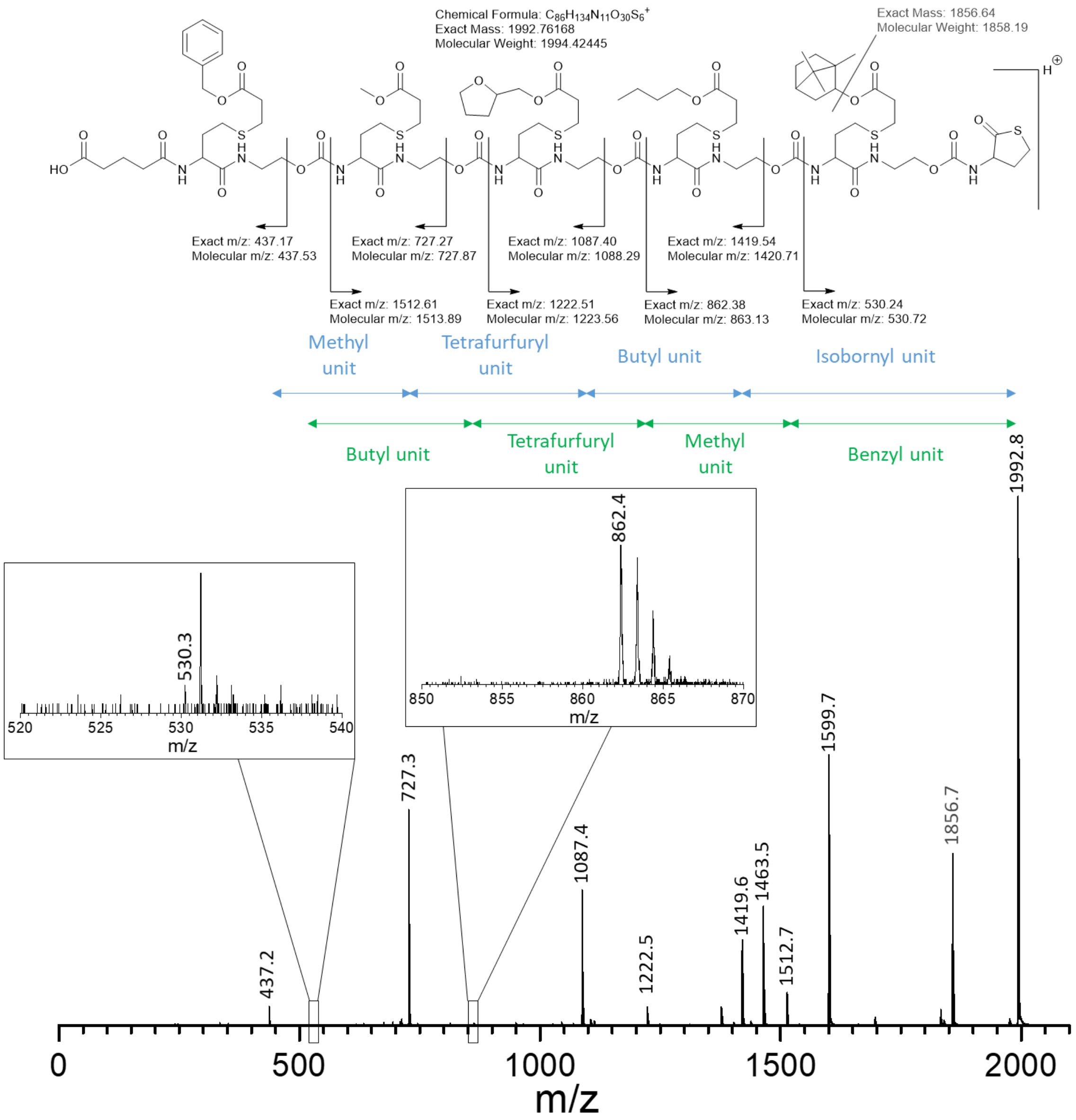

Supplementary Figure 16 | ESI-MS/MS spectrum with peak assignment of **Z5**.

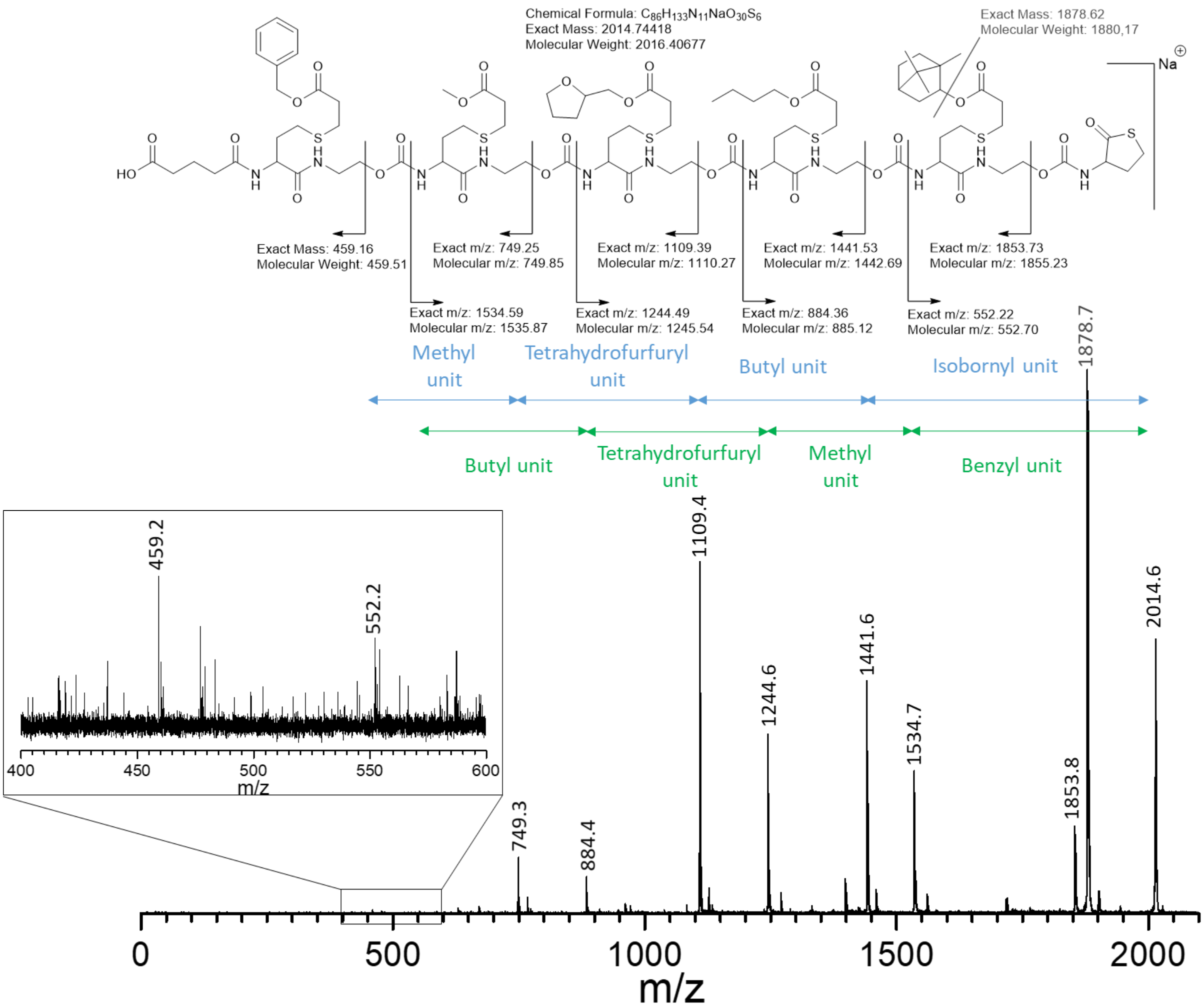

Supplementary Figure 17 | MALDI-MS/MS spectrum with peak assignment of **Z5**.

*Characterization of **H1** using mass spectrometry (Supplementary Figure 18), NMR spectroscopy (Supplementary Figure 19) and MALDI-MS/MS analysis (Supplementary Figure 20).*

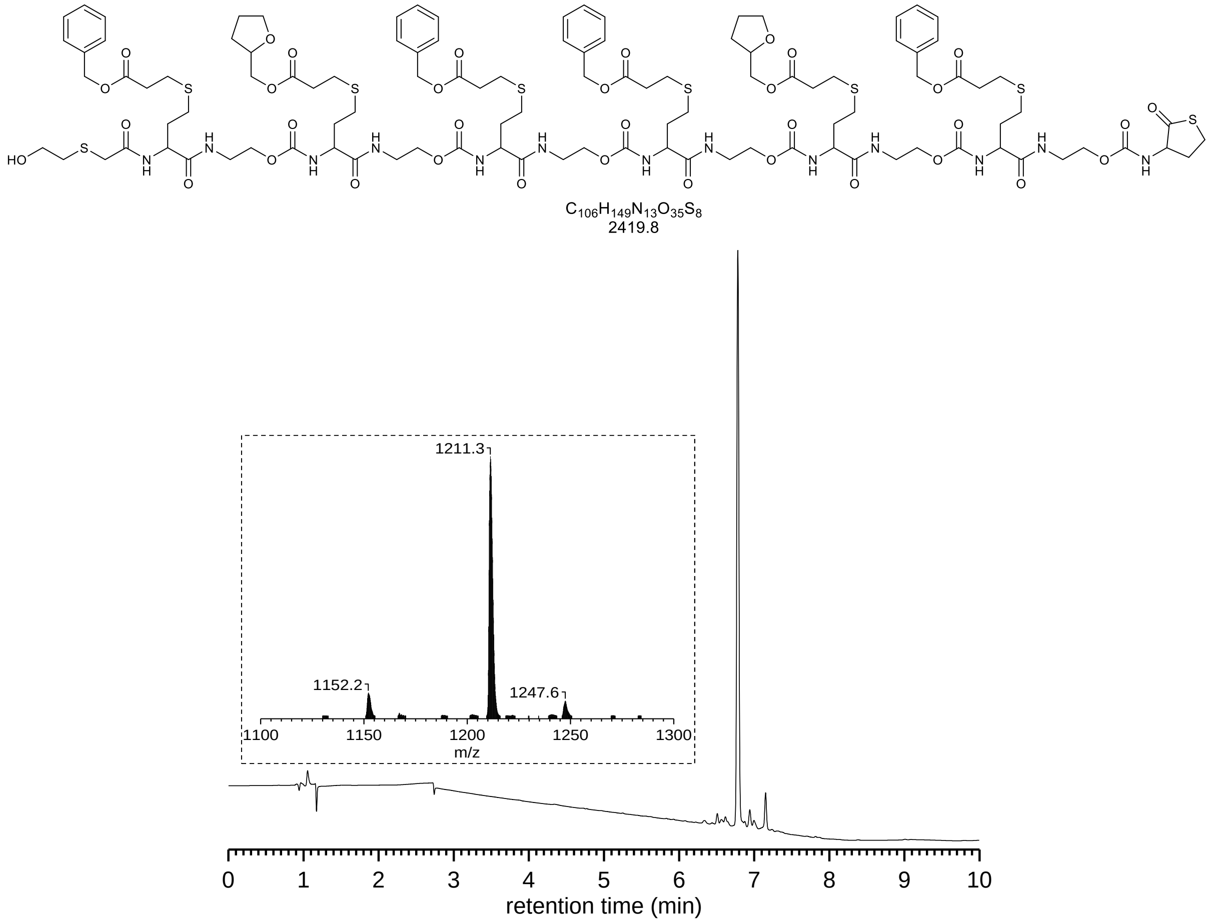

Supplementary Figure 18 | LC-ESI-MS analysis of **H1**. Insert: ESI-MS-spectrum of dominant species (positive mode).

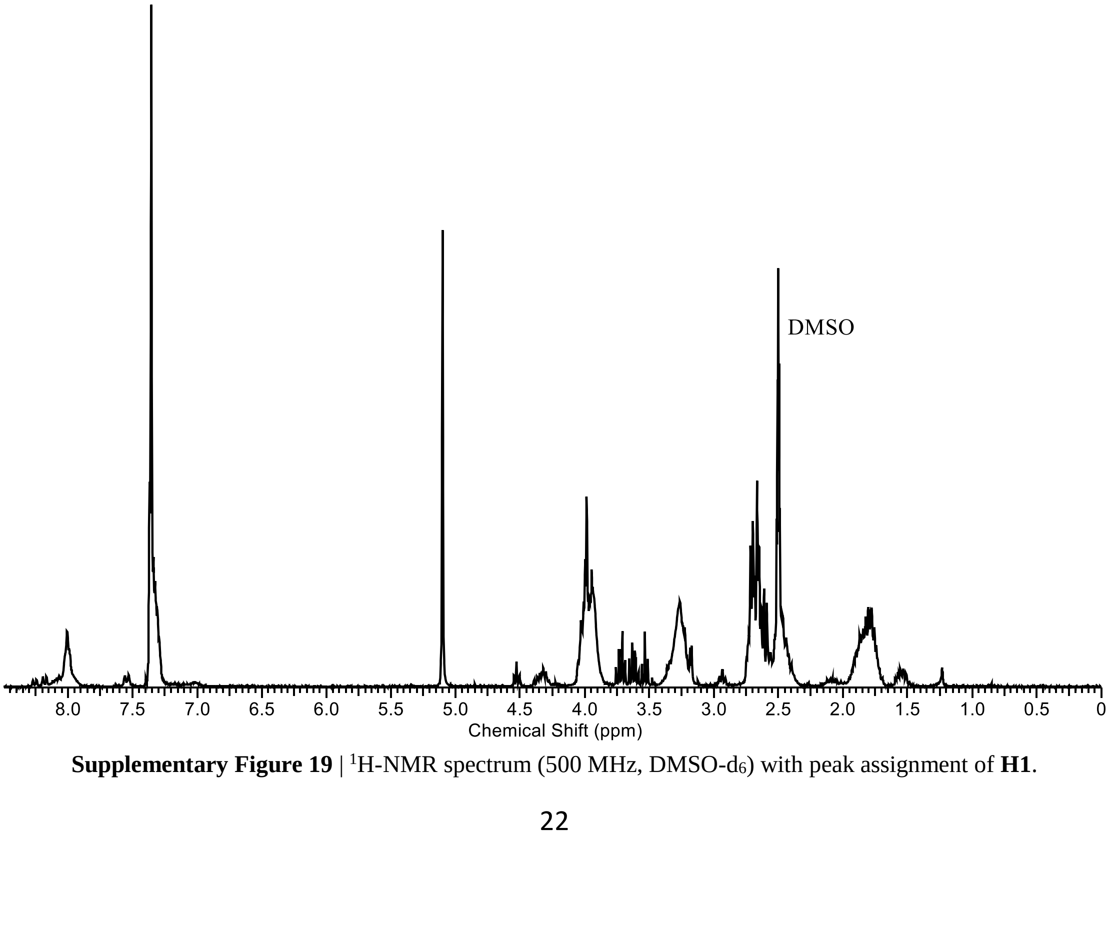

Supplementary Figure 19 | ^{1}H-NMR spectrum (500 MHz, DMSO-d$_6$) with peak assignment of **H1**.

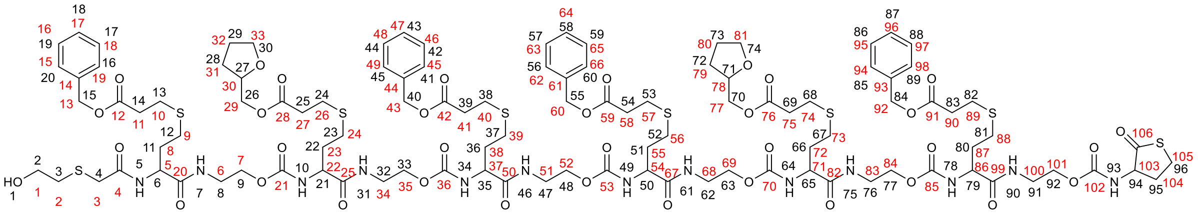

Supplementary Table 3 | determination of the ^{1}H- and ^{13}C-chemical shift values of **H1**

δ (ppm)	H	δ (ppm)	C
8.18	5	169.37	4
8.07	7	171.21	20
8.01	31, 46, 61, 75 and 90	171.69	25, 50, 67, 82 and 99
7.55	93	155.88	102
7.35	16-20, 41-45, 56-60 and 85-89	127.93, 128.01 and 128.43	15-19, 45-49, 62-66 and 94-98
7.32	10, 34, 49, 64 and 78	155.88	21, 36, 53, 70 and 85
5.09	15, 40, 55 and 84	65.59	13, 43, 60 and 92
4.53	1	/	/
4.35	94	59.88	103
4.31	6	51.99	5
4.01	26 and 70	66	29 and 77
4	27 and 71	75.75	30 and 78
3.97	92, 77, 63, 48, 33, 9 and 21, 35, 50, 65, 79	62.50 and 53.90	101, 84, 69, 52, 35, 7 and 22, 37, 54, 71, 86
3.73	30 and 74	67.42	33 and 81
3.63	30 and 74	67.42	33 and 81
3.53	2	60.45	1
3.3	96	26.4	105
3.27	8, 32, 47, 62, 76 and 91	38.15	6, 34, 51, 68, 83 and 100
3.17	4	34.5	3
2.69	13, 24, 38, 53, 68 and 82	26.04	10, 26, 40, 57, 74 and 89
2.64	3	34.54	2
2.6	14, 25, 39, 54, 69 and 83	34.27 and 34.30	11, 27, 41, 58, 75 and 90
2.46	12, 23, 37, 52, 67 and 81	27.49	9, 24, 39, 56, 73 and 88
2.43	95	29.84	104
2.09	95	29.84	104
1.89	28 and 72	27.49	31 and 79
1,81	11, 22, 36, 51, 66 and 80; 29 and 73	32.11 and 25.20	8, 23, 38, 55, 72 and 87; 32 and 80
1.55	28 and 72	27.49	31 and 79
		205.55	106
		171.4	28 and 76
		171.34	12, 42, 59 and 91
		136.06	14, 44, 61 and 93

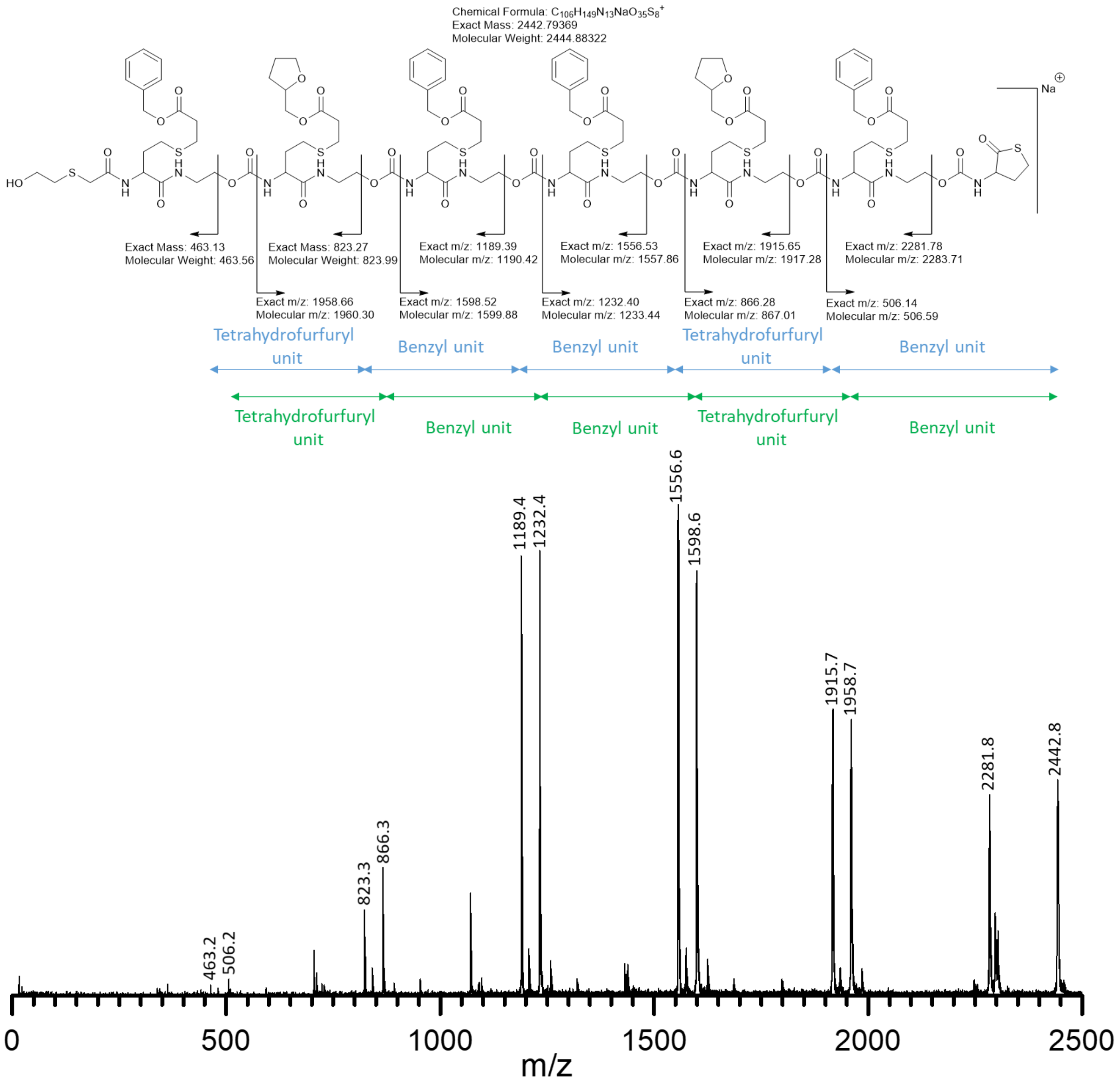

Supplementary Figure 20 | MALDI-MS/MS spectrum with peak assignment of **H1**.

*Characterization of **H2** using mass spectrometry (Supplementary Figure 21), NMR spectroscopy (Supplementary Figure 22) and MALDI-MS/MS analysis (Supplementary Figure 23).*

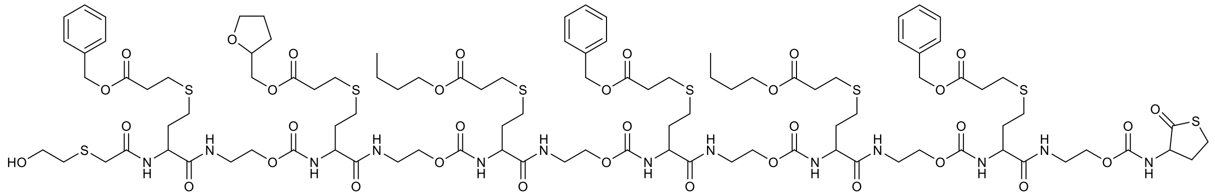

$C_{102}H_{151}N_{13}O_{34}S_8$
2357.8

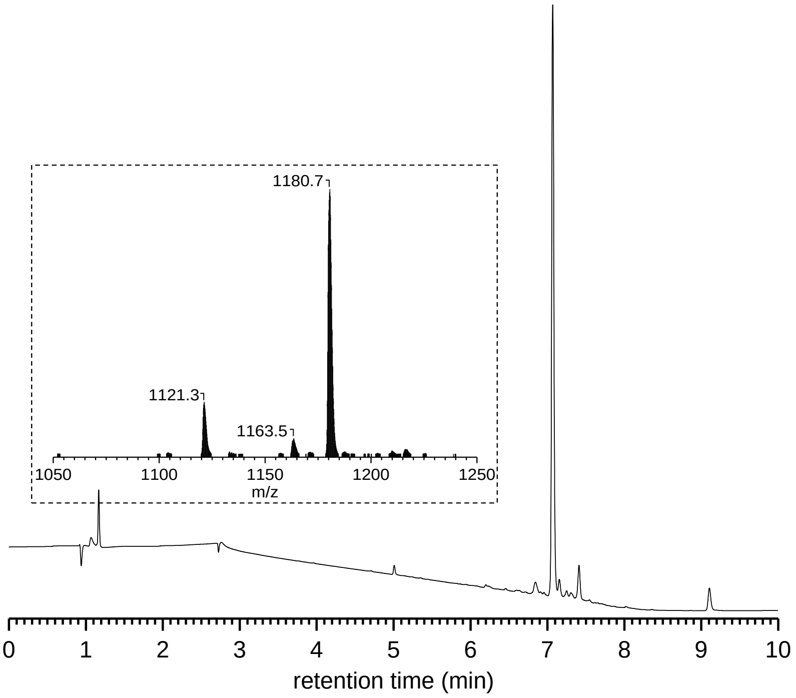

Supplementary Figure 21 | LC-ESI-MS analysis of **H2**. Insert: ESI-MS-spectrum of dominant species (positive mode).

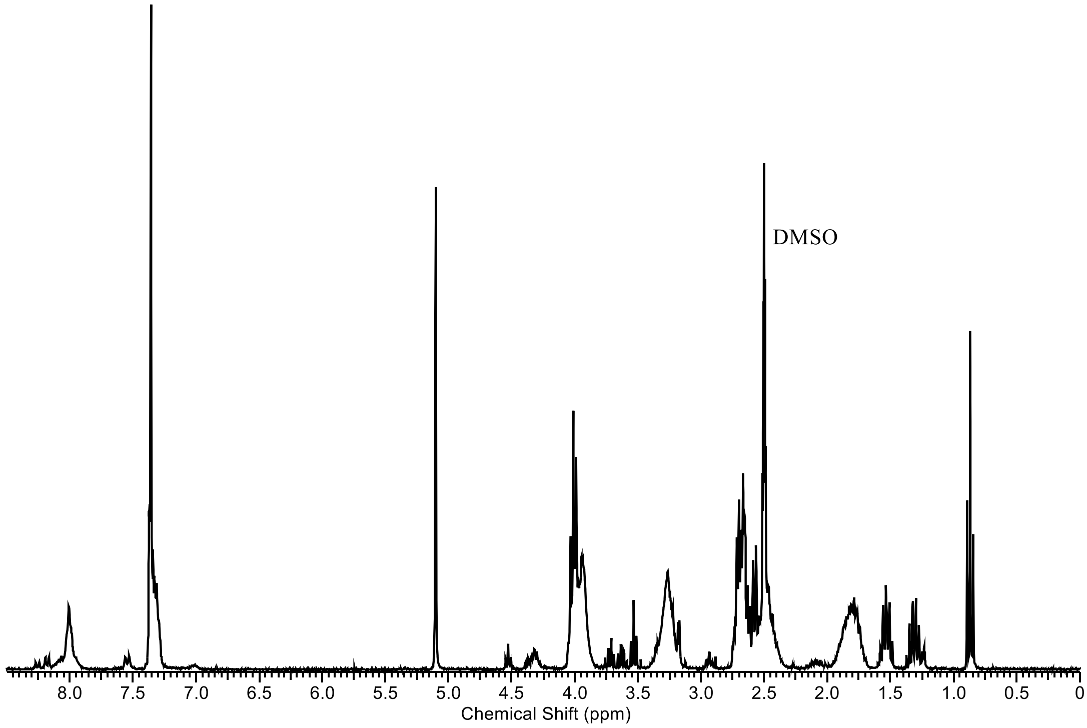

Supplementary Figure 22 | ^{1}H-NMR spectrum (500 MHz, DMSO-d_6) with peak assignment of **H2**.

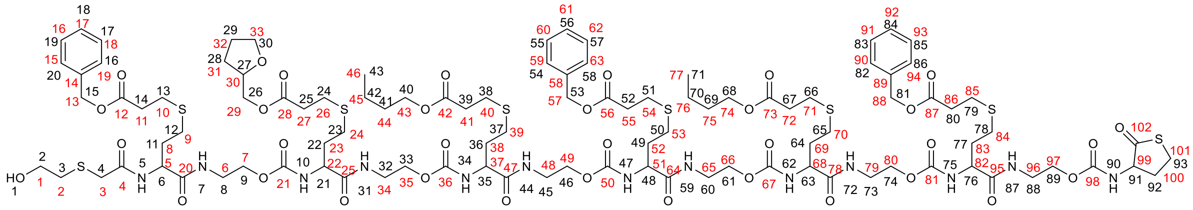

Supplementary Table 4 | determination of the ^{1}H- and ^{13}C-chemical shift values of **H2**

δ (ppm)	H	δ (ppm)	C
8.18	5	169.37	4
8.07	7	171.21	20
8.01	31, 44, 59, 72 and 87	171.69	25, 47, 64, 78 and 95
7.55	90	155.88	98
7.35	16-20, 54-58, and 82-86	127.93, 128.01 and 128.43	15-19, 59-63 and 90-94
7.32	10, 34, 47, 62 and 75	155.88	21, 36, 50, 67 and 81
5.09	15, 53 and 81	65.59	13, 57 and 88
4.53	1	/	/
4.35	91	59.88	99
4.31	6	51.99	5
4.02	40 and 68	63.72	43 and 74
4.01	26	66	29
4	27	75.75	30
3.97	89, 74, 61, 46, 33, 9 and 21, 35, 48, 63, 76	62.50 and 53.90	97, 80, 66, 49, 35, 7 and 22, 37, 51, 68, 82
3.73	30	67.42	33
3.63	30	67.42	33
3.53	2	60.45	1
3.3	93	26.4	101
3.27	8, 32, 45, 60, 73 and 88	38.15	6, 34, 48, 65, 79 and 96
3.17	4	34.5	3
2.69	13, 24, 38, 51, 66 and 79	26.02 and 26.10	10, 26, 40, 54, 71 and 85
2.64	3	34.54	2
2.6	14, 25, 39, 52, 67 and 80	34.27 and 34.30	11, 27, 41, 55, 72 and 86
2.46	12, 23, 37, 50, 65 and 78	27.49	9, 24, 39, 53, 70 and 84
2.43	92	29.84	100
2.09	92	29.84	100
1.89	28	27.49	31
1,81	11, 22, 36, 49, 64, 77 and 29	32.11 and 25.20	8, 23, 38, 52, 69, 83 and 32
1.55	28 and 41, 69	27.49 and 30.16	31 and 44, 75
1.33	42 and 70	18.59	45 and 76
0.88	43 and 71	13.53	46 and 77
		205.55	102
		171.49	42 and 73
		171.40	28
		171.34	12, 56 and 87
		136.06	14, 58 and 89

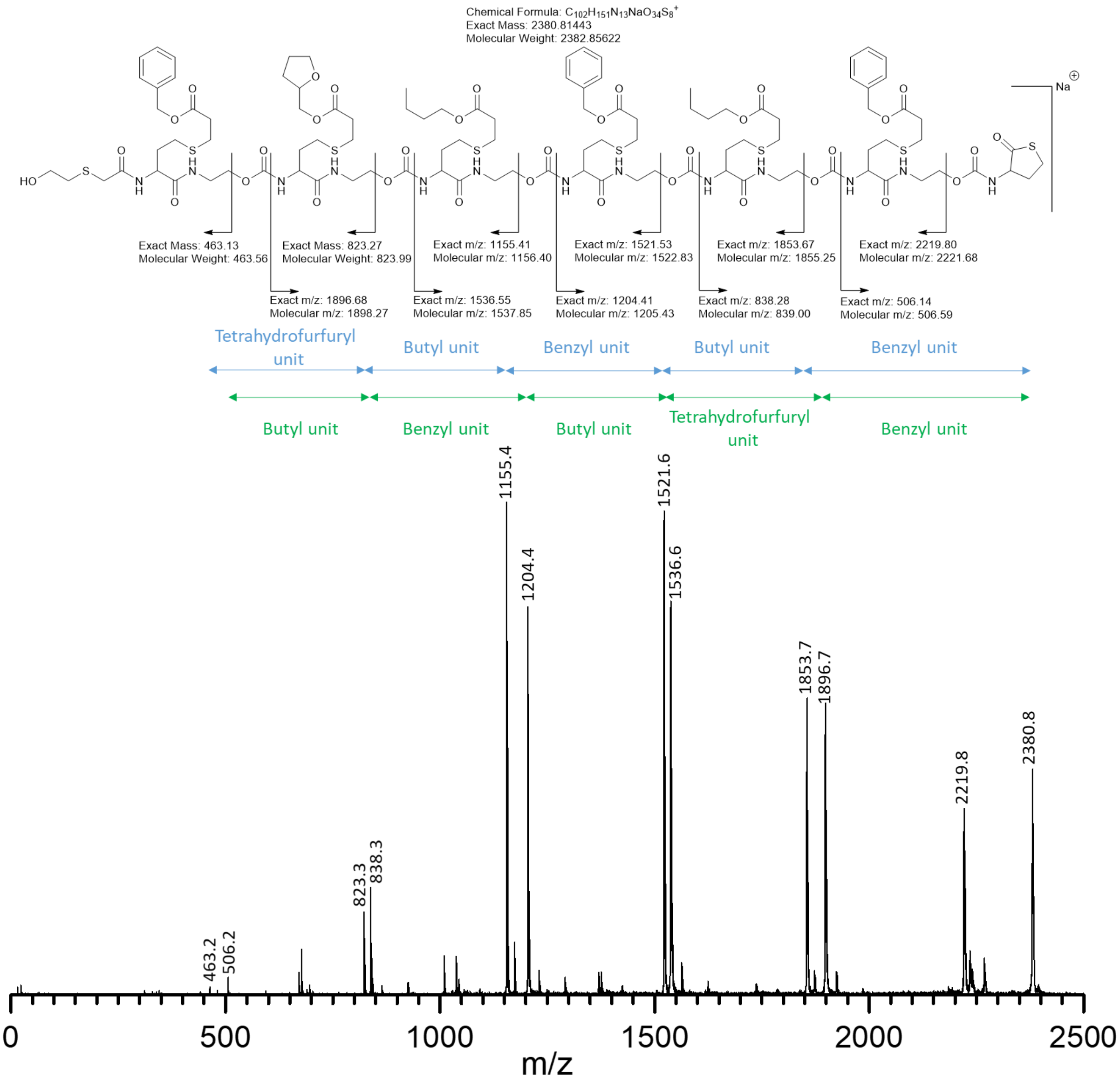

Supplementary Figure 23 | MALDI-MS/MS spectrum with peak assignment of **H2**.

*Characterization of **H3** using mass spectrometry (Supplementary Figure 24), NMR spectroscopy (Supplementary Figure 25) and MALDI-MS/MS analysis (Supplementary Figure 26).*

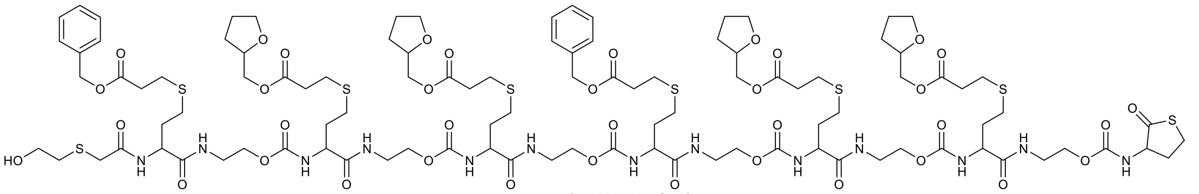

$C_{102}H_{153}N_{13}O_{37}S_8$
2407.8

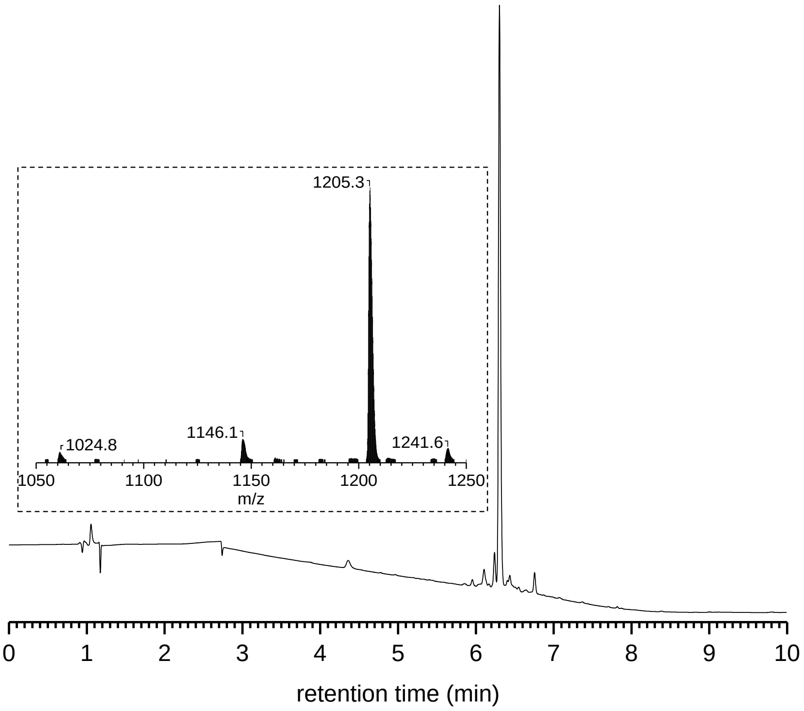

Supplementary Figure 24 | LC-ESI-MS analysis of **H3**. Insert: ESI-MS-spectrum of dominant species (positive mode).

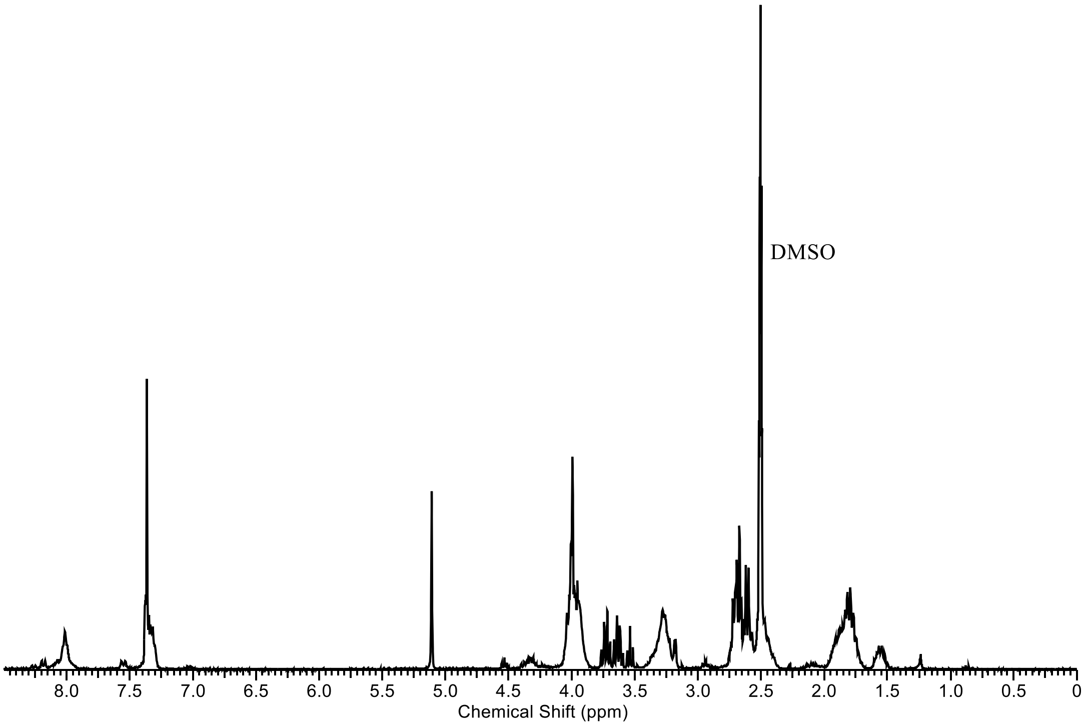

Supplementary Figure 25 | ^{1}H-NMR spectrum (500 MHz, DMSO-d_6) with peak assignment of **H3**.

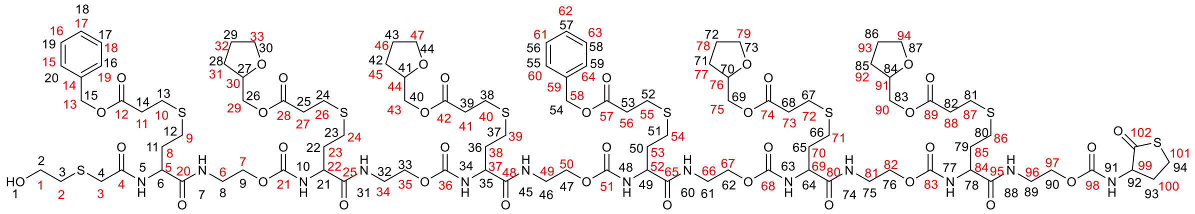

Supplementary Table 5 | determination of the ^{1}H- and ^{13}C-chemical shift values of **H3**

δ (ppm)	H	δ (ppm)	C
8.18	5	169.37	4
8.07	7	171.21	20
8.01	31, 45, 60, 74 and 88	171.69	25, 48, 65, 80 and 95
7.55	91	155.88	98
7.35	16-20 and 55-59	127.93, 128.01 and 128.43	15-19 and 60-64
7.32	10, 34, 48, 63 and 77	155.88	21, 36, 51, 68 and 83
5.09	15 and 54	65.59	13 and 58
4.53	1	/	/
4.35	92	59.88	99
4.31	6	51.99	5
4.01	26, 40, 69 and 83	66	29, 43, 75 and 90
4	27, 41, 70 and 84	75.75	30, 44, 76 and 91
3.97	90, 76, 62, 47, 33, 9 and 21, 35, 49, 64, 78	62.50 and 53.90	97, 82, 67, 50, 35, 7 and 22, 37, 52, 69, 84
3.73	30, 44, 73 and 87	67.42	33, 47, 79 and 94
3.63	30, 44, 73 and 87	67.42	33, 47, 79 and 94
3.53	2	60.45	1
3.3	94	26.4	101
3.27	8, 32, 46, 61, 75 and 89	38.15	6, 34, 49, 66, 81 and 96
3.17	4	34.5	3
2.69	13, 24, 38, 52, 67 and 81	26.04	10, 26, 40, 55, 72 and 87
2.64	3	34.54	2
2.6	14, 25, 39, 53, 68 and 82	34.27 and 34.30	11, 27, 41, 56, 73 and 88
2.46	12, 23, 37, 51, 66 and 80	27.49	9, 24, 39, 54, 71 and 86
2.43	93	29.84	100
2.09	93	29.84	100
1.89	28, 42, 71 and 85	27.49	31, 45, 77 and 92
1,81	11, 22, 36, 50, 65 and 79; 29, 43, 72 and 86	32.11 and 25.20	8, 23, 38, 53, 70 and 85; 32, 46, 78 and 93
1.55	28, 42, 71 and 85	27.49	31, 45, 77 and 92
		205.55	102
		171.4	28, 42, 74 and 89
		171.34	12 and 57
		136.06	14 and 59

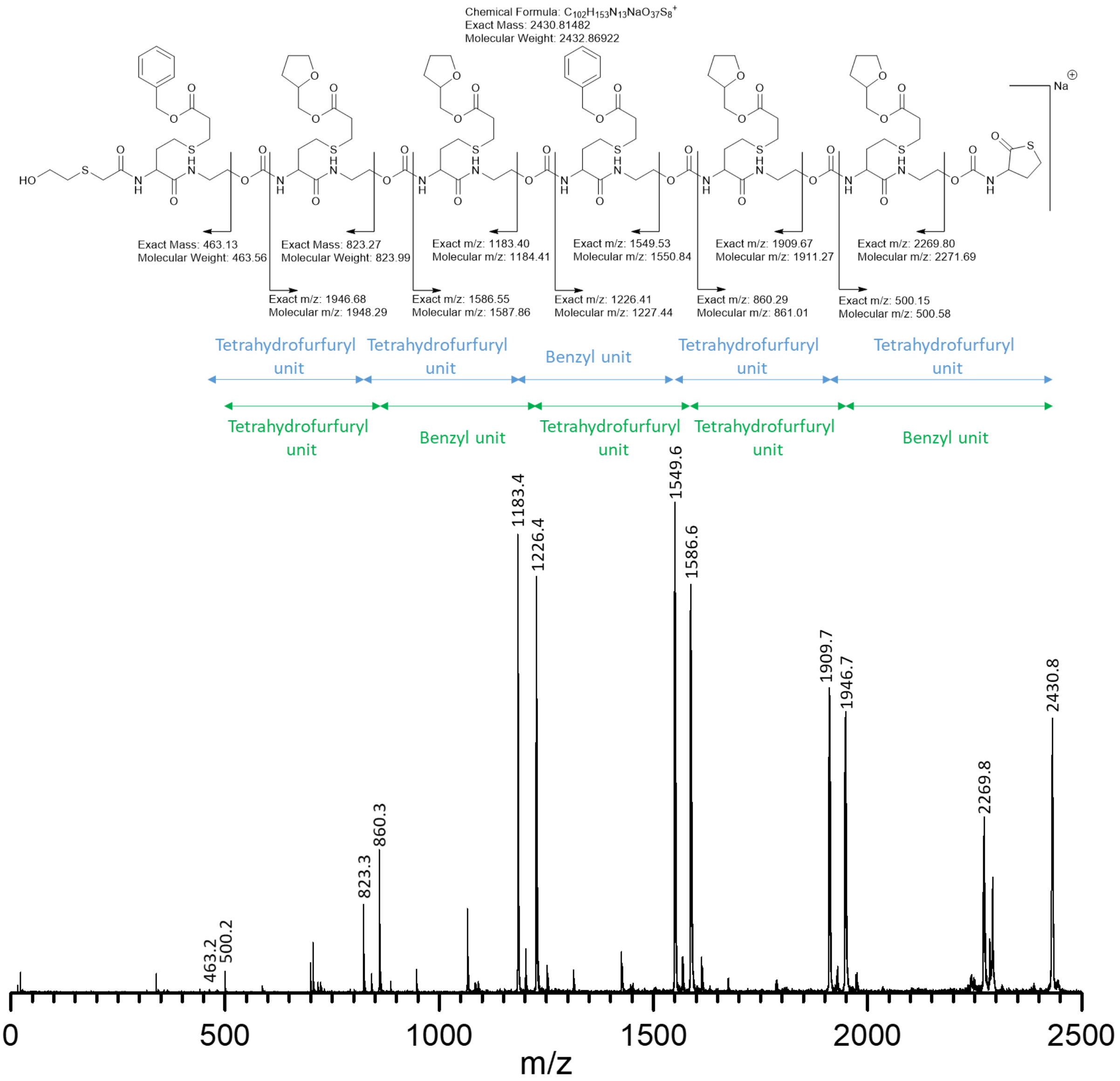

Supplementary Figure 26 | MALDI-MS/MS spectrum with peak assignment of **H3**.

*Characterization of **H4** using mass spectrometry (Supplementary Figure 27), NMR spectroscopy (Supplementary Figure 28) and MALDI-MS/MS analysis (Supplementary Figure 29).*

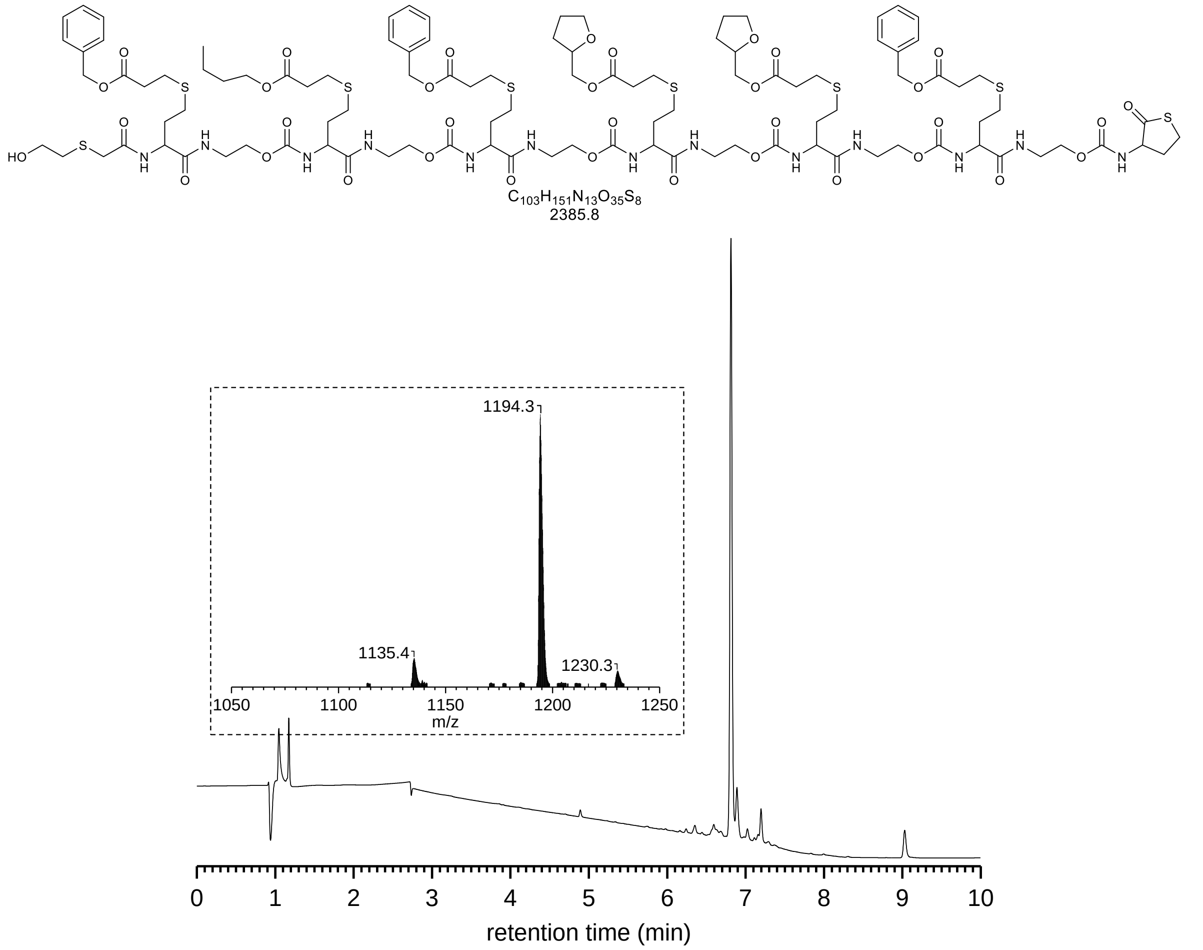

Supplementary Figure 27 | LC-ESI-MS analysis of **H4**. Insert: ESI-MS-spectrum of dominant species (positive mode).

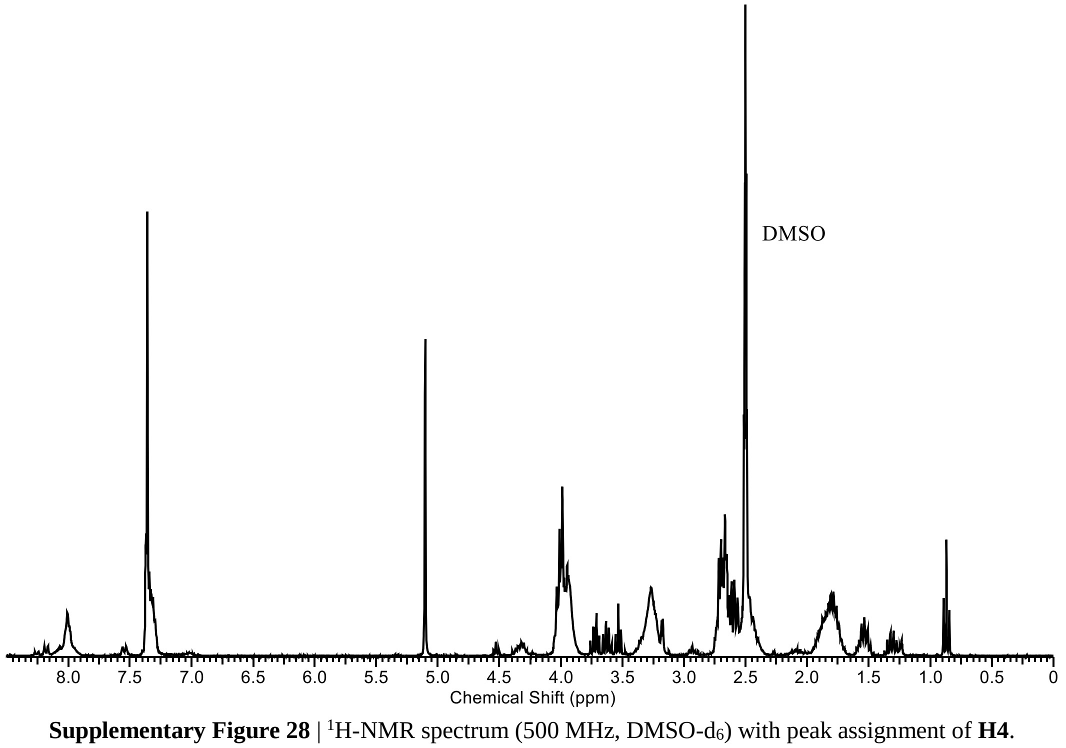

Supplementary Figure 28 | ^{1}H-NMR spectrum (500 MHz, DMSO-d$_6$) with peak assignment of **H4**.

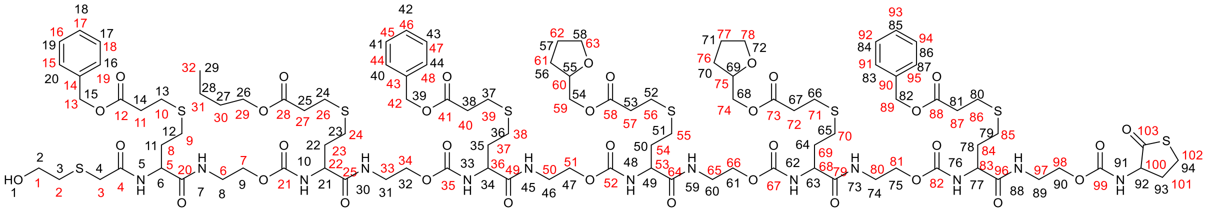

Supplementary Table 6 | determination of the ^{1}H- and ^{13}C-chemical shift values of **H4**

δ (ppm)	H	δ (ppm)	C
8.18	5	169.37	4
8.07	7	171.21	20
8.01	30, 45, 59, 73 and 88	171.69	25, 49, 64, 79 and 96
7.55	91	155.88	99
7.35	16-20, 40-44, and 83-87	127.93, 128.01 and 128.43	15-19, 44-48 and 91-95
7.32	10, 33, 48, 62 and 76	155.88	21, 35, 52, 67 and 82
5.09	15, 39 and 82	65.59	13, 42 and 89
4.53	1	/	/
4.35	92	59.88	100
4.31	6	51.99	5
4.02	26	63.72	29
4.01	54 and 68	66	59 and 74
4	55 and 69	75.75	60 and 75
3.97	90, 75, 61, 47, 32, 9 and 21, 34, 49, 63, 77	62.50 and 53.90	98, 81, 66, 51, 34, 7 and 22, 36, 53, 68, 83
3.73	58 and 72	67.42	63 and 78
3.63	58 and 72	67.42	63 and 78
3.53	2	60.45	1
3.3	94	26.4	102
3.27	8, 31, 46, 60, 74 and 89	38.15	6, 33, 50, 65, 80 and 97
3.17	4	34.5	3
2.69	13, 24, 37, 52, 66 and 80	26.02 and 26.10	10, 26, 39, 56, 71 and 86
2.64	3	34.54	2
2.6	14, 25, 38, 53, 67 and 81	34.27 and 34.30	11, 27, 40, 57, 72 and 87
2.46	12, 23, 36, 51, 65 and 79	27.49	9, 24, 38, 55, 70 and 85
2.43	93	29.84	101
2.09	93	29.84	101
1.89	56 and 70	27.49	61 and 76
1,81	11, 22, 35, 50, 64, 78; 57, 71	32.11; 25.20	8, 23, 37, 54, 69, 84; 62, 77
1.55	56, 70 and 27	27.49 and 30.16	61, 76 and 30
1.33	28	18.59	31
0.88	29	13.53	32
		205.55	103
		171.49	28
		171.40	58 and 73
		171.34	12, 41 and 88
		136.06	14, 43 and 90

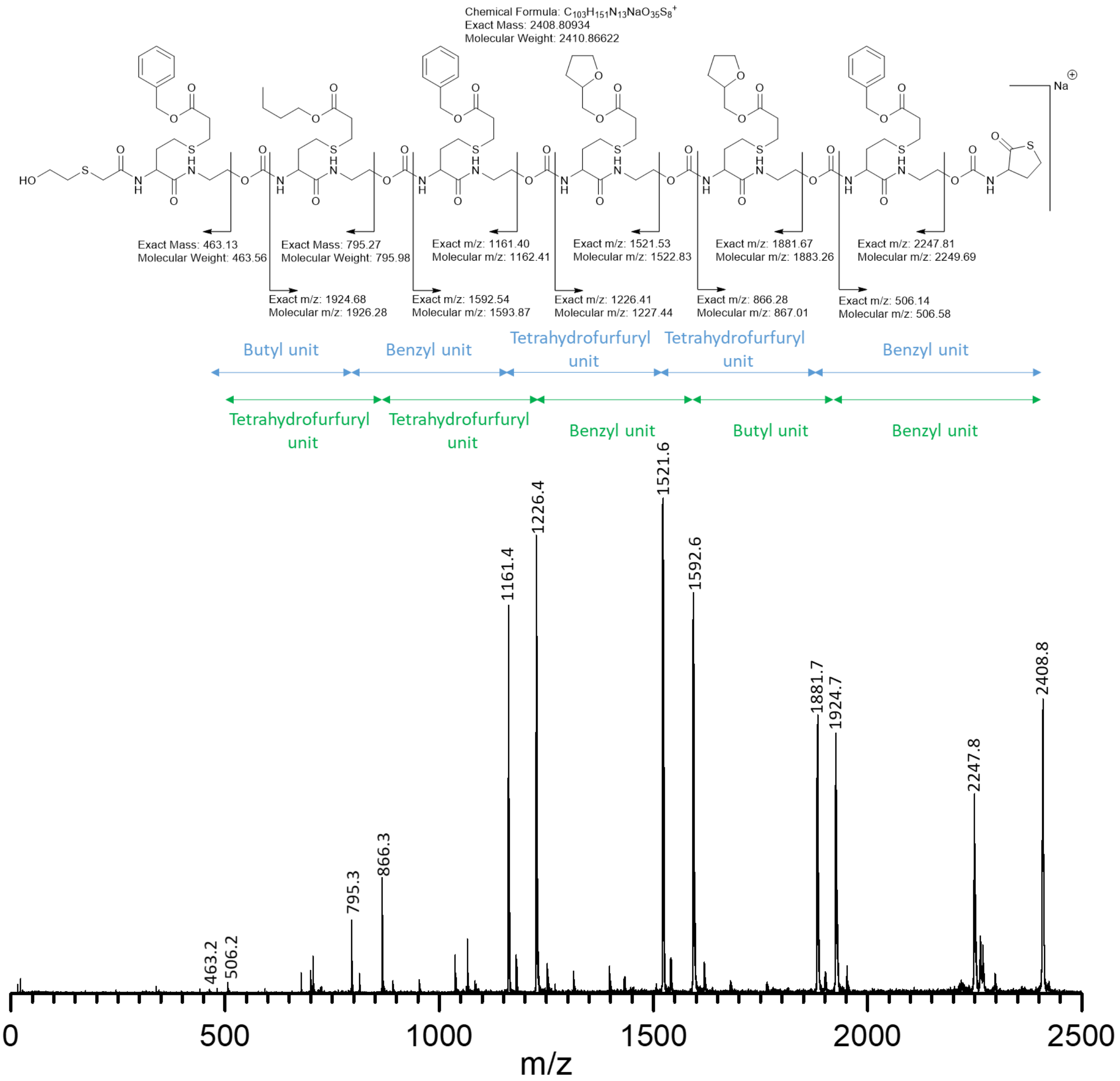

Supplementary Figure 29 | MALDI-MS/MS spectrum with peak assignment of **H4**.

*Characterization of **H5** using mass spectrometry (Supplementary Figure 30), NMR spectroscopy (Supplementary Figure 31) and MALDI-MS/MS analysis (Supplementary Figure 32).*

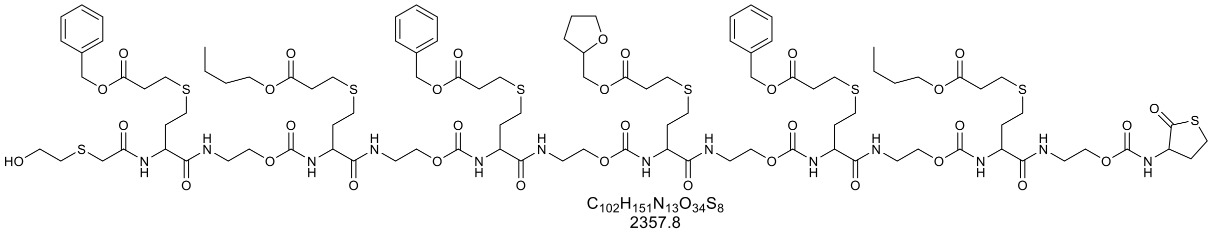

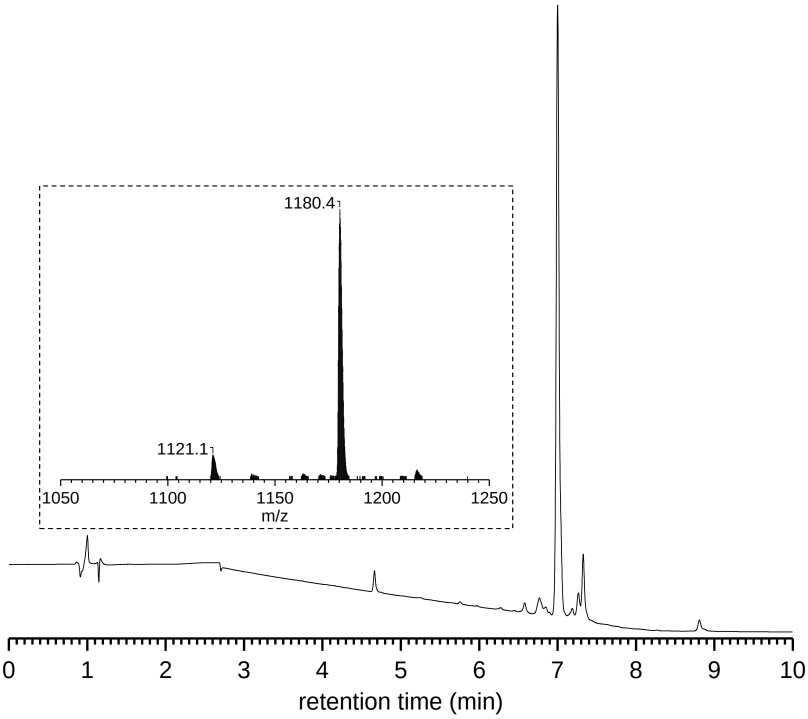

Supplementary Figure 30 | LC-ESI-MS analysis of **H5**. Insert: ESI-MS-spectrum of dominant species (positive mode).

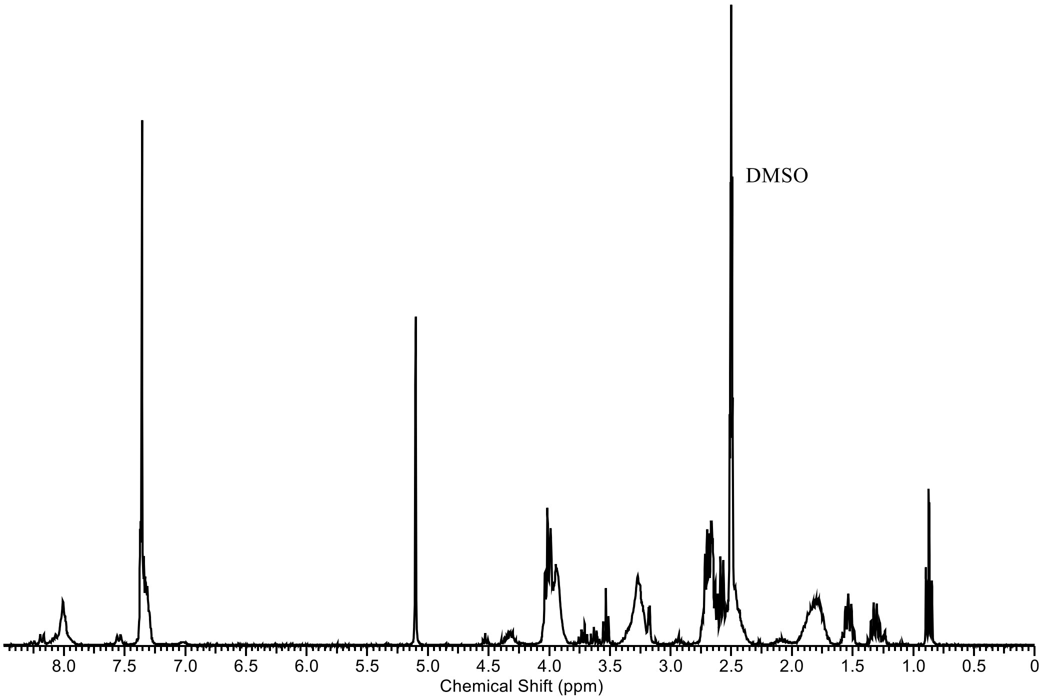

Supplementary Figure 31 | ^{1}H-NMR spectrum (500 MHz, DMSO-d$_6$) with peak assignment of **H5**.

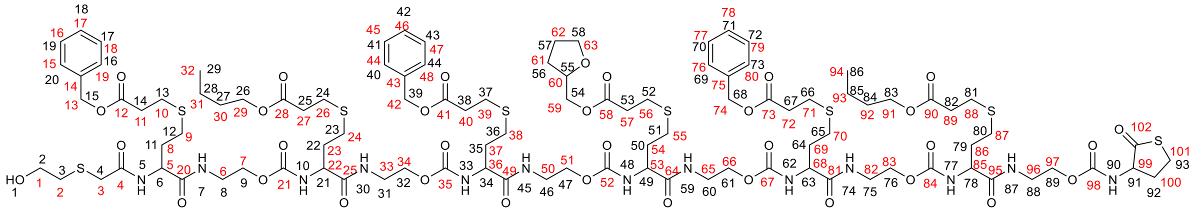

Supplementary Table 7 | determination of the ^{1}H- and ^{13}C-chemical shift values of **H5**

δ (ppm)	H	δ (ppm)	C
8.18	5	169.37	4
8.07	7	171.21	20
8.01	30, 45, 59, 74 and 87	171.69	25, 49, 64, 81 and 95
7.55	90	155.88	98
7.35	16-20, 40-44, and 69-73	127.93, 128.01 and 128.43	15-19, 44-48 and 76-80
7.32	10, 33, 48, 62 and 77	155.88	21, 35, 52, 67 and 84
5.09	15, 39 and 68	65.59	13, 42 and 74
4.53	1	/	/
4.35	91	59.88	99
4.31	6	51.99	5
4.02	26 and 83	63.72	29 and 91
4.01	54	66	59
4	55	75.75	60
3.97	89, 76, 61, 47, 32, 9 and 21, 34, 49, 63, 78	62.50 and 53.90	97, 83, 66, 51, 34, 7 and 22, 36, 53, 68, 85
3.73	58	67.42	63
3.63	58	67.42	63
3.53	2	60.45	1
3.3	93	26.4	101
3.27	8, 31, 46, 60, 75 and 88	38.15	6, 33, 50, 65, 82 and 96
3.17	4	34.5	3
2.69	13, 24, 37, 52, 66 and 81	26.02 and 26.10	10, 26, 39, 56, 71 and 88
2.64	3	34.54	2
2.6	14, 25, 38, 53, 67 and 82	34.27 and 34.30	11, 27, 40, 57, 72 and 89
2.46	12, 23, 36, 51, 65 and 80	27.49	9, 24, 38, 55, 70 and 87
2.43	92	29.84	100
2.09	92	29.84	100
1.89	56	27.49	61
1,81	11, 22, 35, 50, 64, 79 and 57	32.11 and 25.20	8, 23, 37, 54, 69, 86 and 62
1.55	56 and 27, 84	27.49 and 30.16	61 and 30, 92
1.33	28 and 85	18.59	31 and 93
0.88	29 and 86	13.53	32 and 94
		205.55	102
		171.49	28 and 90
		171.40	58
		171.34	12, 41 and 73
		136.06	14, 43 and 75

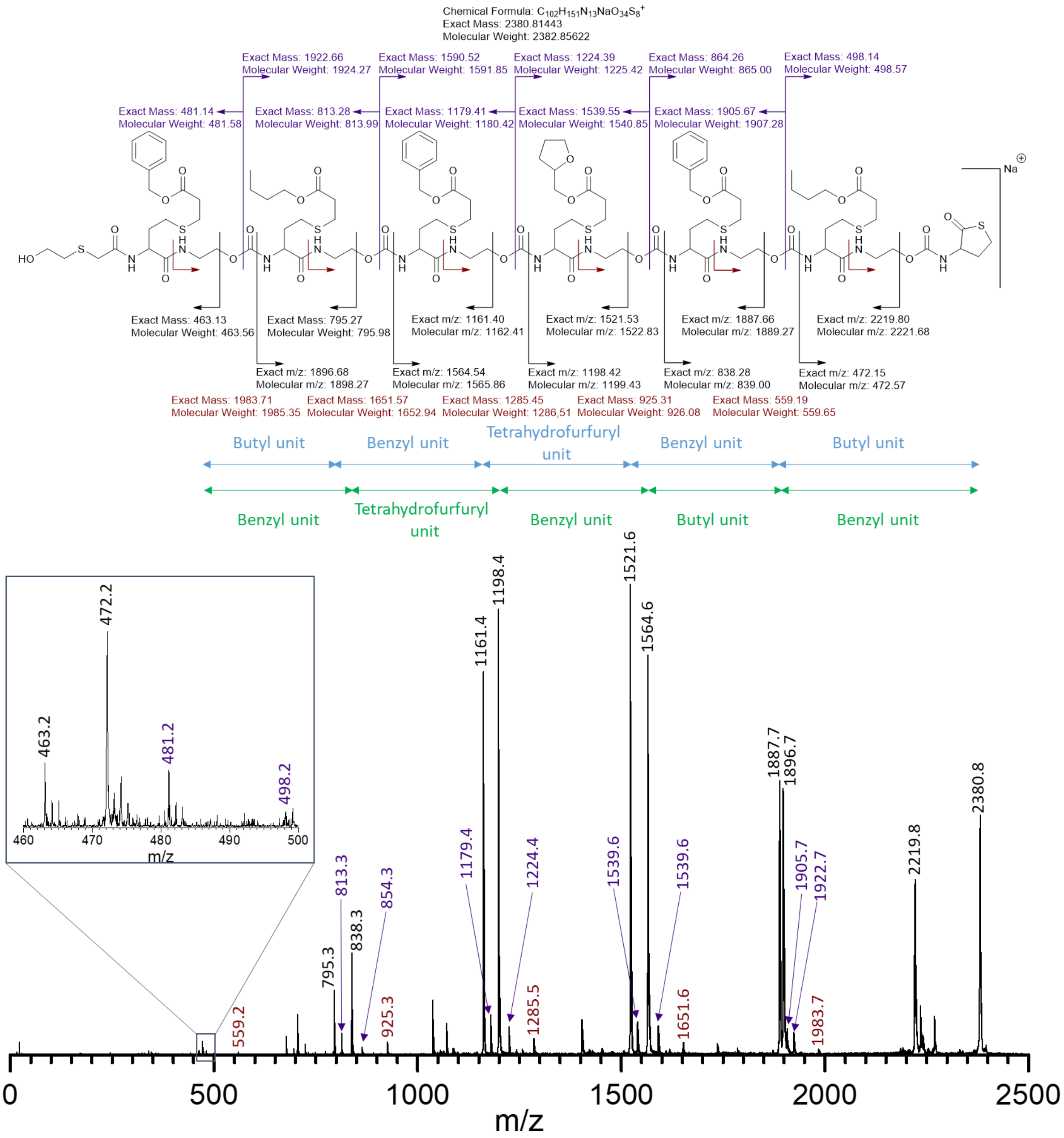

Supplementary Figure 32 | MALDI-MS/MS spectrum with peak assignment of **H5**.

*Characterization of **H6** using mass spectrometry (Supplementary Figure 33), NMR spectroscopy (Supplementary Figure 34) and MALDI-MS/MS analysis (Supplementary Figure 35).*

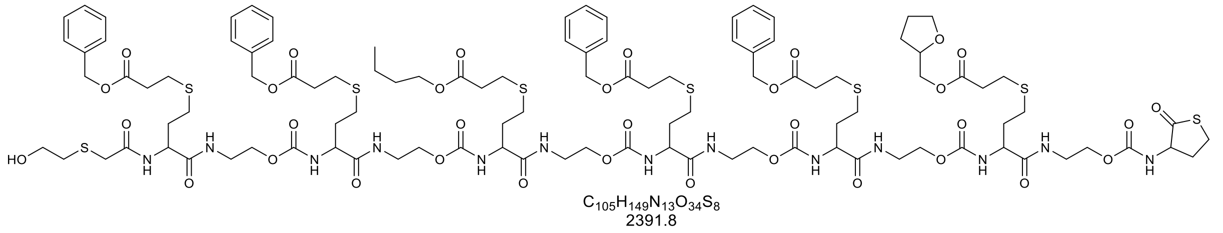

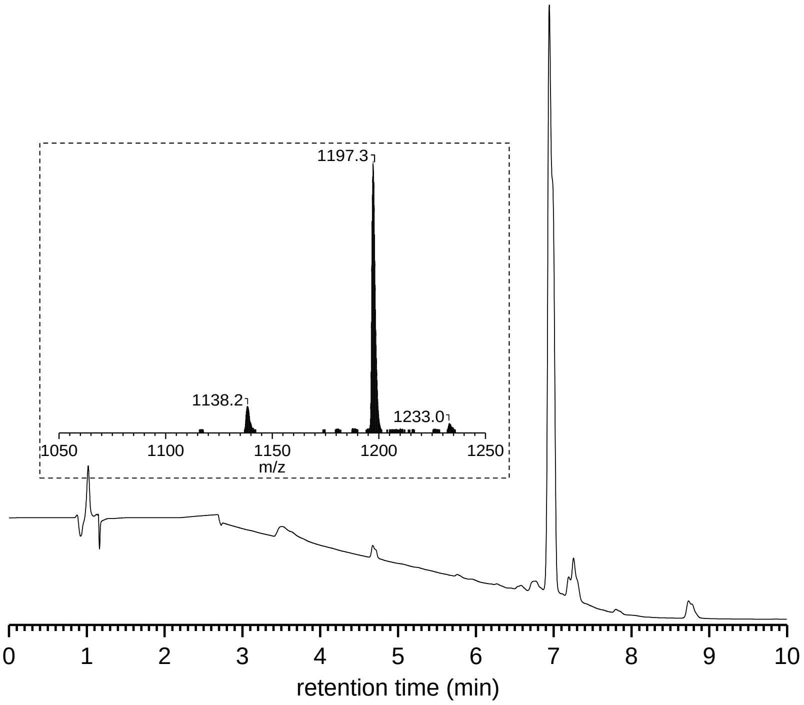

Supplementary Figure 33 | LC-ESI-MS analysis of **H6**. Insert: ESI-MS-spectrum of dominant species (positive mode).

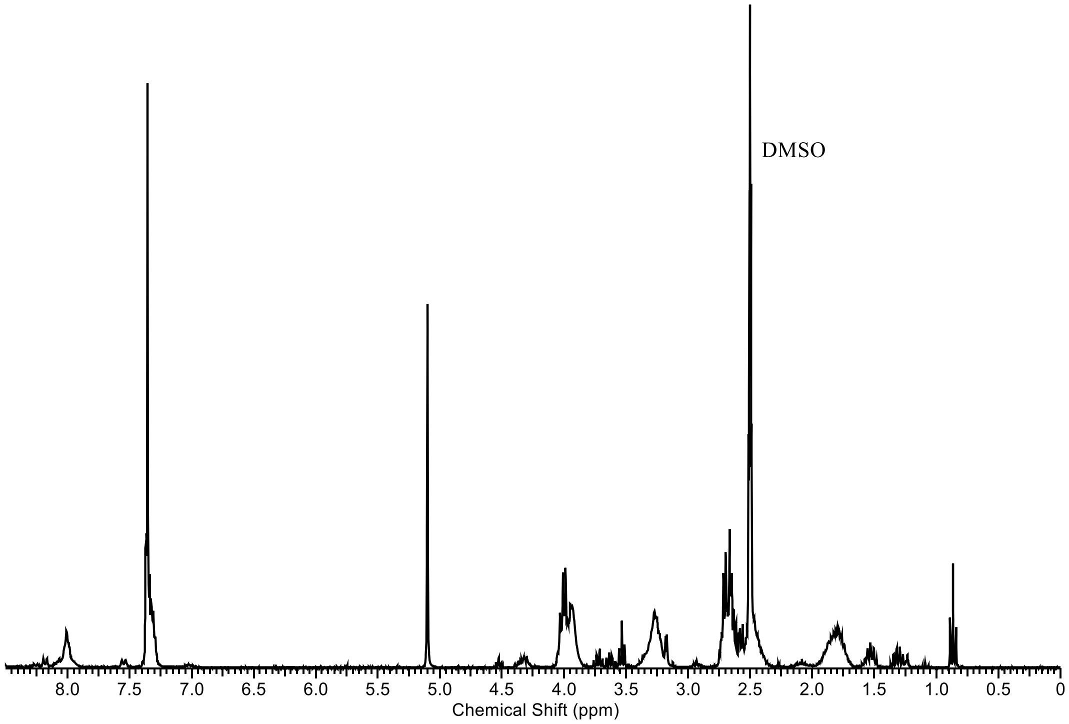

Supplementary Figure 34 | ^{1}H-NMR spectrum (500 MHz, DMSO-d_6) with peak assignment of **H6**.

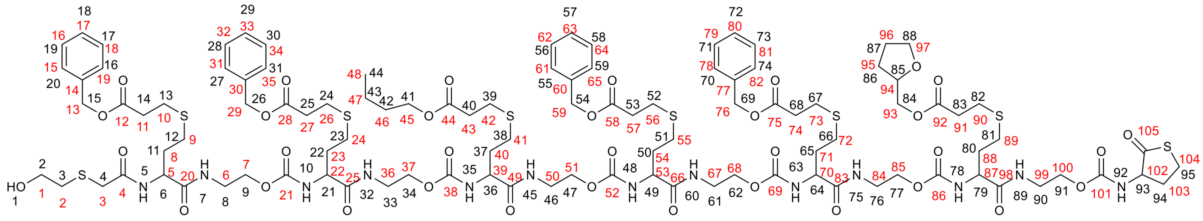

Supplementary Table 8 | determination of the ^{1}H- and ^{13}C-chemical shift values of **H6**

δ (ppm)	H	δ (ppm)	C
8.18	5	169.37	4
8.07	7	171.21	20
8.01	32, 45, 60, 75 and 89	171.69	25, 49, 66, 83 and 98
7.55	92	155.88	101
7.35	16-20, 27-31, 55-59, 70-74	127.93, 128.01 and 128.43	15-19, 31-35, 61-65, 78-82
7.32	10, 35, 48, 63 and 78	155.88	21, 38, 52, 69 and 86
5.09	15, 26, 54 and 69	65.59	13, 29, 59 and 76
4.53	1	/	/
4.35	93	59.88	102
4.31	6	51.99	5
4.02	41	63.72	45
4.01	84	66	93
4	85	75.75	94
3.97	91, 77, 62, 47, 34, 9 and 21, 39, 49, 64, 79	62.50 and 53.90	100, 85, 68, 51, 37, 7 and 22, 36, 53, 70, 87
3.73	88	67.42	97
3.63	88	67.42	97
3.53	2	60.45	1
3.3	95	26.4	104
3.27	8, 33, 46, 61, 76 and 90	38.15	6, 36, 50, 67, 84 and 99
3.17	4	34.5	3
2.69	13, 24, 39, 52, 67 and 82	26.02 and 26.10	10, 26, 42, 56, 73 and 90
2.64	3	34.54	2
2.6	14, 25, 40, 53, 68 and 83	34.27 and 34.30	11, 27, 43, 57, 74 and 91
2.46	12, 23, 38, 51, 66 and 81	27.49	9, 24, 41, 55, 72 and 89
2.43	94	29.84	103
2.09	94	29.84	103
1.89	86	27.49	95
1,81	11, 22, 37, 50, 65, 80 and 87	32.11 and 25.20	8, 23, 40, 54, 71, 88 and 96
1.55	86 and 42	27.49 and 30.16	95 and 46
1.33	43	18.59	47
0.88	44	13.53	48
		205.55	105
		171.49	44
		171.40	92
		171.34	12, 28, 58 and 75
		136.06	14, 30, 60 and 77

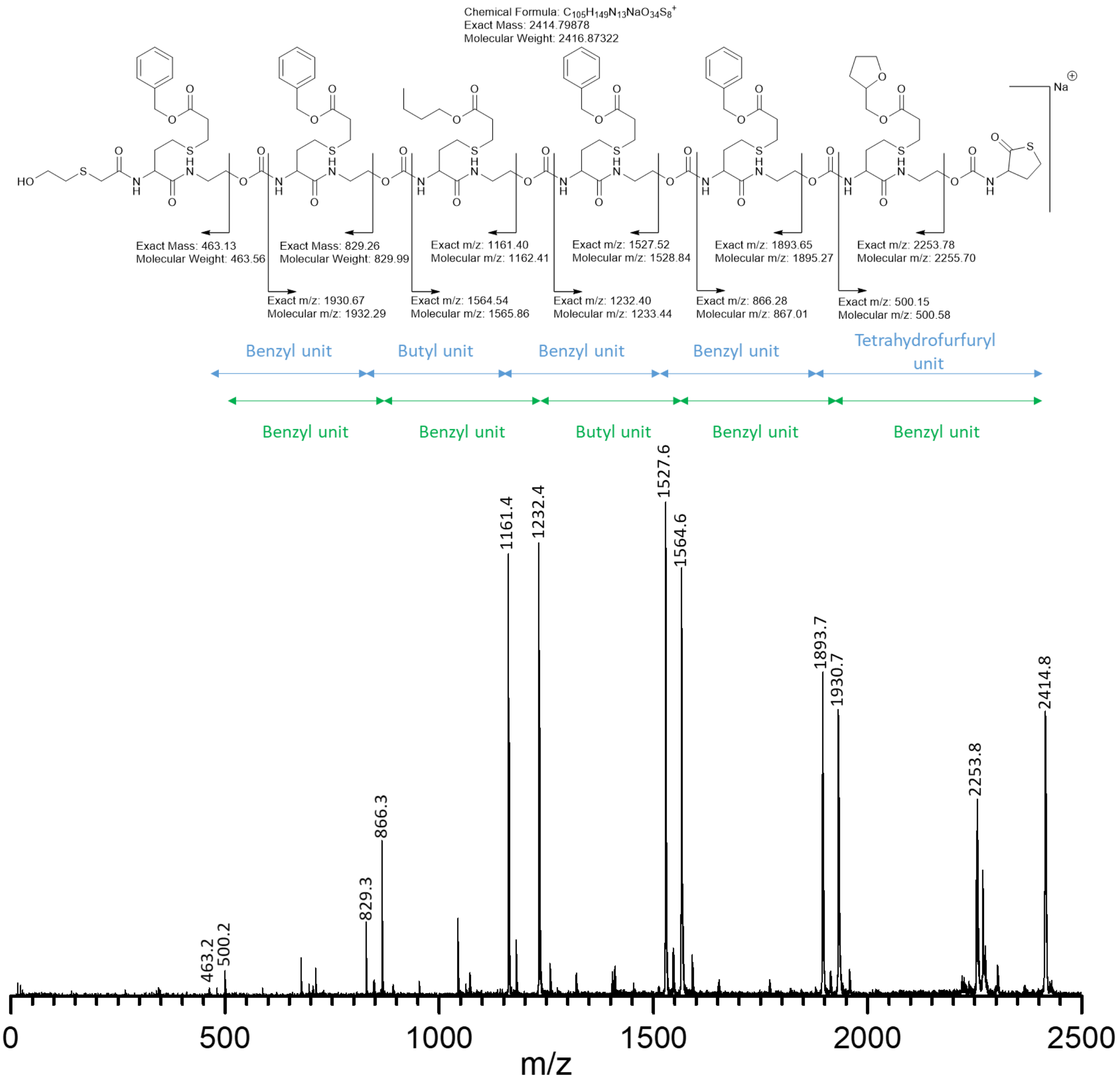

Supplementary Figure 35 | MALDI-MS/MS spectrum with peak assignment of **H6**.

Sentence

Supplementary Table 9 | The alphabet (the different functionalities) used to write the sentence '1To 2write 3or 4not 5to 6write 7on 8oligos?' on sequence-defined poly(amide-urethane)s

.Symbol	Acrylate functionality	Symbol	Acrylate functionality
1	2-cyanoethyl	I	Ethyl
2	2-(2-ethoxyethoxy)ethyl	L	Cyclohexyl
3	Ethylhexyl	N	Isobornyl
4	Nonyl	O	Methyl
5	Citronellyl	R	Tetrahydrofurfuryl
6	Heptyl	S	Isoamyl
7	Acetylethyl	T	Benzyl
8	Propargyl	W	Butyl
E	Propyl	?	2-ethoxyethyl
G	2-methoxyethyl		

*Characterization of **A1TO** using mass spectrometry (Supplementary Figure 36), NMR spectroscopy (Supplementary Figure 37) and MALDI-MS/MS analysis (Supplementary Figure 38).*

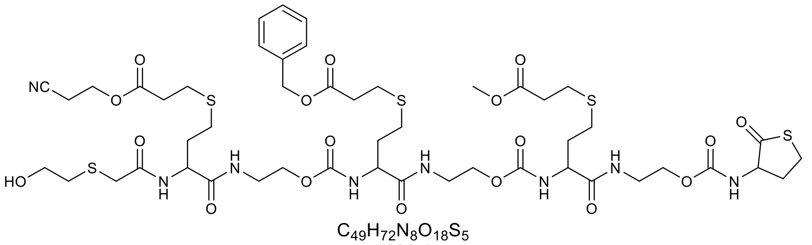

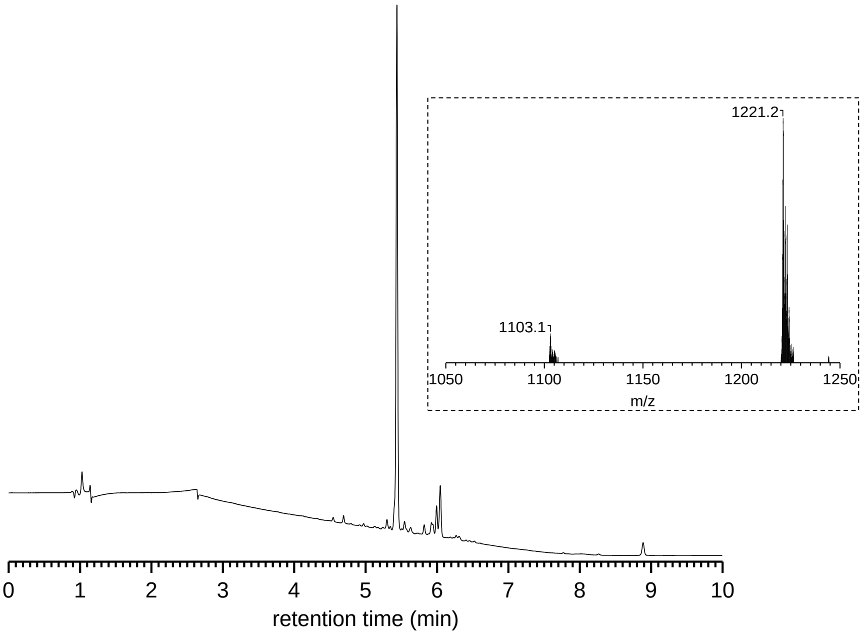

Supplementary Figure 36 | LC-ESI-MS analysis of **A1TO**. Insert: ESI-MS-spectrum of dominant species (positive mode).

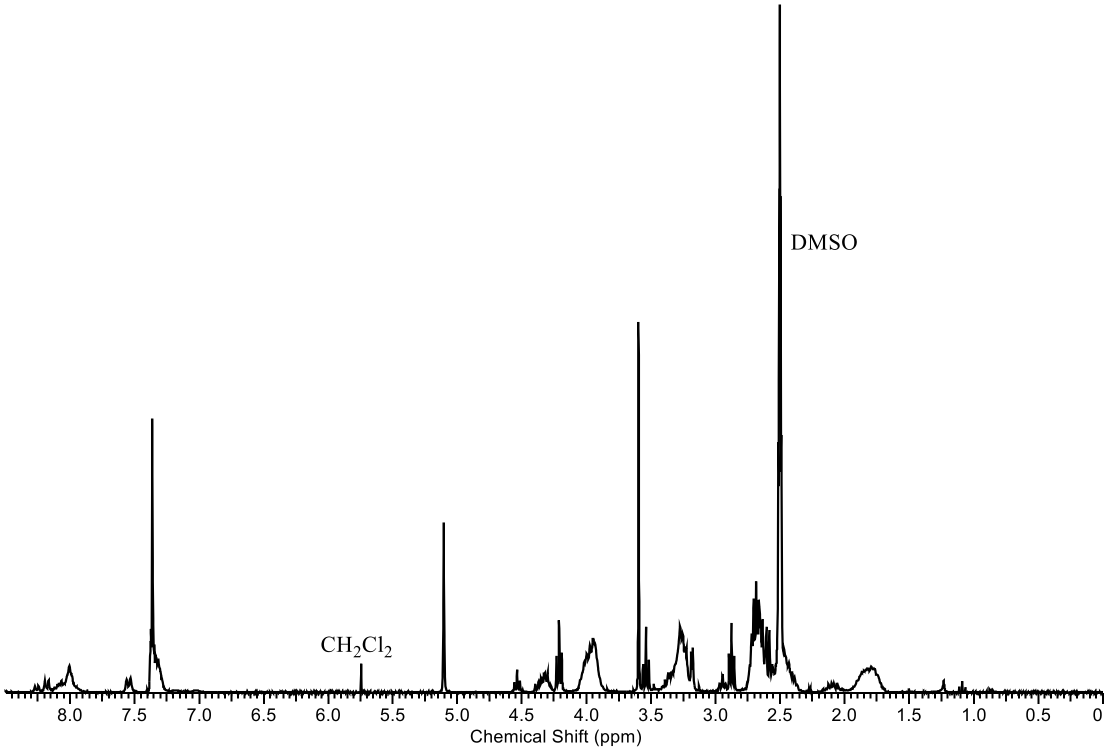

Supplementary Figure 37 | ^{1}H-NMR spectrum (500 MHz, DMSO-d$_6$) with peak assignment of **A1TO**.

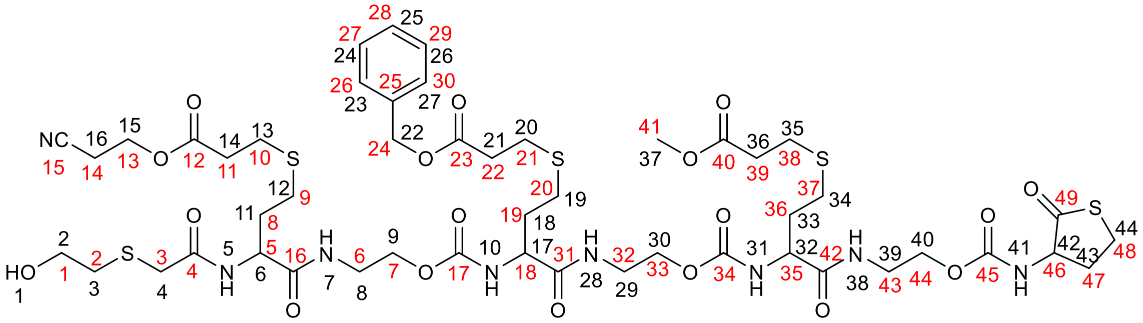

Supplementary Table 10 | determination of the ^{1}H- and ^{13}C-chemical shift values of **A1TO**

δ (ppm)	H	δ (ppm)	C
8.18	5	169.37	4
8.07	7	171.21	16
8.01	28 and 38	171.69	31 and 42
7.55	41	155.88	45
7.36	23-27	127.9, 127.99 and 128.42	26-30
7.33	10 and 31	171.66	17 and 34
5.11	22	65.59	24
4.53	1	/	/
4.35	42	59.84	46
4.31	6	51.81	5
4.21	15	59.09	13
3.97	40, 30, 9 and 17, 32	62.51 and 53.86	44, 33, 7 and 18, 35
3.6	37	51.41	41
3.53	2	60.45	1
3.3	44	26.36	48
3.27	8, 29 and 39	38.15	6, 32 and 43
3.17	4	34.5	3
2.87	16	17.39	14
2.72	20	26.11	21
2.69	13 and 35	26.01	10 and 38
2.64	3, 14 and 21	34.54, 34.33 and 34.26	2, 11 and 22
2.6	36	34.11	39
2.46	12, 19 and 34	27.33 and 27.48	9, 20 and 37
2.43	43	29.8	47
2.09	43	29.8	47
1.81	11, 18 and 33	32.12	8, 19 and 36
		171.34	23
		136.06	25
		205.55	49
		171.93	40
		171.15	12
		118.45	15

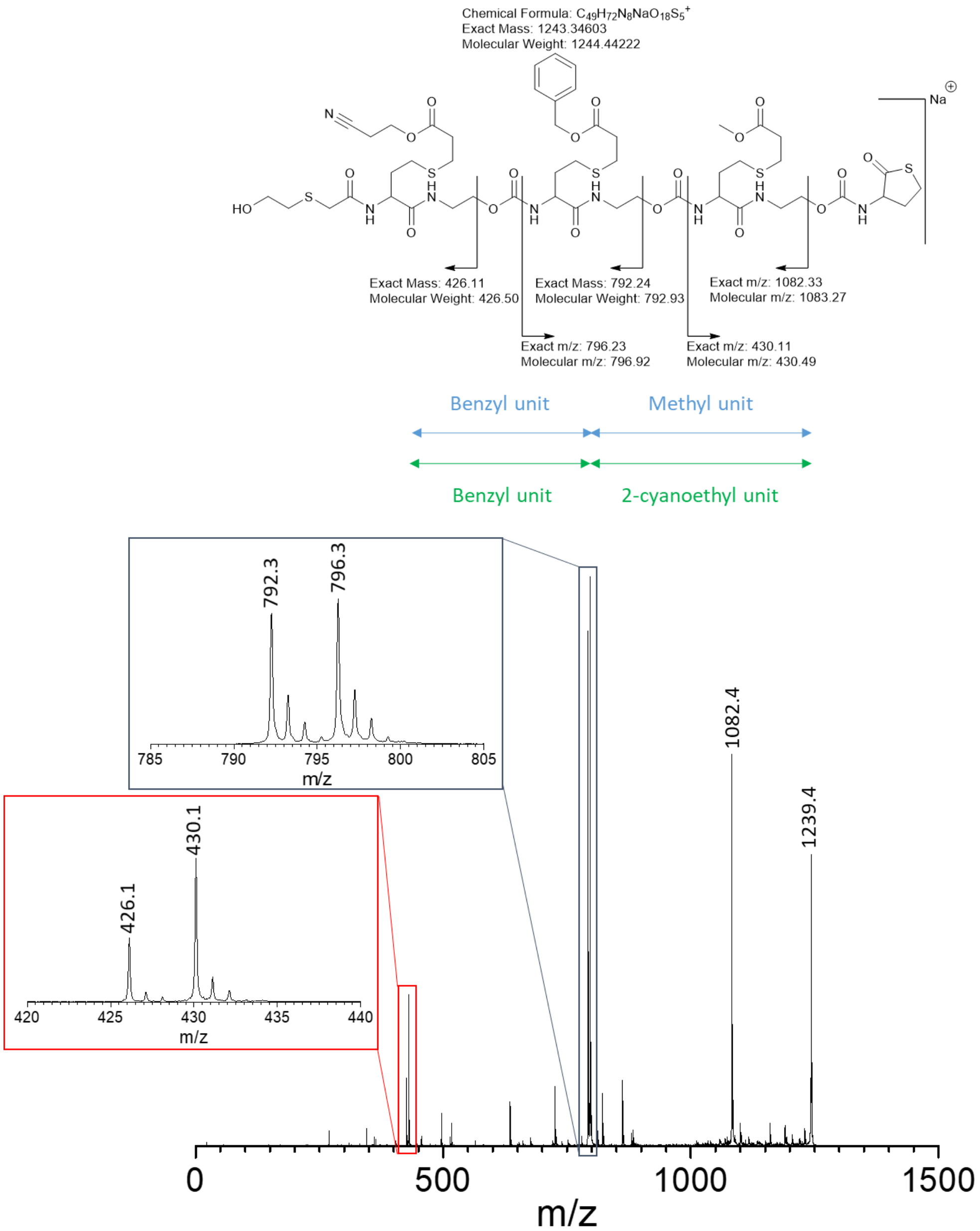

Supplementary Figure 38 | MALDI-MS/MS spectrum with peak assignment of **A1TO**.

*Characterization of **A2WRITE** using mass spectrometry (Supplementary Figure 39), NMR spectroscopy (Supplementary Figure 40) and MALDI-MS/MS analysis (Supplementary Figure 41).*

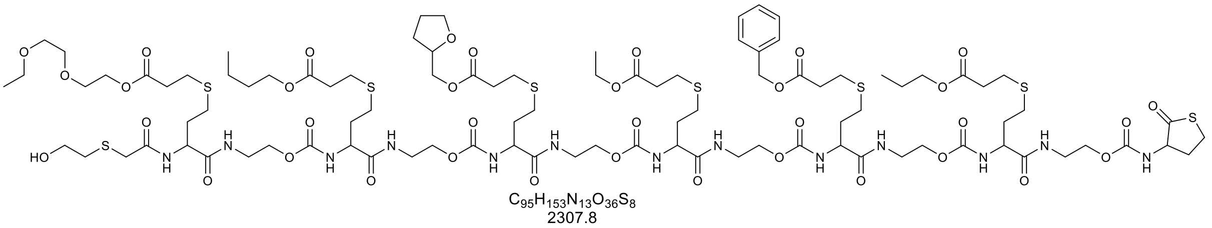

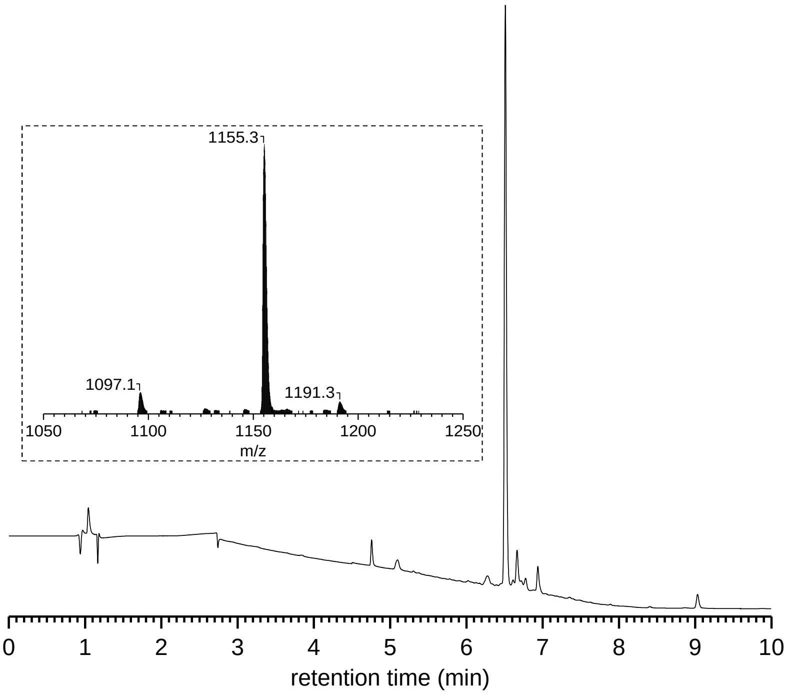

Supplementary Figure 39 | LC-ESI-MS analysis of **A2WRITE**. Insert: ESI-MS-spectrum of dominant species (positive mode).

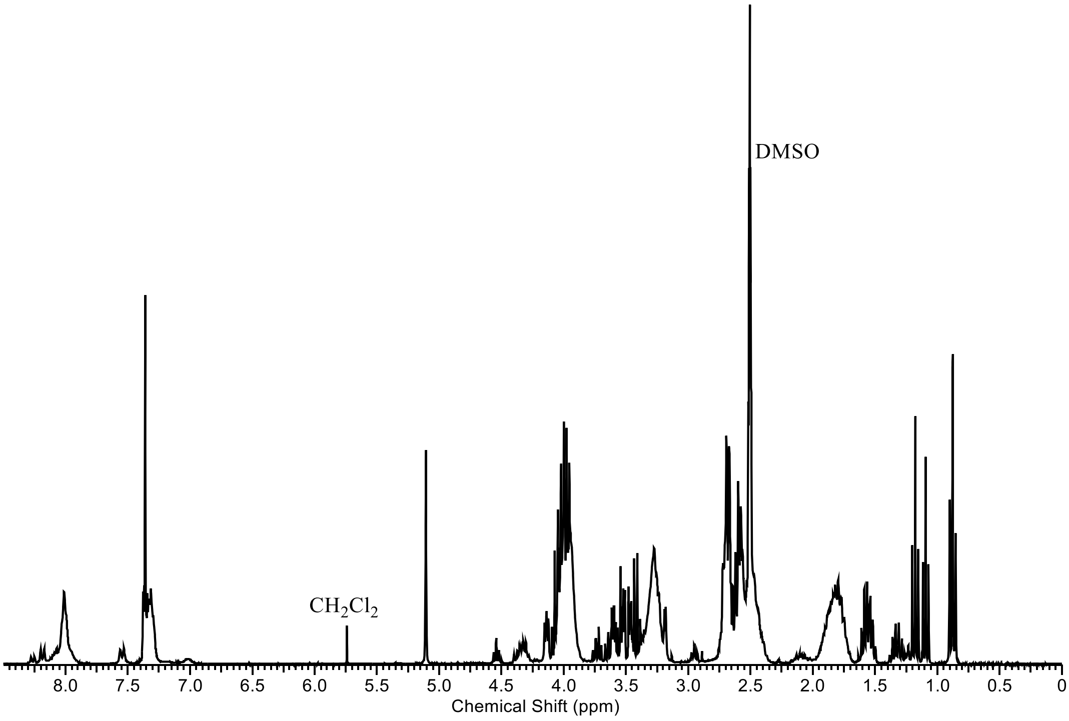

Supplementary Figure 40 | ^{1}H-NMR spectrum (500 MHz, DMSO-d6) with peak assignment of **A2WRITE**.

Supplementary Table 11 | determination of the ^{1}H- and ^{13}C-chemical shift values of **A2WRITE**

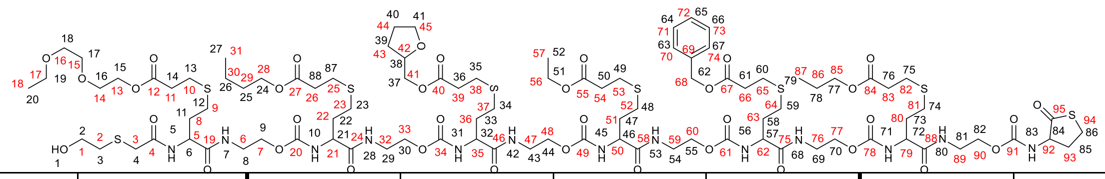

δ (ppm)	H	δ (ppm)	C	δ (ppm)	H	δ (ppm)	C	δ (ppm)	H	δ (ppm)	C
8.18	5	169.37	4	3.97	82, 70, 55, 44, 30, 9 and 21, 32, 46, 57, 72	62.50 and 53.90	90, 77, 60, 48, 33, 7 and 21, 35, 50, 62, 79	2.09/2.43	85	29.8	93
8.07	7	171.21	19					1.81	11, 22, 33, 47, 58 and 73; 39 and 40	32.05 and 32.18; 27.46 and 25.17	8, 22, 36, 51, 63 and 80; 43 and 44
8.01	28, 42, 53, 68 and 80	171.69	24, 46, 58, 75 and 88	3.73/3.63	41	67.42	45				
				3.59	16	68.30	14	1.55	39, 25 and 78	27.46, 30.16 and 21.65	43, 29 and 86
7.55	83	155.88	91	3.53	2	60.45	1				
7.36	63-67	127.9, 127.99 and 128.41	70-74	3.49	17 and 18	69.21/69.88	15 and 16	1.33	26	18.59	30
				3.41	19	65.66	17	1.18	52	14.11	57
7.33	10, 31, 45, 56 and 71	155.88	20, 34, 49, 61 and 78	3.3	86	26.36	94	1.09	20	15.12	18
				3.27	8, 29, 43, 54, 69 and 81	38.15 /33.86	6, 32, 47, 59, 76 and 89	0.88	27 and 79	13.53 and 10.26	31 and 87
5.11	62	65.61	68								
4.53	1	/	/	3.17	4	34.5	3				
4.35	84	59.88	92	2.72	60	26.11	65				
4.31	6	51.81	5	2.69	13, 87, 35, 49 and 75	26.09/26.15	10, 25, 38, 53 and 82	δ (ppm)	C	δ (ppm)	C
4.13	15	63.48	13					171.34	67	171.49	27
4.04	51	60.07	56	2.64	3, 14, 88, 36, 50, 61 and 76	34.29/34.32/ 34.36/34.54	2, 11, 26, 39, 54, 66 and 83	136.06	69	171.56	55
4.02	24 and 77	63.72 and 65.54	28 and 85					205.55	95	171.57	84
4.01	37	66	41	2.46	12, 23, 34, 48, 59 and 74	27.36/ 27.49	9, 23, 37, 52, 64 and 81	171.58	12		
4	38	75.75	42					171.4	40		

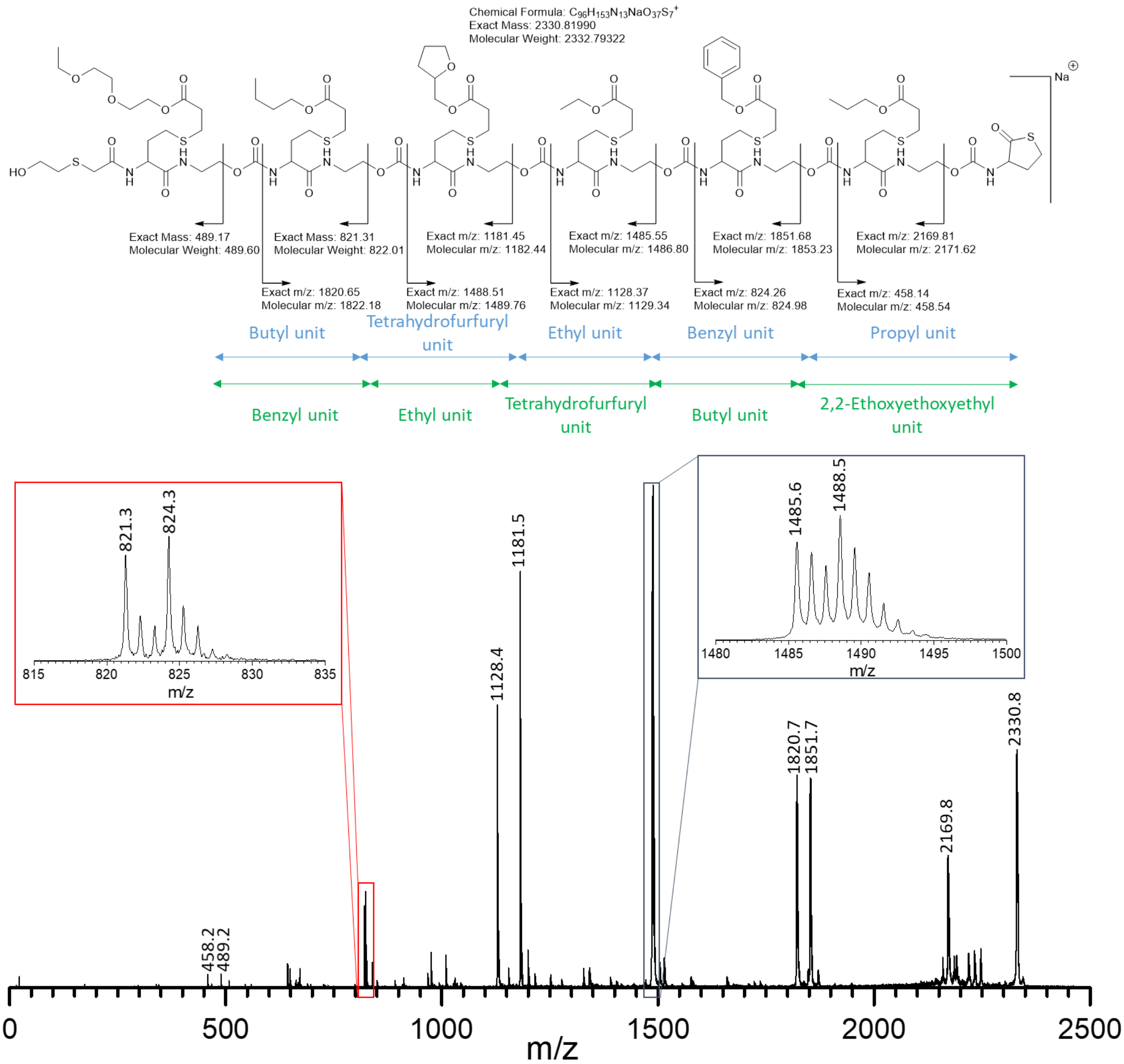

Supplementary Figure 41 | MALDI-MS/MS spectrum with peak assignment of **A2WRITE**.

*Characterization of **A3OR** using mass spectrometry (Supplementary Figure 42), NMR spectroscopy (Supplementary Figure 43) and MALDI-MS/MS analysis (Supplementary Figure 44).*

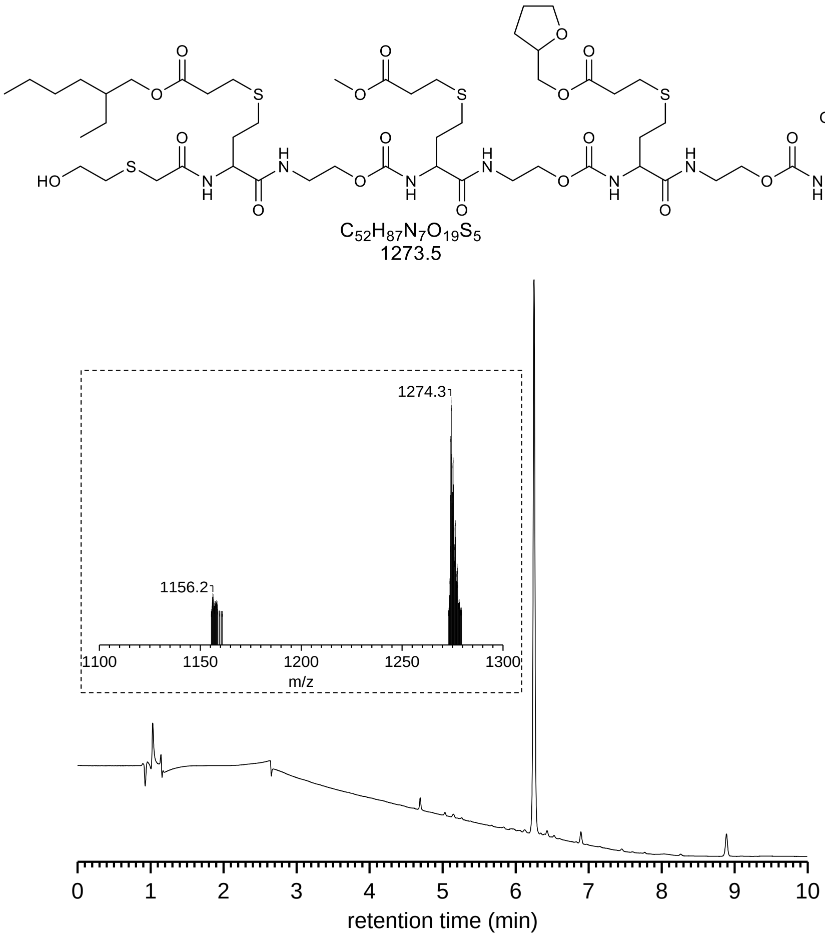

Supplementary Figure 42 | LC-ESI-MS analysis of **A3OR**. Insert: ESI-MS-spectrum of dominant species (positive mode).

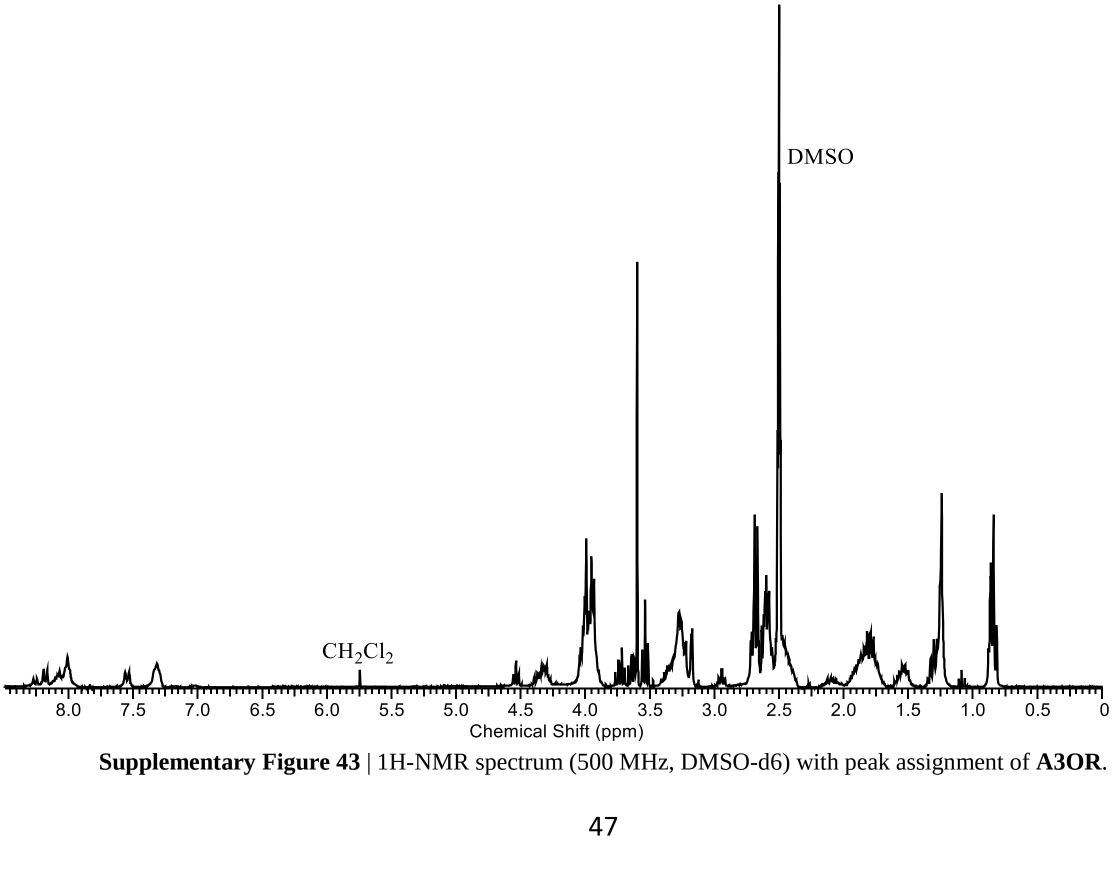

Supplementary Figure 43 | 1H-NMR spectrum (500 MHz, DMSO-d6) with peak assignment of **A3OR**.

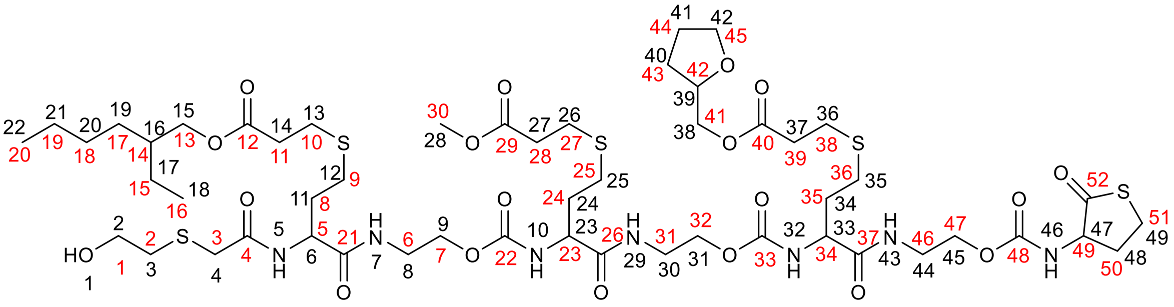

Supplementary Table 12 | determination of the ^{1}H- and ^{13}C-chemical shift values of **A3OR**

δ (ppm)	H	δ (ppm)	C
8.18	5	169.37	4
8.07	7	171.21	21
8.01	29 and 43	171.66	26 and 37
7.55	46	155.88	48
7.33	10 and 32	171.66	22 and 33
4.53	1	/	/
4.35	47	59.84	49
4.31	6	51.81	5
4.01	38 and 15	66.02 and 66.06	41 and 13
4	39	75.75	42
3.97	45, 31, 9 and 23, 33	62.51 and 53.86	47, 32, 7 and 23, 34
3.73	42	67.42	45
3.63	42	67.42	45
3.6	28	51.41	30
3.53	2	60.45	1
3.3	49	26.36	51
3.27	8, 30 and 44	38.15	6, 31 and 46
3.17	4	34.5	3
2.69	13, 26 and 36	26.01 and 26.21	10, 27 and 38
2.64	3, 14 and 37	34.54, 34.24 and 34.29	2, 11 and 39
2.6	27	34.11	28
2.46	12, 25 and 35	27.44 and 27.49	9, 25 and 36
2.43	48	29.8	50
2.09	48	29.8	50
1.81	11, 24 and 34, 40 and 41	32.09 and 32.20, 27.46 and 25.17	8, 24 and 35, 43 and 44
1.55	16 and 40	38.14 and 27.46	14 and 43
1.25	17, 19, 20 and 21	22.42, 23.22, 28.33 and 29.77	15, 17, 18 and 19
0.85	18 and 22	10.79 and 13.90	16 and 20
		171.50	12
		205.55	52
		171.93	29
		171.41	40

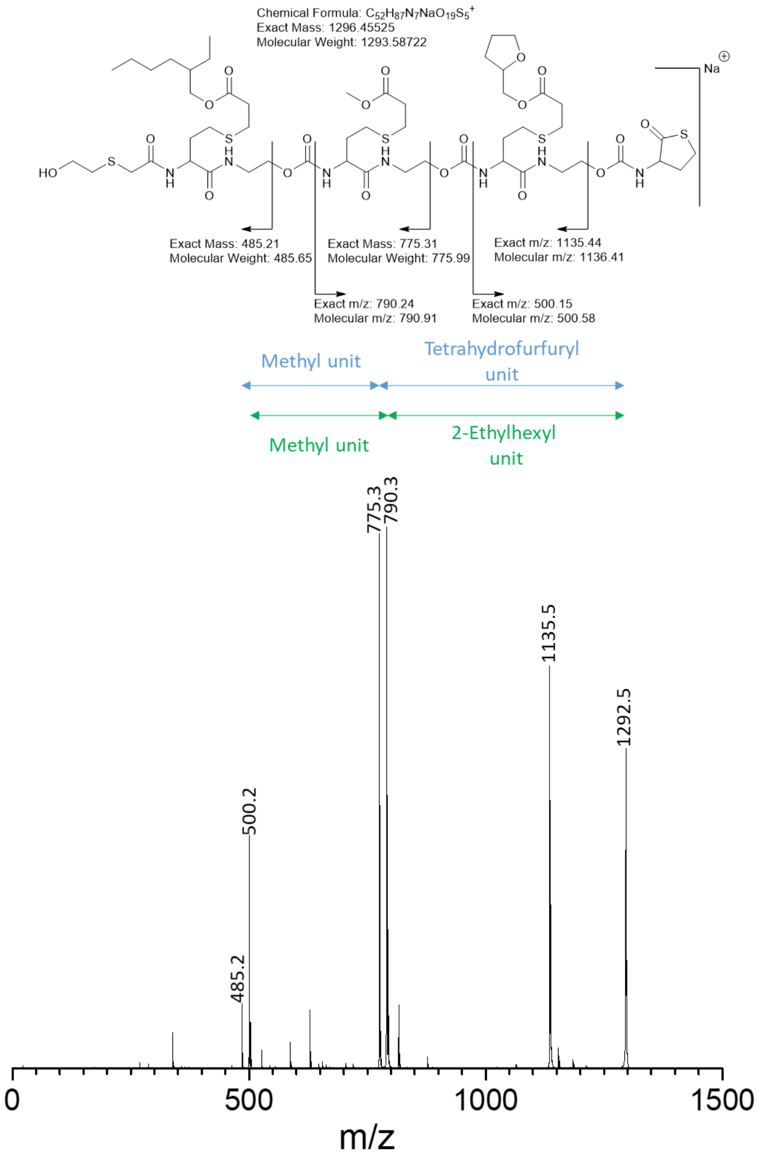

Supplementary Figure 44 | MALDI-MS/MS spectrum with peak assignment of **A3OR**.

*Characterization of **A4NOT** using mass spectrometry (Supplementary Figure 45), NMR spectroscopy (Supplementary Figure 46) and MALDI-MS/MS analysis (Supplementary Figure 47).*

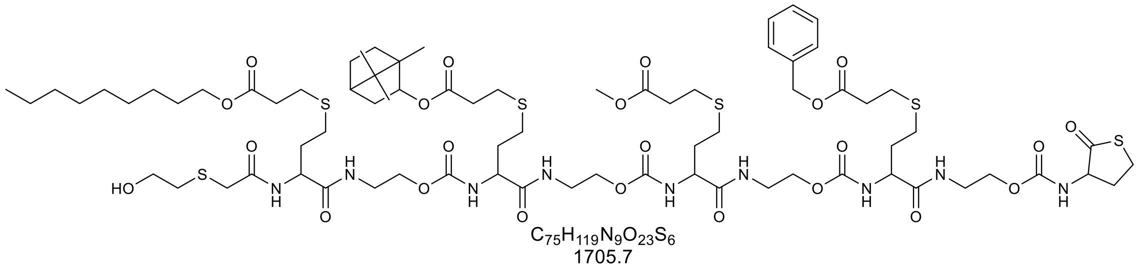

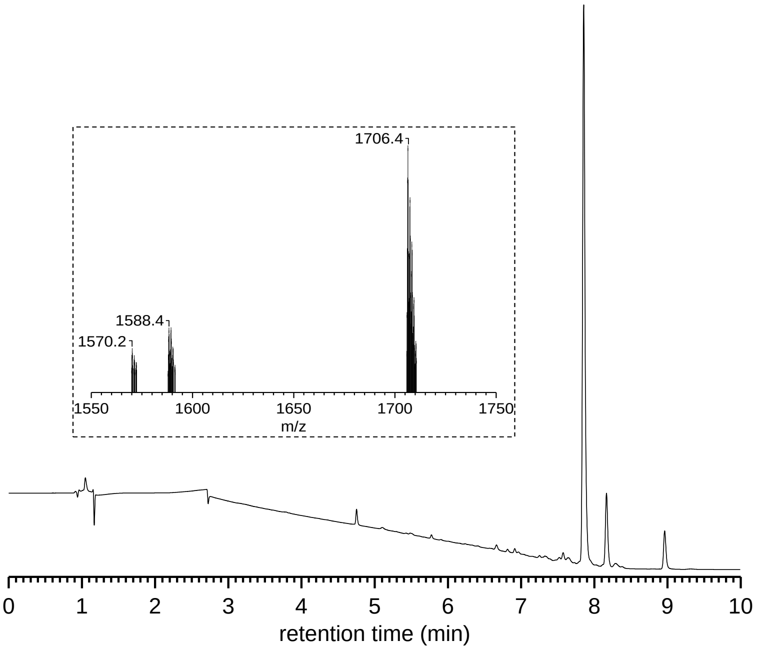

Supplementary Figure 45 | LC-ESI-MS analysis of **A4NOT**. Insert: ESI-MS-spectrum of dominant species (positive mode).

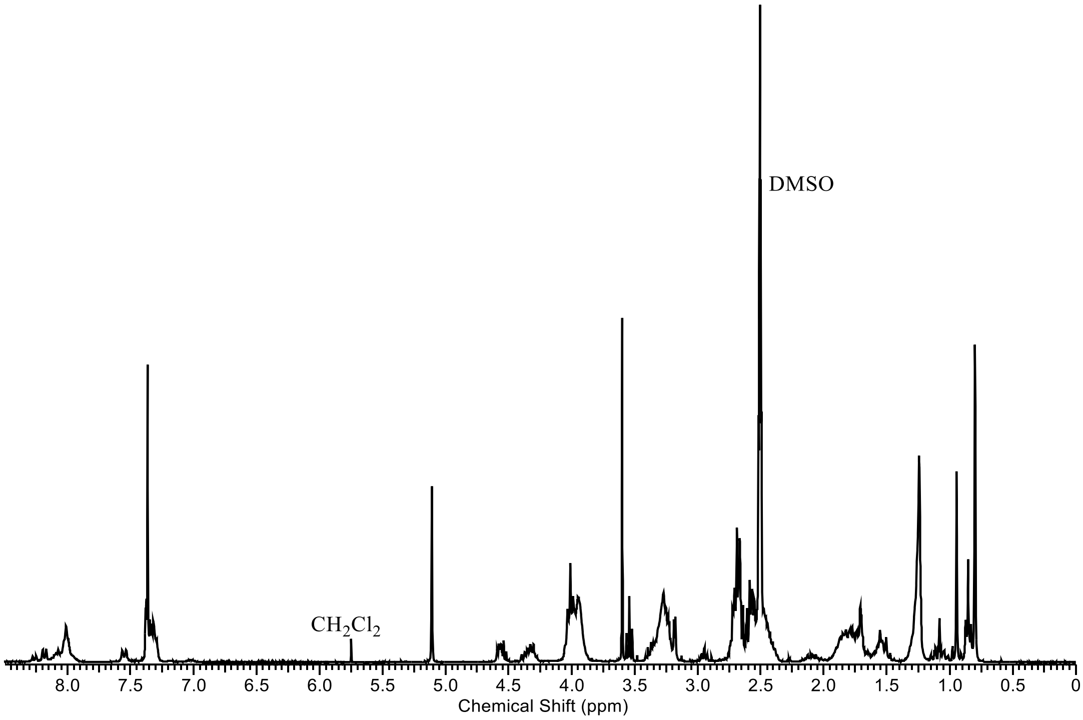

Supplementary Figure 46 | 1H-NMR spectrum (500 MHz, DMSO-d6) with peak assignment of **A4NOT**.

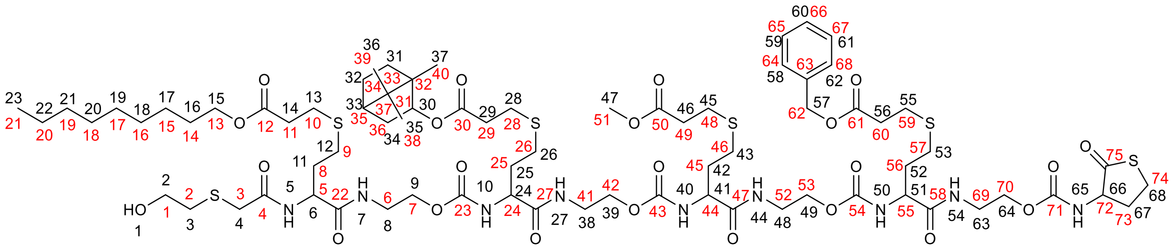

Supplementary Table 13 | determination of the ^{1}H- and ^{13}C-chemical shift values of **A4NOT**

δ (ppm)	H	δ (ppm)	C
8.18	5	169.37	4
8.07/8.01	7/27, 44 and 54	171.21/171.69	22/27, 47 and 58
7.55	65	155.88	71
7.36	58-62	127.9, 127.99 and 128.42	64-68
7.33	10, 40 and 50	171.66	23, 43 and 54
5.11	57	65.59	62
4.58	30	80.3	31
4.53	1	/	/
4.35/4.31	66 and 6	59.84/51.81	72 and 5
4	15	64.02	13
3.97	64, 49, 39, 9 and 24, 41, 51	62.51 and 53.86	70, 53, 42, 7 and 24, 44, 55
3.6	47	51.41	51
3.53	2	60.45	1
3.3	68	26.36	74
3.27	8, 38, 48 and 63	38.15	6, 41, 52 and 69
3.17	4	34.5	3
2.72	55	26.11	59
2.69	13, 28 and 45	26.01 and 26.22	10, 28 and 48
2.64	3, 14, 29 and 56	34.54, 34.61, 34.33 and 34.24	2, 11, 29 and 60
2.6	46	34.11	49
2.46	12, 26, 43 and 53	27.33 and 27.48	9, 26, 46 and 57
2.43/2.09	67	29.8	73
1.81	11, 25, 42 and 52	32.12	8, 25, 45 and 56
1.7	33 and 34	44.37 and 38.32	35 and 36
1.63	31	26.6	33
1.55/1.5	16 and 32	28.11/33.24	14 and 34
1.24	17-22	22.10, 25.34, 28.64, 28.89 and 31.27	15-20
1.08	31 and 32	26.6 and 33.24	33 and 34
0.95	37	19.73	40
0.85	23	13.95	21
0.8	35 and 36	11.39 and 19.93	38 and 39
171.47	12	171.34	61
170.73	30	136.06	63
46.53	32	205.55	75
48.21	37	171.93	50

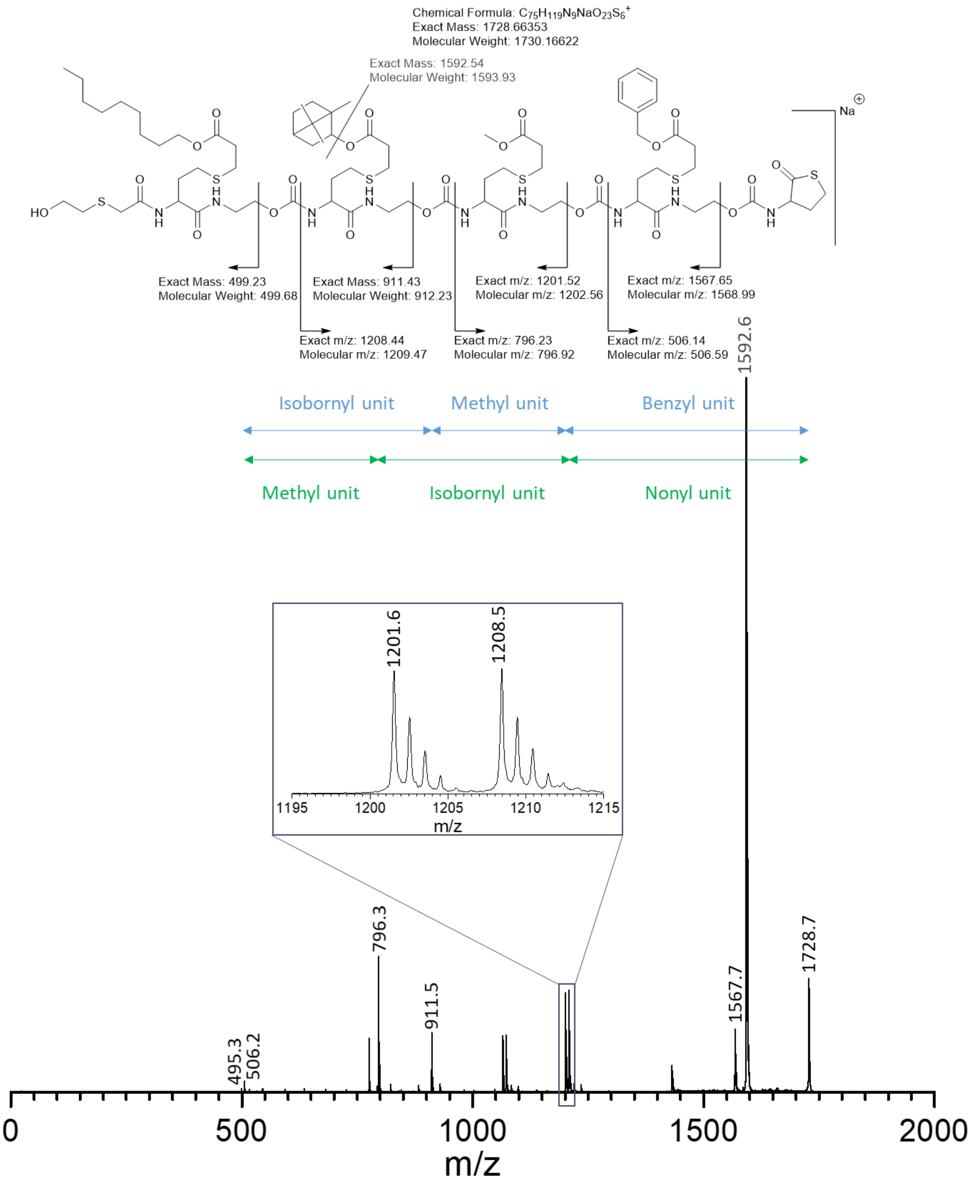

Supplementary Figure 47 | MALDI-MS/MS spectrum with peak assignment of **A4NOT**.

*Characterization of **A5TO** using mass spectrometry (Supplementary Figure 48), NMR spectroscopy (Supplementary Figure 49) and MALDI-MS/MS analysis (Supplementary Figure 50).*

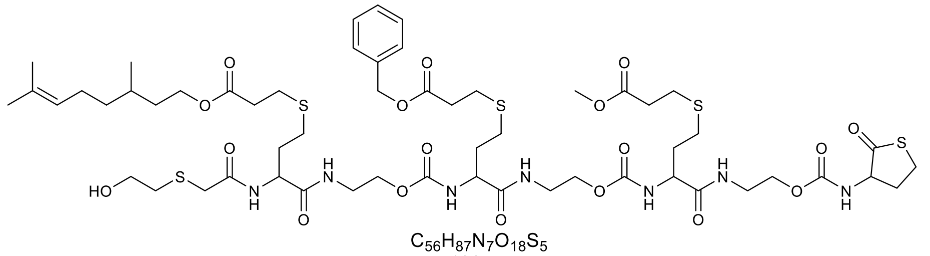

$C_{56}H_{87}N_{7}O_{18}S_{5}$
1305.5

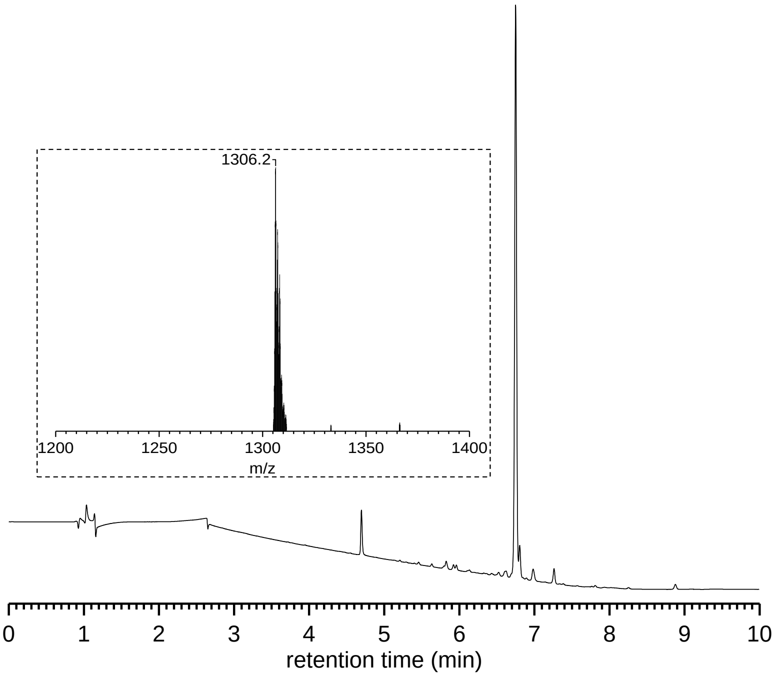

Supplementary Figure 48 | LC-ESI-MS analysis of **A5TO**. Insert: ESI-MS-spectrum of dominant species (positive mode).

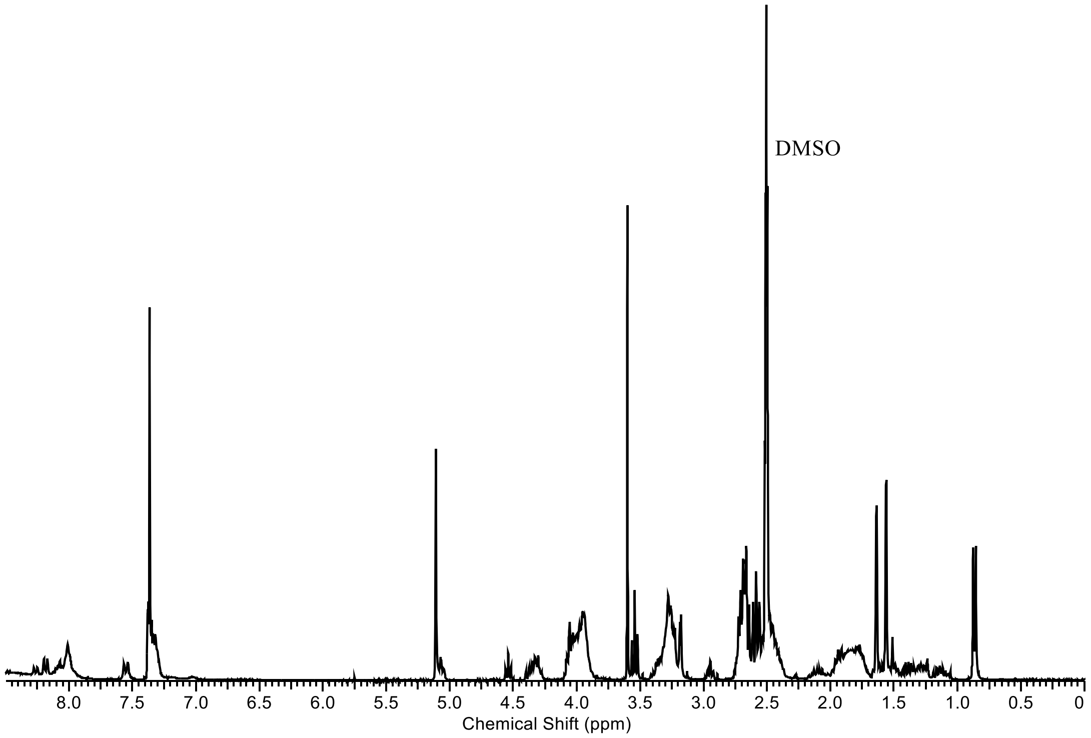

Supplementary Figure 49 | ^{1}H-NMR spectrum (500 MHz, DMSO-d6) with peak assignment of **A5TO**.

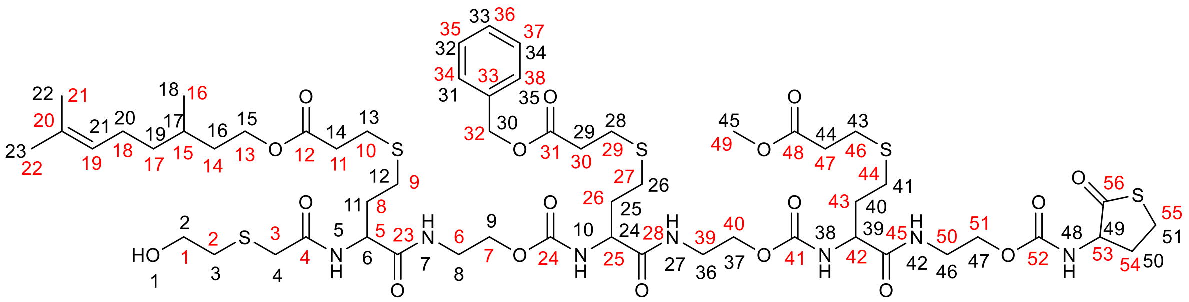

Supplementary Table 14 | determination of the ¹H- and ¹³C-chemical shift values of **A5TO**

δ (ppm)	H	δ (ppm)	C
8.18	5	169.37	4
8.07	7	171.21	23
8.01	27 and 42	171.69	28 and 45
7.55	48	155.88	52
7.36	31-35	127.9, 127.99 and 128.42	34-38
7.33	10 and 38	171.66	24 and 41
5.11	30	65.59	32
5.06	21	124.52	19
4.53	1	/	/
4.35	49	59.84	53
4.31	6	51.81	5
4.05	15	62.36	13
3.97	47, 37, 9 and 24, 39	62.51 and 53.86	51, 40, 7 and 25, 42
3.6	45	51.41	49
3.53	2	60.45	1
3.3	51	26.36	55
3.27	8, 36 and 46	38.15	6, 39 and 50
3.17	4	34.5	3
2.72	28	26.11	29
2.69	13 and 43	26.01	10 and 46
2.64	3, 14 and 29	34.54, 34.32 and 34.25	2, 11 and 30
2.6	44	34.11	47
2.46	12, 26 and 41	27.33 and 27.48	9, 27 and 44
2.43/2.09	50	29.8	54
1.93	20	24.86	18
1.81	11, 25 and 40	32.07 and 32.21	8, 26 and 43
1.63/1.55	22 and 23	17.51 and 25.51	21 and 22
1.59/1.38	16	34.93	14
1.51	17	28.84	15
1.28/1.13	19	36.46	17
0.86	18	19.11	16
171.93	48	171.34	31
171.45	12	136.06	33
130.60	20	205.55	56

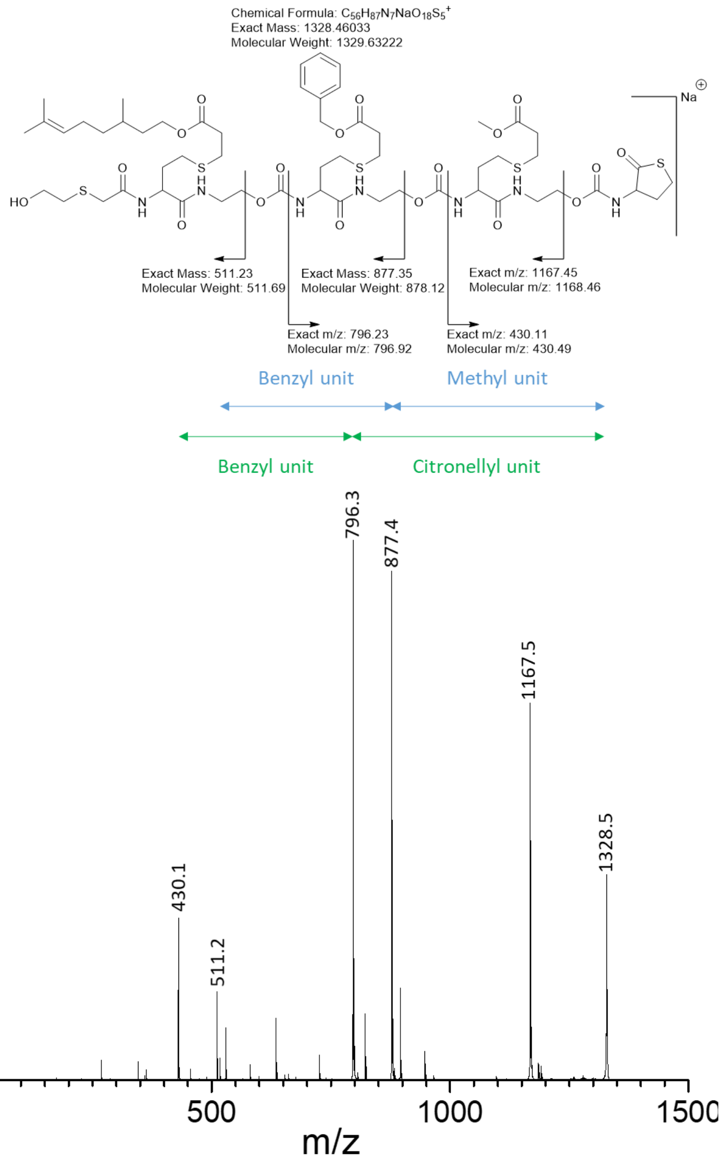

Supplementary Figure 50 | MALDI-MS/MS spectrum with peak assignment of **A5TO**.

*Characterization of **A6WRITE** using mass spectrometry (Supplementary Figure 51), NMR spectroscopy (Supplementary Figure 52) and MALDI-MS/MS analysis (Supplementary Figure 53).*

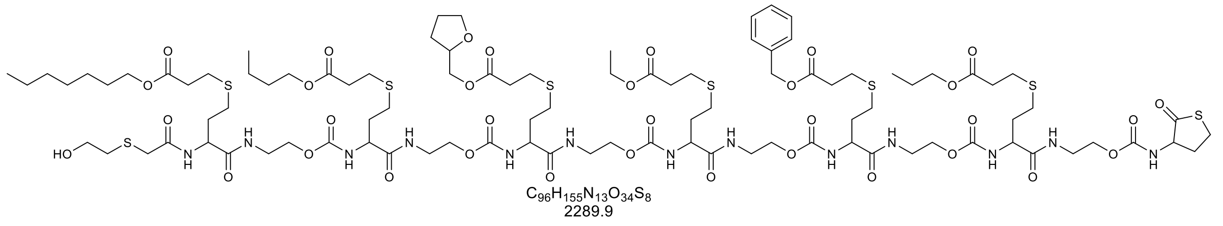

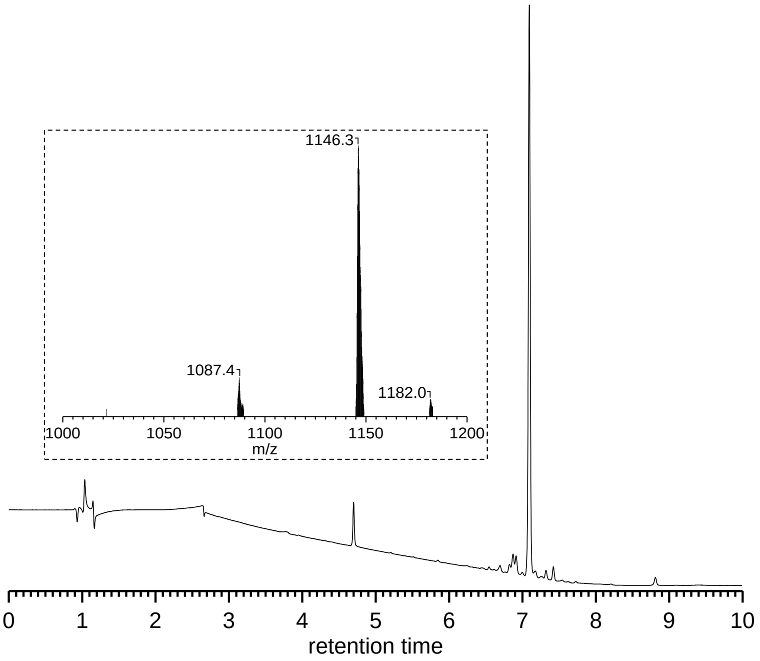

Supplementary Figure 51 | LC-ESI-MS analysis of **A6WRITE**. Insert: ESI-MS-spectrum of dominant species (positive mode).

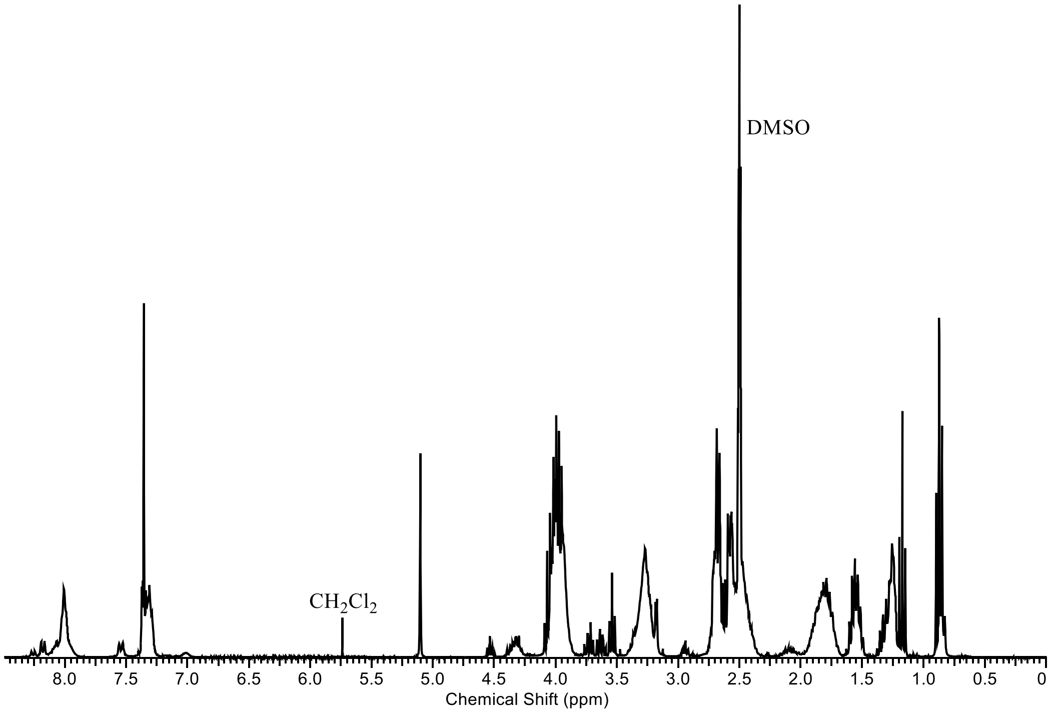

Supplementary Figure 52 | ^{1}H-NMR spectrum (500 MHz, DMSO-d6) with peak assignment of **A6WRITE**.

Supplementary Table 15 | determination of the ^{1}H- and ^{13}C-chemical shift values of **A6WRITE**

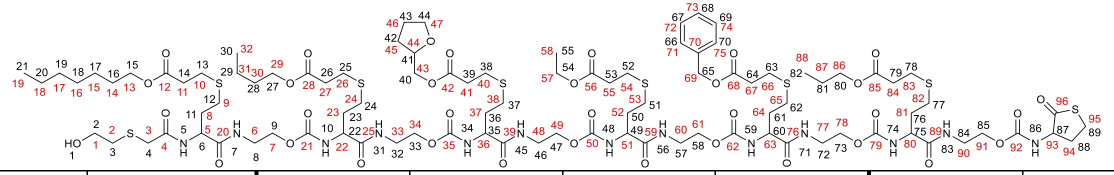

δ (ppm)	H	δ (ppm)	C
8.18	5	169.37	4
8.07 and 8.01	7, 31, 45, 56, 71 and 83	171.21 and 171.69	20, 25, 39, 59, 76 and 89
7.55	86	155.88	92
7.36	66-70	127.9, 127.99 and 128.41	71-75
7.33	10, 34, 48, 59 and 74	155.88	21, 35, 50, 62 and 79
5.11	65	65.61	69
4.53	1	/	/
4.35	87	59.88	93
4.31	6	51.81	5
4.04	54	60.07	57
4.03	15	64.03	13
4.02	27 and 80	63.72 and 65.54	29 and 86
4.01 and 4	40 and 41	66 and 75.75	43 and 44
3.97	85, 73, 58, 47, 33, 9 and 22, 35, 49, 60, 75	62.50 and 53.90	91, 78, 61, 49, 34, 7 and 22, 36, 51, 63, 80

δ (ppm)	H	δ (ppm)	C
3.73/3.63	44	67.42	47
3.53	2	60.45	1
3.3	89	26.36	95
3.27	8, 32, 46, 57, 72 and 84	38.15 /33.86	6, 33, 48, 60, 77 and 90
3.17	4	34.5	3
2.72	63	26.11	66
2.69	13, 25, 38, 52 and 78	26.05/26.08/ 26.18	10, 26, 40, 54 and 83
2.64	3, 14, 26, 39, 53, 64 and 79	34.54/34.26/ 34.30/34.33	2, 11, 27, 41, 55, 67 and 84
2.46	12, 24, 37, 51, 62 and 77	27.36/ 27.49	9, 24, 38, 53, 65 and 82
2.43/2.09	88	29.8	94
1.81	11, 23, 36, 50, 61 and 76; 42 and 43	32.05 and 32.18; 27.46 and 25.17	8, 23, 37, 52, 64 and 81; 45 and 46
1.55	42, 28, 16 and 81	27.46, 30.16, 28.13 and 21.65	45, 30, 14 and 87

δ (ppm)	H	δ (ppm)	C
1.33	29	18.59	31
1.26	17-20	22.04, 25.33, 28.32 and 31.18	15-18
1.18	55	14.11	58
0.88	30 and 82	13.53 and 10.26	32 and 88
0.85	21	13.92	19
**δ (ppm)**	**C**	**δ (ppm)**	**C**
171.34	68	171.49	28
136.06	70	171.52	56
205.55	96	171.54	85
171.49	12		
171.4	42		

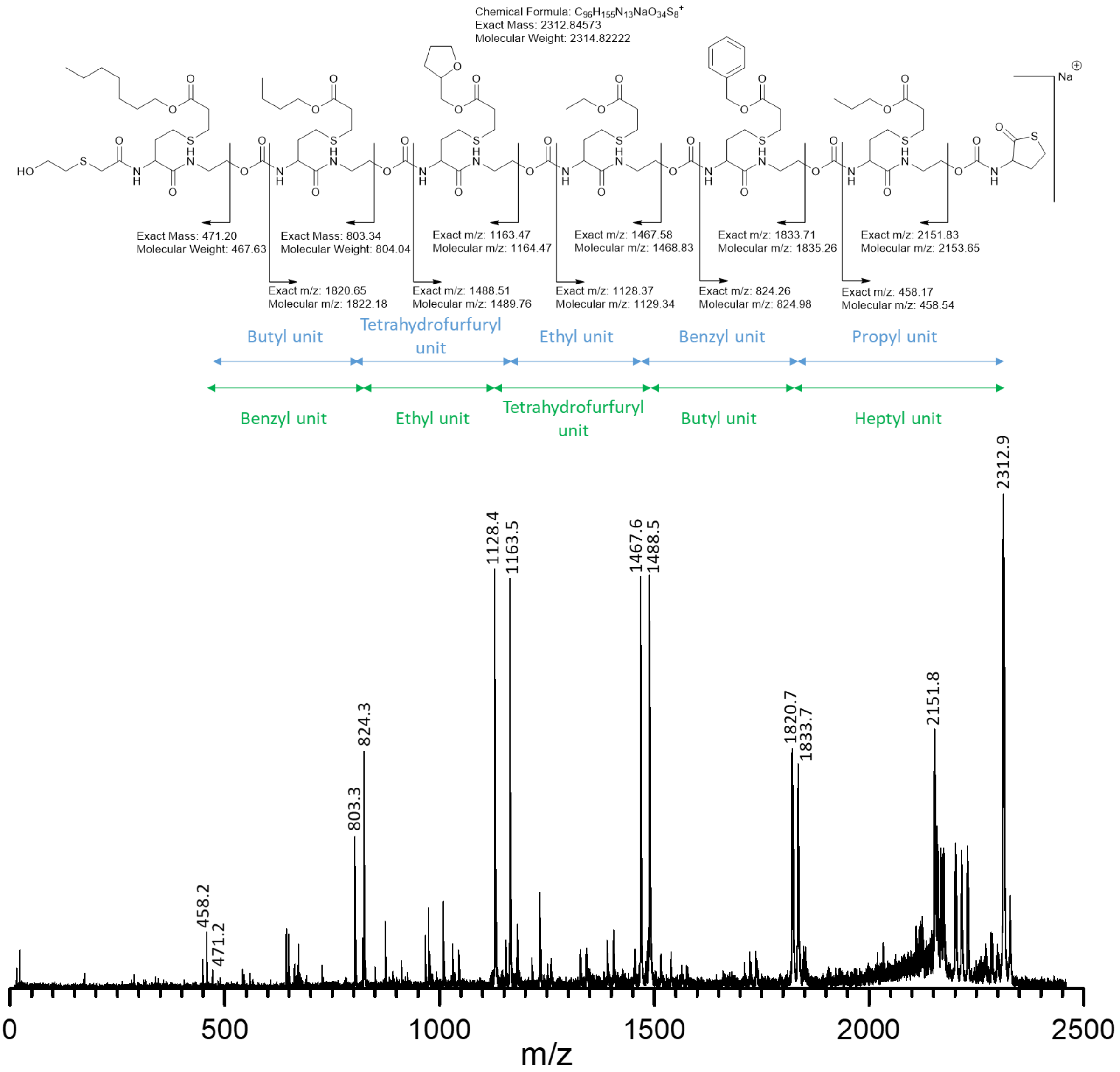

Supplementary Figure 53 | MALDI-MS/MS spectrum with peak assignment of **A6WRITE**.

*Characterization of **A7ON** using mass spectrometry (Supplementary Figure 54), NMR spectroscopy (Supplementary Figure 55) and MALDI-MS/MS analysis (Supplementary Figure 56).*

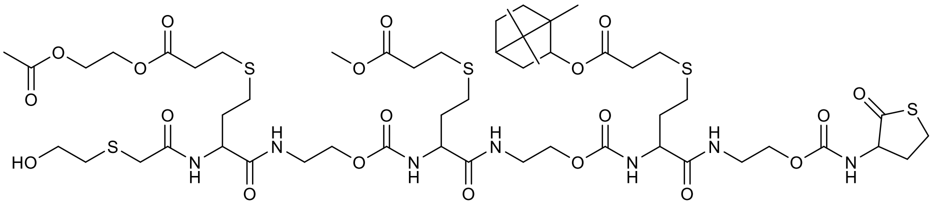

$C_{53}H_{85}N_7O_{20}S_5$
1299.45

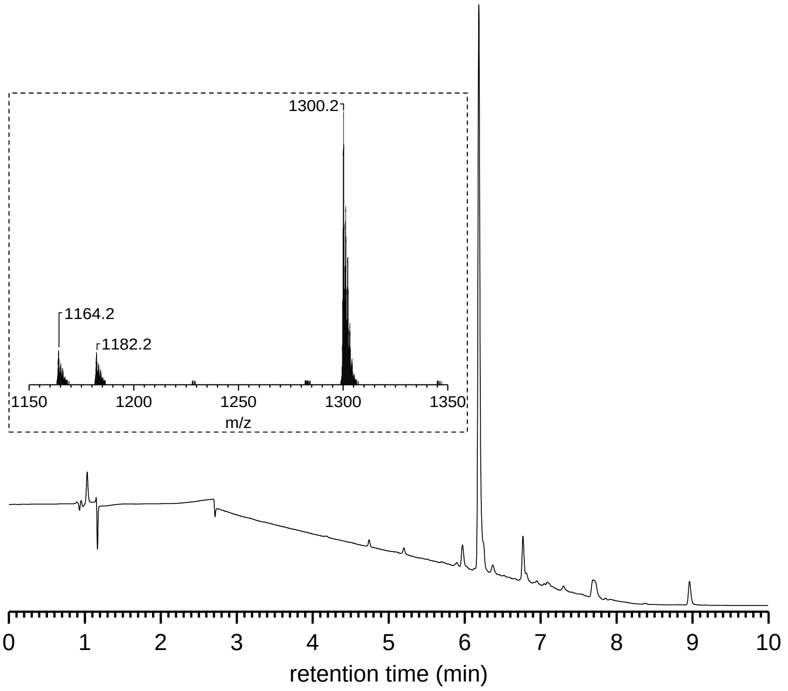

Supplementary Figure 54 | LC-ESI-MS analysis of **A7ON**. Insert: ESI-MS-spectrum of dominant species (positive mode).

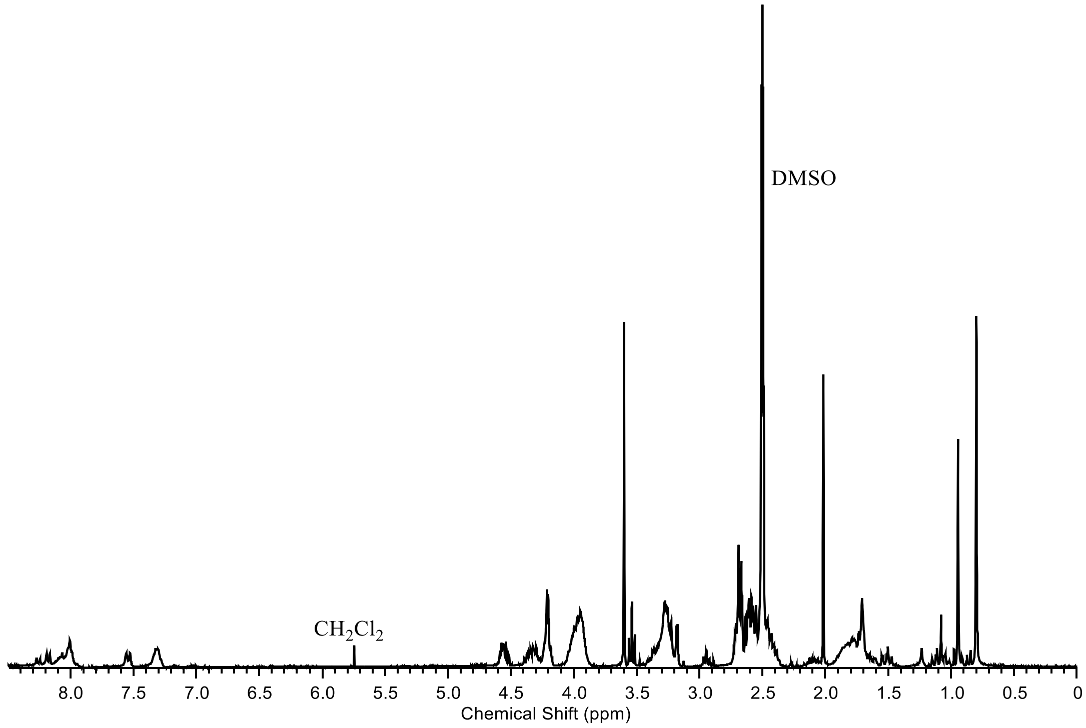

Supplementary Figure 55 | ^{1}H-NMR spectrum (500 MHz, DMSO-d6) with peak assignment of **A7ON**.

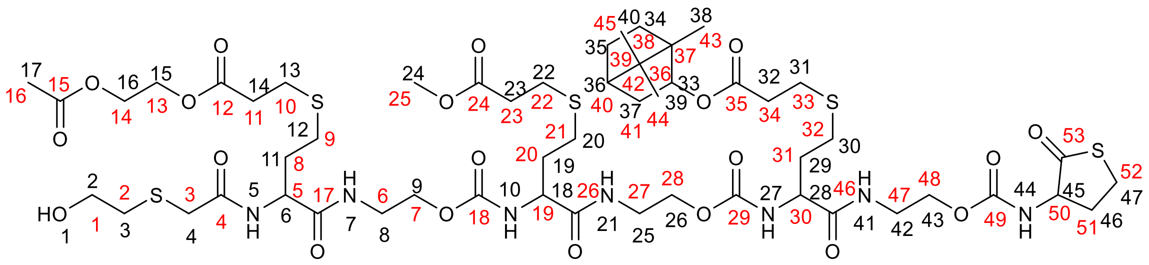

Supplementary Table 16 | determination of the ^{1}H- and ^{13}C-chemical shift values of **A7ON**

δ (ppm)	H	δ (ppm)	C
8.18	5	169.37	4
8.07	7	171.21	17
8.01	21 and 41	171.69	26 and 46
7.55	44	155.88	49
7.33	10 and 27	171.66	18 and 29
4.58	33	80.3	36
4.53	1	/	/
4.35	45	59.84	50
4.31	6	51.81	5
4.21	15 and 16	62.11 and 61.87	13 and 14
3.97	43, 26, 9 and 18, 28	62.51 and 53.86	48, 28, 7 and 19, 30
3.6	24	51.41	25
3.53	2	60.45	1
3.3	47	26.36	52
3.27	8, 25 and 42	38.15	6, 27 and 47
3.17	4	34.5	3
2.69	13, 22 and 31	26.01 and 26.22	10, 22 and 33
2.64	3, 14 and 32	34.54, 34.17 and 34.61	2, 11 and 34
2.6	23	34.11	23
2.46	12, 20 and 30	27.33 and 27.48	9, 21 and 32
2.43/2.09	46	29.8	51
2.01	17	20.61	16
1.81	11, 19 and 29	32.12	8, 20 and 31
1.7	36 and 37	44.37 and 38.32	40 and 41
1.63	34	26.6	38
1.5	35	33.24	39
1.08	34 and 35	26.6 and 33.24	38 and 39
0.95	38	19.73	43
0.8	39 and 40	11.39 and 19.93	44 and 45
		171.39	12
		170.28	15
		205.55	53
		171.93	24
		170.73	35
		46.53	37
		48.21	42

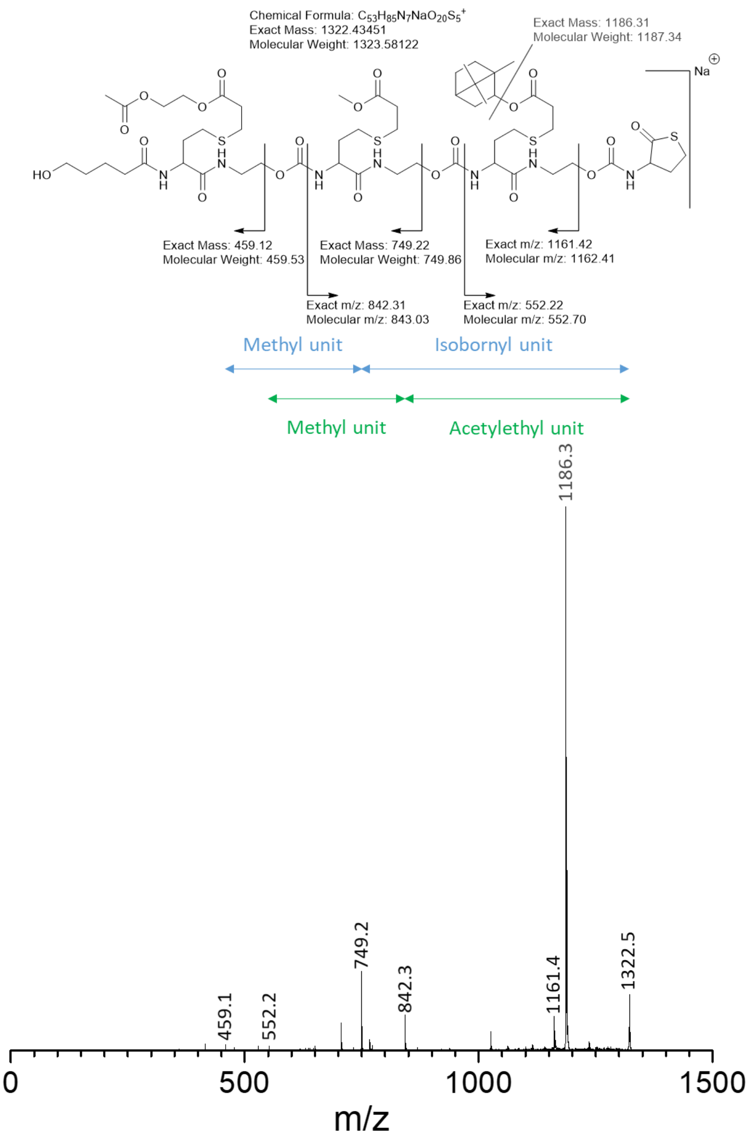

Supplementary Figure 56 | MALDI-MS/MS spectrum with peak assignment of **A7ON**.

*Characterization of **A8OLIGOS?** using mass spectrometry (Supplementary Figure 57), NMR spectroscopy (Supplementary Figure 58) and MALDI-MS/MS analysis (Supplementary Figure 59).*

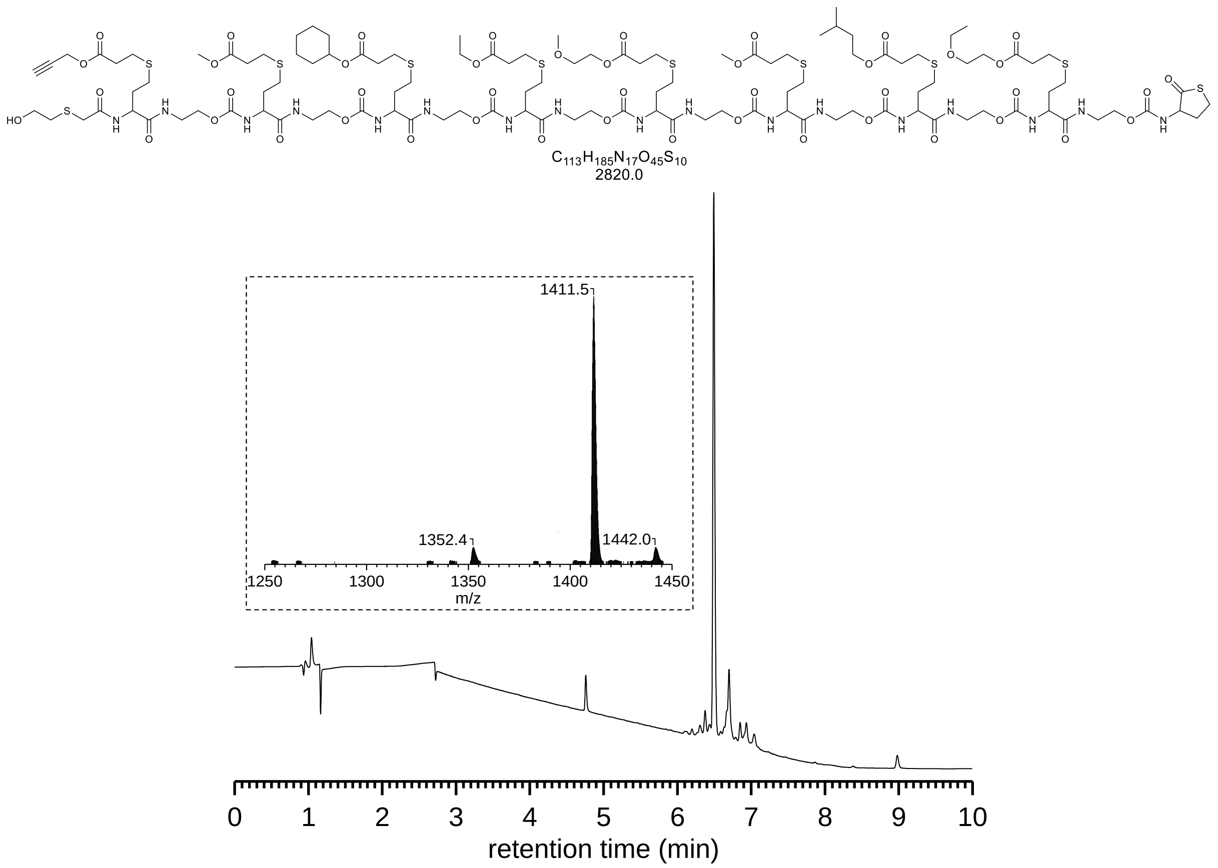

Supplementary Figure 57 | LC-ESI-MS analysis of **A8OLIGOS?**. Insert: ESI-MS-spectrum of dominant species (positive mode).

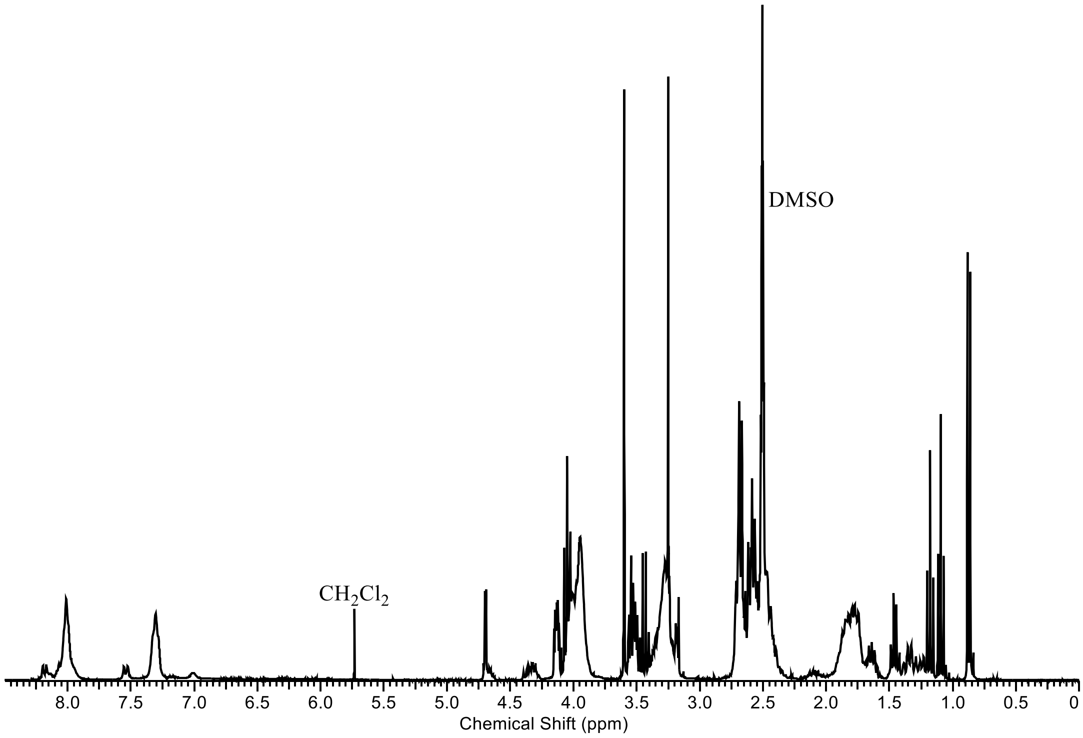

Supplementary Figure 58 | ^{1}H-NMR spectrum (500 MHz, DMSO-d6) with peak assignment of **A8OLIGOS?**.

Supplementary Table 17 | determination of the ^{1}H- and ^{13}C-chemical shift values of **A8OLIGOS?**

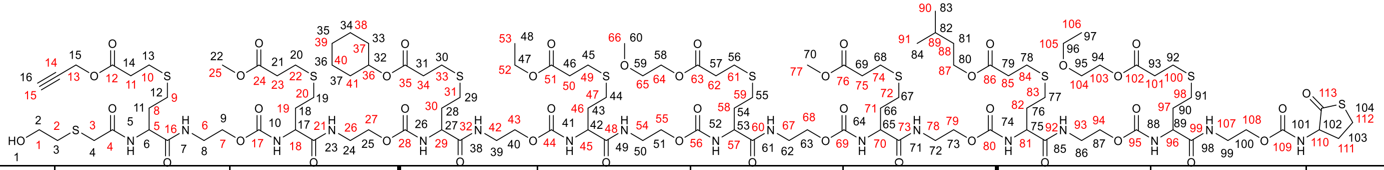

δ (ppm)	H	δ (ppm)	C
8.18	5	169.37	4
8.01/8.07	7, 23, 38, 49, 61, 71, 85 and 98	171.21 and 171.76	16, 21, 32, 48, 60, 73, 92 and 99
7.55	101	156.01	109
7.33	10, 26, 41, 52, 64, 74 and 88	156.01	17, 28, 44, 56, 69, 80 and 95
4.69/4.66	15 and 32	51.89 and 72.14	13 and 36
4.53	1	/	/
4.35	102	59.95	110
4.31	6	51.93	5
4.13	58 and 94	63.56 and 65.30	64 and 103
4.04	47 and 80	60.07 and 62.62	52 and 87
3.97	100, 87, 73, 63, 51, 40, 25, 9 and 17, 27, 42, 53, 65, 75, 89	62.57 and 53.98	108, 94, 79, 68, 55, 43, 27, 7 and 18, 29, 45, 57, 70, 81, 96
3.60	22 and 70	51.47	25 and 77
3.53	2	60.45	1

δ (ppm)	H	δ (ppm)	C
3.52	59 and 95	67.71 and 69.79	65 and 104
3.49	16	77.72	15
3.43	96	65.66	105
3.3	104	26.30	112
3.27	8, 24, 39, 50, 62, 72, 87 and 99	38.22	6, 26, 42, 54, 67, 78, 93 and 107
3.25	60	58.10	66
3.17	4	34.5	3
2.69	13, 20, 30, 45, 56, 68, 78 and 92	26.01/26.09/ 26.12/26.15/ 26.19/26.47	10, 22, 33, 49, 61, 74, 84 and 100
2.64	3, 14, 31, 46, 57, 79 and 93	34.54/34.34/ 34.41/34.71	2, 11, 34, 50, 62, 85 and 101
2.6	21 and 69	34.11	23 and 75
2.46	12, 19, 29, 44, 55, 67, 77 and 91	27.60	9, 20, 31, 47, 59, 72, 83 and 98
2.43/2.09	103	29.8	111
1.81	11, 18, 28, 43, 54, 66, 76 and 90	32.17	8, 19, 30, 46, 58, 71, 82 and 97

δ (ppm)	H	δ (ppm)	C
1.76-1.7	33 and 37	31.17	37 and 41
1.63	82 and 34, 36	24.59 and 23.24	89 and 38, 40
1.45	81 and 35	36.94 and 24.95	88 and 39
1.33	33, 37 and 34, 36	31.17 and 23.24	37, 41 and 38, 40
1.22	35	24.95	39
1.17	48	14.11	53
1.09	97	15.07	106
0.87	83 and 84	22.33	90 and 91

δ (ppm)	C	δ (ppm)	C
171.58	86	171.59 and 171.61	63 and 102
78.41	14		
205.55	113	171.52	51
170.93	12	170.89	35
171.93	24 and 76		

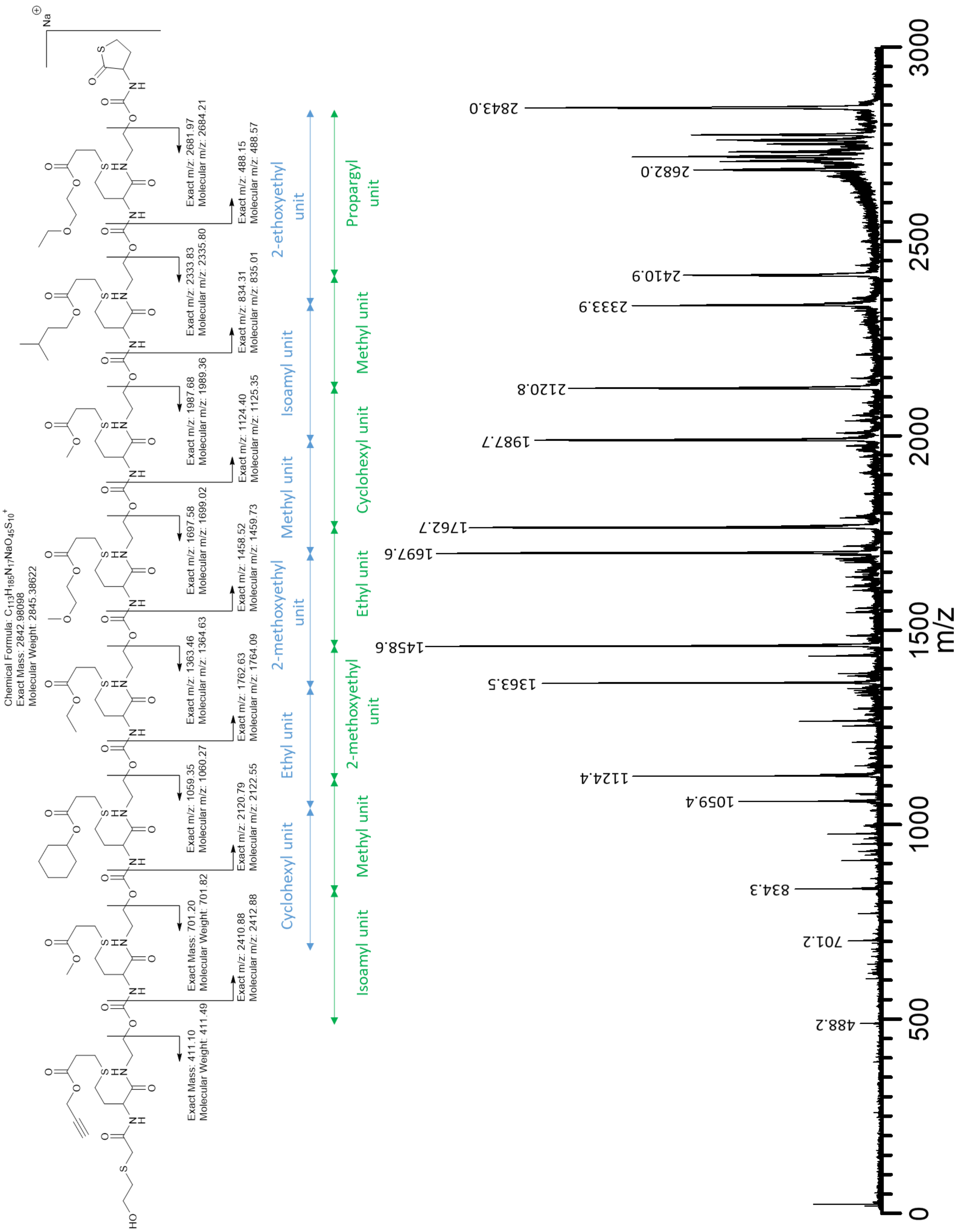

Supplementary Figure 59 | MALDI-MS/MS spectrum with peak assignment of **A8OLIGOS?**.

*Characterization of **Z1TO** using mass spectrometry (Supplementary Figure 60), NMR spectroscopy (Supplementary Figure 61) and MALDI-MS/MS analysis (Supplementary Figure 62).*

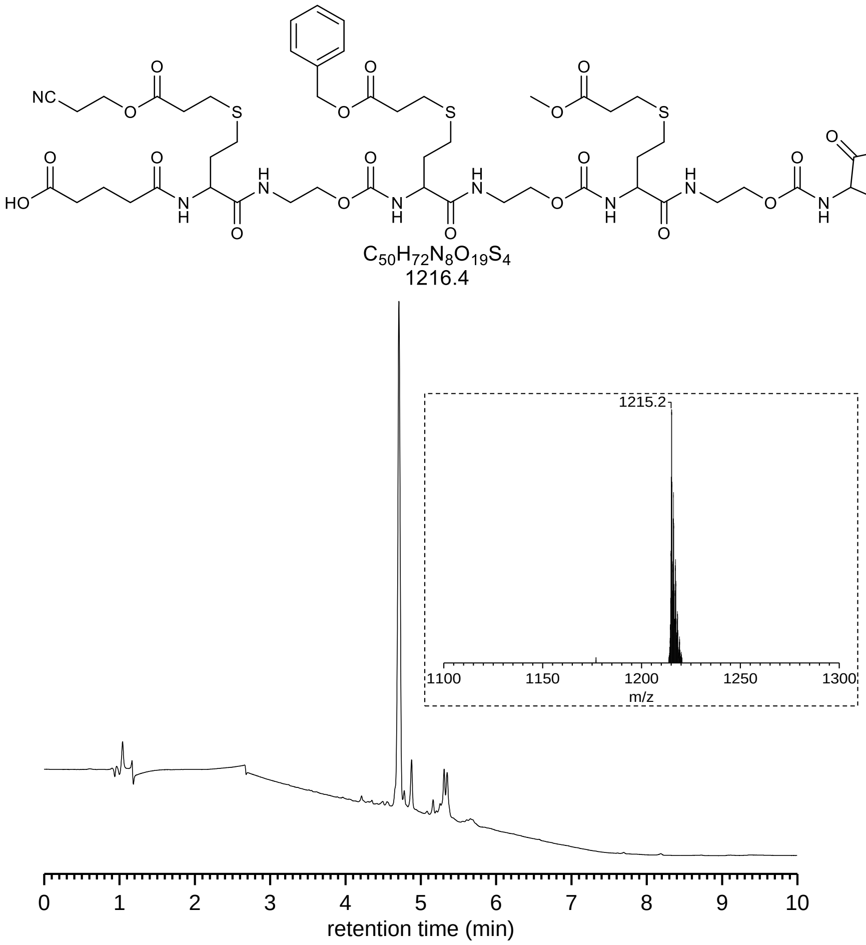

Supplementary Figure 60 | LC-ESI-MS analysis of **Z1TO**. Insert: ESI-MS-spectrum of dominant species (negative mode).

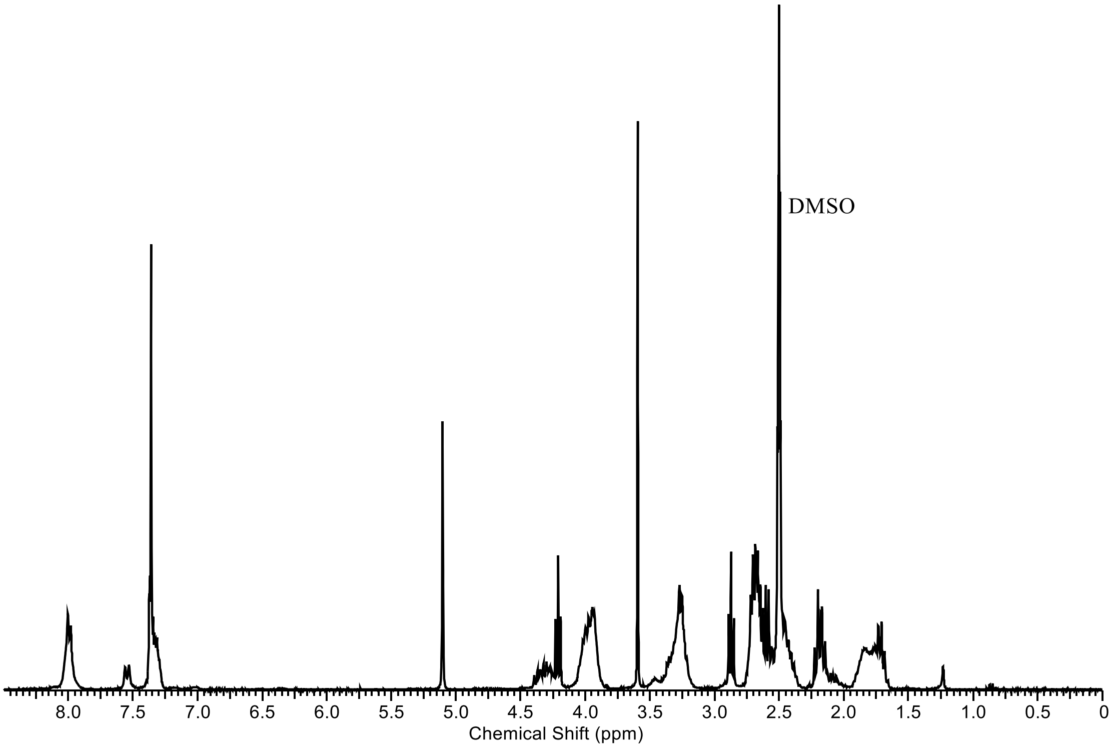

Supplementary Figure 61 | ^{1}H-NMR spectrum (500 MHz, DMSO-d_6) with peak assignment of **Z1TO**.

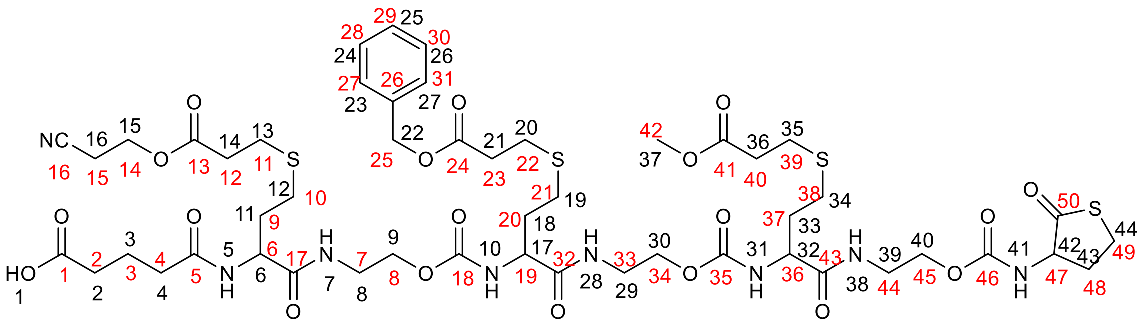

Supplementary Table 18 | determination of the ^{1}H- and ^{13}C-chemical shift values of **Z1TO**

δ (ppm)	H	δ (ppm)	C
13.25	1	174.24	1
8.01	5, 7, 28 and 38	171.69	5, 17, 32 and 43
7.55	41	155.88	46
7.36	23-27	127.9, 127.99 and 128.42	27-31
7.33	10 and 31	171.66	18 and 35
5.11	22	65.59	25
4.35	42	59.84	47
4.31	6	51.81	6
4.21	15	59.09	14
3.97	40, 30, 9 and 17, 32	62.51 and 53.86	45, 34, 8 and 19, 36
3.6	37	51.41	42
3.3	44	26.36	49
3.27	8, 29 and 39	38.15	7, 33 and 44
2.87	16	17.39	15
2.72	20	26.11	22
2.69	13 and 35	26.01	11 and 39
2.64	14 and 21	34.33 and 34.26	12 and 23
2.6	36	34.11	40
2.46	12, 19 and 34	27.33 and 27.48	10, 21 and 38
2.43	43	29.8	48
2.18	2 and 4	33.03 and 34.25	2 and 4
2.09	43	29.8	48
1.81	11, 18 and 33	32.12	9, 20 and 37
1.7	3	20.61	3
		171.34	24
		136.06	26
		205.55	50
		171.93	41
		171.15	13
		118.45	16

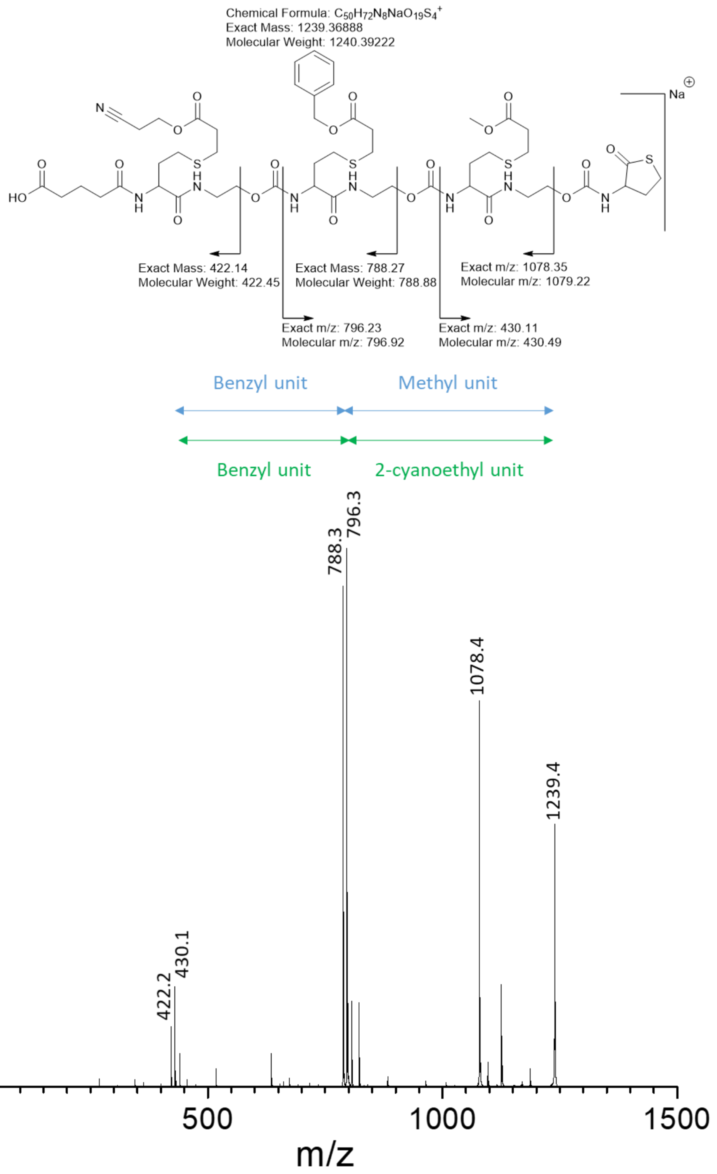

Supplementary Figure 62 | MALDI-MS/MS spectrum with peak assignment of **Z1TO**.

*Characterization of **Z2WRITE** using mass spectrometry (Supplementary Figure 63), NMR spectroscopy (Supplementary Figure 64) and MALDI-MS/MS analysis (Supplementary Figure 65).*

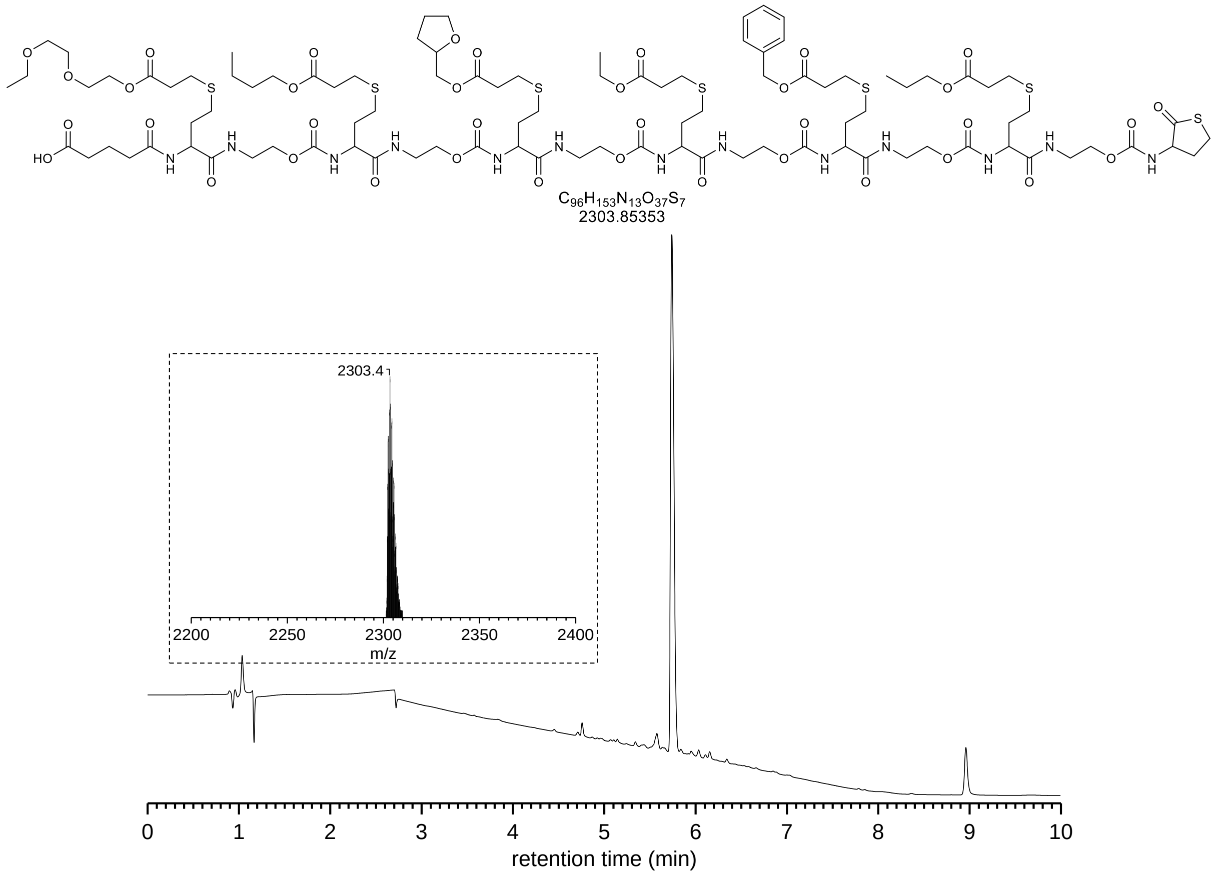

Supplementary Figure 63 | LC-ESI-MS analysis of **Z2WRITE**. Insert: ESI-MS-spectrum of dominant species (negative mode).

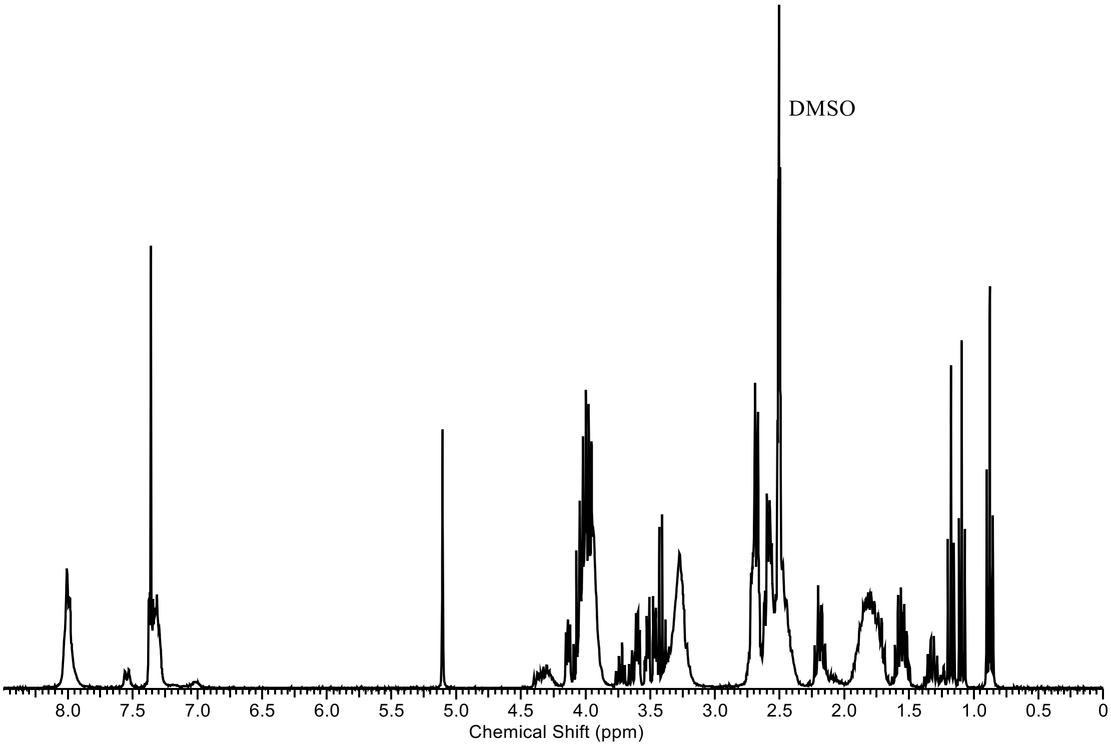

Supplementary Figure 64 | ^{1}H-NMR spectrum (500 MHz, DMSO-d_6) with peak assignment of **Z2WRITE**.

Supplementary Table 19 | determination of the ^{1}H- and ^{13}C-chemical shift values of **Z2WRITE**

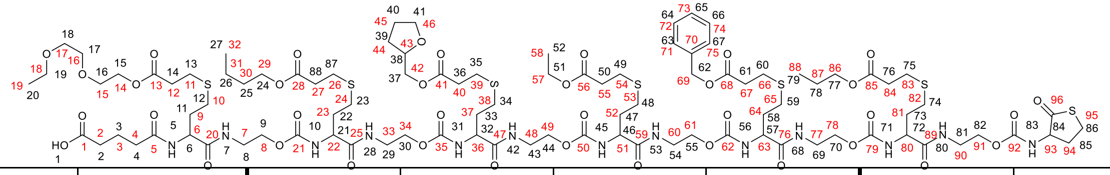

δ (ppm)	H	δ (ppm)	C
13.25	1	174.24	1
8.01	5, 7, 28, 42, 53, 68 and 80	171.69	5, 20, 25, 47, 59, 76 and 89
7.55	83	155.88	92
7.36	63-67	127.9, 127.99 and 128.41	71-75
7.33	10, 31, 45, 56 and 71	155.88	21, 35, 50, 62 and 79
5.11	62	65.61	69
4.35	84	59.88	93
4.31	6	51.81	6
4.13	15	63.48	14
4.04	51	60.07	57
4.02	24 and 77	63.72 and 65.54	29 and 86
4.01	37	66	42
4	38	75.75	43
3.97	82, 70, 55, 44, 30, 9 and 21, 32, 46, 57, 72	62.50 and 53.90	91, 78, 61, 49, 34, 8 and 22, 36, 51, 63, 80

δ (ppm)	H	δ (ppm)	C
3.73/3.63	41	67.42	46
3.59	16	68.30	15
3.49	17 and 18	69.21/69.88	16 and 17
3.41	19	65.66	18
3.3	86	26.36	95
3.27	8, 29, 43, 54, 69 and 81	38.15 /33.86	7, 33, 48, 60, 77 and 90
2.72	60	26.11	66
2.69	13, 87, 35, 49 and 75	26.09/26.15	11, 26, 39, 54 and 83
2.64	14, 88, 36, 50, 61 and 76	34.29/34.32/ 34.36	12, 27, 40, 55, 67 and 84
2.46	12, 23, 34, 48, 59 and 74	27.36/ 27.49	10, 24, 38, 53, 65 and 82
2.43	85	29.8	94
2.18	2 and 4	33.03/ 34.25	2 and 4
2.09	85	29.8	94

δ (ppm)	H	δ (ppm)	C
1.81	11, 22, 33, 47, 58 and 73; 39 and 40	32.05 and 32.18; 27.46 and 25.17	9, 23, 37, 52, 64 and 81; 44 and 45
1.7	3	20.61	3
1.55	39, 25 and 78	27.46, 30.16 and 21.65	44, 30 and 87
1.33	26	18.59	31
1.18	52	14.11	58
1.09	20	15.12	19
0.88	27 and 79	13.53 and 10.26	32 and 88
δ (ppm)	C	δ (ppm)	C
171.34	68	171.49	28
136.06	70	171.56	56
205.55	96	171.57	85
171.58	13		
171.4	41		

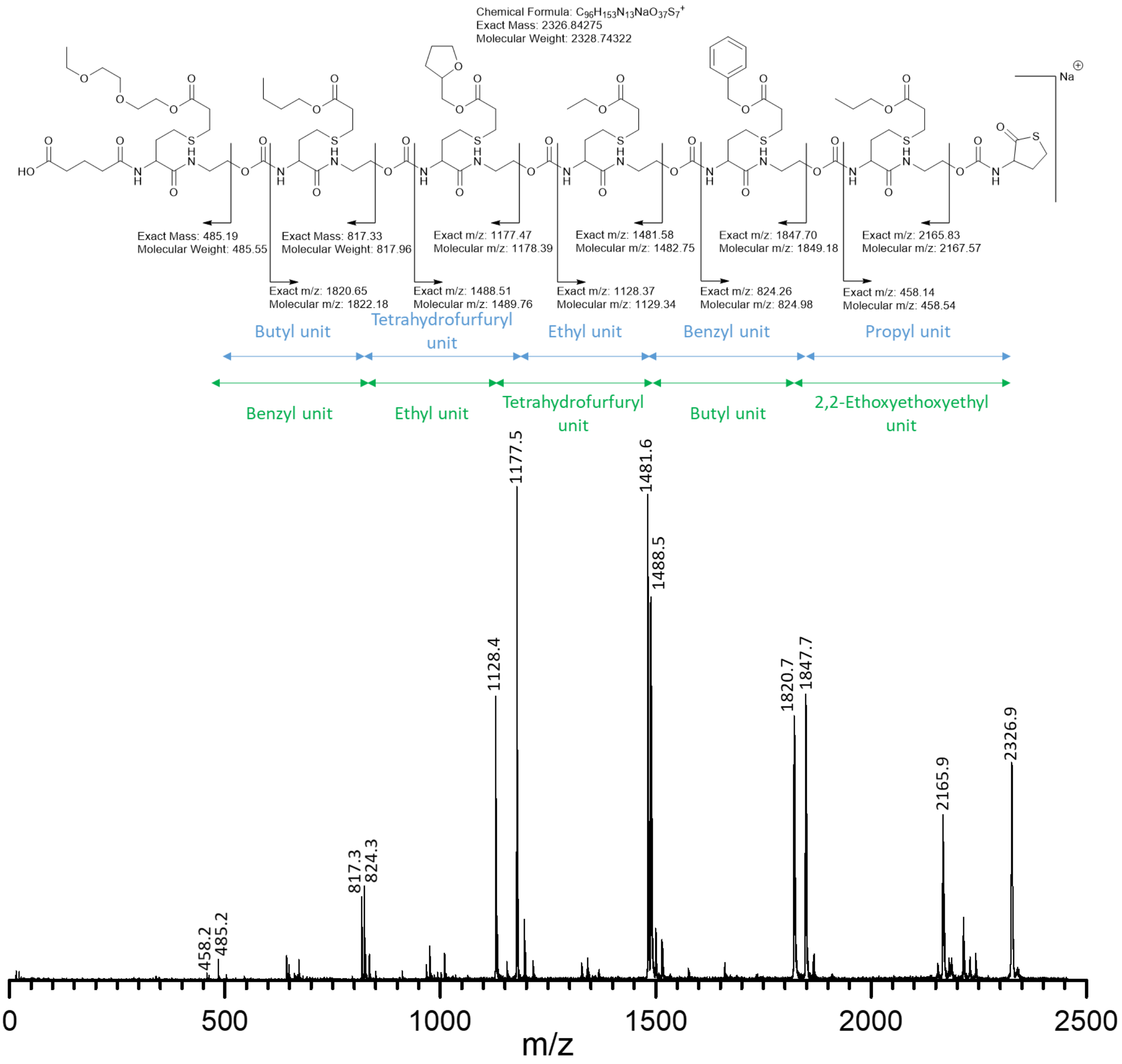

Supplementary Figure 65 | MALDI-MS/MS spectrum with peak assignment of **Z2WRITE**.

*Characterization of **Z3OR** using mass spectrometry (Supplementary Figure 66), NMR spectroscopy (Supplementary Figure 67) and MALDI-MS/MS analysis (Supplementary Figure 68).*

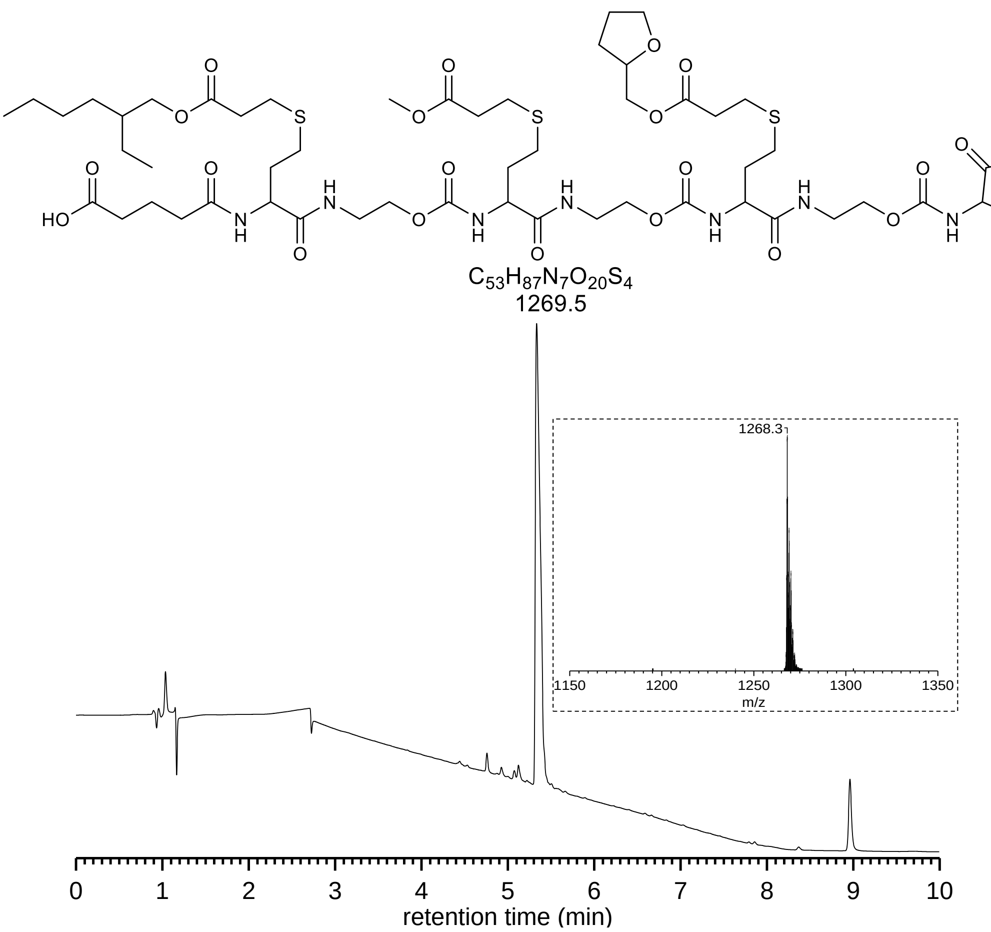

Supplementary Figure 66 | LC-ESI-MS analysis of **Z3OR**. Insert: ESI-MS-spectrum of dominant species (negative mode).

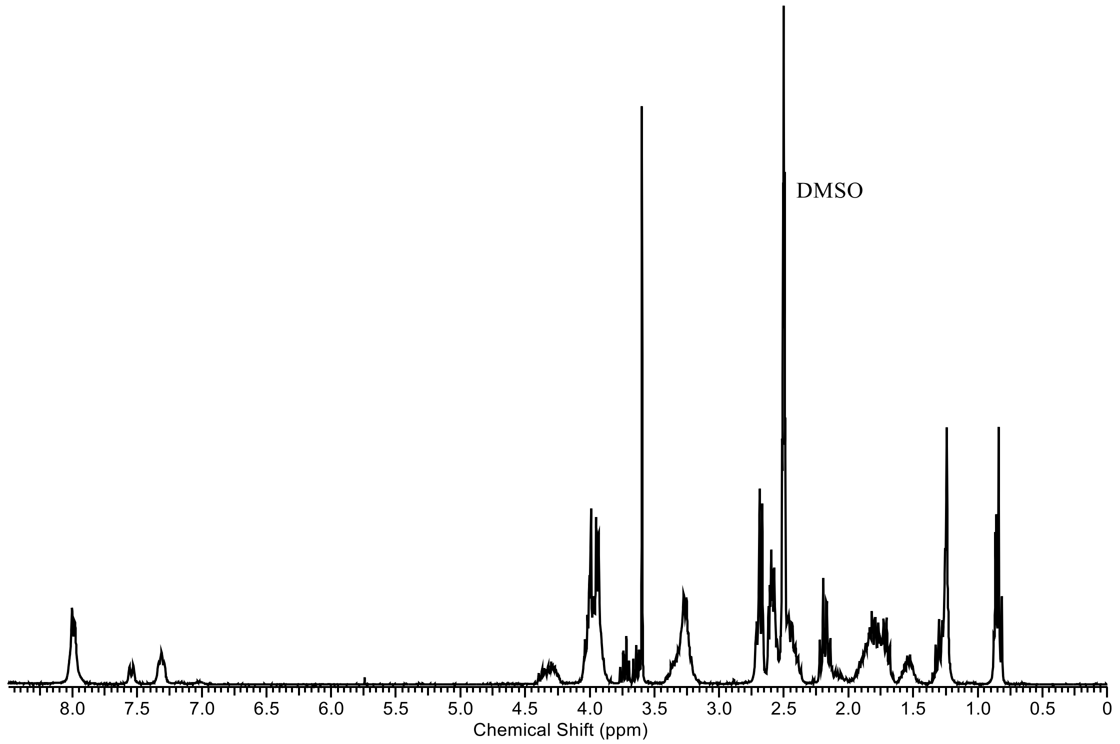

Supplementary Figure 67 | ^{1}H-NMR spectrum (500 MHz, DMSO-d_6) with peak assignment of **Z3OR**.

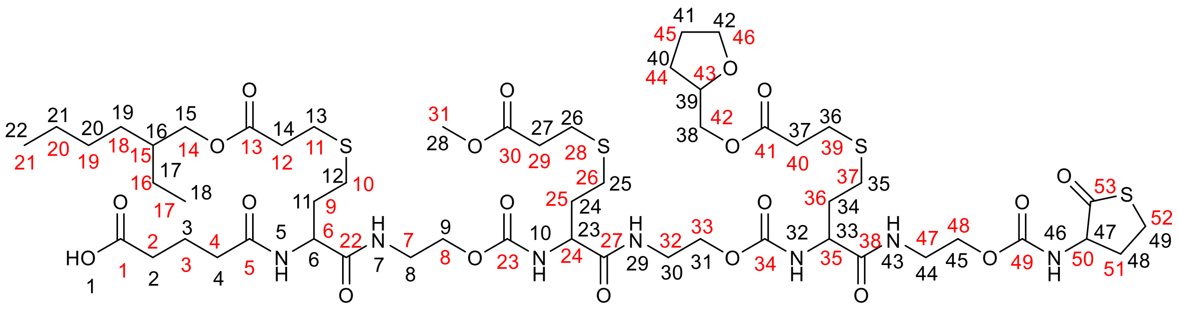

Supplementary Table 20 | determination of the [1]H- and ^{13}C-chemical shift values of **Z3OR**

δ (ppm)	H	δ (ppm)	C
13.25	1	174.24	1
8.01	5, 7, 29 and 43	171.66	5, 22, 27 and 38
7.55	46	155.88	49
7.33	10 and 32	171.66	23 and 34
4.35	47	59.84	50
4.31	6	51.81	6
4.01	38 and 15	66.02 and 66.06	42 and 14
4	39	75.75	43
3.97	45, 31, 9 and 23, 33	62.51 and 53.86	48, 33, 8 and 24, 35
3.73	42	67.42	46
3.63	42	67.42	46
3.6	28	51.41	31
3.3	49	26.36	52
3.27	8, 30 and 44	38.15	7, 32 and 47
2.69	13, 26 and 36	26.01 and 26.21	11, 28 and 39
2.64	14 and 37	34.24 and 34.29	12 and 40
2.6	27	34.11	29
2.46	12, 25 and 35	27.44 and 27.49	10, 26 and 37
2.43	48	29.8	51
2.18	2 and 4	33.03 and 34.25	2 and 4
2.09	48	29.8	51
1.81	11, 24 and 34, 40 and 41	32.09 and 32.20, 27.46 and 25.17	9,25 and 36, 44 and 45
1.7	3	20.61	3
1.55	16 and 40	38.14 and 27.46	15 and 44
1.25	17, 19, 20 and 21	22.42, 23.22, 28.33 and 29.77	16, 18, 19 and 20
0.85	18 and 22	10.79 and 13.90	17 and 21
		171.50	13
		205.55	53
		171.93	30
		171.41	41

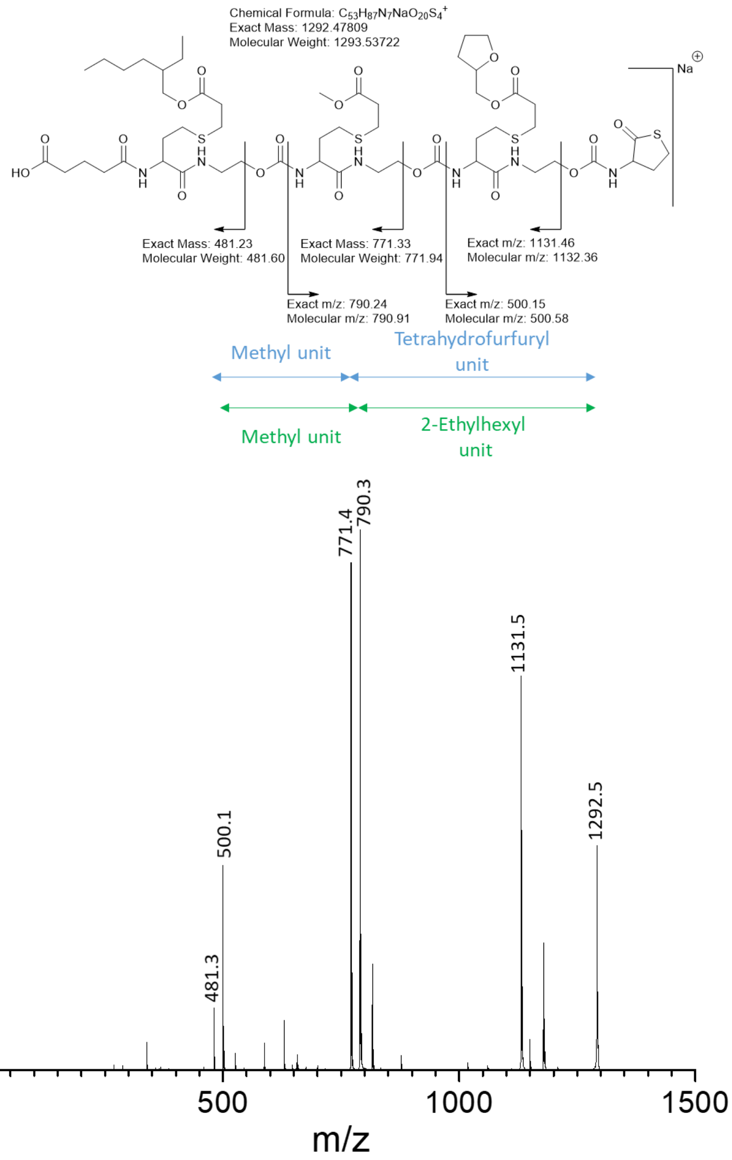

Supplementary Figure 68 | MALDI-MS/MS spectrum with peak assignment of **Z3OR**.

*Characterization of **Z4NOT** using mass spectrometry (Supplementary Figure 69), NMR spectroscopy (Supplementary Figure 70) and MALDI-MS/MS analysis (Supplementary Figure 71).*

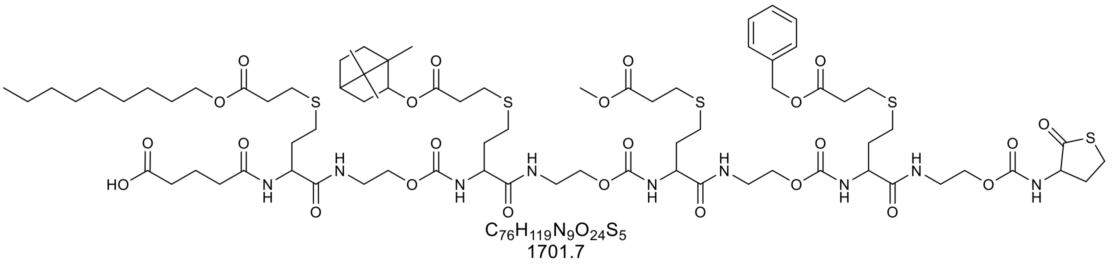

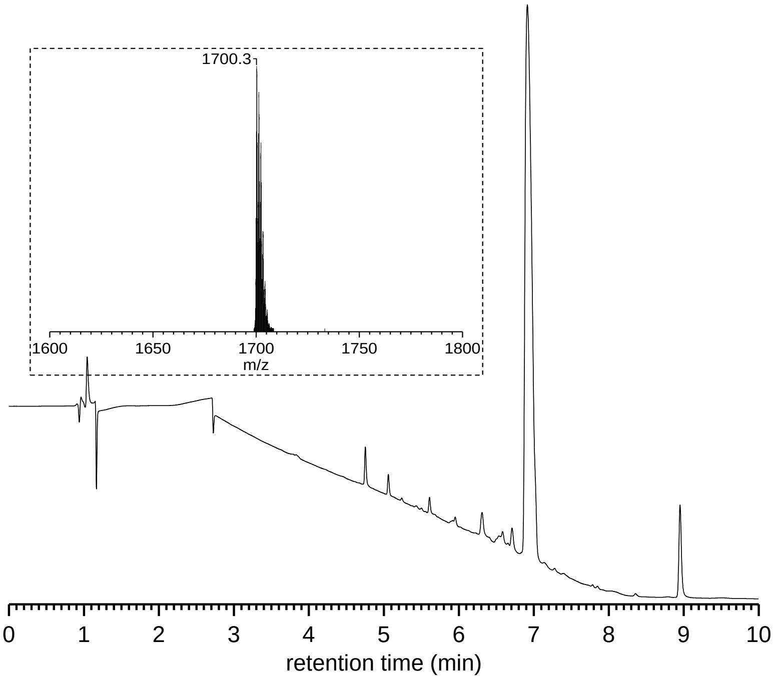

Supplementary Figure 69 | LC-ESI-MS analysis of **Z4NOT**. Insert: ESI-MS-spectrum of dominant species (negative mode).

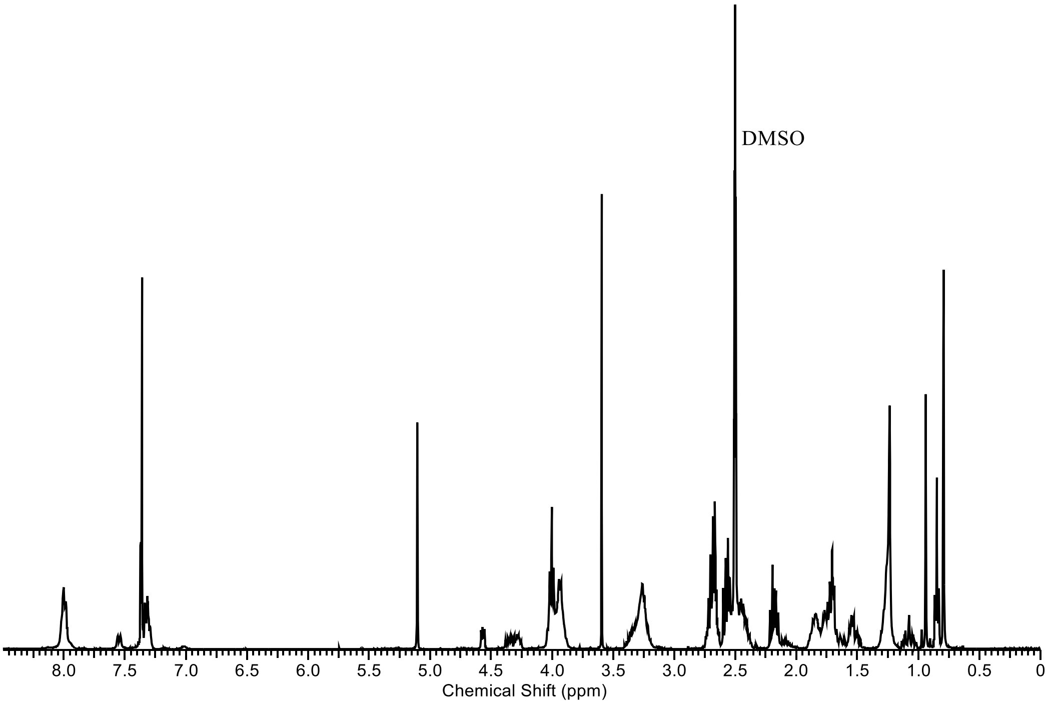

Supplementary Figure 70 | ^{1}H-NMR spectrum (500 MHz, DMSO-d6) with peak assignment of **Z4NOT**.

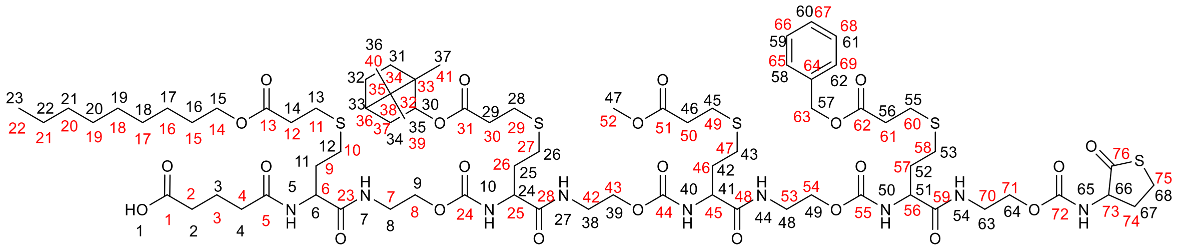

Supplementary Table 21 | determination of the ^{1}H- and ^{13}C-chemical shift values of **Z4NOT**

δ (ppm)	H	δ (ppm)	C
13.25	1	174.24	1
8.01	5, 7, 27, 44 and 54	171.69	5, 23, 28, 48 and 59
7.55	65	155.88	72
7.36	58-62	127.9, 127.99 and 128.42	65-69
7.33	10, 40 and 50	171.66	24, 44 and 55
5.11	57	65.59	63
4.58	30	80.3	32
4.35	66	59.84	73
4.31	6	51.81	6
4	15	64.02	14
3.97	64, 49, 39, 9 and 24, 41, 51	62.51 and 53.86	71, 54, 43, 8 and 25, 45, 56
3.6	47	51.41	52
3.3	68	26.36	75
3.27	8, 38, 48 and 63	38.15	7, 42, 53 and 70
2.72	55	26.11	60
2.69	13, 28 and 45	26.01 and 26.22	11, 29 and 49
2.64	14, 29 and 56	34.61, 34.33 and 34.24	12, 30 and 61
2.6	46	34.11	50
2.46	12, 26, 43 and 53	27.33 and 27.48	10, 27, 47 and 58
2.43	67	29.8	74
2.18	2 and 4	33.03 and 34.25	2 and 4
2.09	67	29.8	74
1.81	11, 25, 42 and 52	32.12	9, 26, 46 and 57
1.7	3 and 33, 34	20.61 and 44.37, 38.32	3 and 36, 37
1.63	31	26.6	34
1.55	16	28.11	15
1.5	32	33.24	35
1.24	17-22	22.10, 25.34, 28.64, 28.89 and 31.27	16-21
1.08	31 and 32	26.6 and 33.24	34 and 35
0.95	37	19.73	41
0.85	23	13.95	22
0.8	35 and 36	11.39 and 19.93	39 and 40
171.47	13	171.34	62
170.73	31	136.06	64
46.53	33	205.55	76

48.21	38	171.93	51

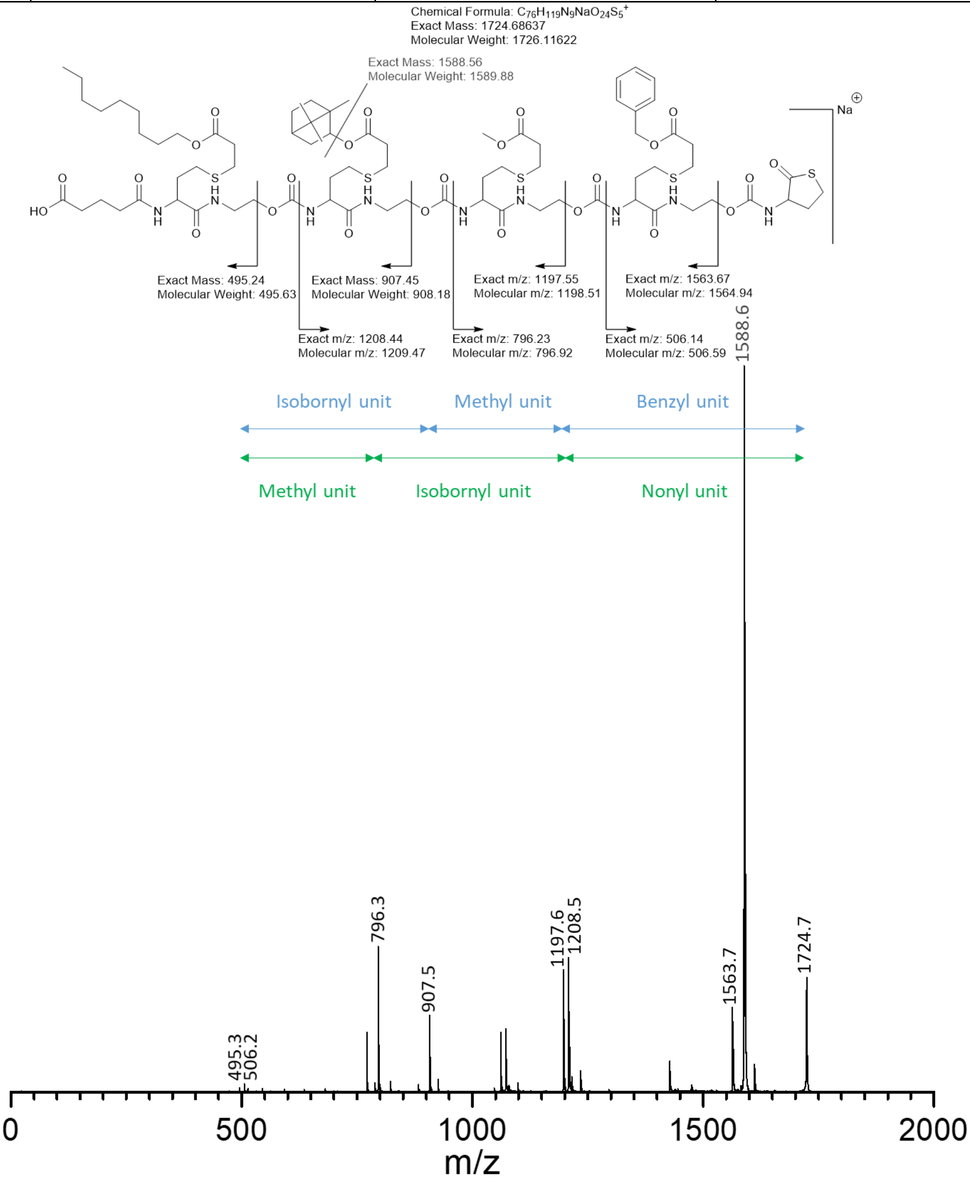

Supplementary Figure 71 | MALDI-MS/MS spectrum with peak assignment of **Z4NOT**.

*Characterization of **Z5TO** using mass spectrometry (Supplementary Figure 72), NMR spectroscopy (Supplementary Figure 73) and MALDI-MS/MS analysis (Supplementary Figure 74).*

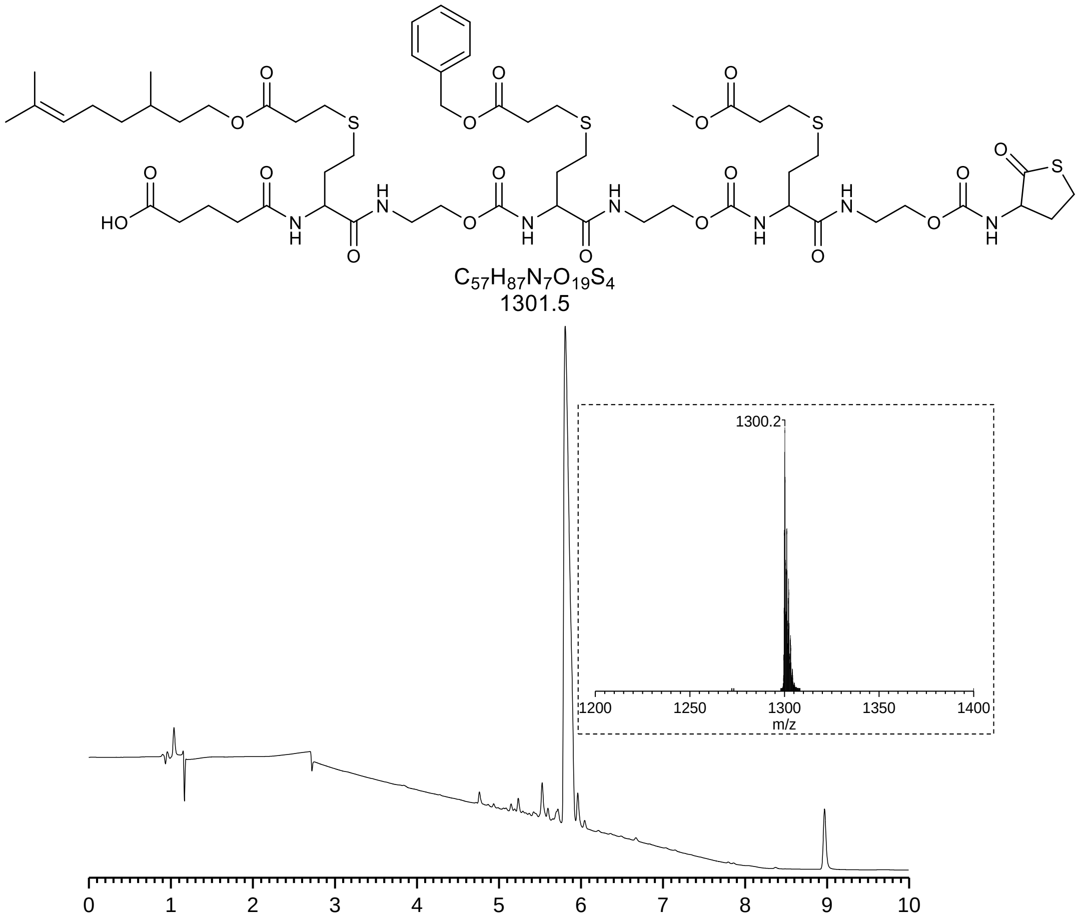

Supplementary Figure 72 | LC-ESI-MS analysis of **Z5TO**. Insert: ESI-MS-spectrum of dominant species (negative mode).

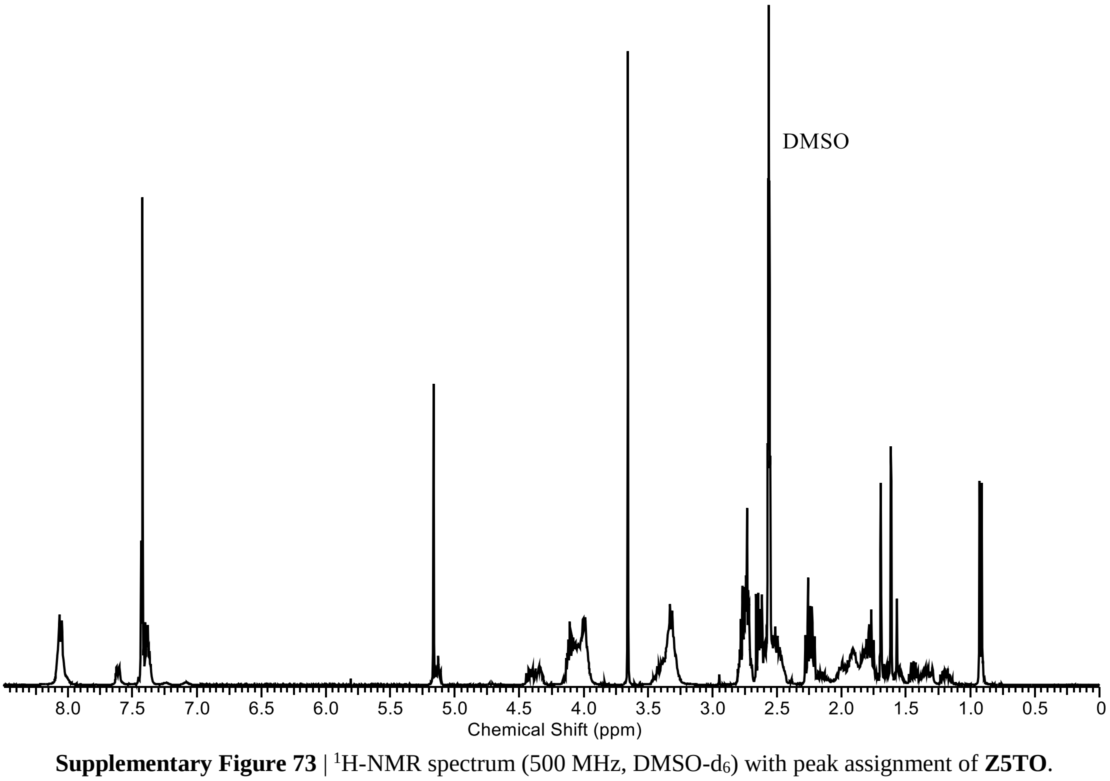

Supplementary Figure 73 | ^{1}H-NMR spectrum (500 MHz, DMSO-d_6) with peak assignment of **Z5TO**.

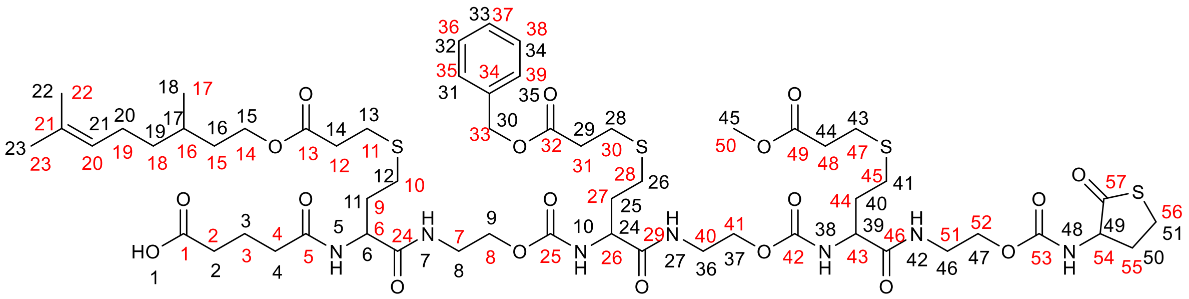

Supplementary Table 22 | determination of the ^{1}H- and ^{13}C-chemical shift values of **Z5TO**

δ (ppm)	H	δ (ppm)	C
13.25	1	174.24	1
8.01	5, 7, 27 and 42	171.69	5, 24, 29 and 46
7.55	48	155.88	53
7.36	31-35	127.9, 127.99 and 128.42	35-39
7.33	10 and 38	171.66	25 and 42
5.11	30	65.59	33
5.06	21	124.52	20
4.35	49	59.84	54
4.31	6	51.81	6
4.05	15	62.36	14
3.97	47, 37, 9 and 24, 39	62.51 and 53.86	52, 41, 8 and 26, 43
3.6	45	51.41	50
3.3	51	26.36	56
3.27	8, 36 and 46	38.15	7, 40 and 51
2.72	28	26.11	30
2.69	13 and 43	26.01	11 and 47
2.64	14 and 29	34.32 and 34.25	12 and 31
2.6	44	34.11	48
2.46	12, 26 and 41	27.33 and 27.48	10, 28 and 45
2.43/2.09	50	29.8	55
2.18	2 and 4	33.03 and 34.25	2 and 4
1.93	20	24.86	19
1.81	11, 25 and 40	32.07 and 32.21	9, 27 and 44
1.7	3	20.61	3
1.63/1.55	22 and 23	17.51 and 25.51	22 and 23
1.59/1.38	16	34.93	15
1.51	17	28.84	16
1.28/1.13	19	36.46	18
0.86	18	19.11	17
		171.34	32
		136.06	34
		205.55	57
		171.93	49
		171.45	13
		130.60	21

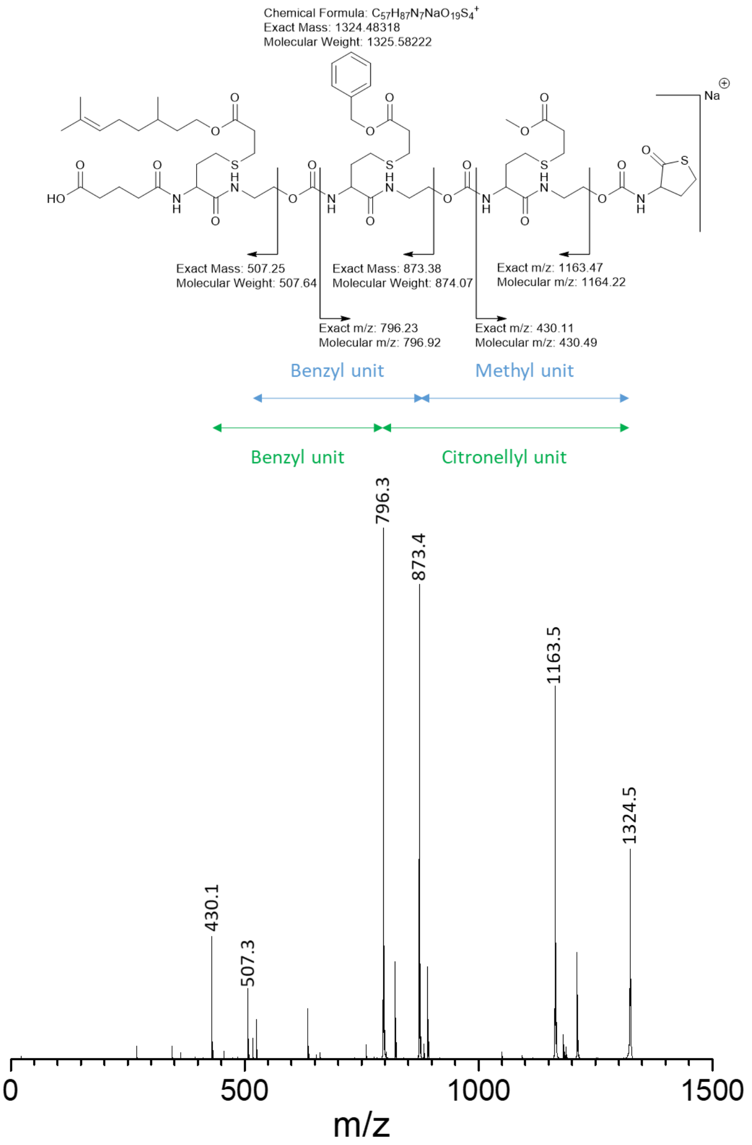

Supplementary Figure 74 | MALDI-MS/MS spectrum with peak assignment of **Z5TO**.

*Characterization of **Z6WRITE** using mass spectrometry (*Supplementary *Figure 75), NMR spectroscopy (*Supplementary *Figure 76) and MALDI-MS/MS analysis (*Supplementary *Figure 77).*

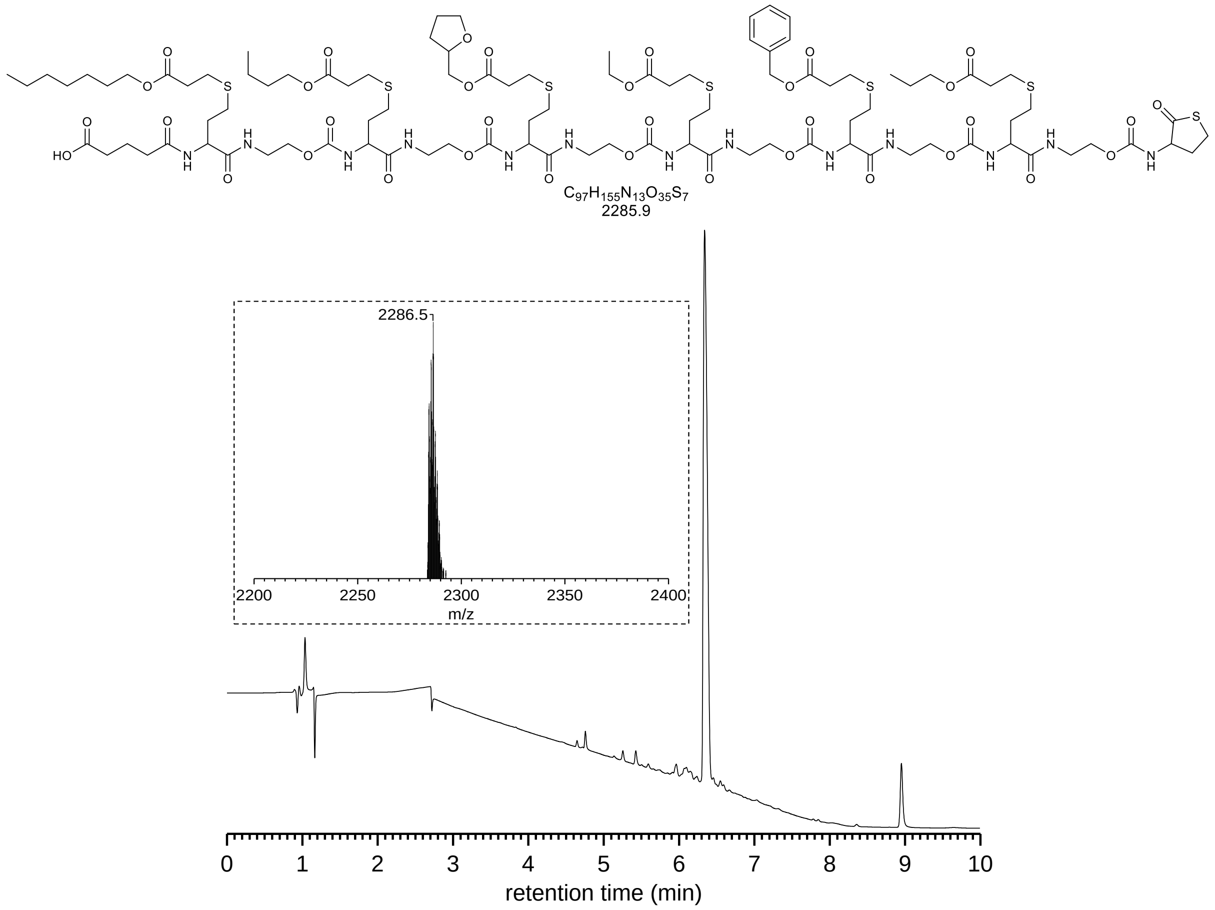

Supplementary Figure 75 | LC-ESI-MS analysis of **Z6WRITE**. Insert: ESI-MS-spectrum of dominant species (negative mode).

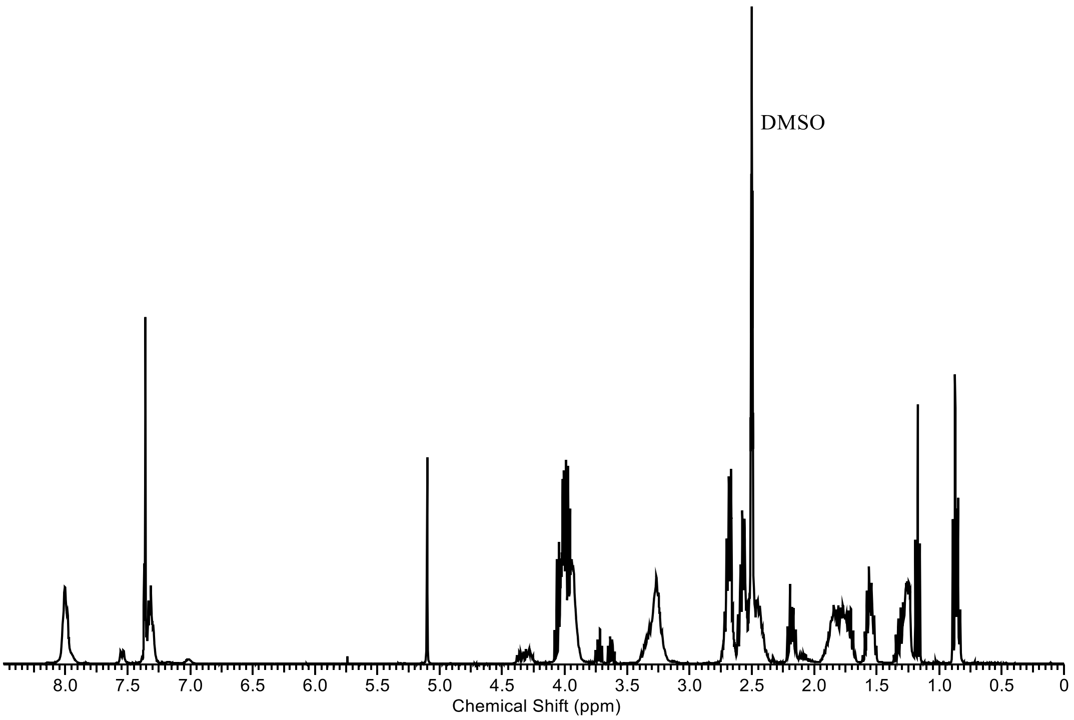

Supplementary Figure 76 | ^{1}H-NMR spectrum (500 MHz, DMSO-d_6) with peak assignment of **Z6WRITE**.

Supplementary Table 23 | determination of the ^{1}H- and ^{13}C-chemical shift values of **Z6WRITE**

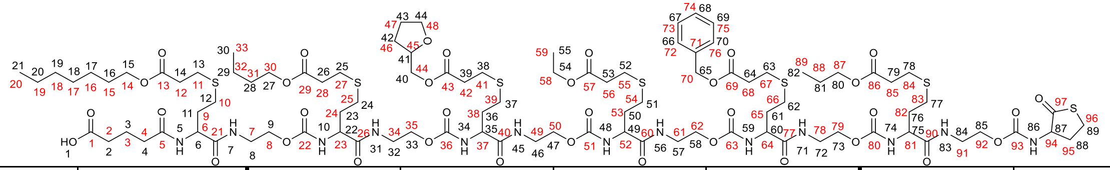

δ (ppm)	H	δ (ppm)	C	δ (ppm)	H	δ (ppm)	C	δ (ppm)	H	δ (ppm)	C
13.25	1	174.24	1	3.3	89	26.36	96	1.33	29	18.59	32
8.01	5, 7, 31, 45, 56, 71 and 83	171.69	5, 21, 26, 40, 60, 77 and 90	3.27	8, 32, 46, 57, 72 and 84	38.15 /33.86	7, 34, 49, 61, 78 and 91	1.26	17-20	22.04, 25.33, 28.32 and 31.18	16-19
7.55	86	155.88	93	2.72	63	26.11	67	1.18	55	14.11	59
7.36	66-70	127.9, 127.99 and 128.41	72-76	2.69	13, 25, 38, 52 and 78	26.05/26.08/ 26.18	11, 27, 41, 55 and 84	0.88	30 and 82	13.53 and 10.26	33 and 89
7.33	10, 34, 48, 59 and 74	155.88	22, 36, 51, 63 and 80	2.64	14, 26, 39, 53, 64 and 79	34.26/34.30/ 34.33	12, 28, 42, 56, 68 and 85	0.85	21	13.92	20
5.11	65	65.61	70	2.46	12, 24, 37, 51, 62 and 77	27.36/ 27.49	10, 25, 39, 54, 66 and 83				
4.35	87	59.88	94								
4.31	6	51.81	6	2.43	88	29.8	95				
4.04	54	60.07	58	2.18	2 and 4	33.03/ 34.25	2 and 4				
4.03	15	64.03	14	2.09	88	29.8	95	δ (ppm)	C	δ (ppm)	C
4.02	27 and 80	63.72 and 65.54	30 and 87	1.81	11, 23, 36, 50, 61 and 76; 42 and 43	32.05 and 32.18; 27.46 and 25.17	9, 24, 38, 53, 65 and 82; 46 and 47	171.34	69	171.49	29
4.01 and 4	40 and 41	66 and 75.75	44 and 45					136.06	71	171.52	57
3.97	85, 73, 58, 47, 33, 9 and 22, 35, 49, 60, 75	62.50 and 53.90	92, 79, 62, 50, 35, 8 and 23, 37, 52, 64, 81	1.7	3	20.61	3	205.55	97	171.54	86
				1.55	42, 28, 16 and 81	27.46, 30.16, 28.13 and 21.65	46, 31, 15 and 88	171.49	13		
3.73/3.63	44	67.42	48					171.4	43		

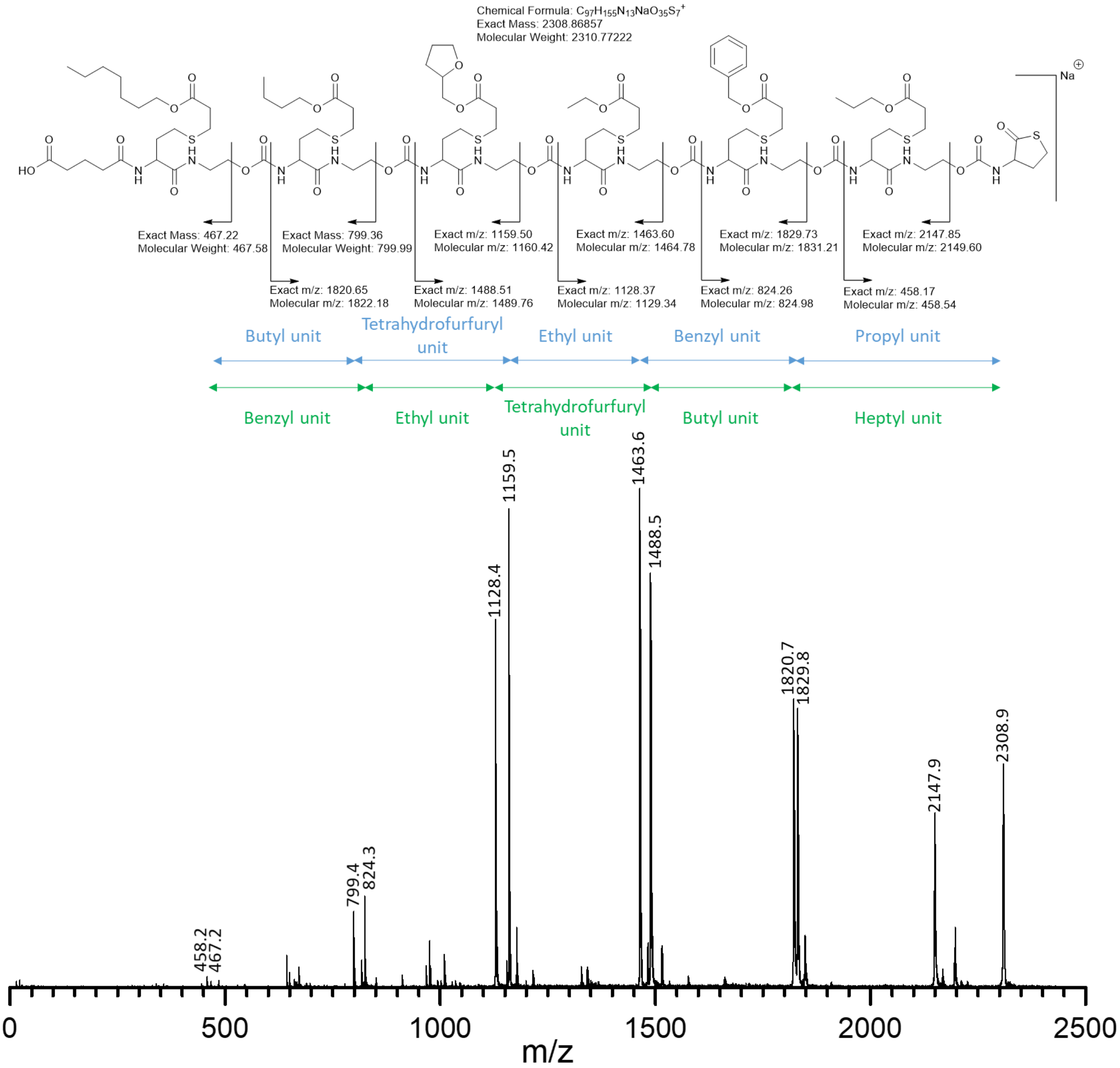

Supplementary Figure 77 | MALDI-MS/MS spectrum with peak assignment of **Z6WRITE**.

*Characterization of **Z7ON** using mass spectrometry (Supplementary Figure 78), NMR spectroscopy (Supplementary Figure 79) and MALDI-MS/MS analysis (Supplementary Figure 80).*

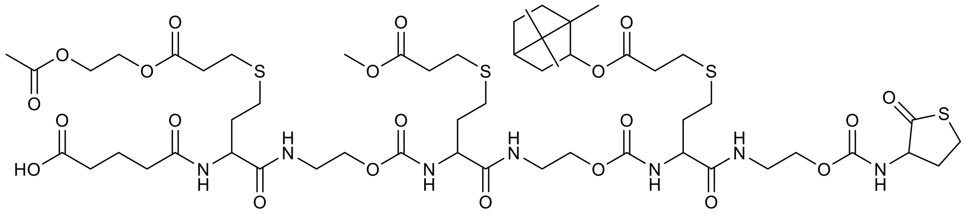

$C_{54}H_{85}N_7O_{21}S_4$
1295.5

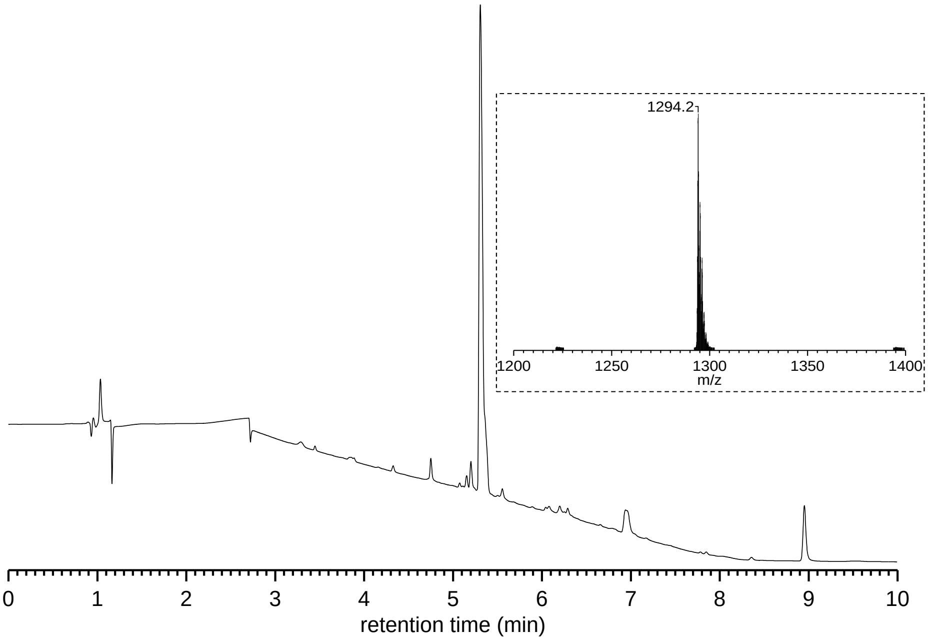

Supplementary Figure 78 | LC-ESI-MS analysis of **Z7ON**. Insert: ESI-MS-spectrum of dominant species (negative mode).

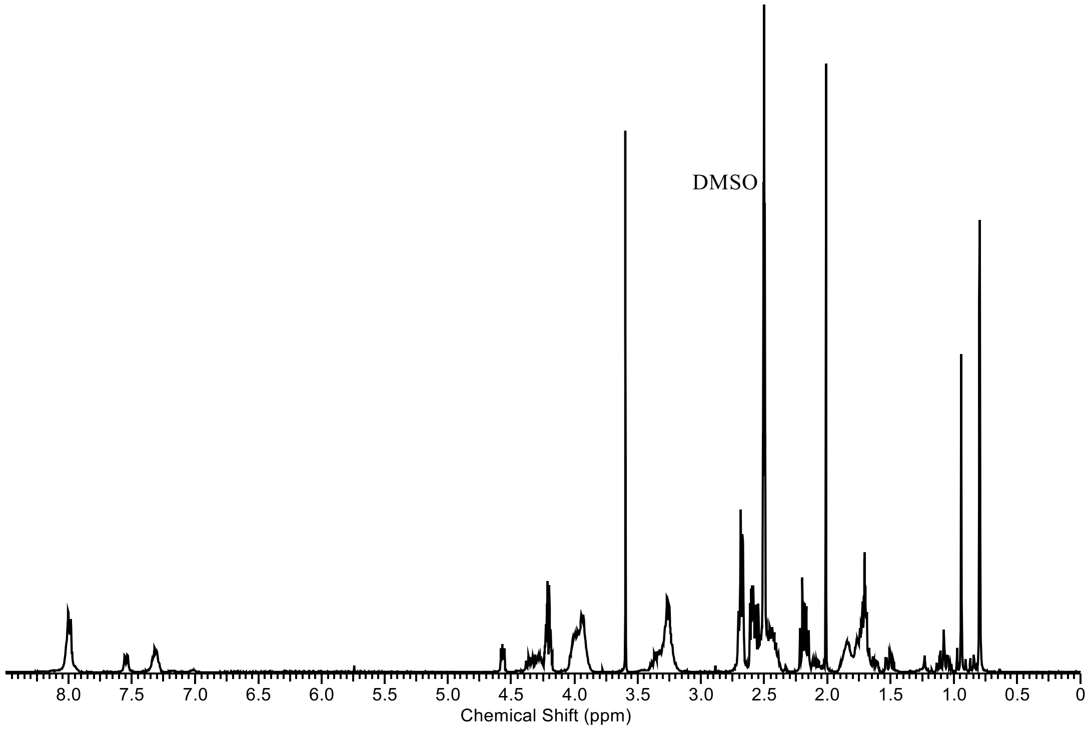

Supplementary Figure 79 | ^{1}H-NMR spectrum (500 MHz, DMSO-d$_6$) with peak assignment of **Z7ON**.

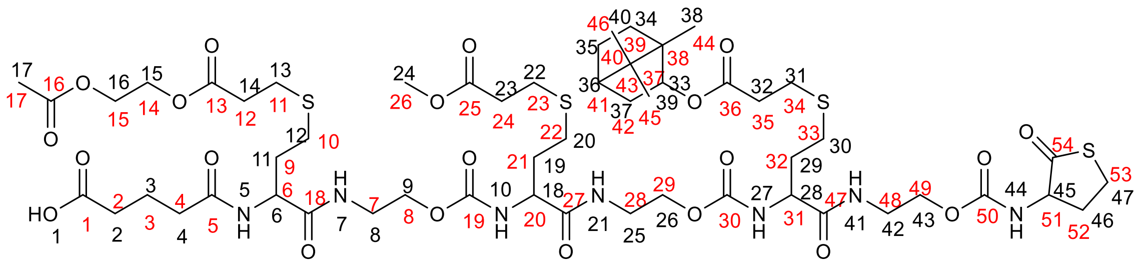

Supplementary Table 24 | determination of the ^{1}H- and ^{13}C-chemical shift values of **Z7ON**

δ (ppm)	H	δ (ppm)	C
13.25	1	174.24	1
8.01	5, 7, 21 and 41	171.69	5, 18, 27 and 47
7.55	44	155.88	50
7.33	10 and 27	171.66	19 and 30
4.58	33	80.3	37
4.35	45	59.84	51
4.31	6	51.81	6
4.21	15 and 16	62.11 and 61.87	14 and 15
3.97	43, 26, 9 and 18, 28	62.51 and 53.86	49, 29, 8 and 20, 31
3.6	24	51.41	26
3.3	47	26.36	53
3.27	8, 25 and 42	38.15	7, 28 and 48
2.69	13, 22 and 31	26.01 and 26.22	11, 23 and 34
2.64	14 and 32	34.17 and 34.61	12 and 35
2.6	23	34.11	24
2.46	12, 20 and 30	27.33 and 27.48	10, 22 and 33
2.43	46	29.8	52
2.18	2 and 4	33.03 and 34.25	2 and 4
2.09	46	29.8	52
2.01	17	20.61	17
1.81	11, 19 and 29	32.12	9, 21 and 32
1.7	3 and 36, 37	20.61 and 44.37, 38.32	3 and 41, 42
1.63	34	26.6	39
1.5	35	33.24	40
1.08	34 and 35	26.6 and 33.24	39 and 40
0.95	38	19.73	44
0.8	39 and 40	11.39 and 19.93	45 and 46
		171.39	13
		170.28	16
		205.55	54
		171.93	25
		170.73	36
		46.53	38
		48.21	43

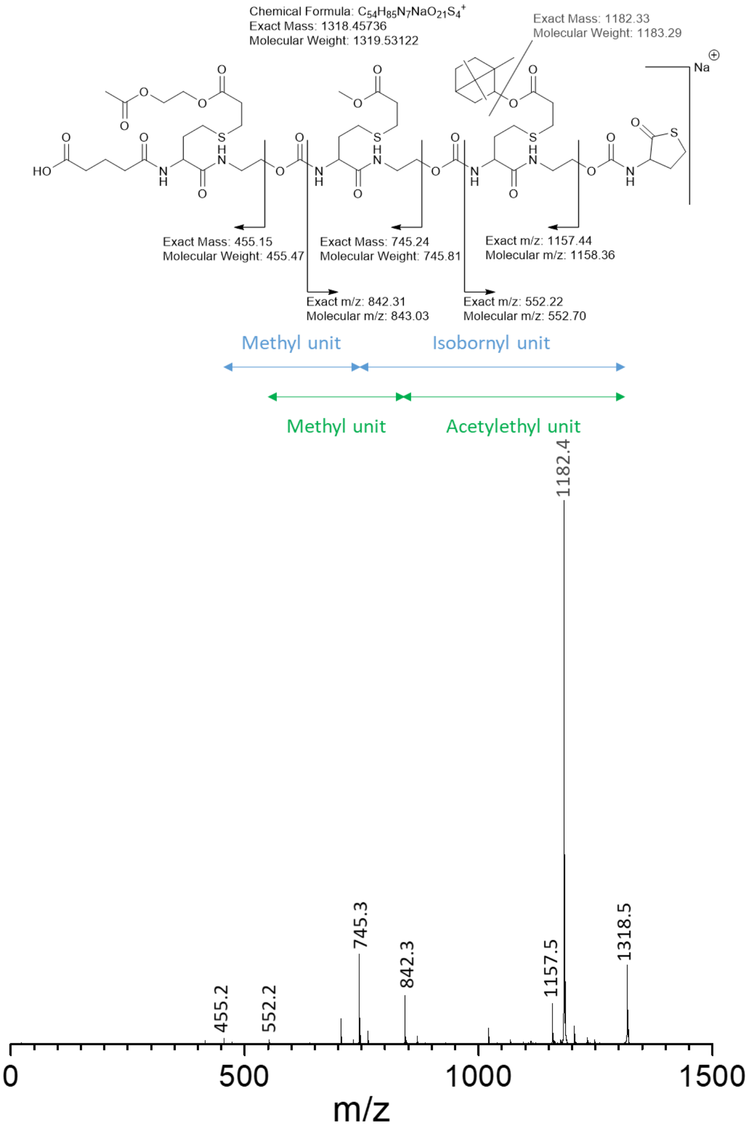

Supplementary Figure 80 | MALDI-MS/MS spectrum with peak assignment of **Z7ON**.

*Characterization of **Z8OLIGOS?** using mass spectrometry (Supplementary Figure 81), NMR spectroscopy (Supplementary Figure 82) and MALDI-MS/MS analysis (Supplementary Figure 83).*

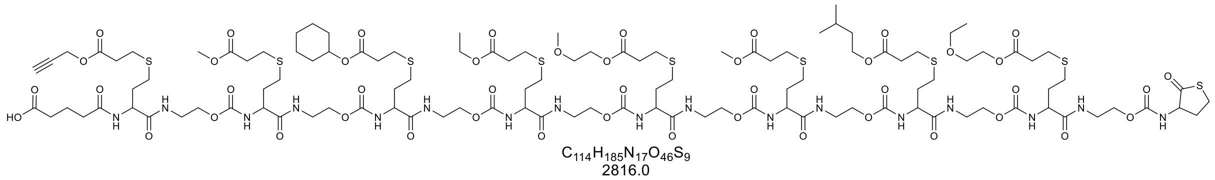

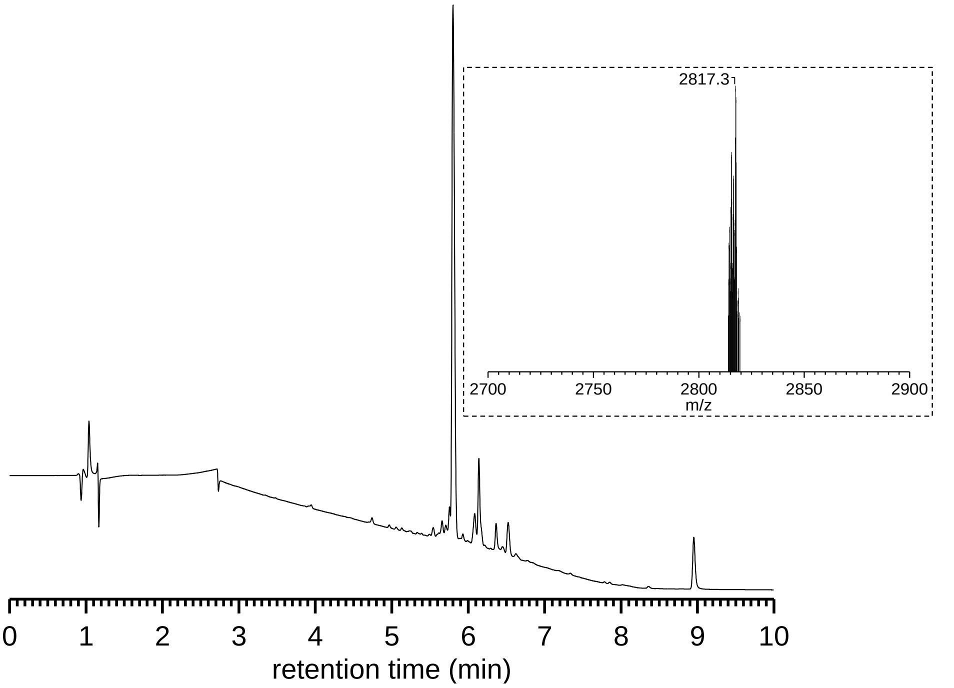

***Supplementary Figure 81** | LC-ESI-MS analysis of **Z8OLIGOS?**. Insert: ESI-MS-spectrum of dominant species (negative mode).*

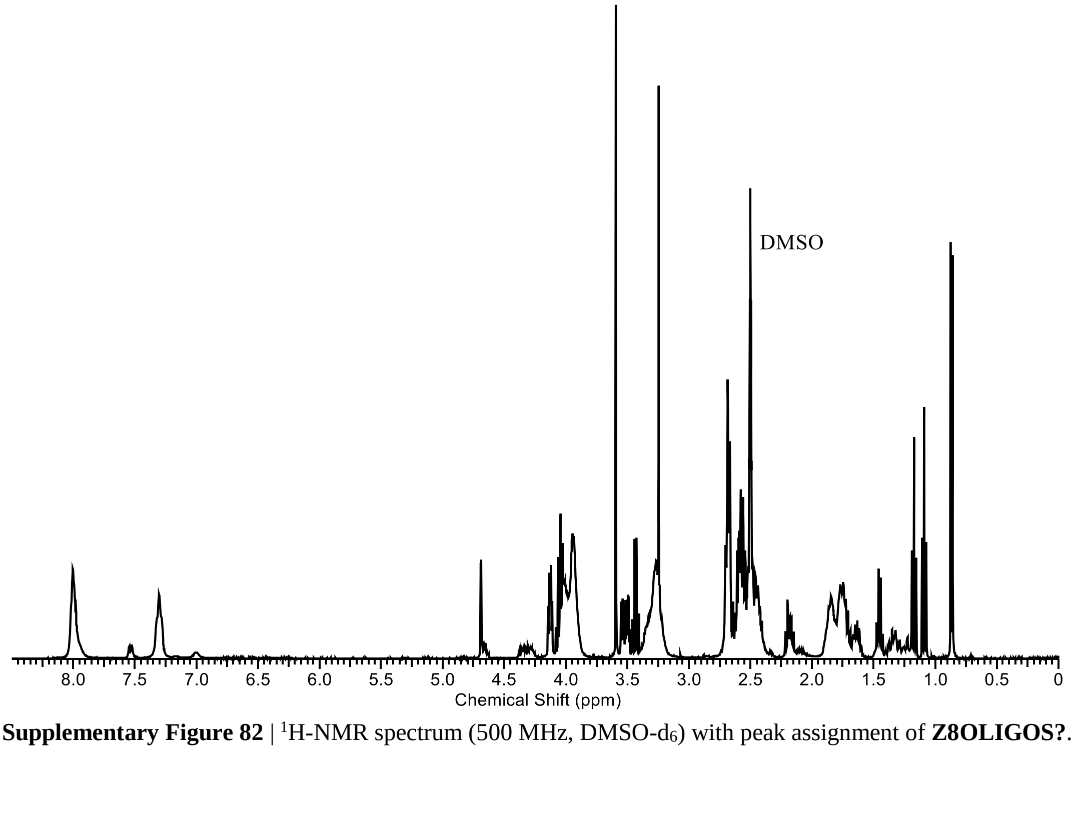

Supplementary Figure 82 | ^{1}H-NMR spectrum (500 MHz, DMSO-d_6) with peak assignment of **Z8OLIGOS?**.

Supplementary Table 25 | determination of the ^{1}H- and ^{13}C-chemical shift values of **Z8OLIGOS?**

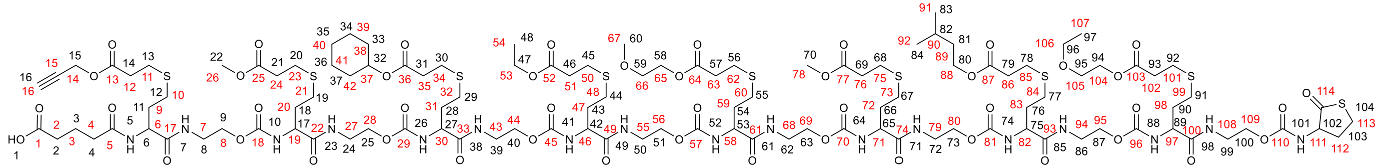

δ (ppm)	H	δ (ppm)	C
13.25	1	174.24	1
8.01	5, 7, 23, 38, 49, 61, 71, 85 and 98	171.76	5, 17, 22, 33, 49, 61, 74, 93 and 100
7.55	101	156.01	110
7.33	10, 26, 41, 52, 64, 74 and 88	156.01	18, 29, 45, 57, 70, 81 and 96
4.69	15	51.89	14
4.66	32	72.14	37
4.35	102	59.95	111
4.31	6	51.93	6
4.13	58 and 94	63.56 and 65.30	65 and 104
4.04	47 and 80	60.07 and 62.62	53 and 88
3.97	100, 87, 73, 63, 51, 40, 25, 9 and 17, 27, 42, 53, 65, 75, 89	62.57 and 53.98	109, 95, 80, 69, 56, 44, 28, 8 and 19, 30, 46, 58, 71, 82, 97
3.60	22 and 70	51.47	26 and 78
3.52	59 and 95	67.71 and 69.79	66 and 105

δ (ppm)	H	δ (ppm)	C
3.49	16	77.72	16
3.43	96	65.66	106
3.3	104	26.30	113
3.27	8, 24, 39, 50, 62, 72, 86 and 99	38.22	7, 27, 43, 55, 68, 79, 94 and 108
3.25	60	58.10	67
2.69	13, 20, 30, 45, 56, 68, 78 and 92	26.01/26.09/ 26.12/26.15/ 26.19/26.47	11, 23, 34, 50, 62, 75, 85 and 101
2.64	14, 31, 46, 57, 79 and 93	34.34/ 34.41/34.71	12, 35, 51, 63, 86 and 102
2.6	21 and 69	34.11	24 and 76
2.46	12, 19, 29, 44, 55, 67, 77 and 91	27.60	10, 21, 32, 48, 60, 73, 84 and 99
2.43	103	29.8	112
2.18	2 and 4	33.03/ 34.22	2 and 4
2.09	103	29.8	112
1.81	11, 18, 28, 43, 54, 66, 76 and 90	32.17	9, 20, 31, 47, 59, 72, 83 and 98

δ (ppm)	H	δ (ppm)	C
1.76-1.7	3, 33 and 37	20.61/31.17	3, 38 and 42
1.63	82 and 34, 36	24.59 and 23.24	90 and 39, 41
1.45	81 and 35	36.94 and 24.95	89 and 40
1.33	33, 37 and 34, 36	31.17 and 23.24	38, 42 and 39, 41
1.22	35	24.95	40
1.17	48	14.11	54
1.09	97	15.07	107
0.87	83 and 84	22.33	91 and 92
**δ (ppm)**	**C**	**δ (ppm)**	**C**
171.58	87	171.59 and 171.61	64 and 103
78.41	15		
205.55	114	171.52	52
170.93	13	170.89	36
171.93	25 and 77		

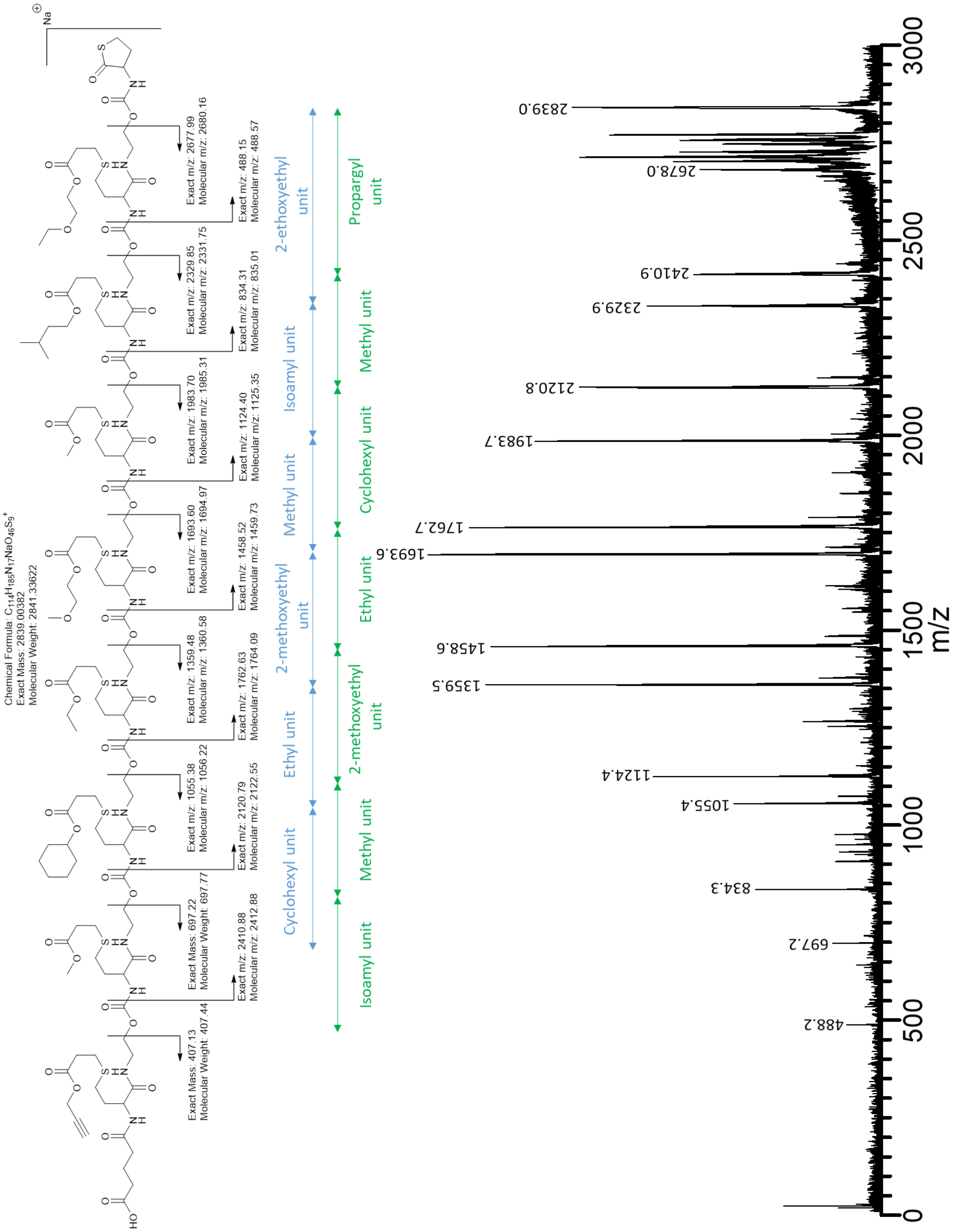

Supplementary Figure 83 | MALDI-MS/MS spectrum with peak assignment of **Z8OLIGOS?**.

QR CODE

Supplementary Table 26 | The alphabet (the different functionalities) used to write the QR code on sequence-defined poly(amide-urethanes).

Symbol	Acrylate Functionality	Symbol	Acrylate Functionality
A	Cyclohexyl	I	2-(Dimethylamino)ethyl
B	Benzyl	J	Isobornyl
C	Tetrahydrofurfuryl	K	2-(2-ethoxyethoxy)ethyl
D	Butyl	L	Propyl
E	Methyl	M	Heptyl
F	Ethyl	N	Ethylhexyl
G	Isoamyl	O	2-Methoxyethyl
H	Diethylacrylamide		

*Characterization of **QR1** using mass spectrometry (Supplementary Figure 84) and MALDI-MS/MS analysis (Supplementary Figure 85).*

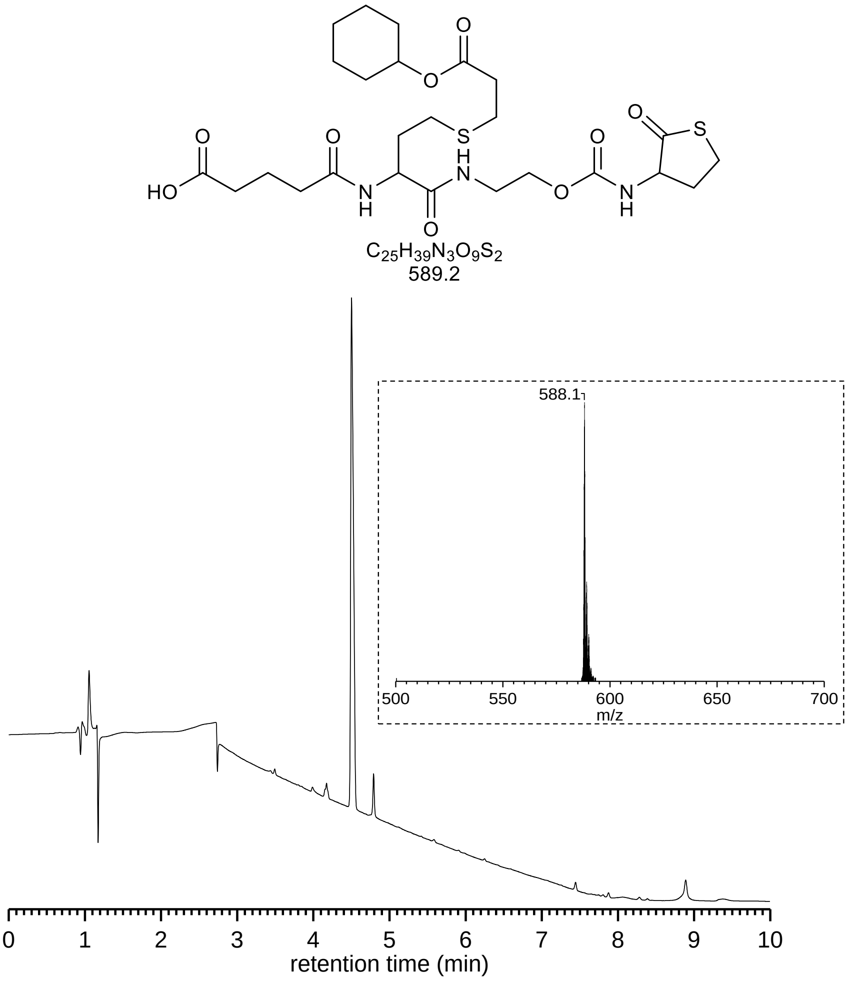

Supplementary Figure 84 | LC-ESI-MS analysis of **QR1**. Insert: ESI-MS-spectrum of dominant species (negative mode).

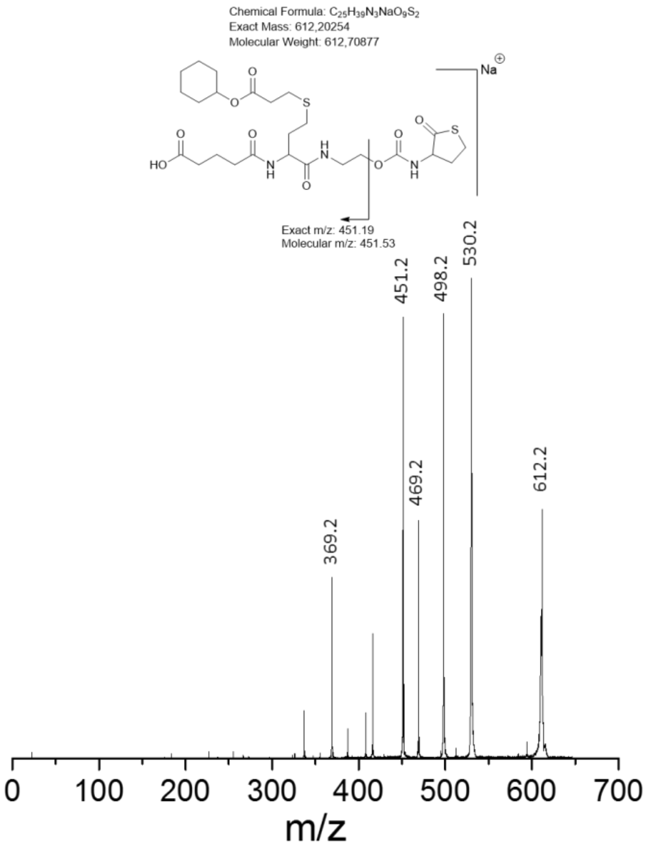

Supplementary Figure 85 | MALDI-MS/MS spectrum with peak assignment of **QR1**.

*Characterization of **QR2** using mass spectrometry (Supplementary Figure 86) and MALDI-MS/MS analysis (Supplementary Figure 87).*

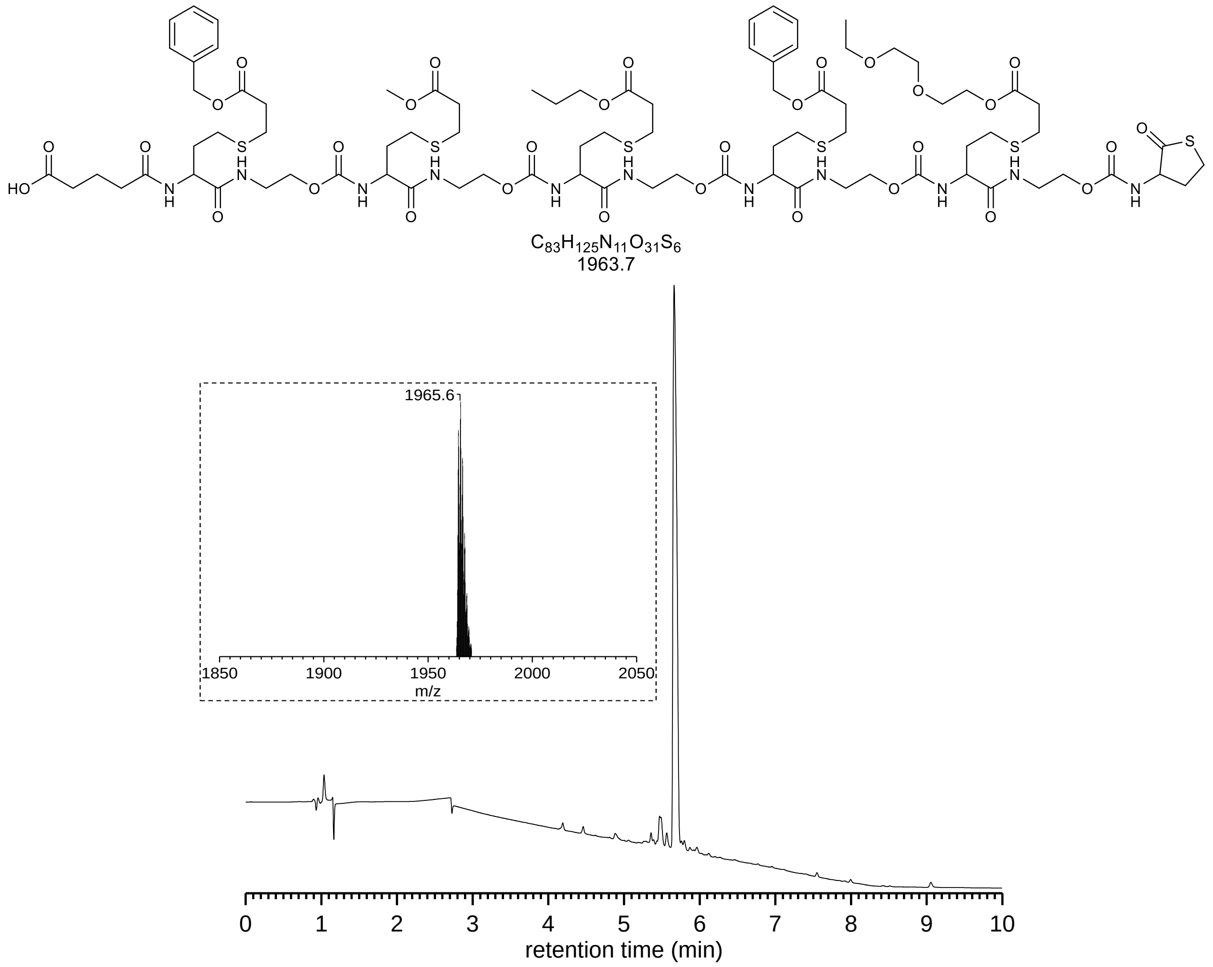

Supplementary Figure 86 | LC-ESI-MS analysis of **QR2**. Insert: ESI-MS-spectrum of dominant species (positive mode).

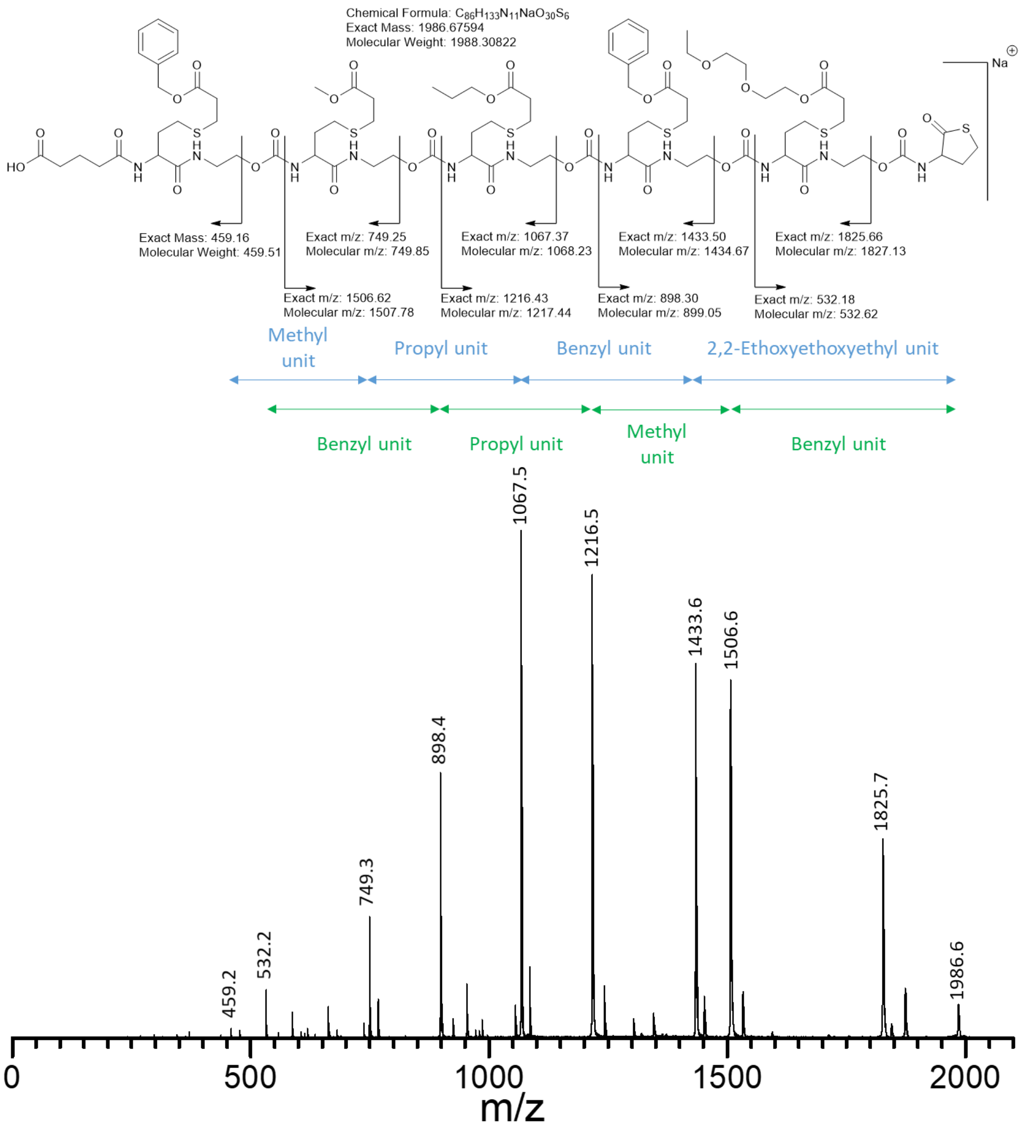

Supplementary Figure 87 | MALDI-MS/MS spectrum with peak assignment of **QR2**.

*Characterization of **QR3** using mass spectrometry (Supplementary Figure 88) and MALDI-MS/MS analysis (Supplementary Figure 89).*

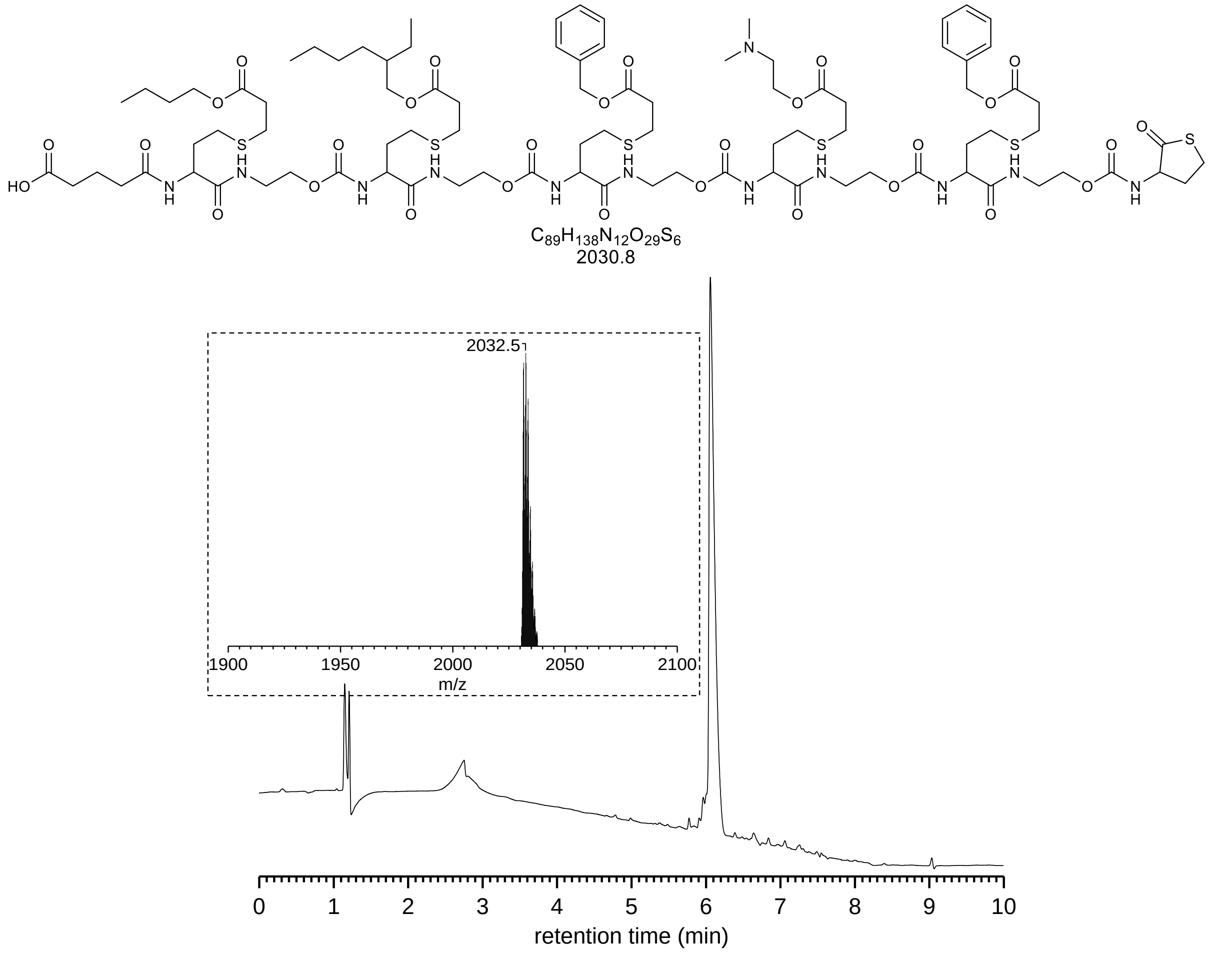

Supplementary Figure 88 | LC-ESI-MS analysis of **QR3**. Insert: ESI-MS-spectrum of dominant species (positive mode).

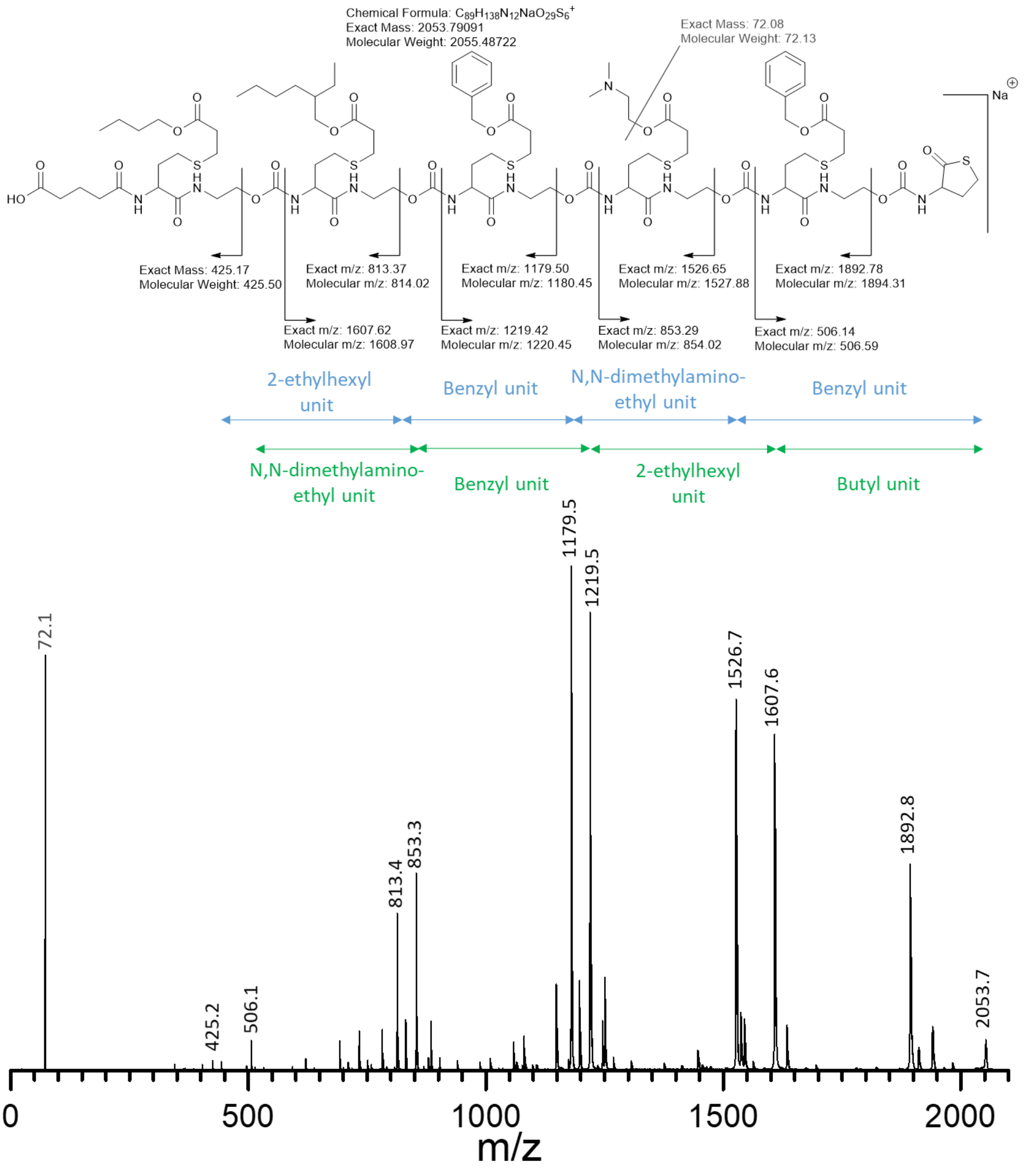

Supplementary Figure 89 | MALDI-MS/MS spectrum with peak assignment of **QR3**.

*Characterization of **QR4** using mass spectrometry (Supplementary Figure 90) and MALDI-MS/MS analysis (Supplementary Figure 91).*

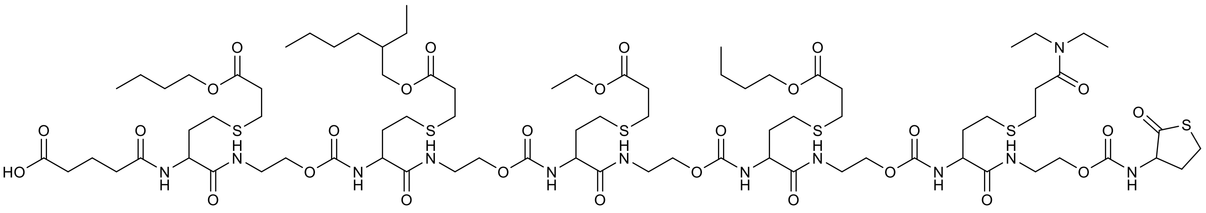

$C_{81}H_{138}N_{12}O_{28}S_6$
1918.8

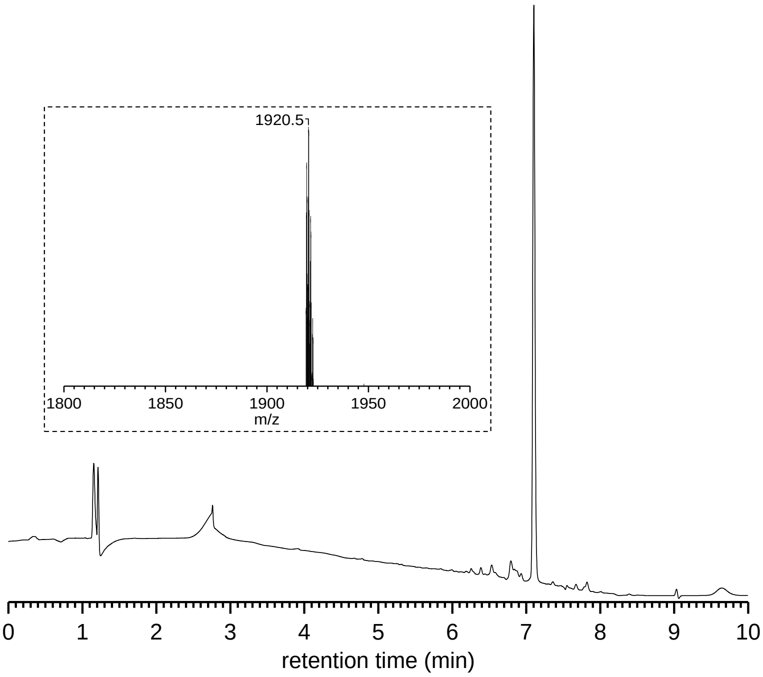

Supplementary Figure 90 | LC-ESI-MS analysis of **QR4**. Insert: ESI-MS-spectrum of dominant species (positive mode).

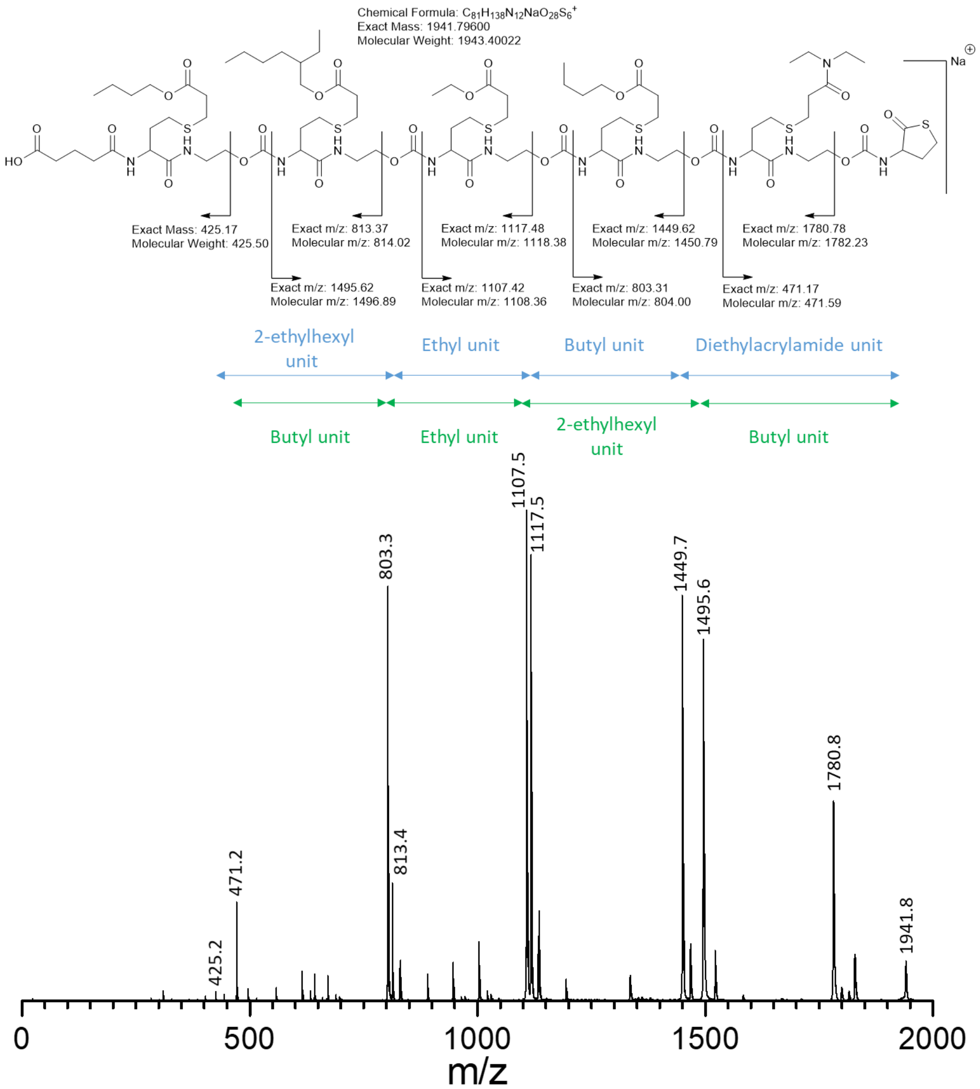

Supplementary Figure 91 | MALDI-MS/MS spectrum with peak assignment of **QR4**.

*Characterization of **QR5** using mass spectrometry (Supplementary Figure 92) and MALDI-MS/MS analysis (Supplementary Figure 93).*

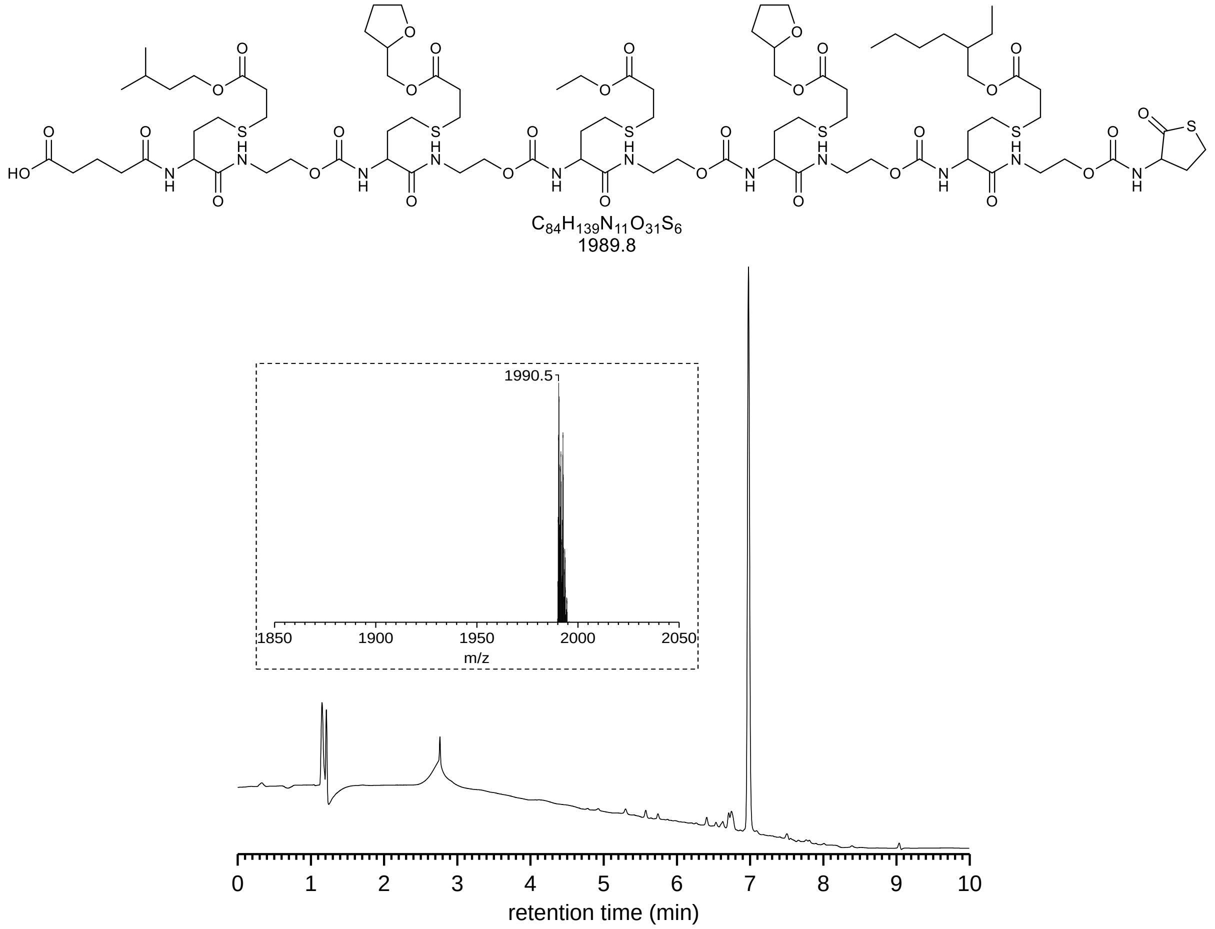

Supplementary Figure 92 | LC-ESI-MS analysis of **QR5**. Insert: ESI-MS-spectrum of dominant species (positive mode).

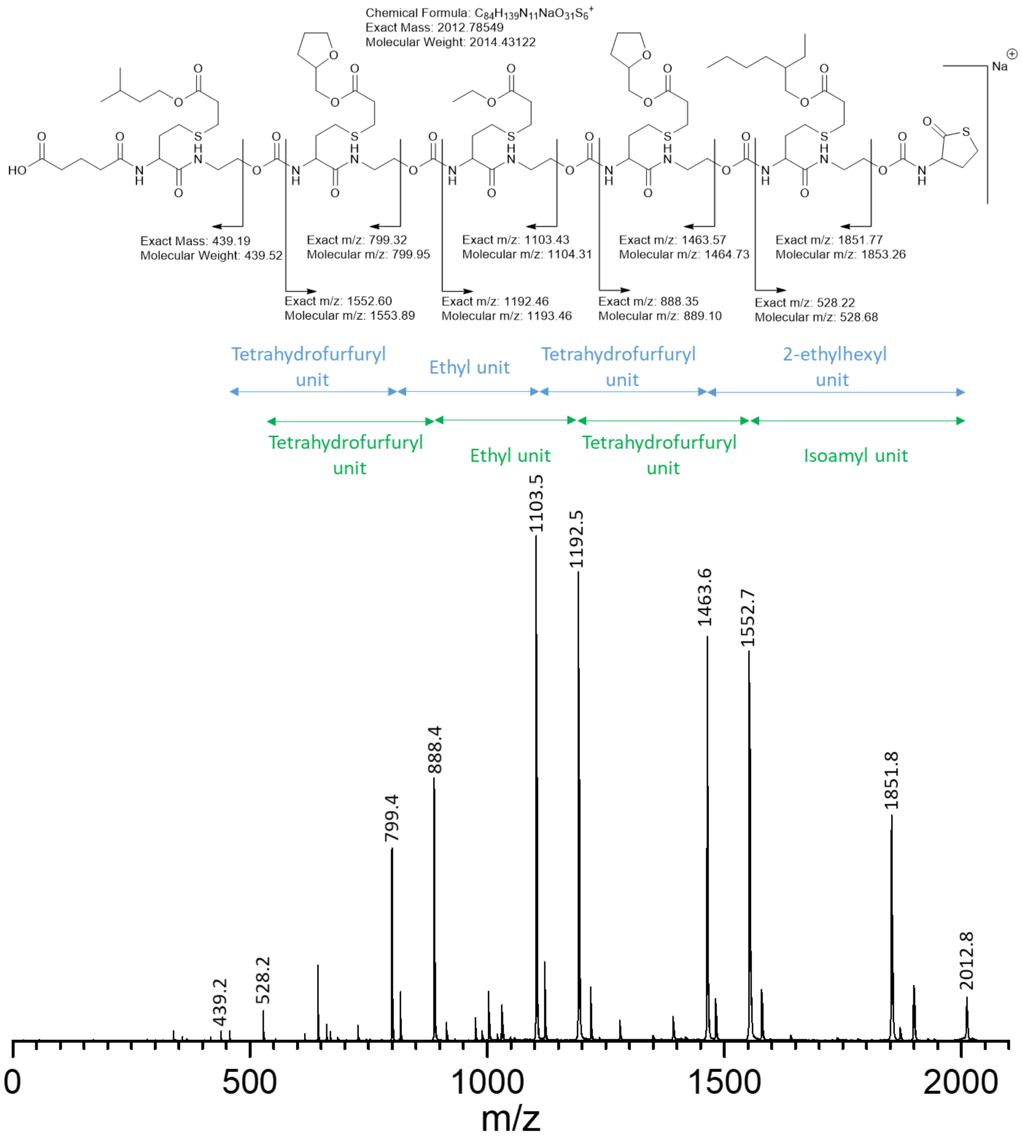

Supplementary Figure 93 | MALDI-MS/MS spectrum with peak assignment of **QR5**.

*Characterization of **QR6** using mass spectrometry (Supplementary Figure 94) and MALDI-MS/MS analysis (Supplementary Figure 95).*

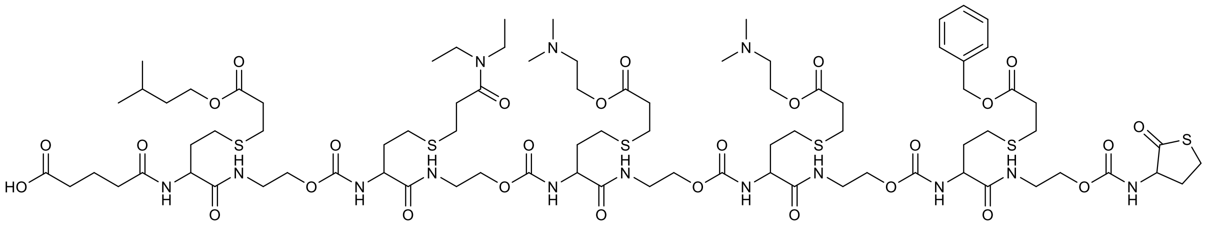

$C_{83}H_{136}N_{14}O_{28}S_6$
1968.8

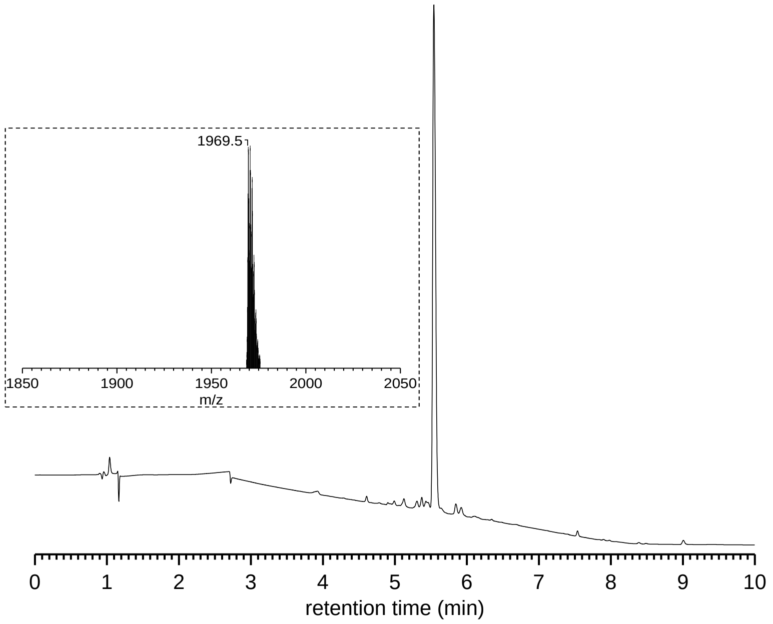

Supplementary Figure 94 | LC-ESI-MS analysis of **QR6**. Insert: ESI-MS-spectrum of dominant species (positive mode).

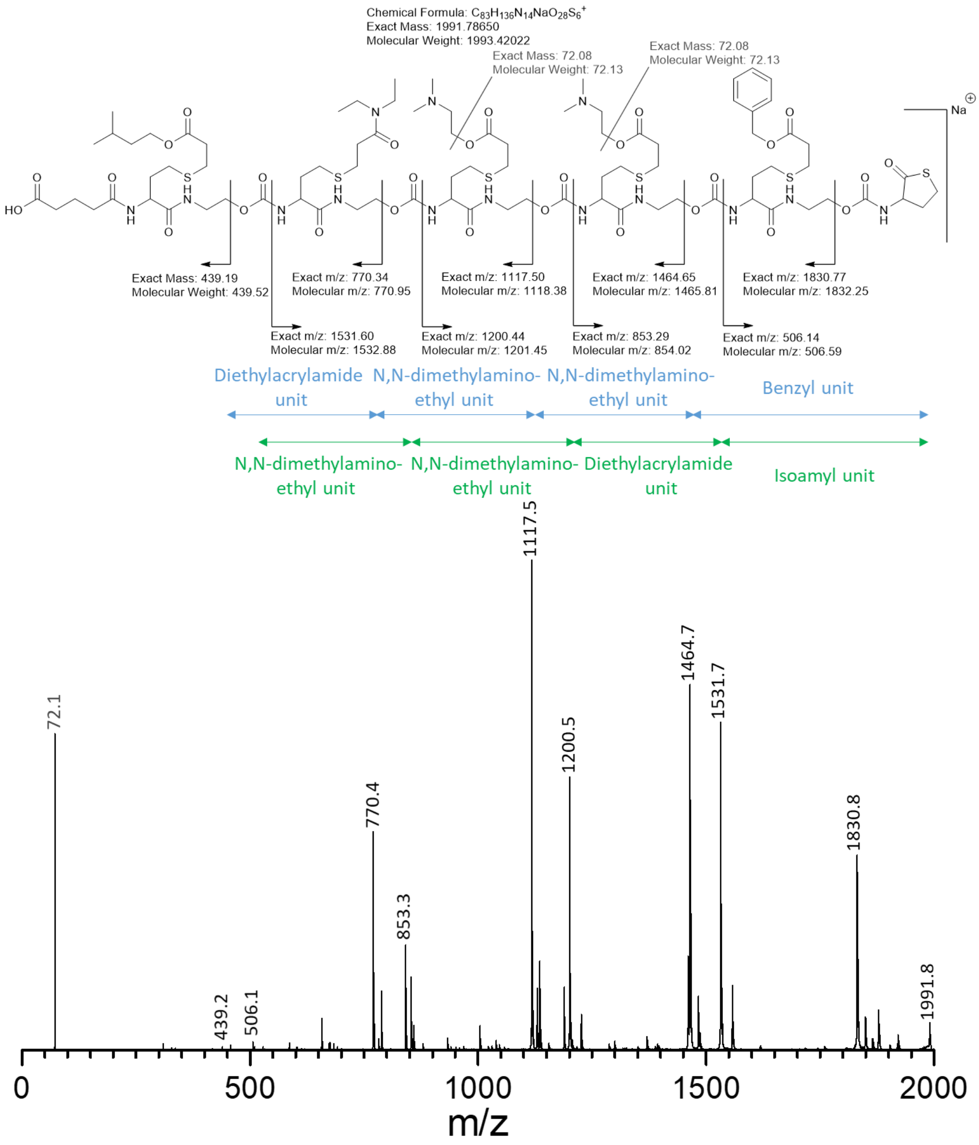

Supplementary Figure 95 | MALDI-MS/MS spectrum with peak assignment of **QR6**.

*Characterization of **QR7** using mass spectrometry (Supplementary Figure 96) and MALDI-MS/MS analysis (Supplementary Figure 97).*

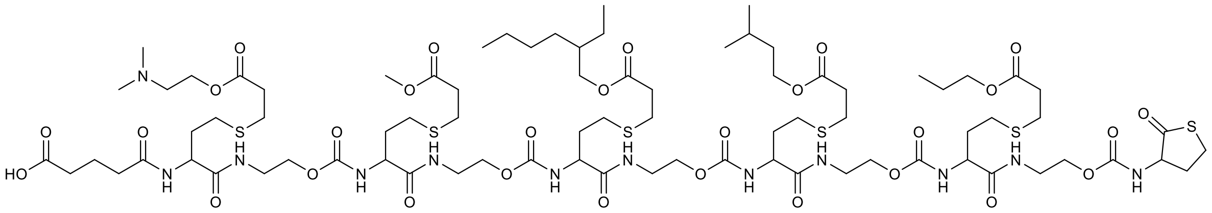

$C_{80}H_{136}N_{12}O_{29}S_6$
1920.78604

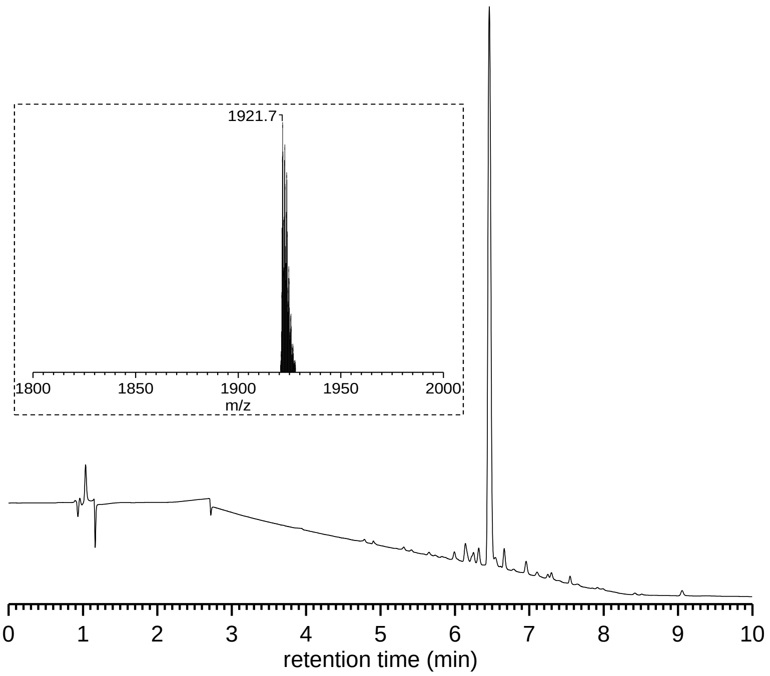

Supplementary Figure 96 | LC-ESI-MS analysis of **QR7**. Insert: ESI-MS-spectrum of dominant species (positive mode).

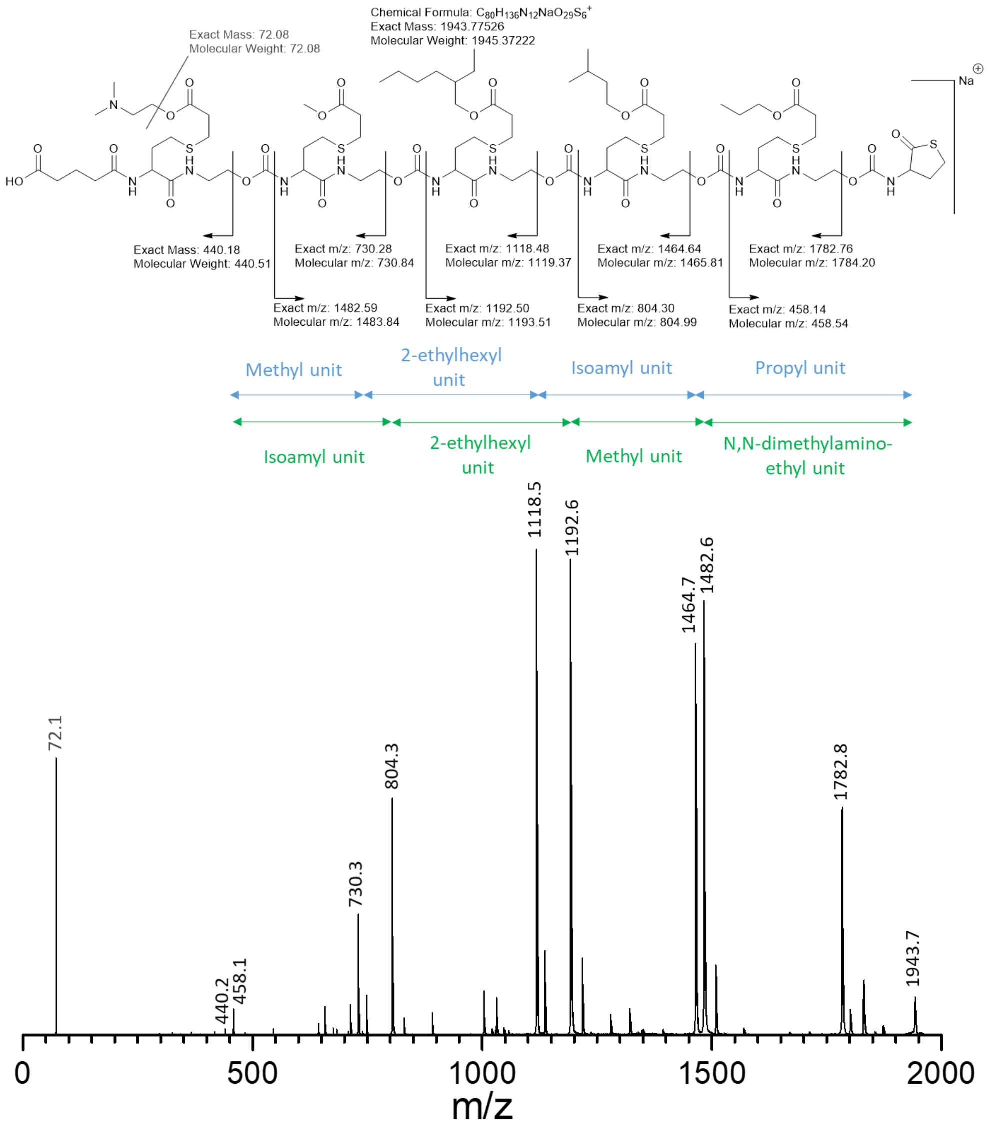

Supplementary Figure 97 | MALDI-MS/MS spectrum with peak assignment of **QR7**.

*Characterization of **QR8** using mass spectrometry (Supplementary Figure 98) and MALDI-MS/MS analysis (Supplementary Figure 99).*

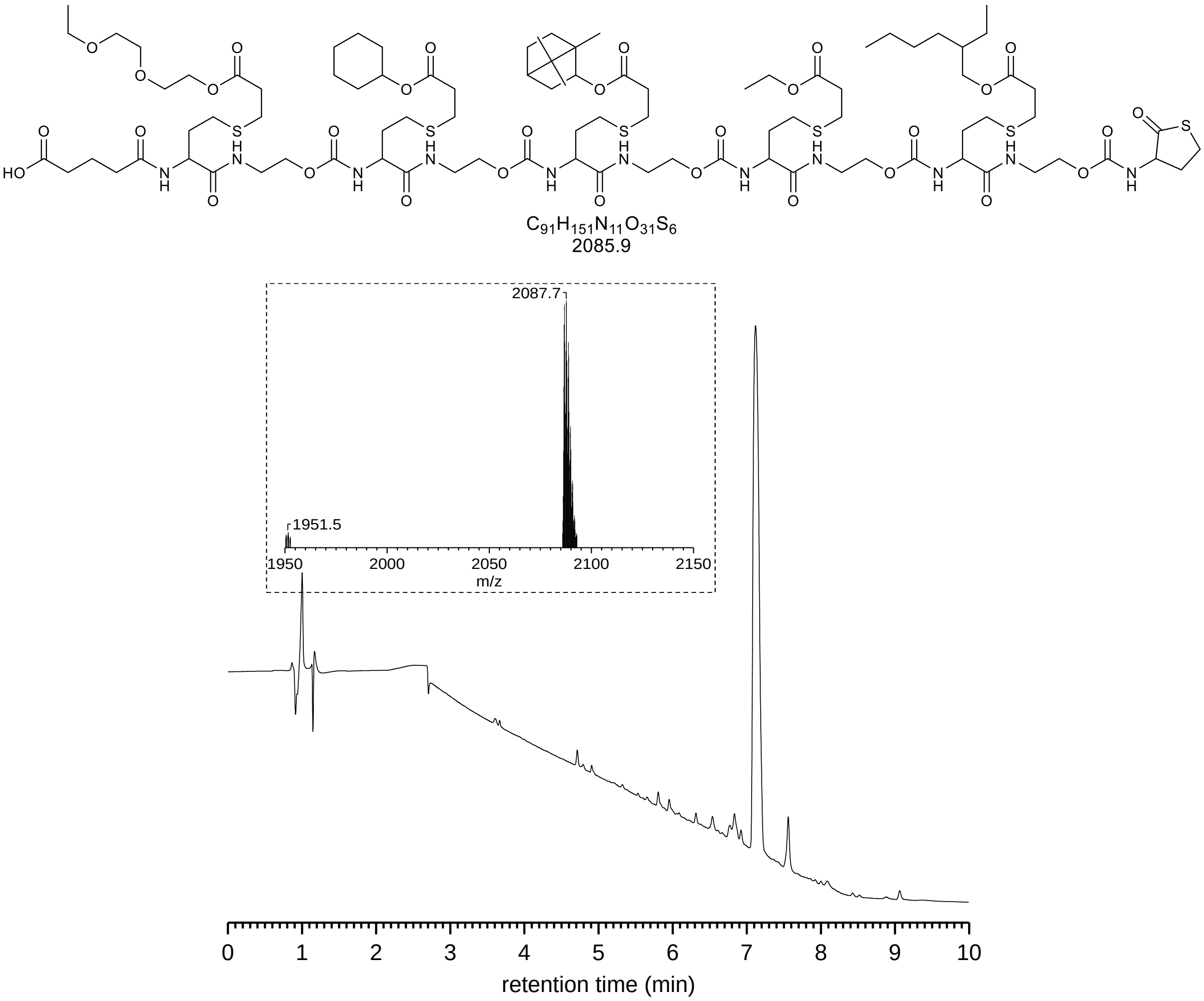

Supplementary Figure 98 | LC-ESI-MS analysis of **QR8**. Insert: ESI-MS-spectrum of dominant species (positive mode).

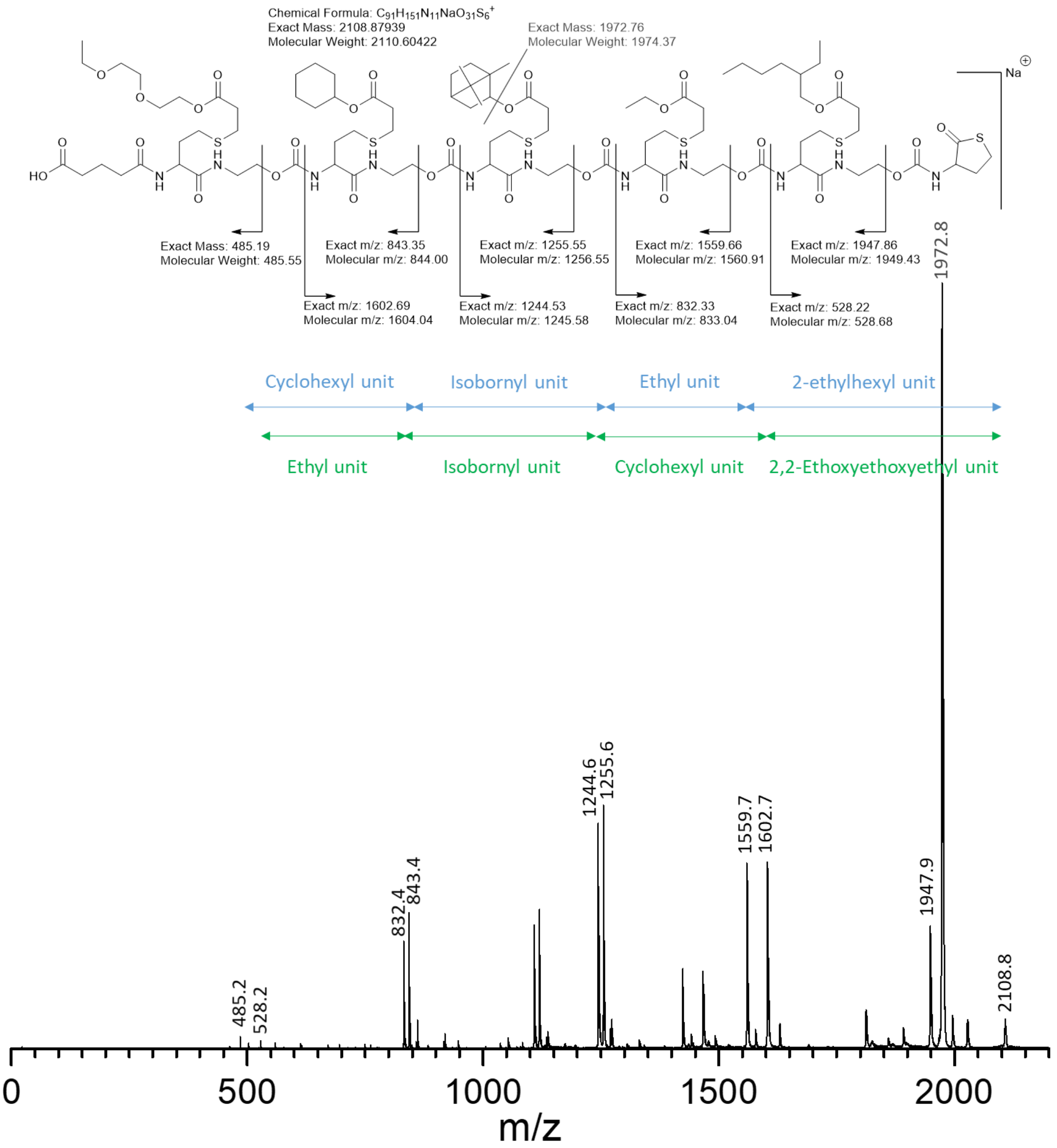

Supplementary Figure 99 | MALDI-MS/MS spectrum with peak assignment of **QR8**.

*Characterization of **QR9** using mass spectrometry (Supplementary Figure 100) and MALDI-MS/MS analysis (Supplementary Figure 101).*

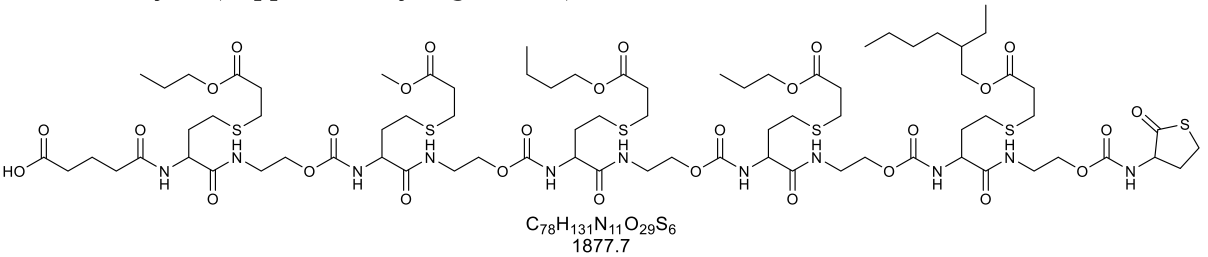

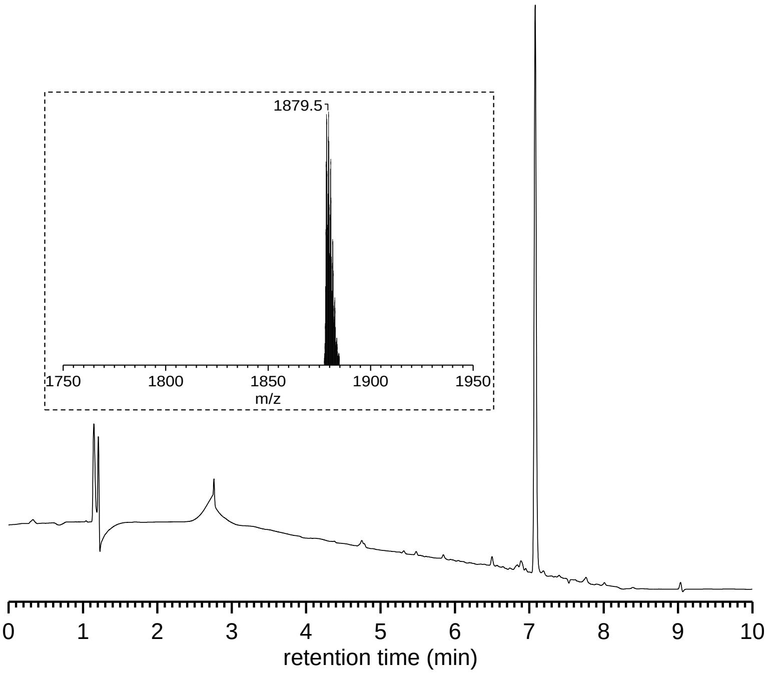

Supplementary Figure 100 | LC-ESI-MS analysis of **QR9**. Insert: ESI-MS-spectrum of dominant species (positive mode).

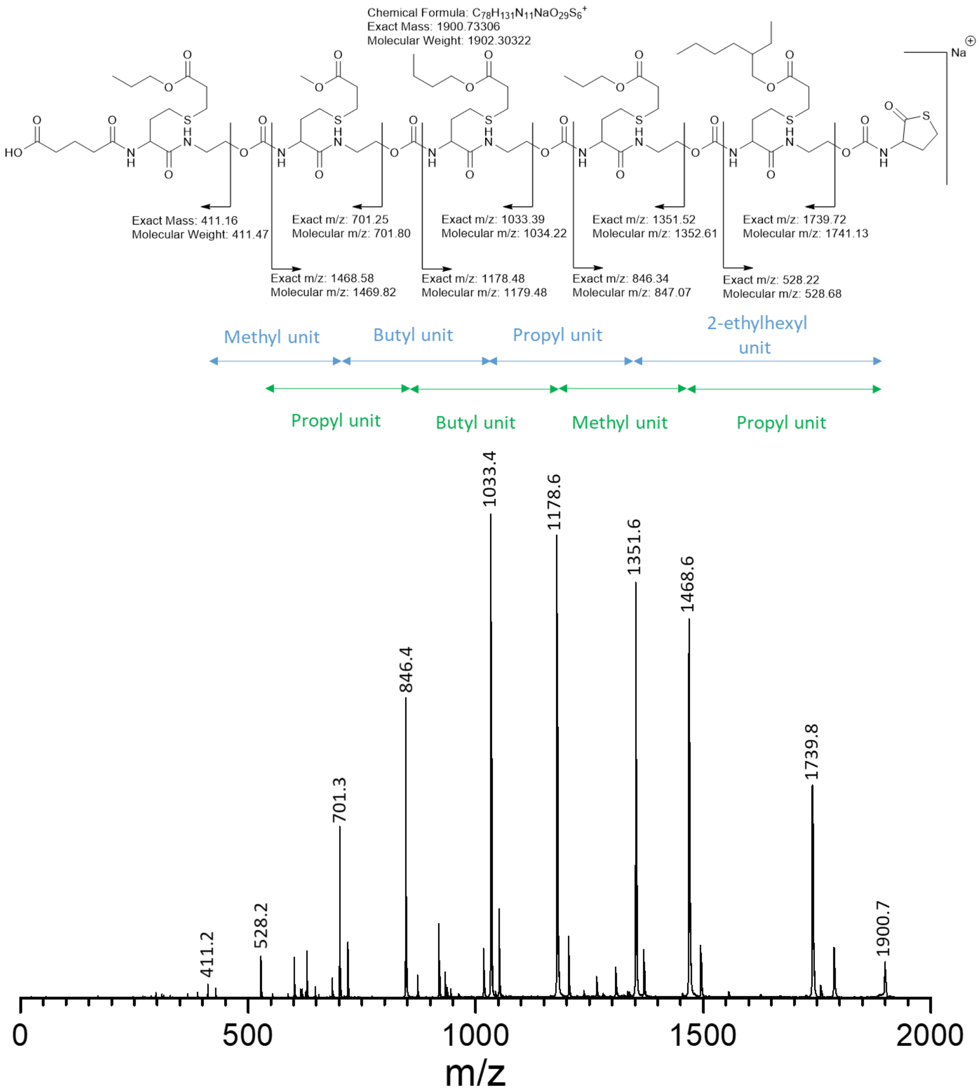

Supplementary Figure 101 | MALDI-MS/MS spectrum with peak assignment of **QR9**.

*Characterization of **QR10** using mass spectrometry (Supplementary Figure 102) and MALDI-MS/MS analysis (Supplementary Figure 103).*

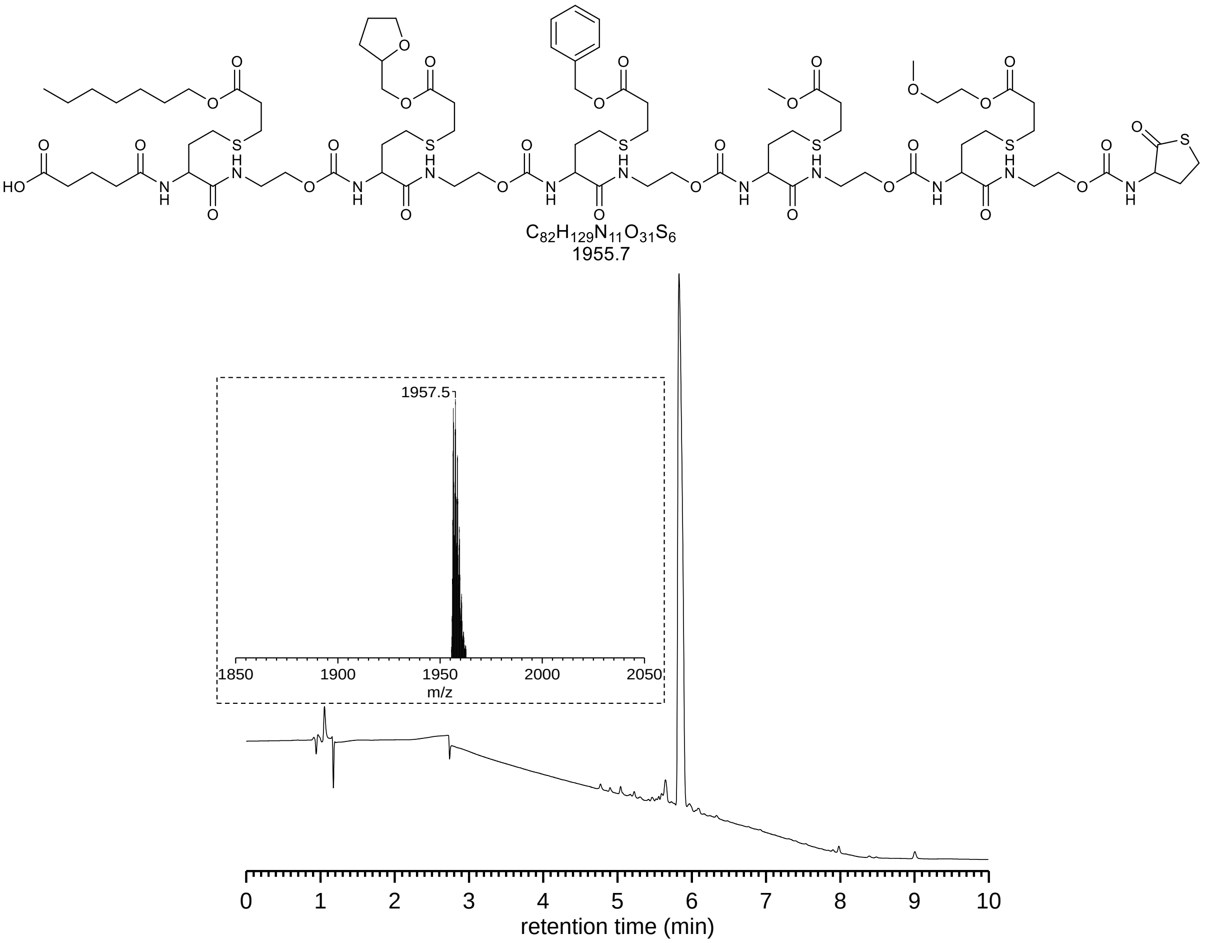

Supplementary Figure 102 | LC-ESI-MS analysis of **QR10**. Insert: ESI-MS-spectrum of dominant species (positive mode).

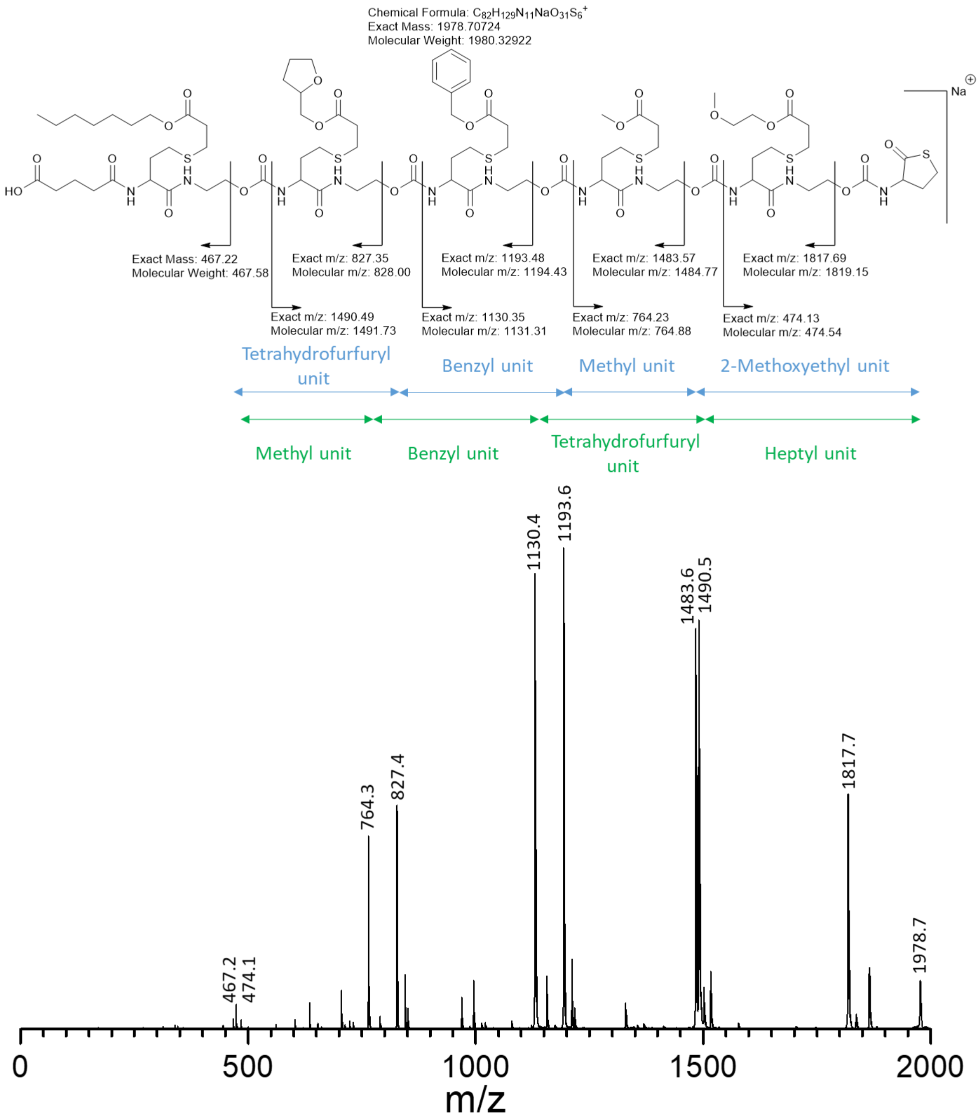

Supplementary Figure 103 | MALDI-MS/MS spectrum with peak assignment of **QR10**.

*Characterization of **QR11** using mass spectrometry (Supplementary Figure 104) and MALDI-MS/MS analysis (Supplementary Figure 105).*

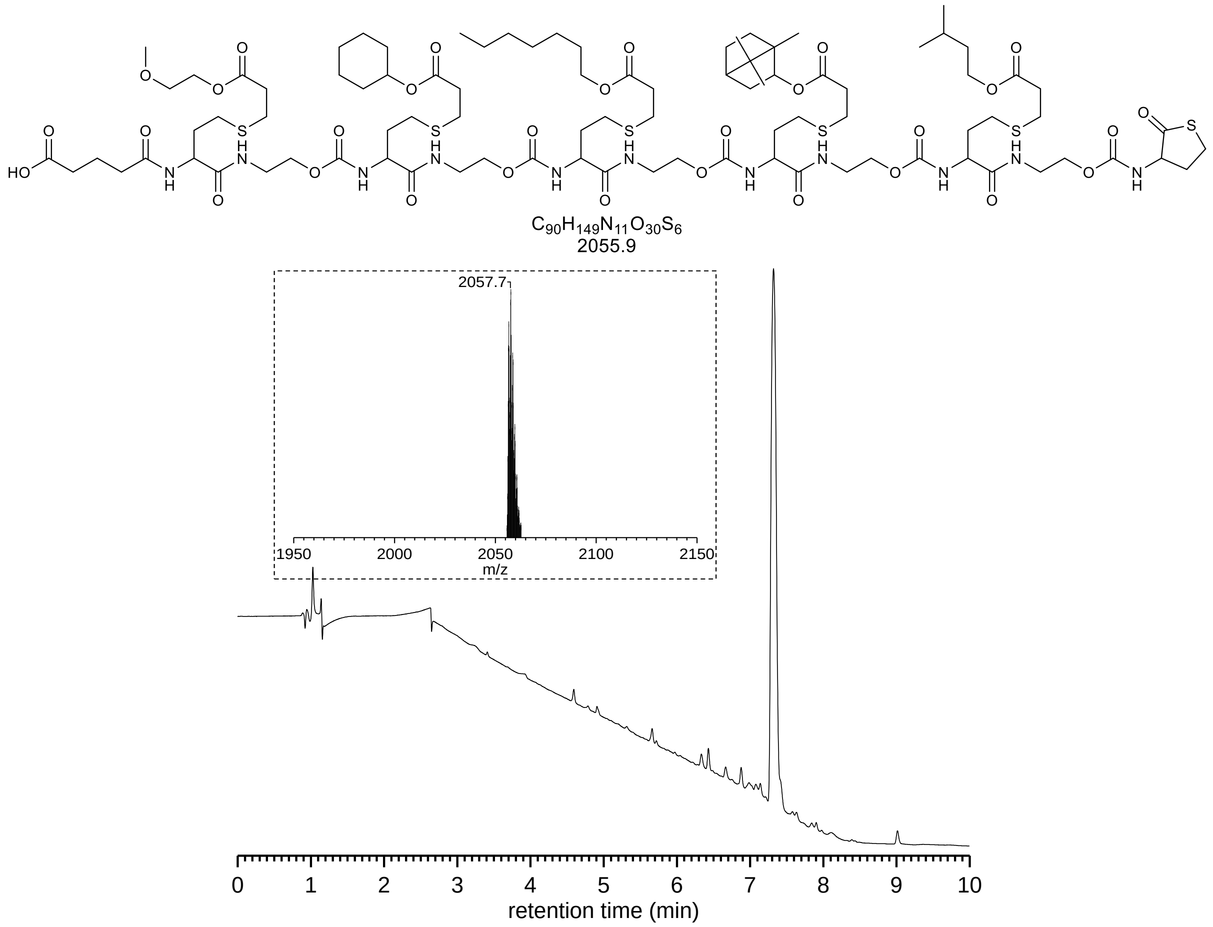

Supplementary Figure 104 | LC-ESI-MS analysis of **QR11**. Insert: ESI-MS-spectrum of dominant species (positive mode).

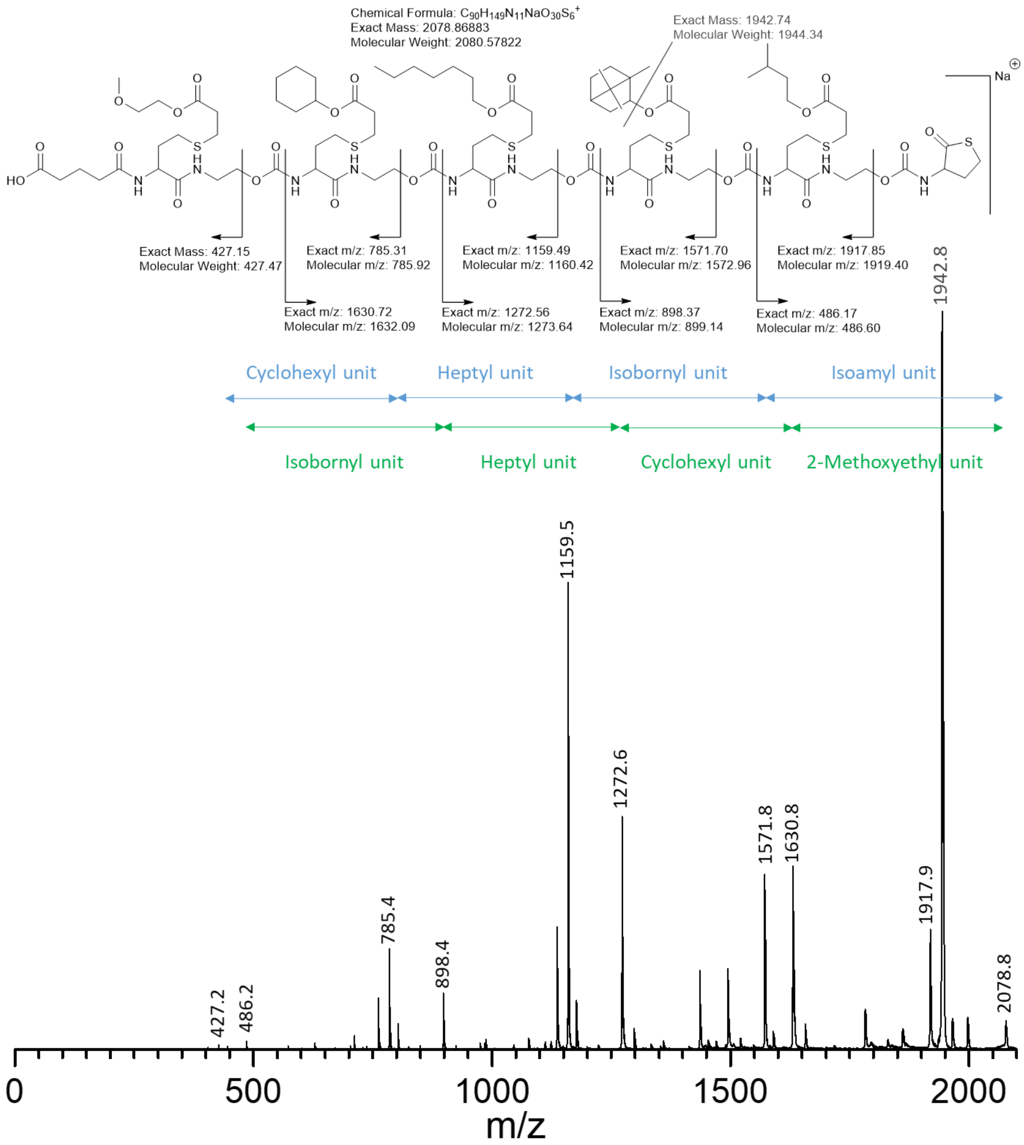

Supplementary Figure 105 | MALDI-MS/MS spectrum with peak assignment of **QR11**.

*Characterization of **QR12** using mass spectrometry (Supplementary Figure 106) and MALDI-MS/MS analysis (Supplementary Figure 107).*

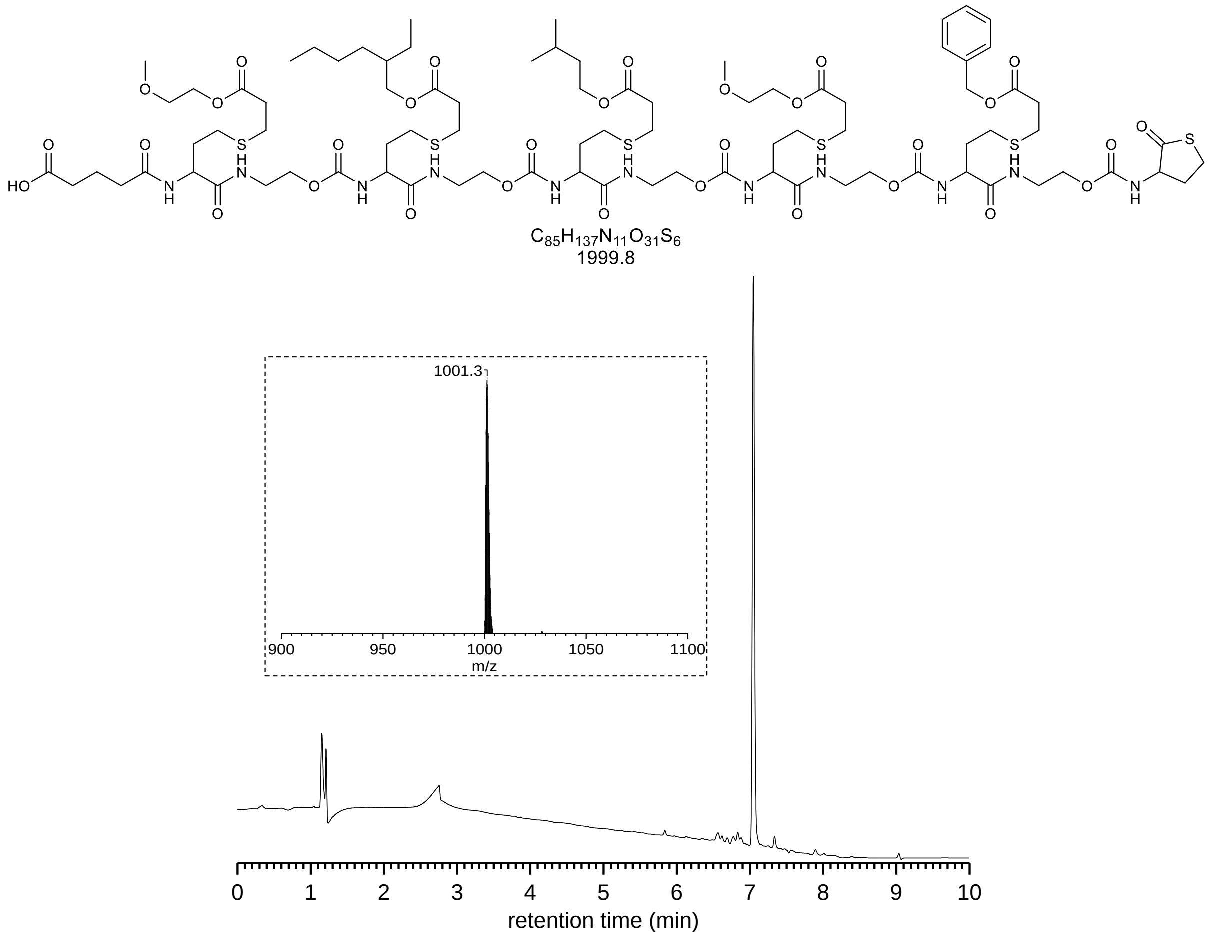

Supplementary Figure 106 | LC-ESI-MS analysis of **QR12**. Insert: ESI-MS-spectrum of dominant species (positive mode).

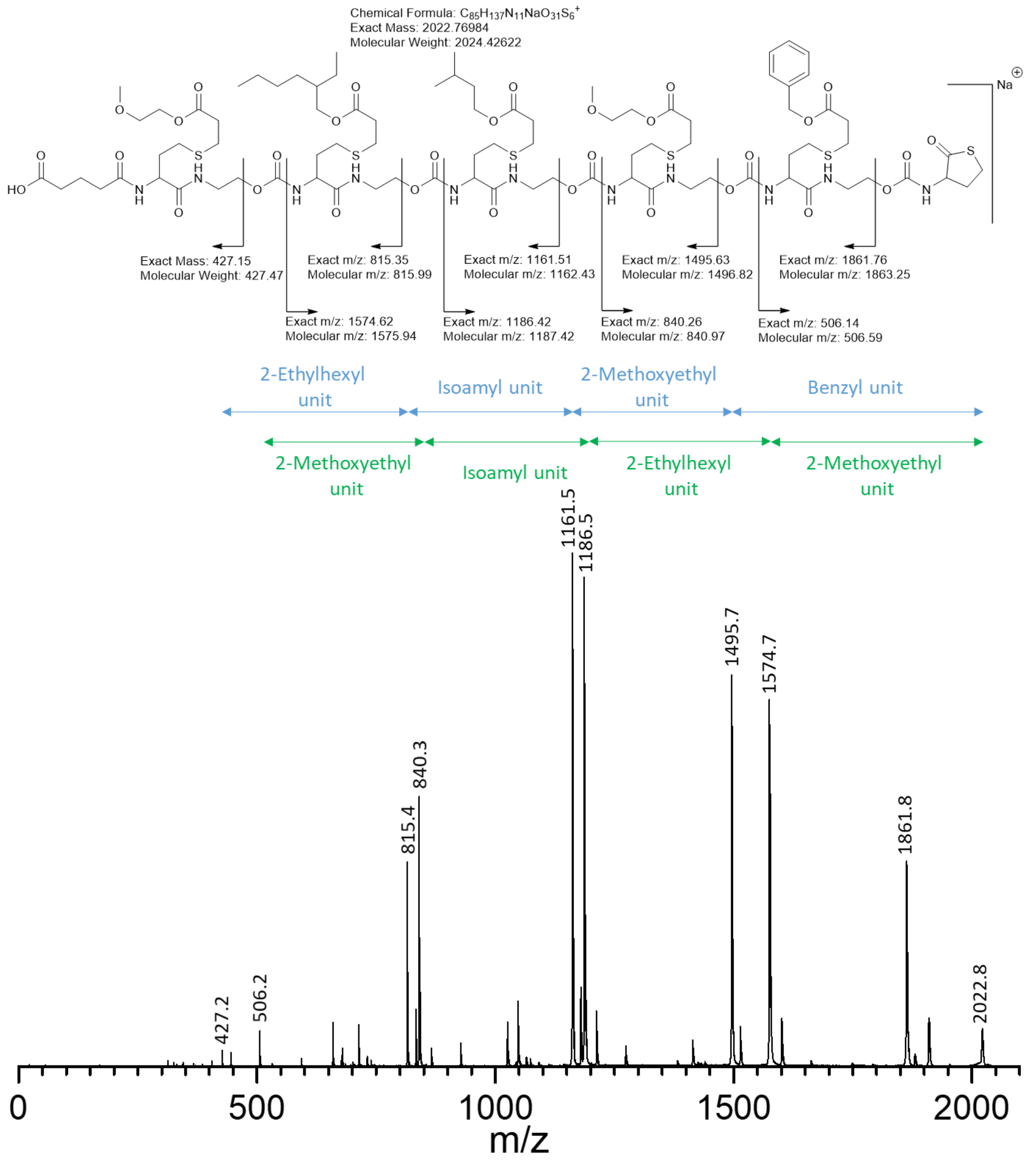

Supplementary Figure 107 | MALDI-MS/MS spectrum with peak assignment of **QR12**.

*Characterization of **QR13** using mass spectrometry (Supplementary Figure 108) and MALDI-MS/MS analysis (Supplementary Figure 109).*

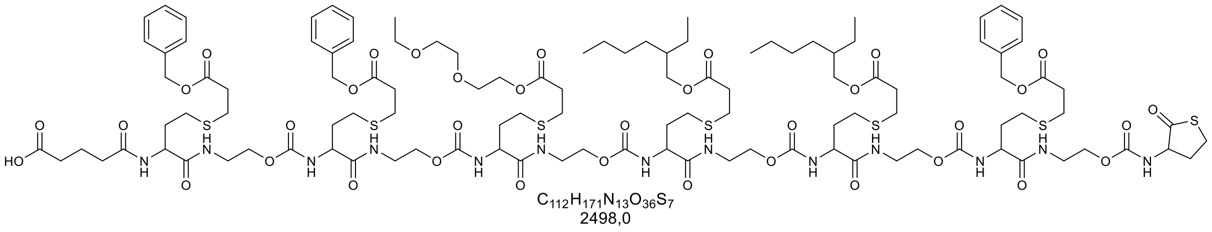

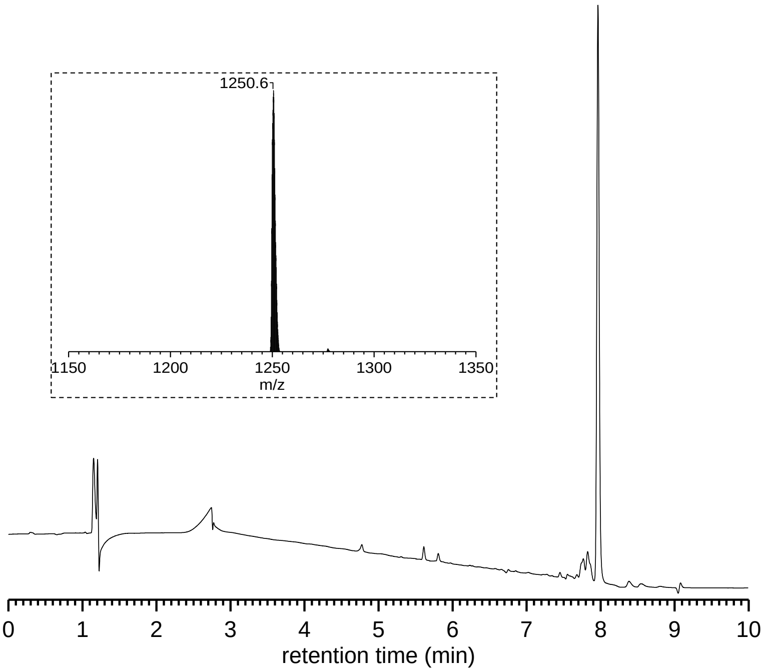

Supplementary Figure 108 | LC-ESI-MS analysis of **QR13**. Insert: ESI-MS-spectrum of dominant species (positive mode).

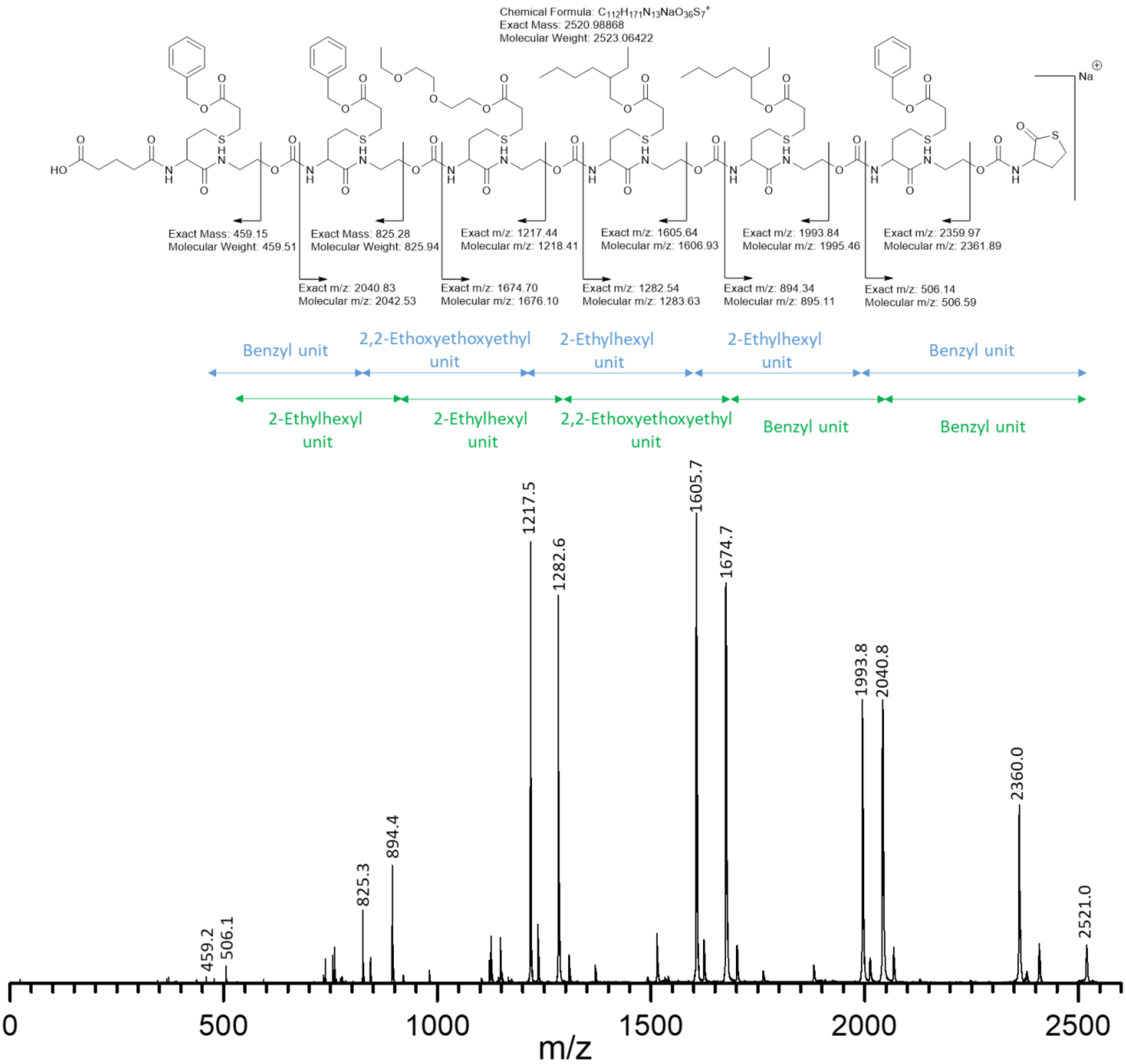

Supplementary Figure 109 | MALDI-MS/MS spectrum with peak assignment of **QR13**.

*Characterization of **QR14** using mass spectrometry (Supplementary Figure 110) and MALDI-MS/MS analysis (Supplementary Figure 111).*

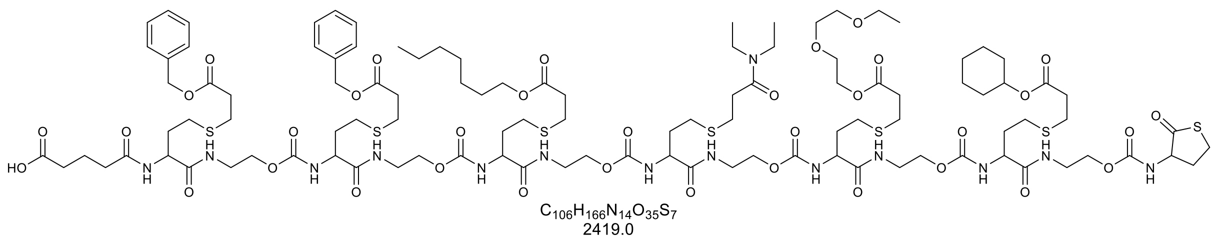

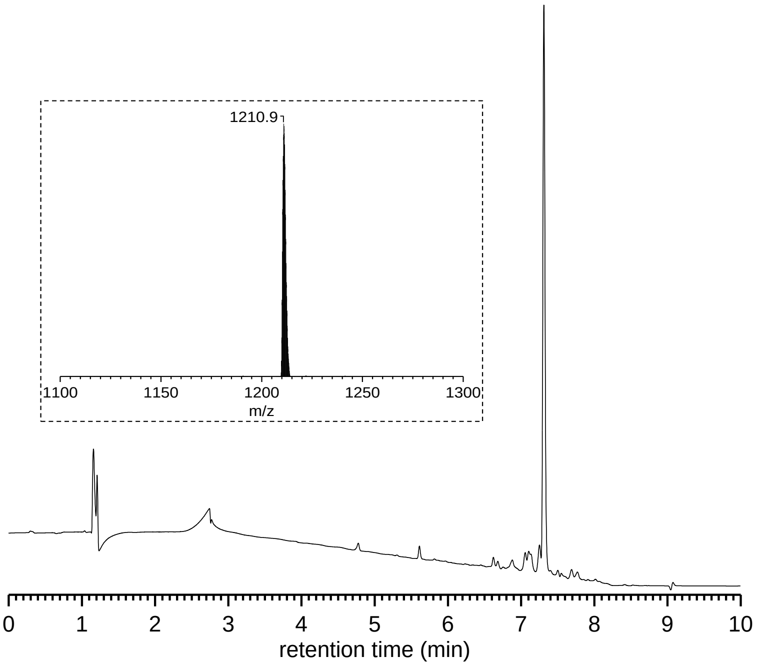

Supplementary Figure 110 | LC-ESI-MS analysis of **QR14**. Insert: ESI-MS-spectrum of dominant species (positive mode).

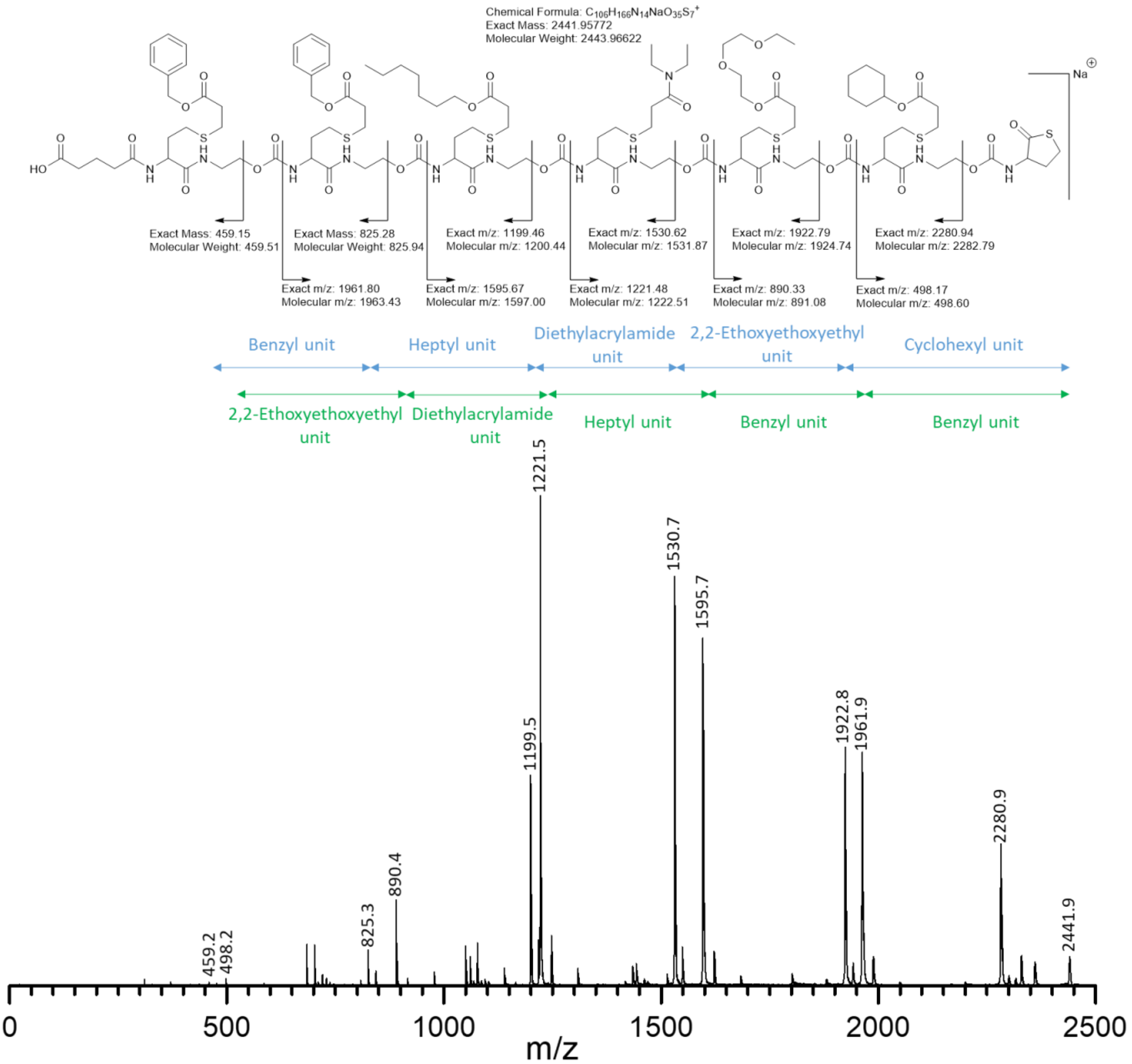

Supplementary Figure 111 | MALDI-MS/MS spectrum with peak assignment of **QR14**.

*Characterization of **QR15** using mass spectrometry (Supplementary Figure 112) and MALDI-MS/MS analysis (Supplementary Figure 113).*

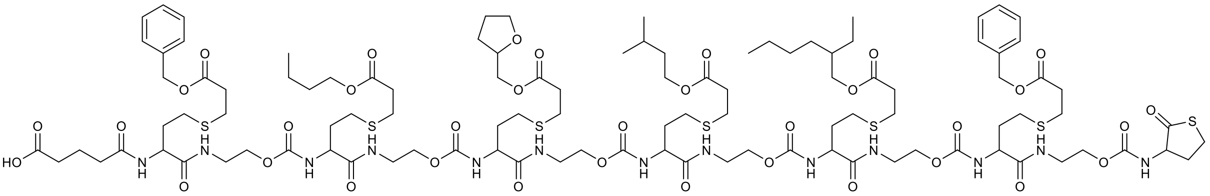

$C_{105}H_{163}N_{13}O_{35}S_7$
2389.9

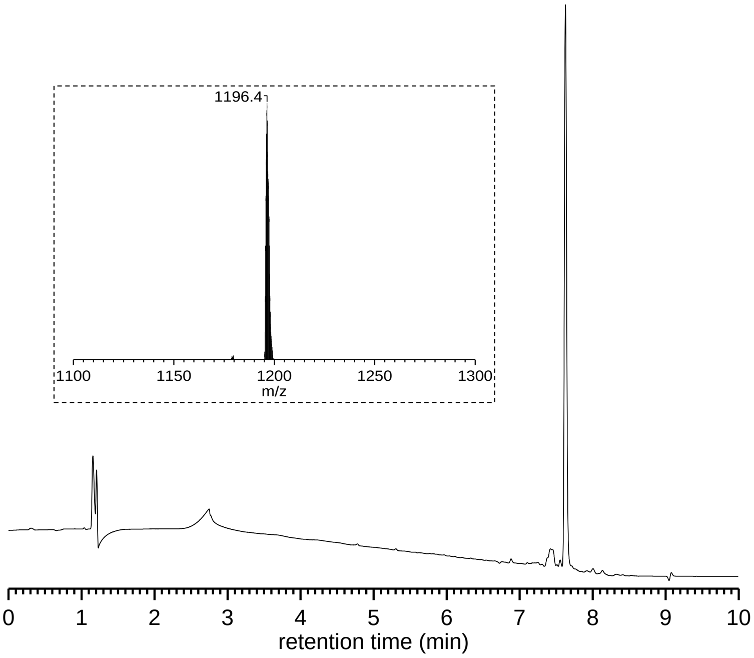

Supplementary Figure 112 | LC-ESI-MS analysis of **QR15**. Insert: ESI-MS-spectrum of dominant species (positive mode).

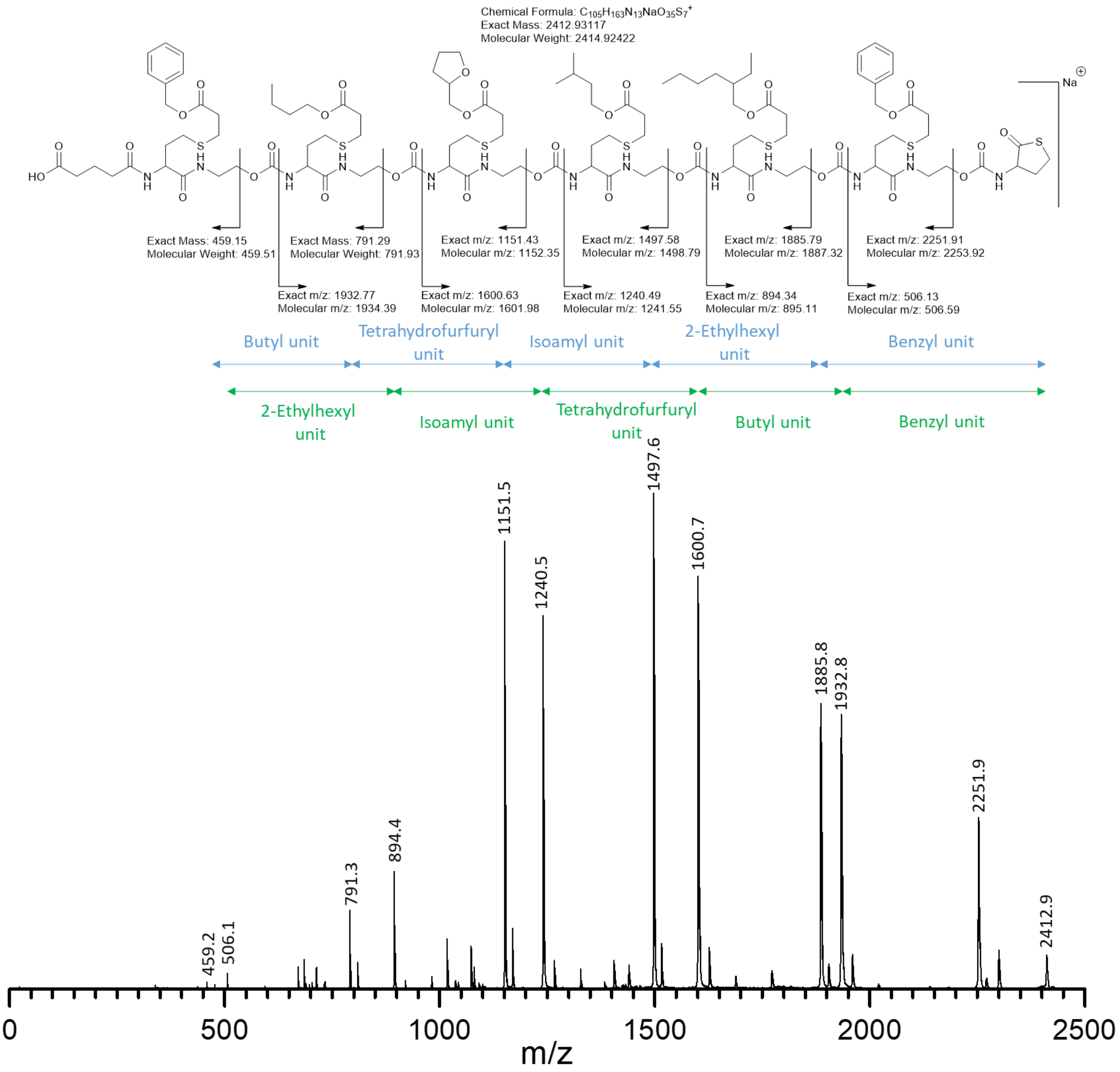

Supplementary Figure 113 | MALDI-MS/MS spectrum with peak assignment of **QR15**.

Characterization of ***QR16*** *using mass spectrometry (Supplementary Figure 114) and MALDI-MS/MS analysis (Supplementary Figure 115).*

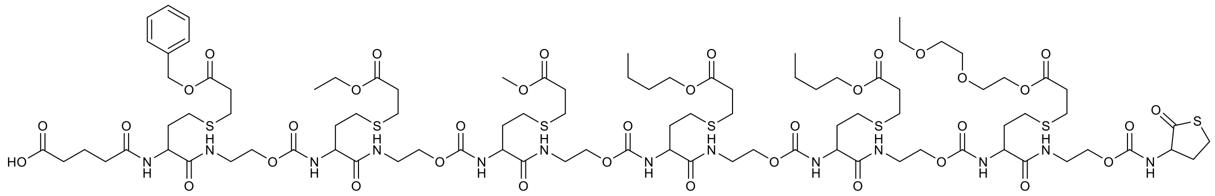

$C_{93}H_{149}N_{13}O_{36}S_7$
2247.8

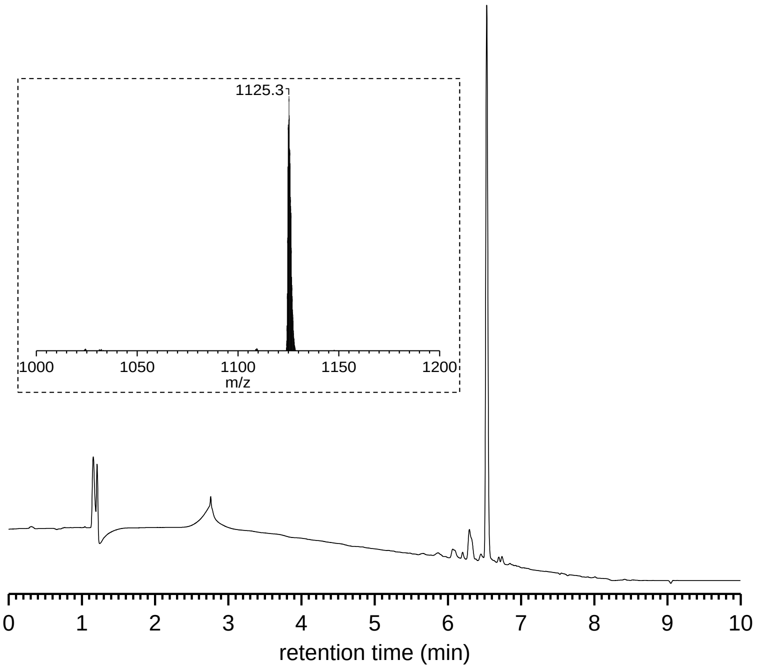

Supplementary Figure 114 | LC-ESI-MS analysis of **QR16**. Insert: ESI-MS-spectrum of dominant species (positive mode).

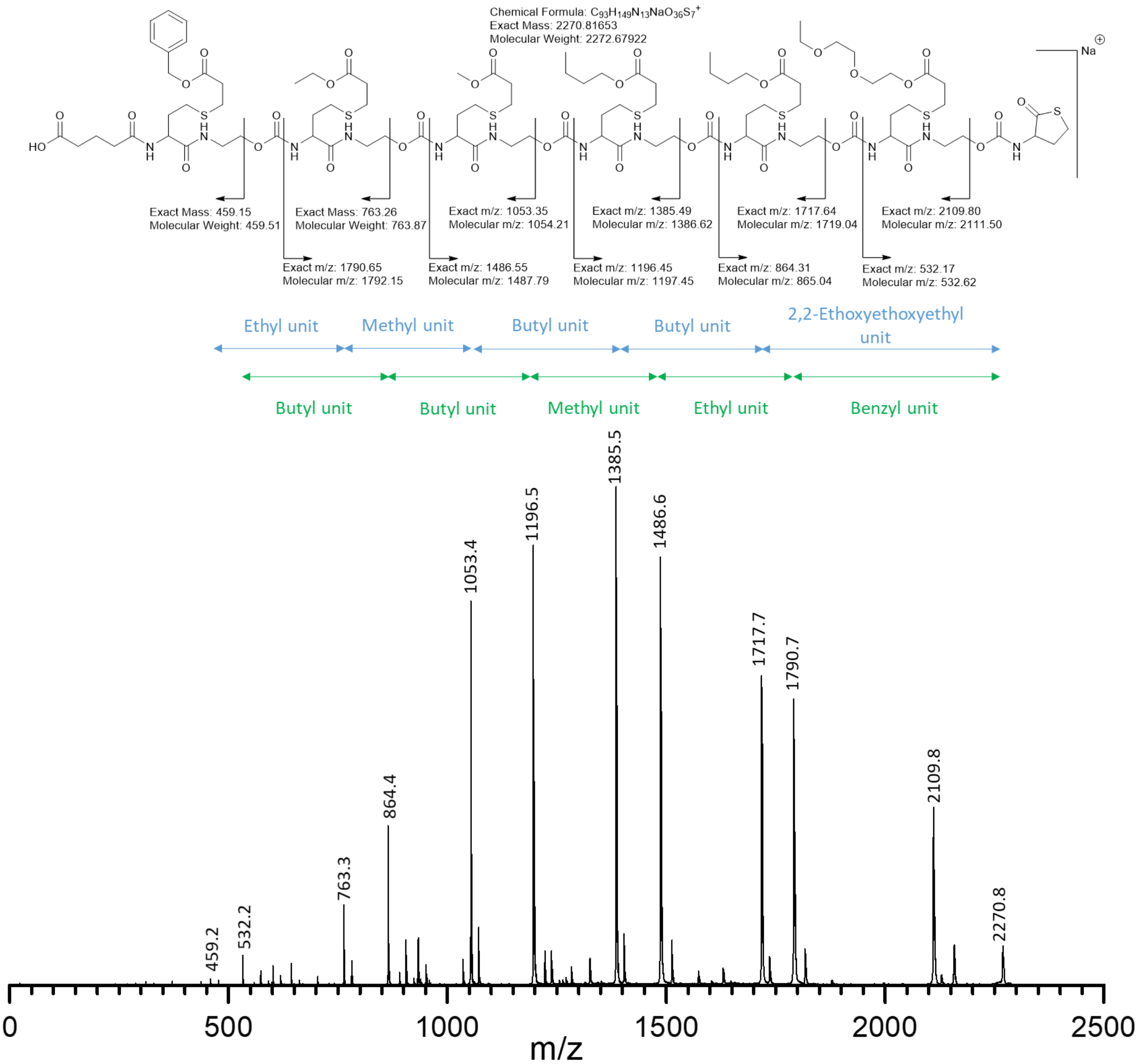

Supplementary Figure 115 | MALDI-MS/MS spectrum with peak assignment of **QR16**.

*Characterization of **QR17** using mass spectrometry (Supplementary Figure 116) and MALDI-MS/MS analysis (Supplementary Figure 117).*

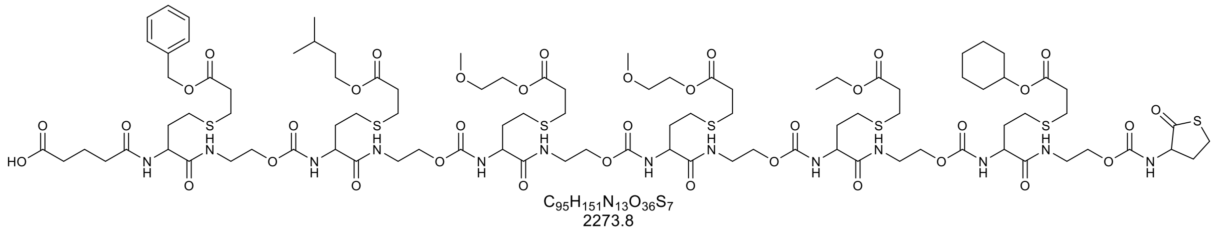

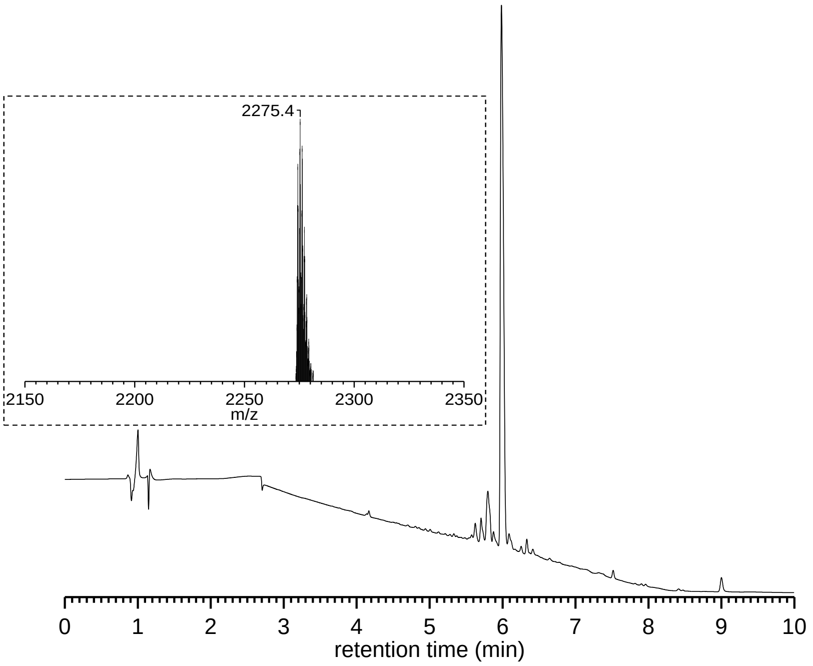

Supplementary Figure 116 | LC-ESI-MS analysis of **QR17**. Insert: ESI-MS-spectrum of dominant species (positive mode).

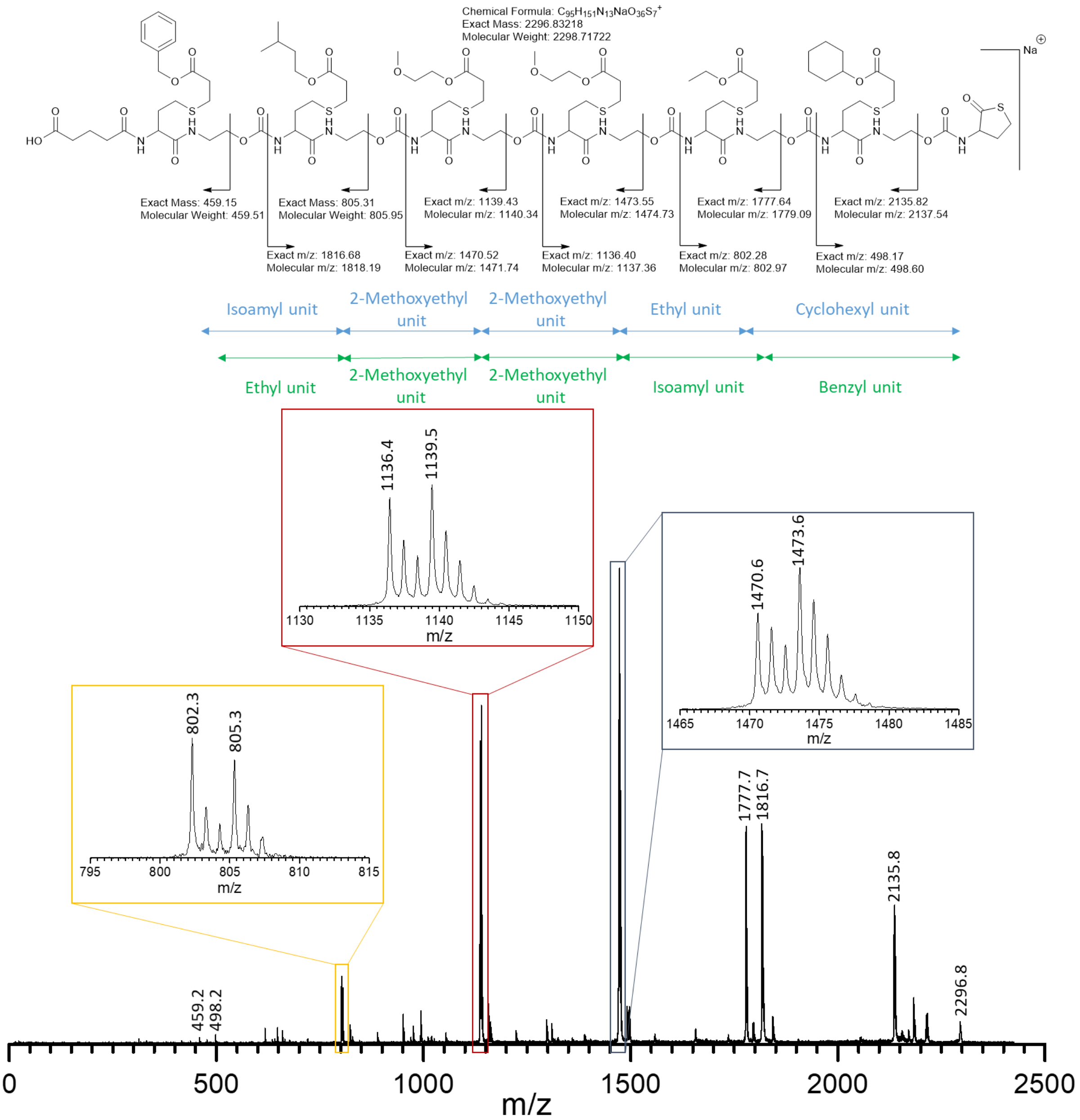

Supplementary Figure 117 | MALDI-MS/MS spectrum with peak assignment of **QR17**.

*Characterization of **QR18** using mass spectrometry (Supplementary Figure 118) and MALDI-MS/MS analysis (Supplementary Figure 119).*

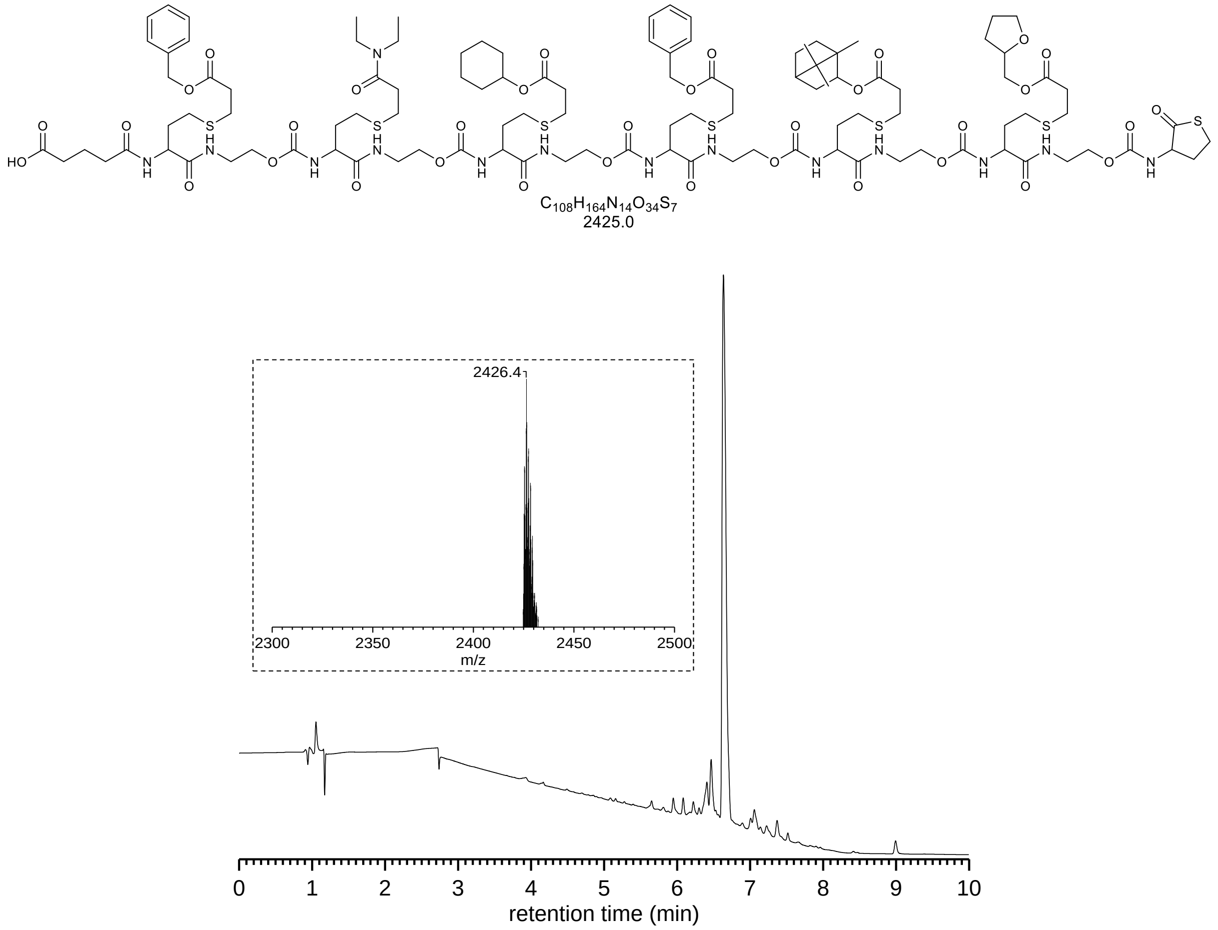

Supplementary Figure 118 | LC-ESI-MS analysis of **QR18**. Insert: ESI-MS-spectrum of dominant species (positive mode).

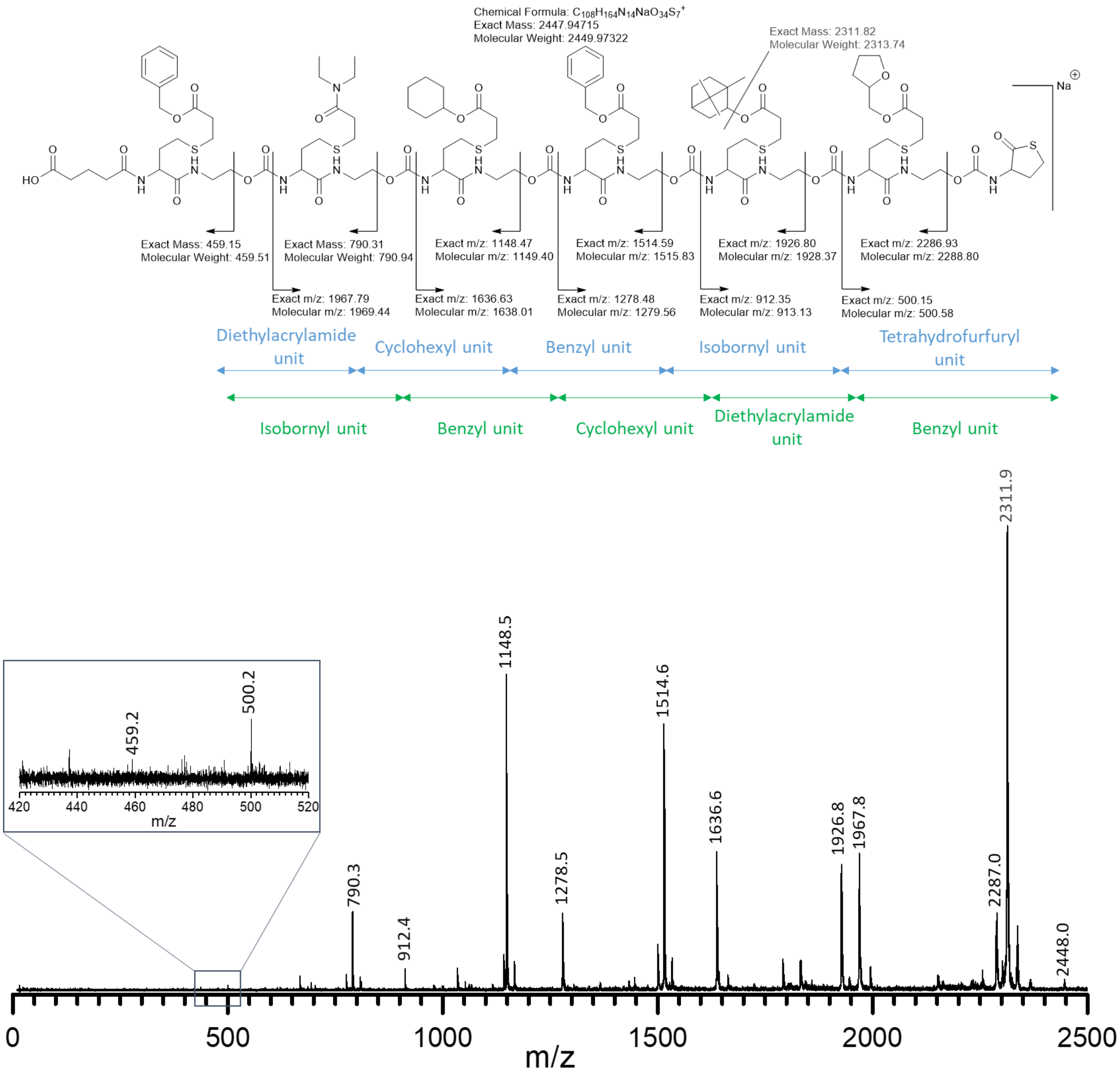

Supplementary Figure 119 | MALDI-MS/MS spectrum with peak assignment of **QR18**.

*Characterization of **QR19** using mass spectrometry (Supplementary Figure 120) and MALDI-MS/MS analysis (Supplementary Figure 121).*

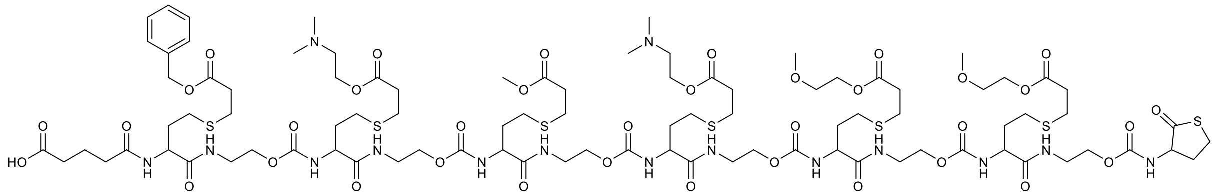

$C_{91}H_{147}N_{15}O_{36}S_7$
2249.8

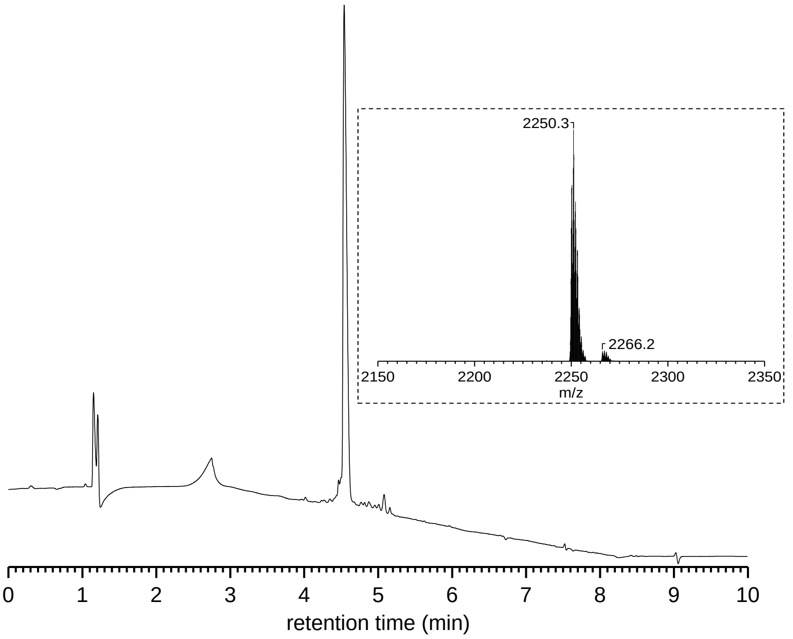

Supplementary Figure 120 | LC-ESI-MS analysis of **QR19**. Insert: ESI-MS-spectrum of dominant species (positive mode).

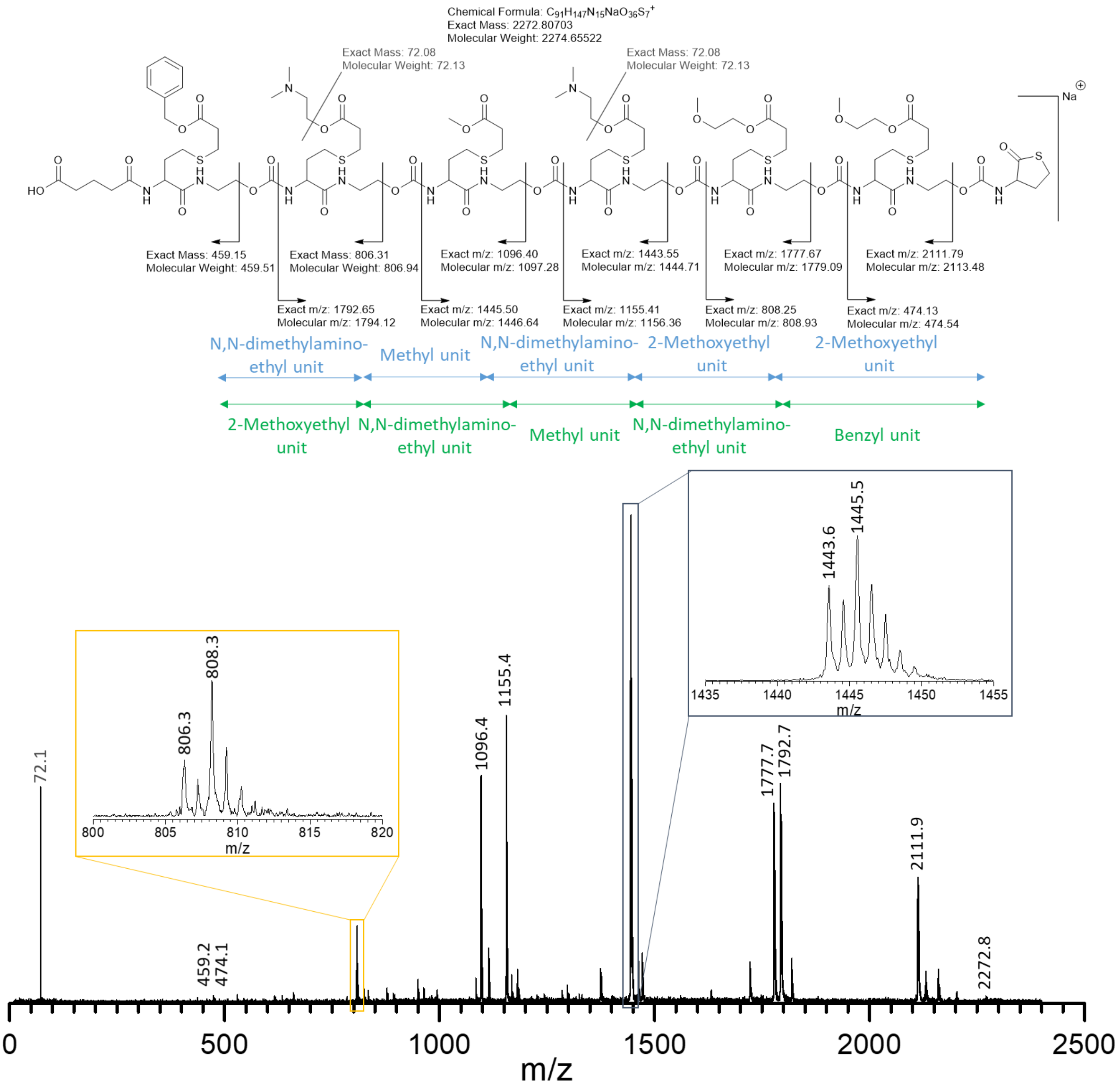

Supplementary Figure 121 | MALDI-MS/MS spectrum with peak assignment of **QR19**.

*Characterization of **QR20** using mass spectrometry (Supplementary Figure 122) and MALDI-MS/MS analysis (Supplementary Figure 123).*

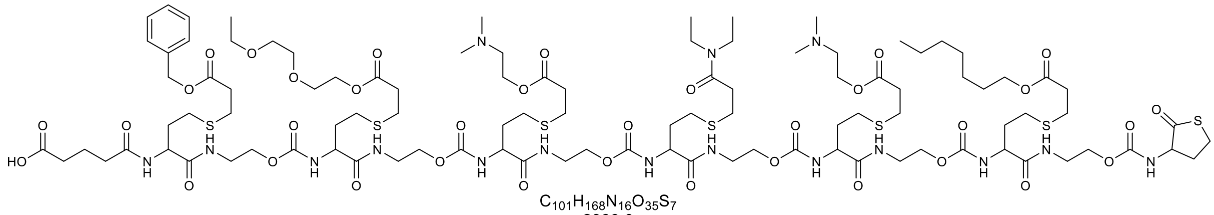

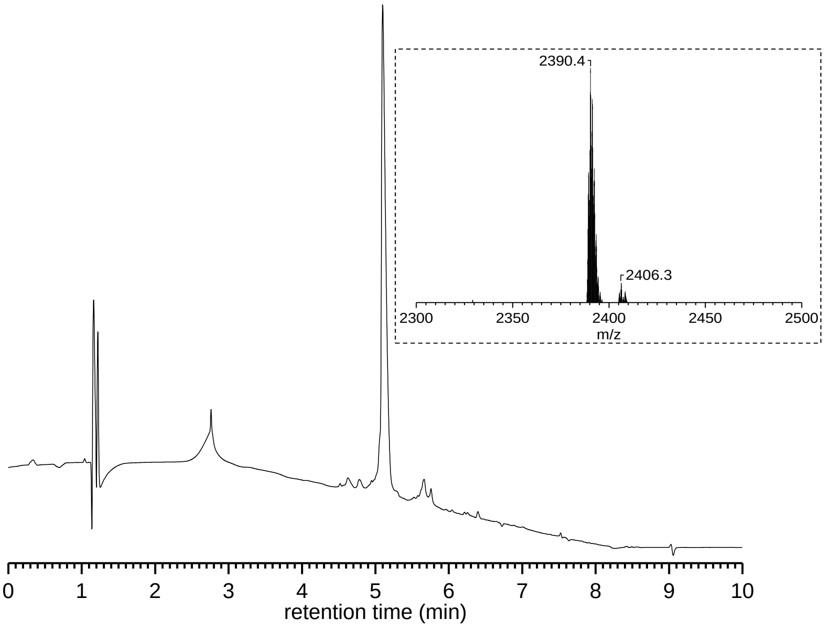

Supplementary Figure 122 | LC-ESI-MS analysis of **QR20**. Insert: ESI-MS-spectrum of dominant species (positive mode).

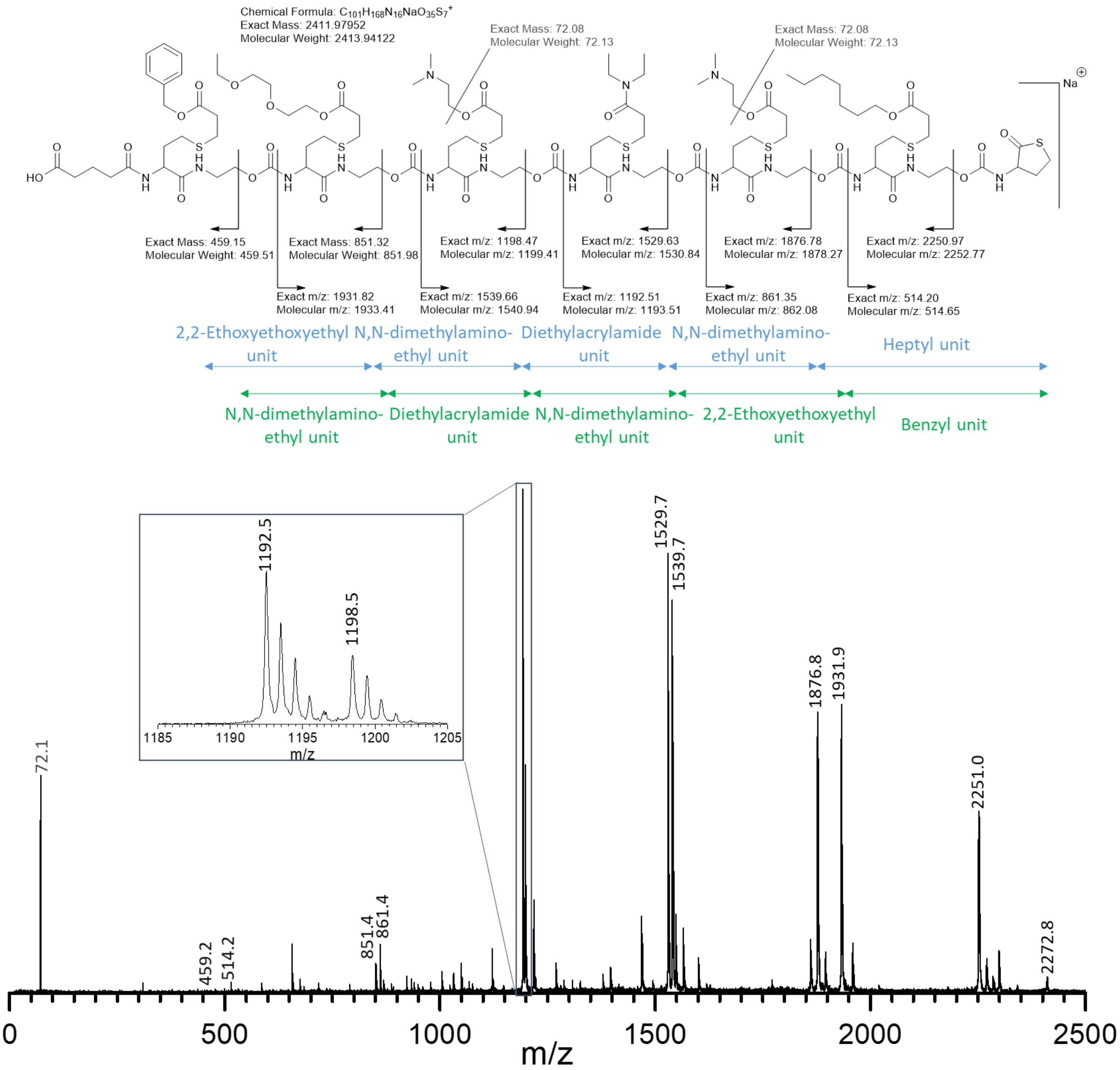

Supplementary Figure 123 | MALDI-MS/MS spectrum with peak assignment of **QR20**.

*Characterization of **QR21** using mass spectrometry (Supplementary Figure 124) and MALDI-MS/MS analysis (Supplementary Figure 125).*

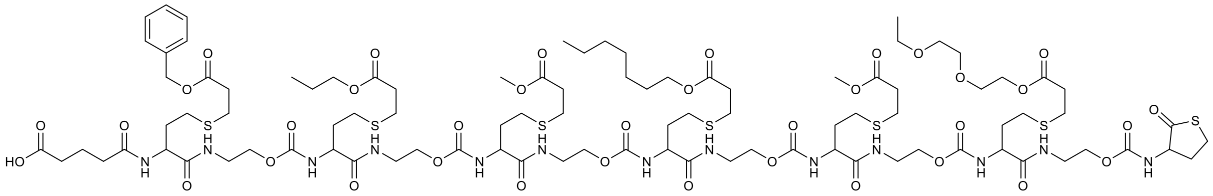

$C_{94}H_{151}N_{13}O_{36}S_7$
2261.8

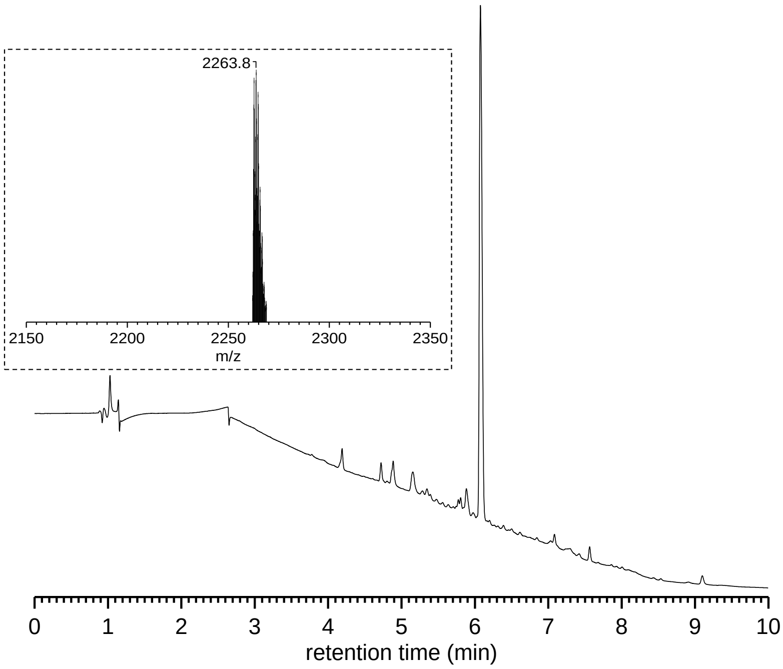

Supplementary Figure 124 | LC-ESI-MS analysis of **QR21**. Insert: ESI-MS-spectrum of dominant species (positive mode).

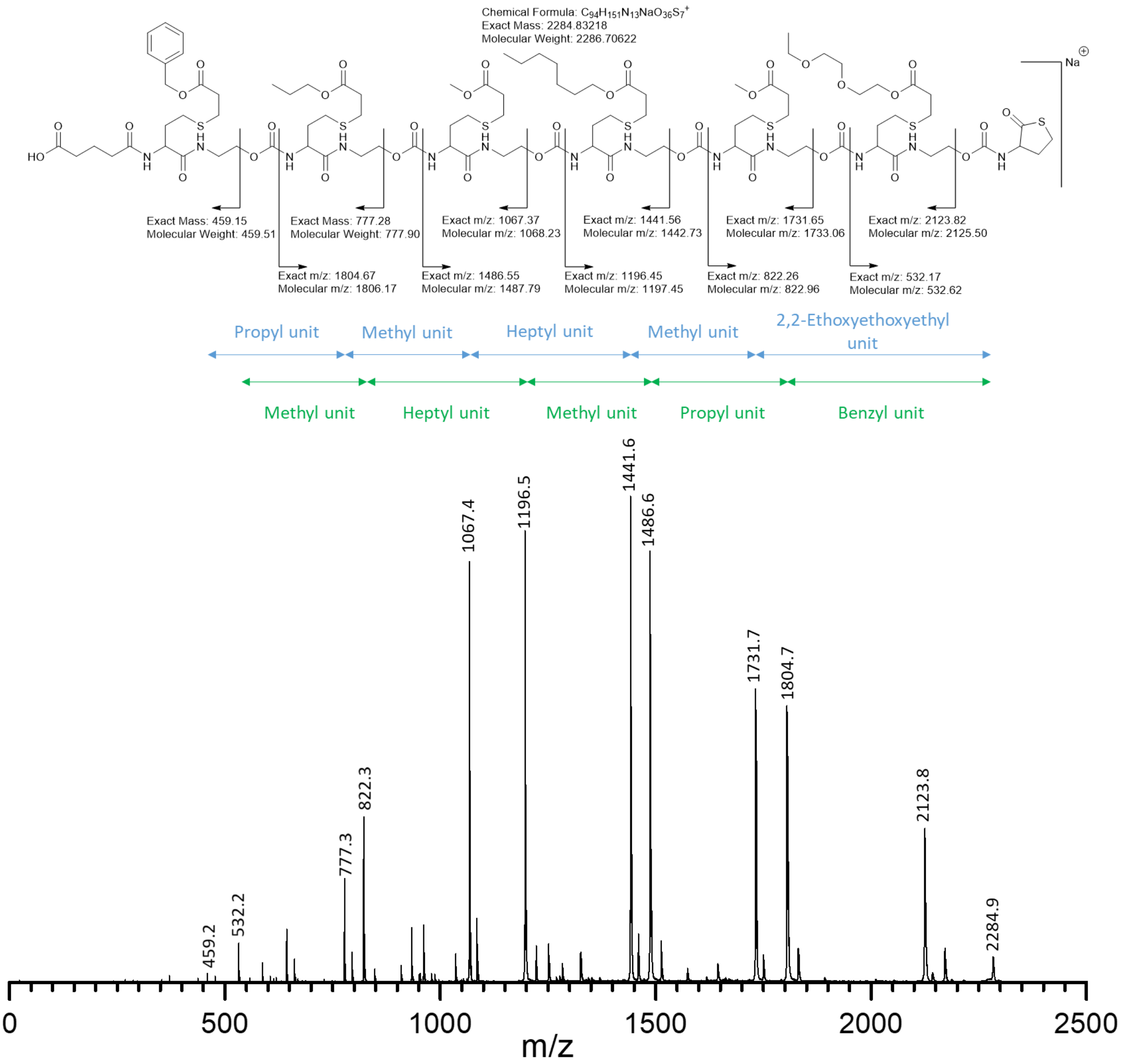

Supplementary Figure 125 | MALDI-MS/MS spectrum with peak assignment of **QR21**.

*Characterization of **QR22** using mass spectrometry (Supplementary Figure 126) and MALDI-MS/MS analysis (Supplementary Figure 127).*

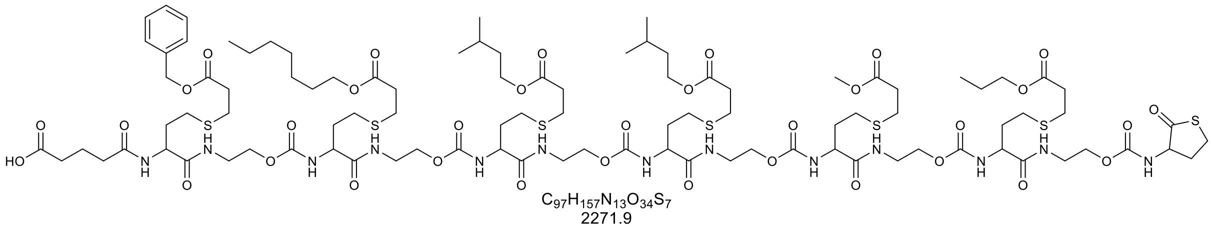

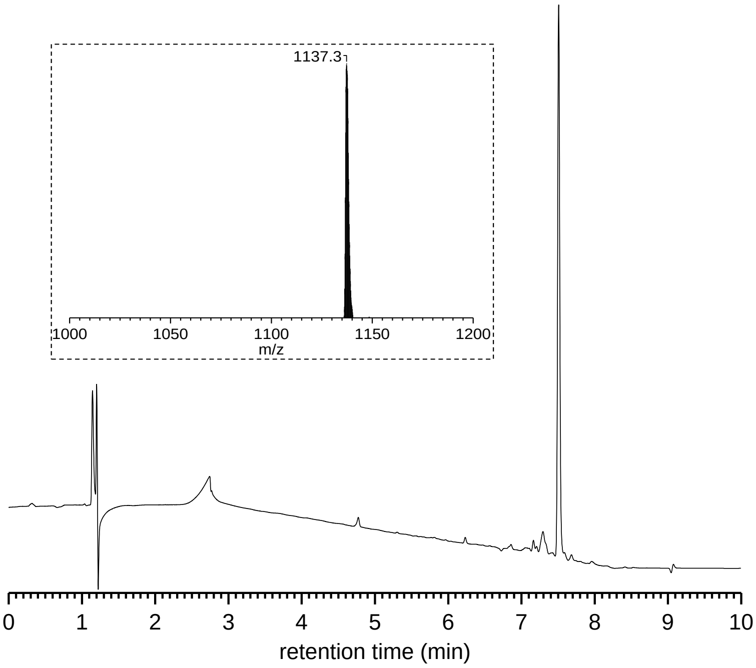

Supplementary Figure 126 | LC-ESI-MS analysis of **QR22**. Insert: ESI-MS-spectrum of dominant species (positive mode).

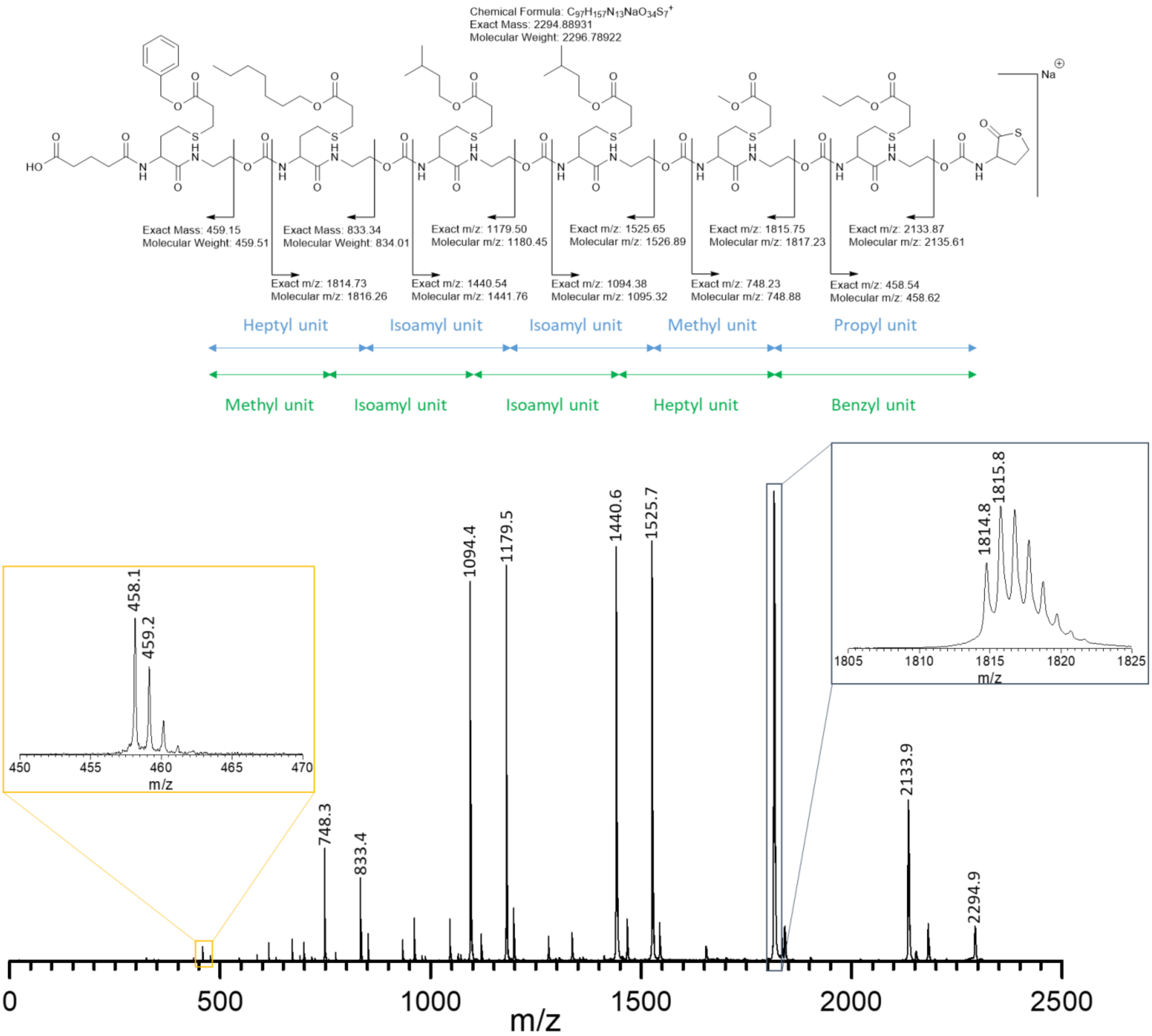

Supplementary Figure 127 | MALDI-MS/MS spectrum with peak assignment of **QR22**.

*Characterization of **QR23** using mass spectrometry (Supplementary Figure 128) and MALDI-MS/MS analysis (Supplementary Figure 129).*

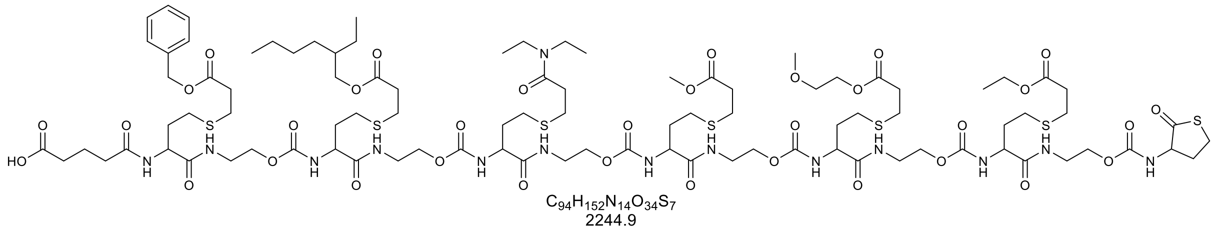

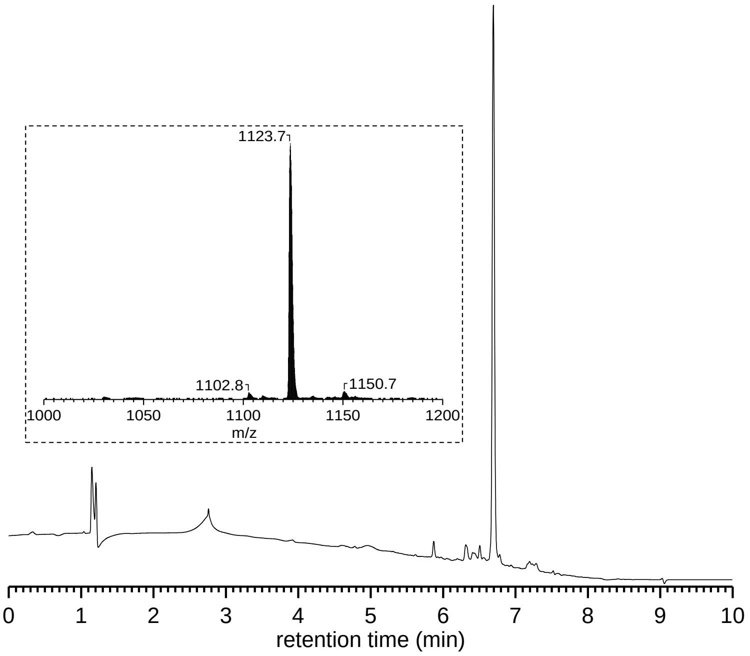

Supplementary Figure 128 | LC-ESI-MS analysis of **QR23**. Insert: ESI-MS-spectrum of dominant species (positive mode).

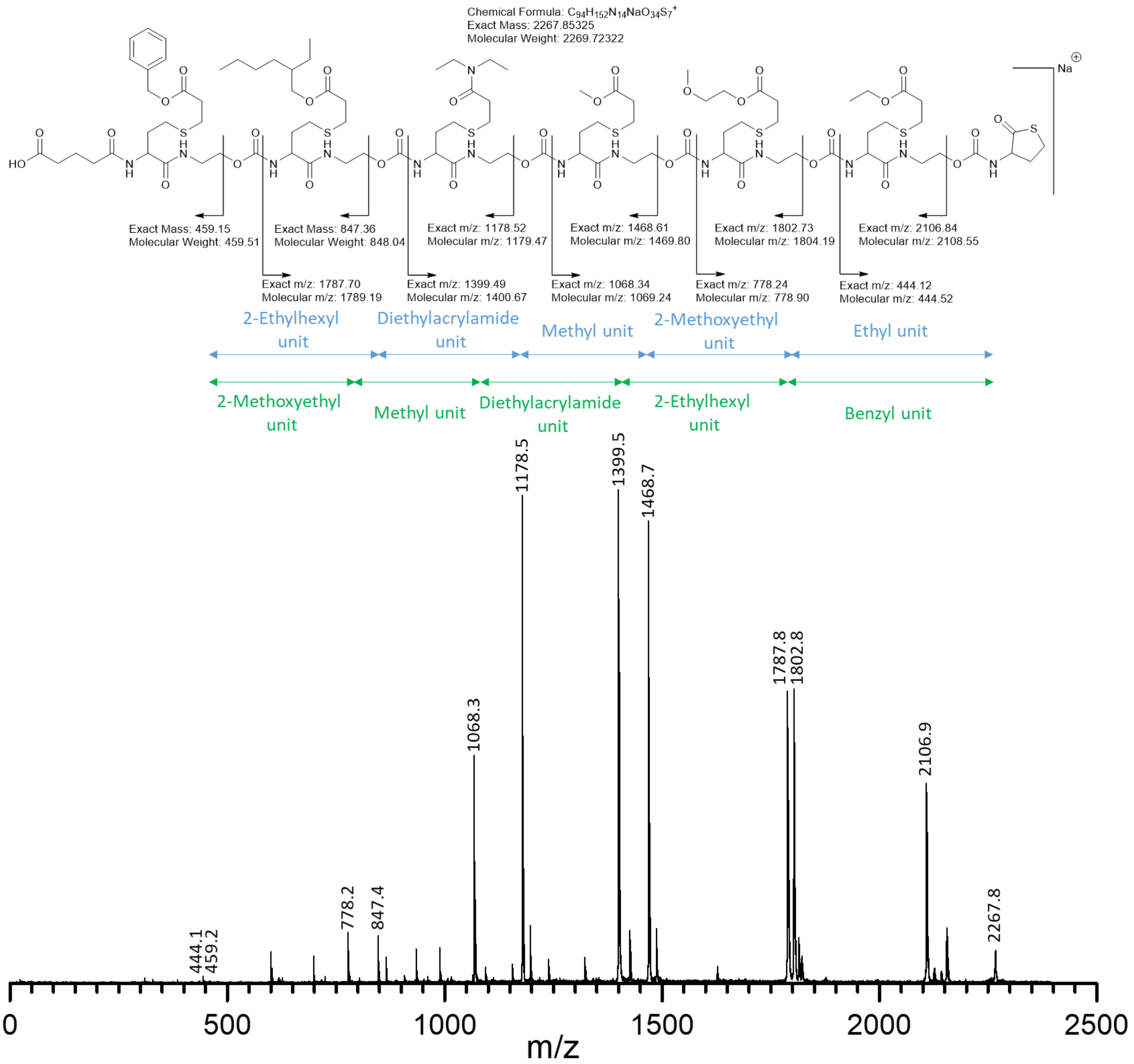

Supplementary Figure 129 | MALDI-MS/MS spectrum with peak assignment of **QR23**.

*Characterization of **QR24** using mass spectrometry (Supplementary Figure 130) and MALDI-MS/MS analysis (Supplementary Figure 131).*

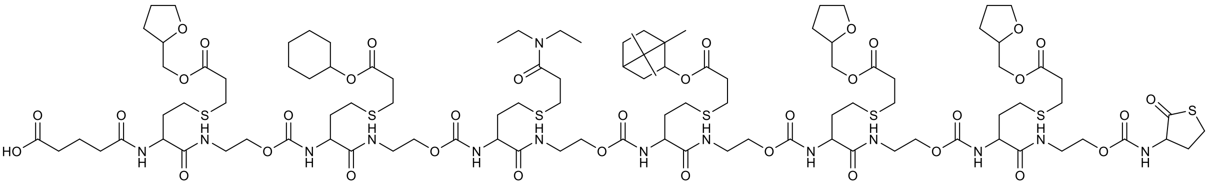

$C_{104}H_{168}N_{14}O_{36}S_7$
2413.0

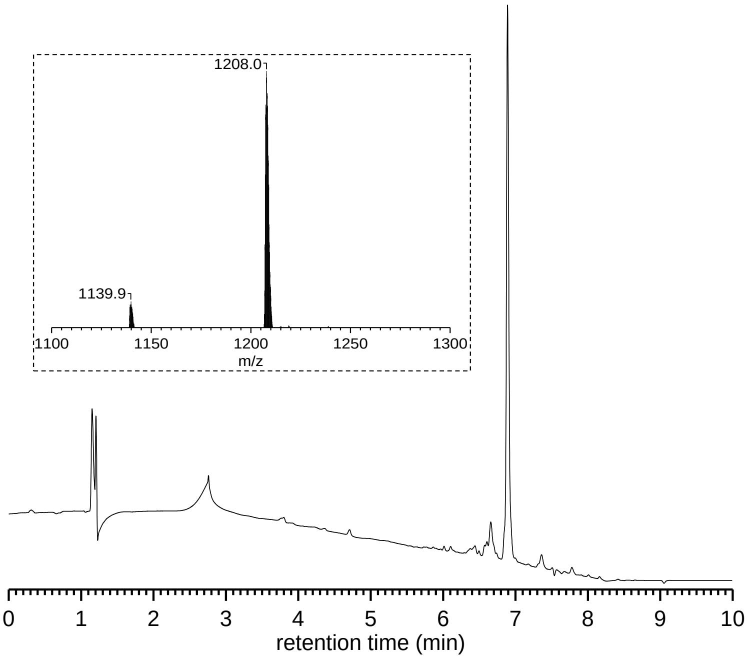

Supplementary Figure 130 | LC-ESI-MS analysis of **QR24**. Insert: ESI-MS-spectrum of dominant species (positive mode).

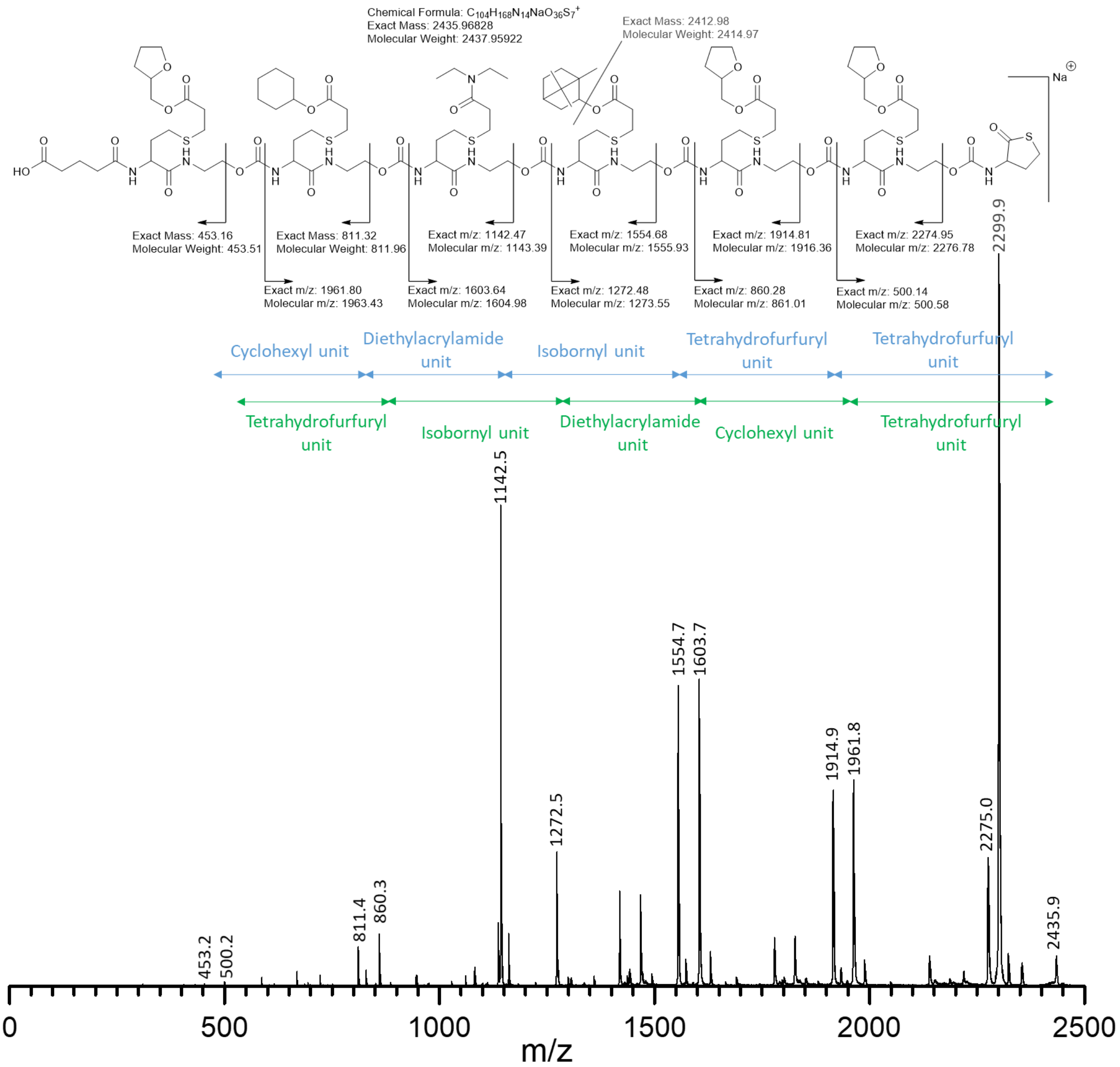

Supplementary Figure 131 | MALDI-MS/MS spectrum with peak assignment of QR24.

*Characterization of **QR25** using mass spectrometry (Supplementary Figure 132) and MALDI-MS/MS analysis (Supplementary Figure 133).*

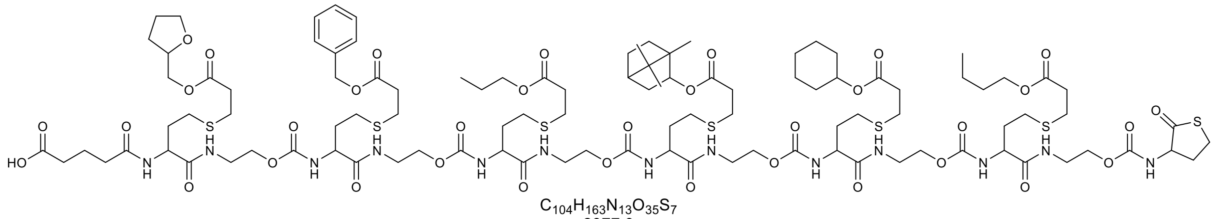

$C_{104}H_{163}N_{13}O_{35}S_7$
2377.9

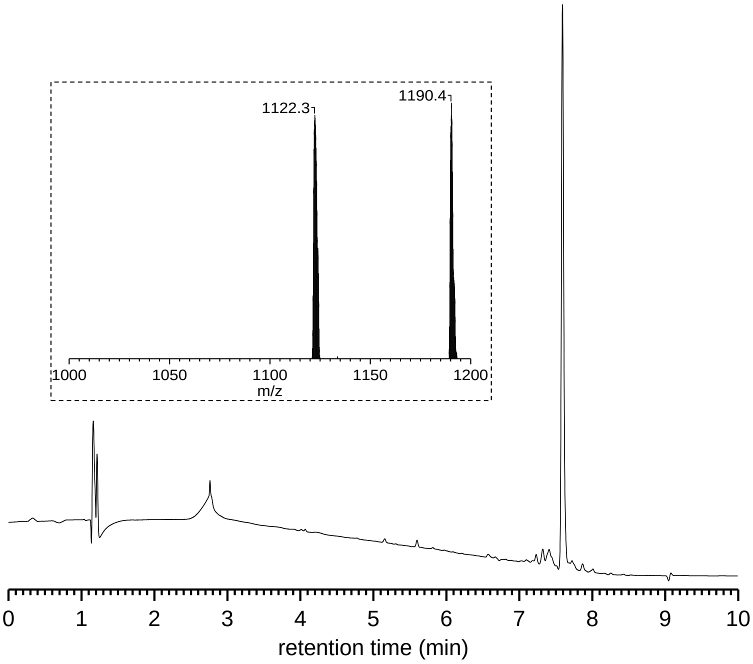

Supplementary Figure 132 | LC-ESI-MS analysis of **QR25**. Insert: ESI-MS-spectrum of dominant species (positive mode).

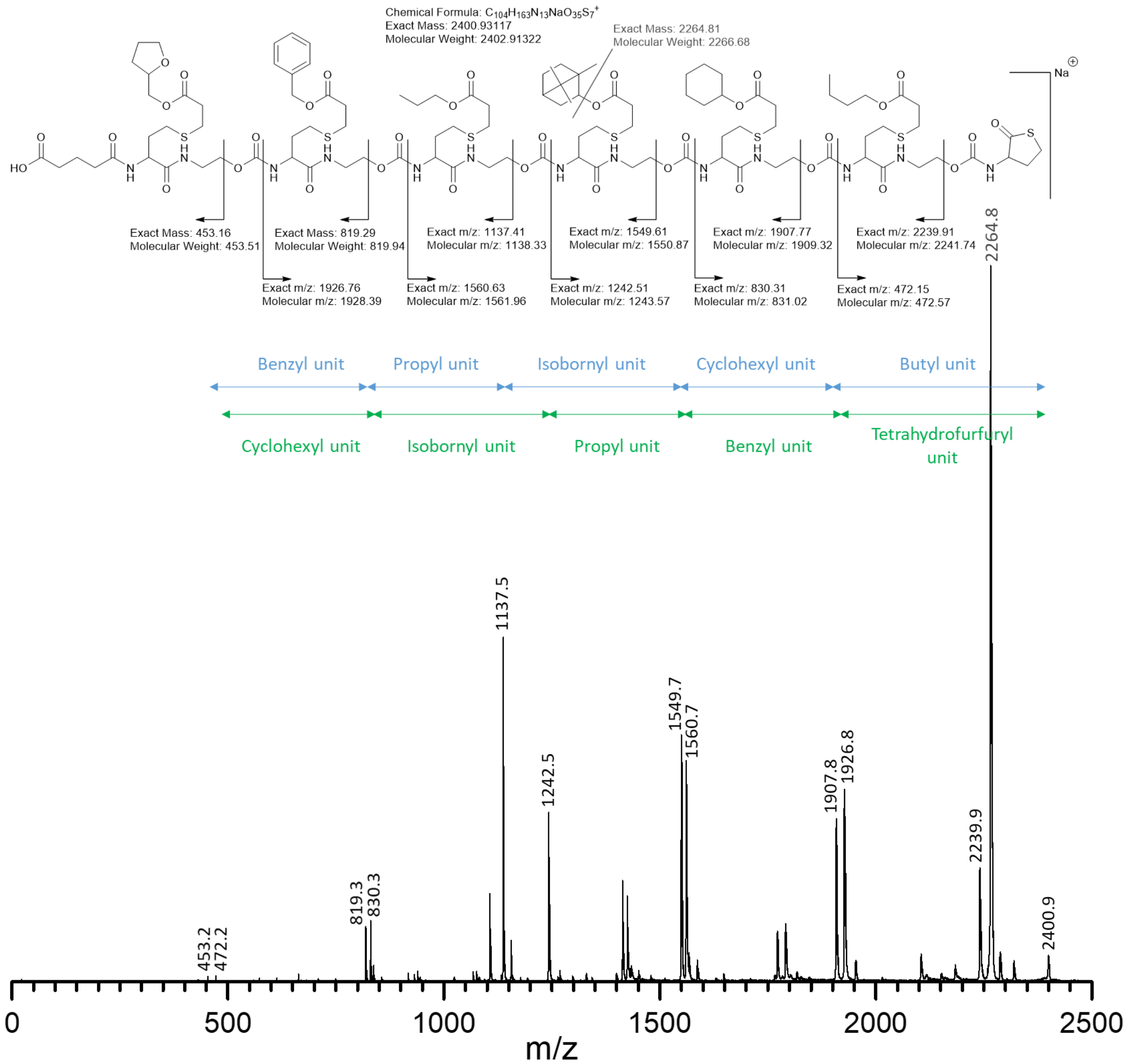

Supplementary Figure 133 | MALDI-MS/MS spectrum with peak assignment of **QR25**.

*Characterization of **QR26** using mass spectrometry (Supplementary Figure 134) and MALDI-MS/MS analysis (Supplementary Figure 135).*

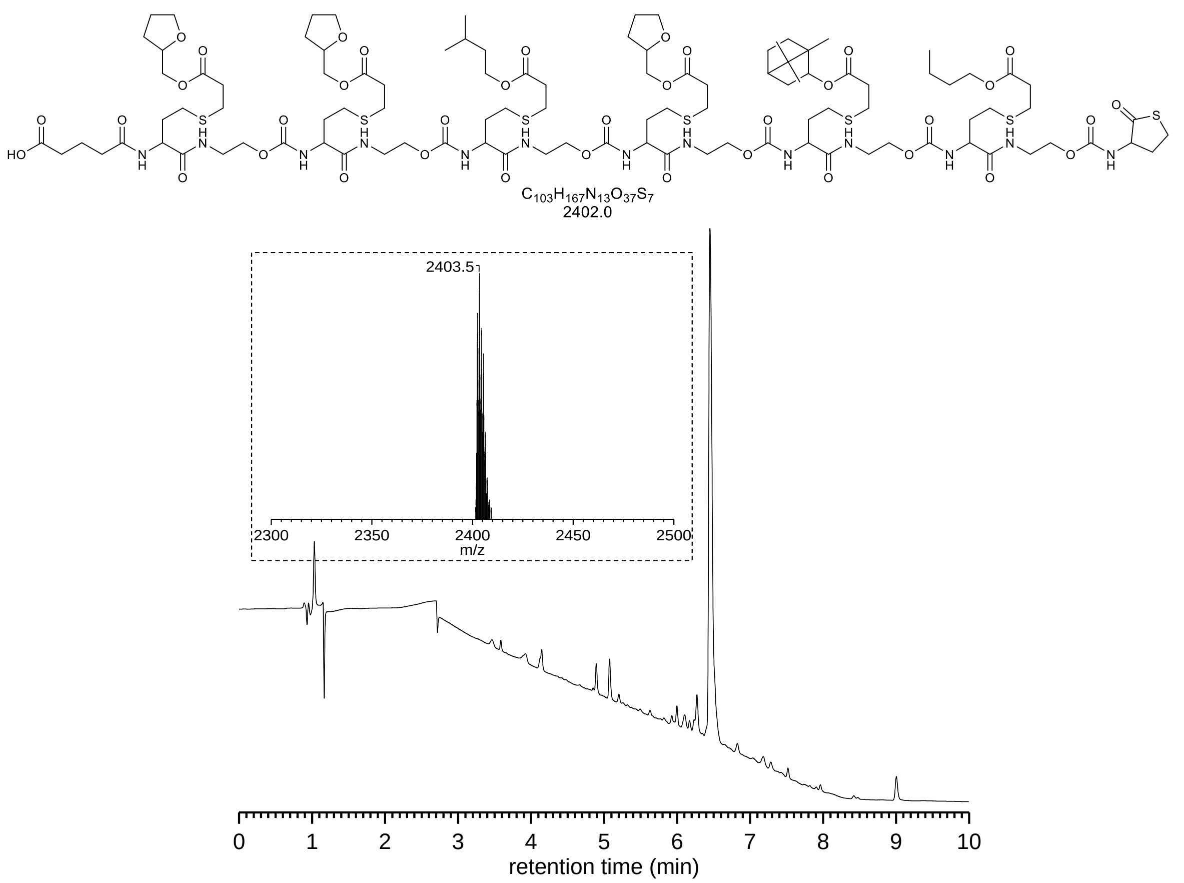

Supplementary Figure 134 | LC-ESI-MS analysis of **QR26**. Insert: ESI-MS-spectrum of dominant species (positive mode).

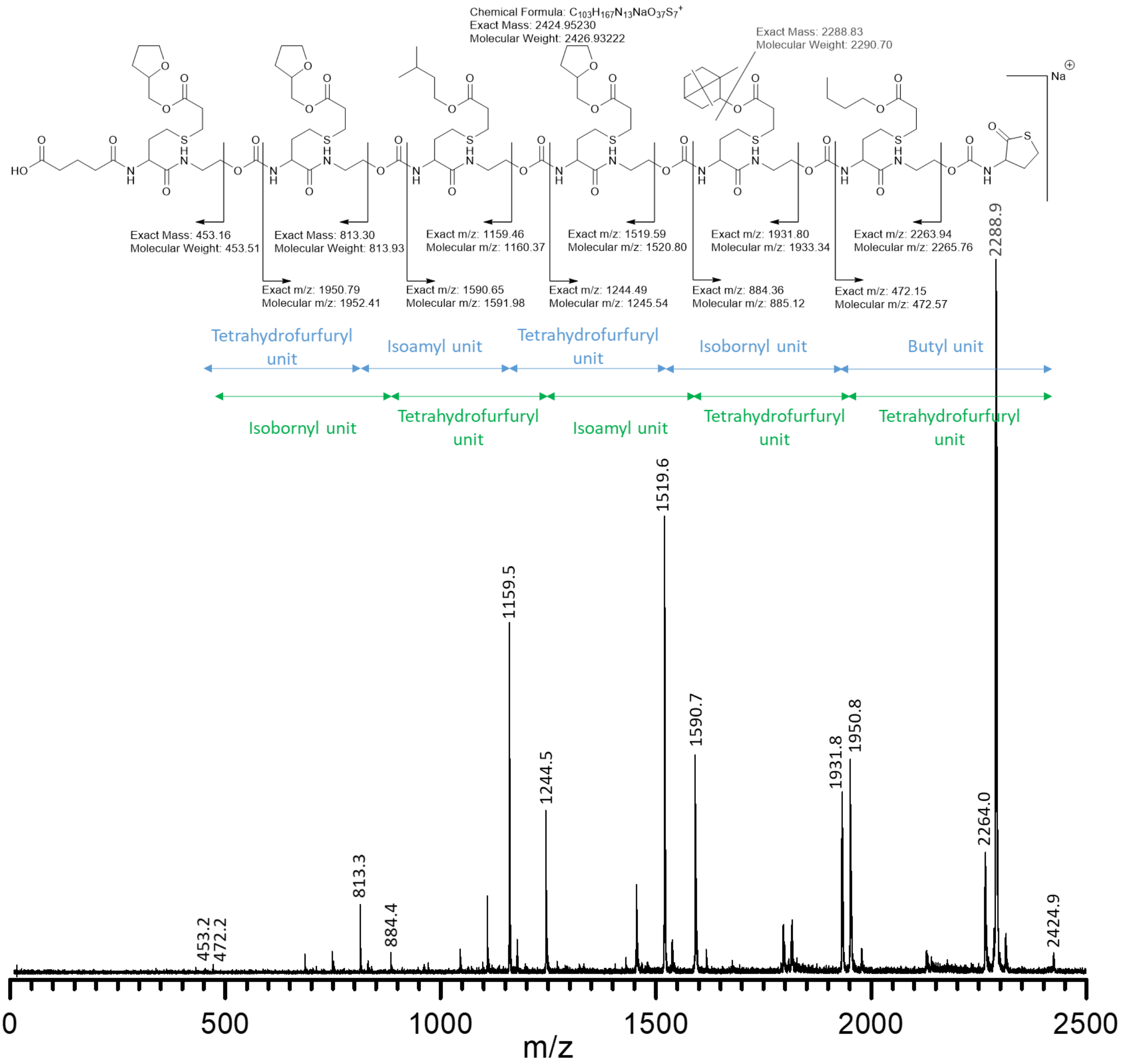

Supplementary Figure 135 | MALDI-MS/MS spectrum with peak assignment of **QR26**.

*Characterization of **QR27** using mass spectrometry (Supplementary Figure 136) and MALDI-MS/MS analysis (Supplementary Figure 137).*

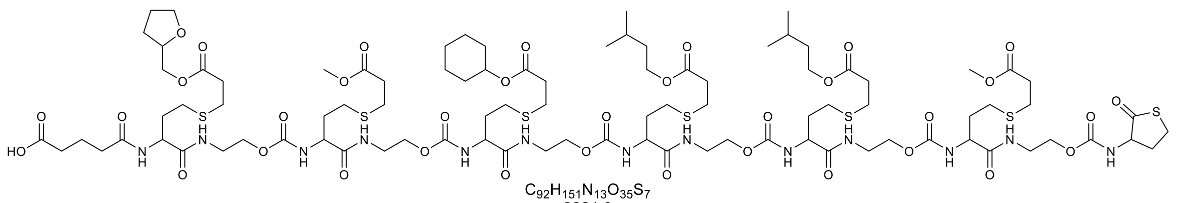

$C_{92}H_{151}N_{13}O_{35}S_7$
2221.9

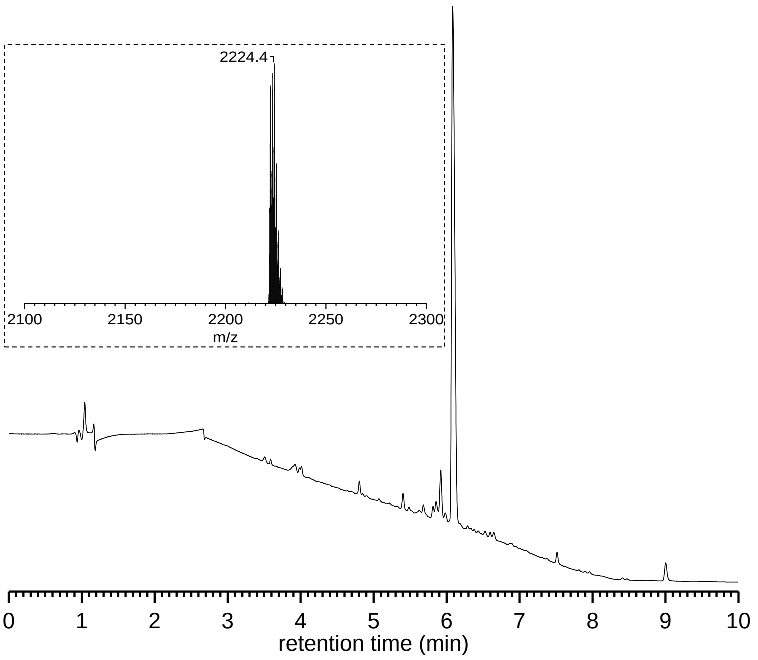

Supplementary Figure 136 | LC-ESI-MS analysis of **QR27**. Insert: ESI-MS-spectrum of dominant species (positive mode).

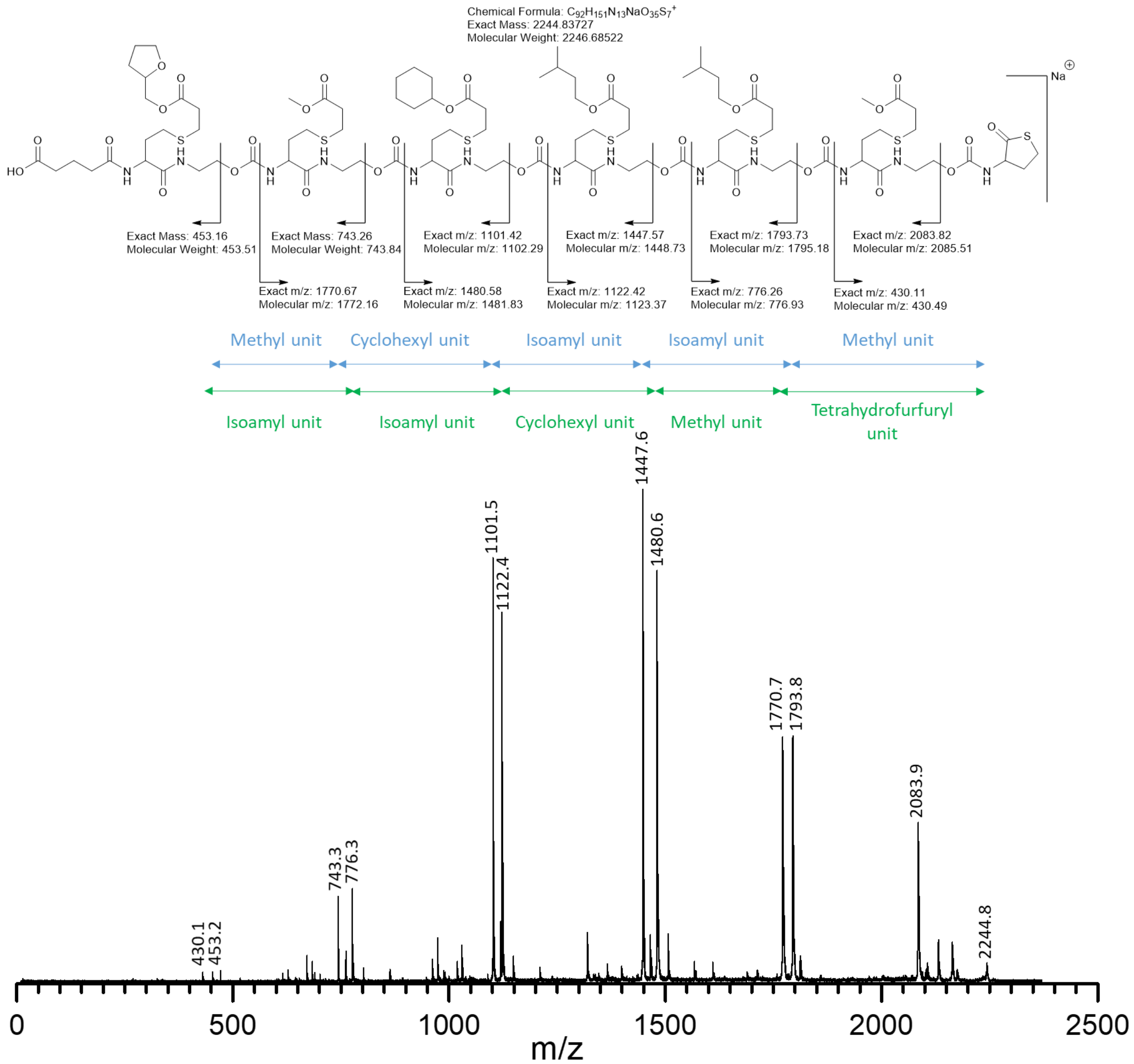

Supplementary Figure 137 | MALDI-MS/MS spectrum with peak assignment of **QR27**.

*Characterization of **QR28** using mass spectrometry (Supplementary Figure 138) and MALDI-MS/MS analysis (Supplementary Figure 139).*

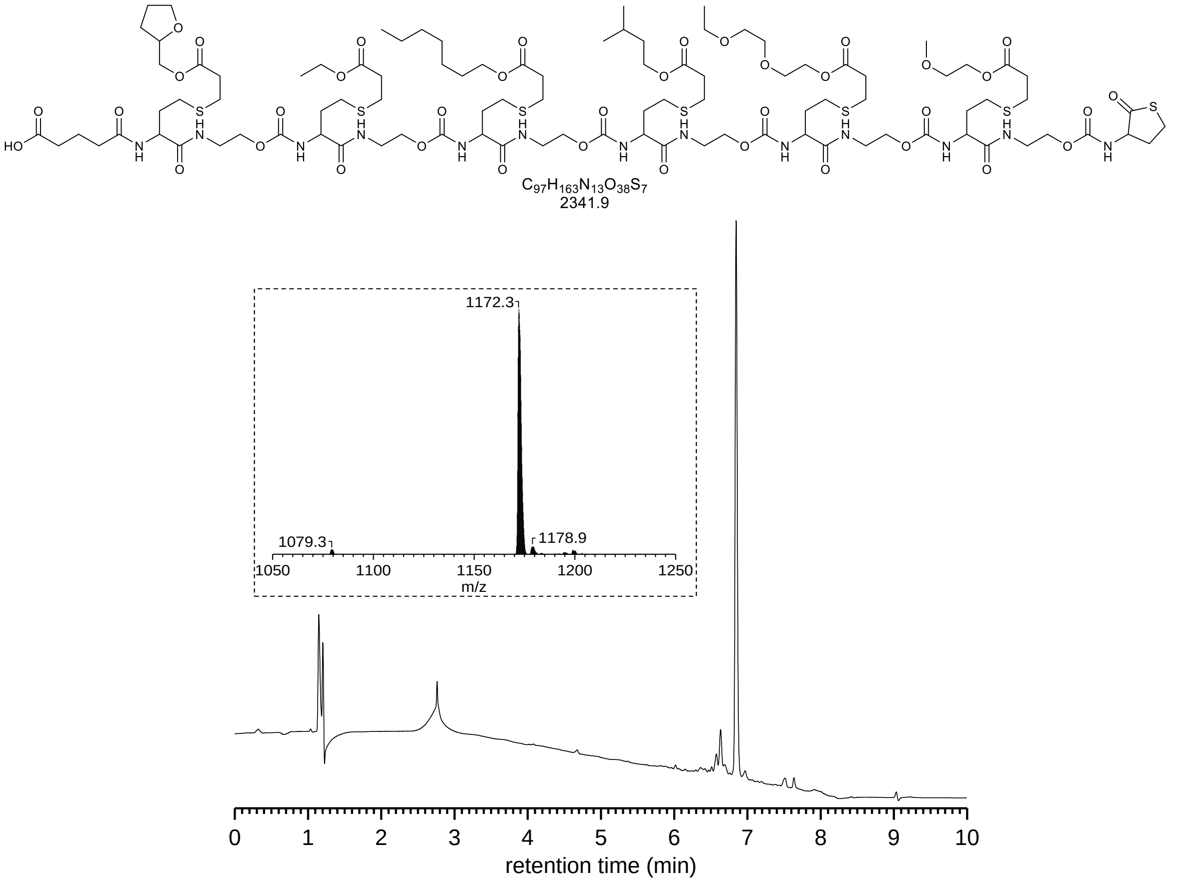

Supplementary Figure 138 | LC-ESI-MS analysis of **QR28**. Insert: ESI-MS-spectrum of dominant species (positive mode).

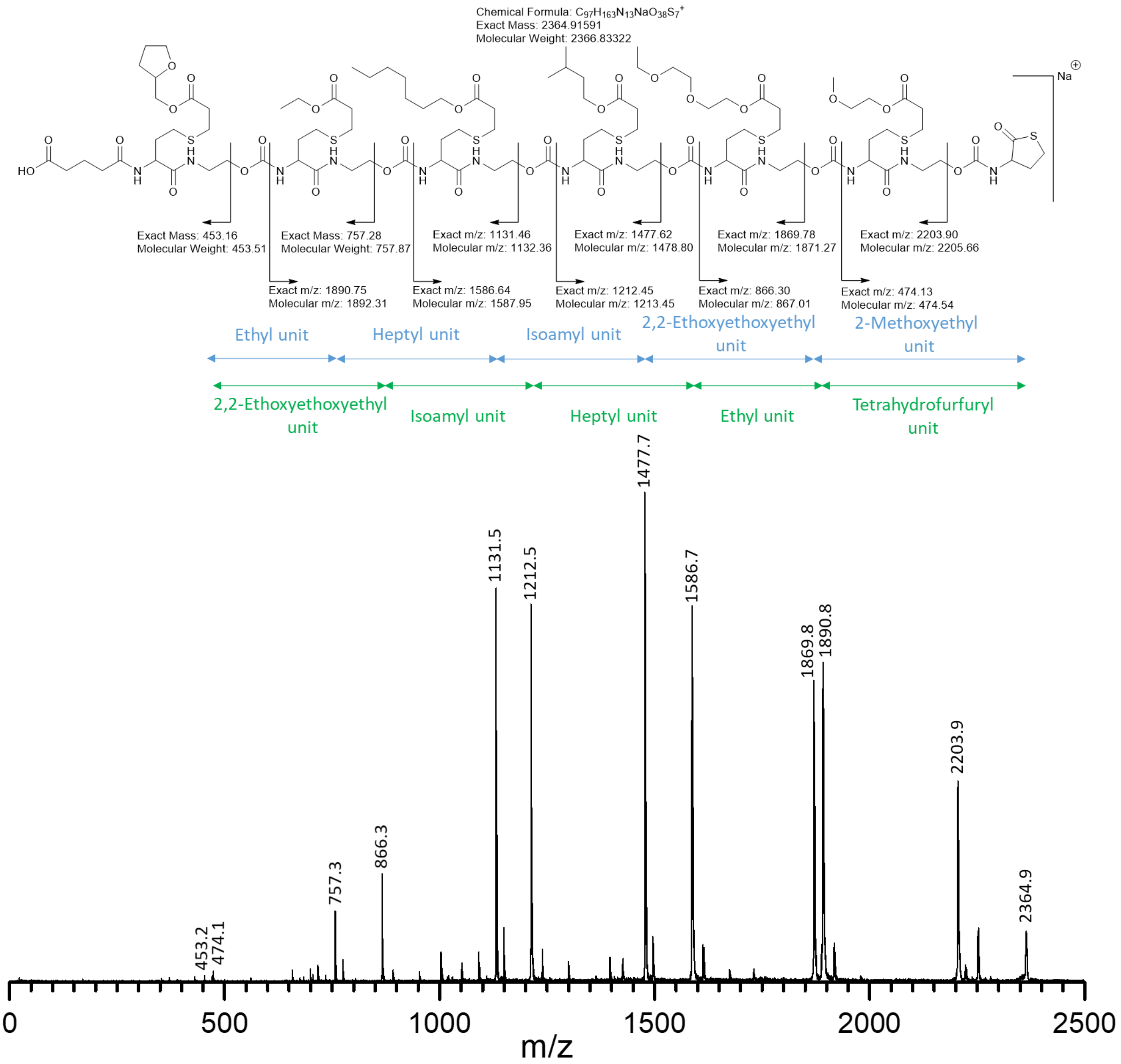

Supplementary Figure 139 | MALDI-MS/MS spectrum with peak assignment of **QR28**.

*Characterization of **QR29** using mass spectrometry (Supplementary Figure 140) and MALDI-MS/MS analysis (Supplementary Figure 141).*

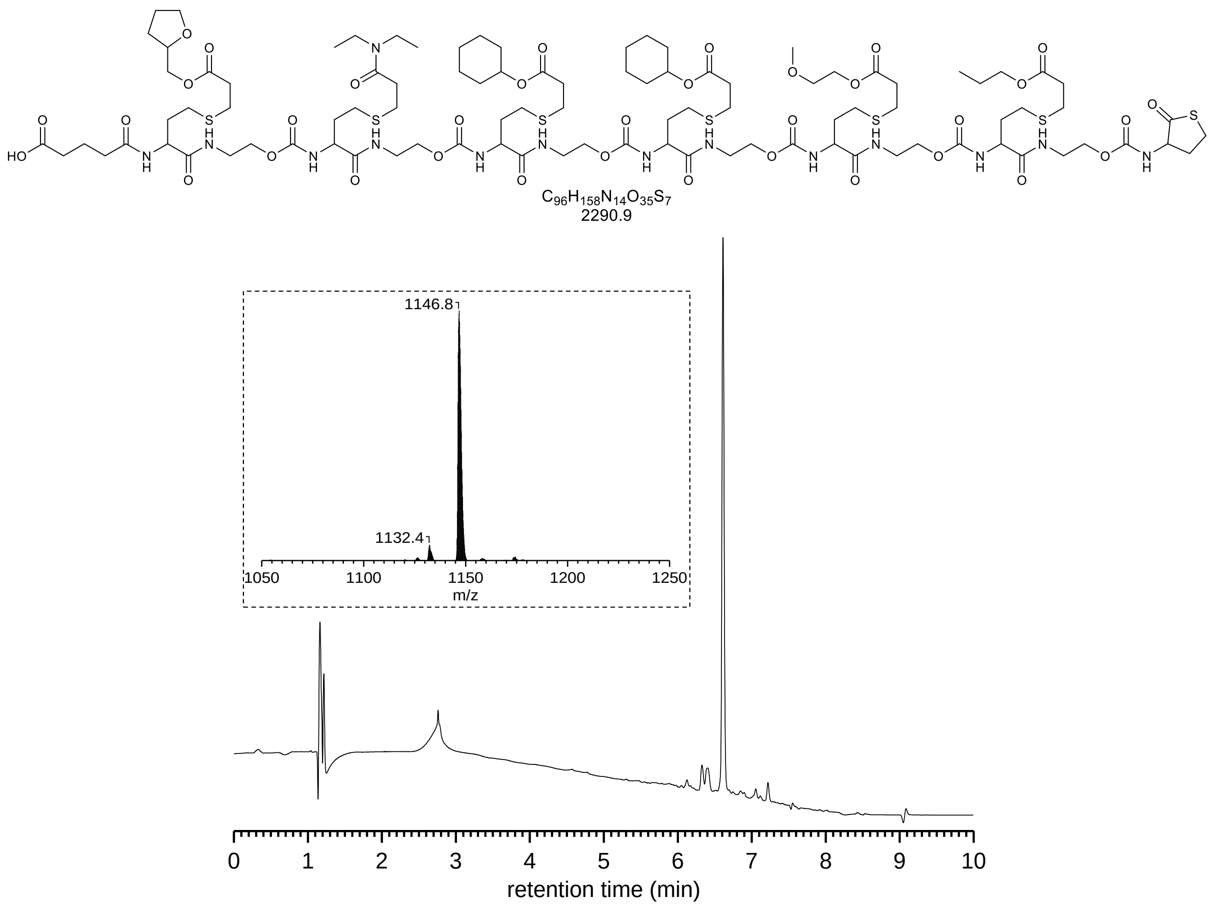

Supplementary Figure 140 | LC-ESI-MS analysis of **QR29**. Insert: ESI-MS-spectrum of dominant species (positive mode).

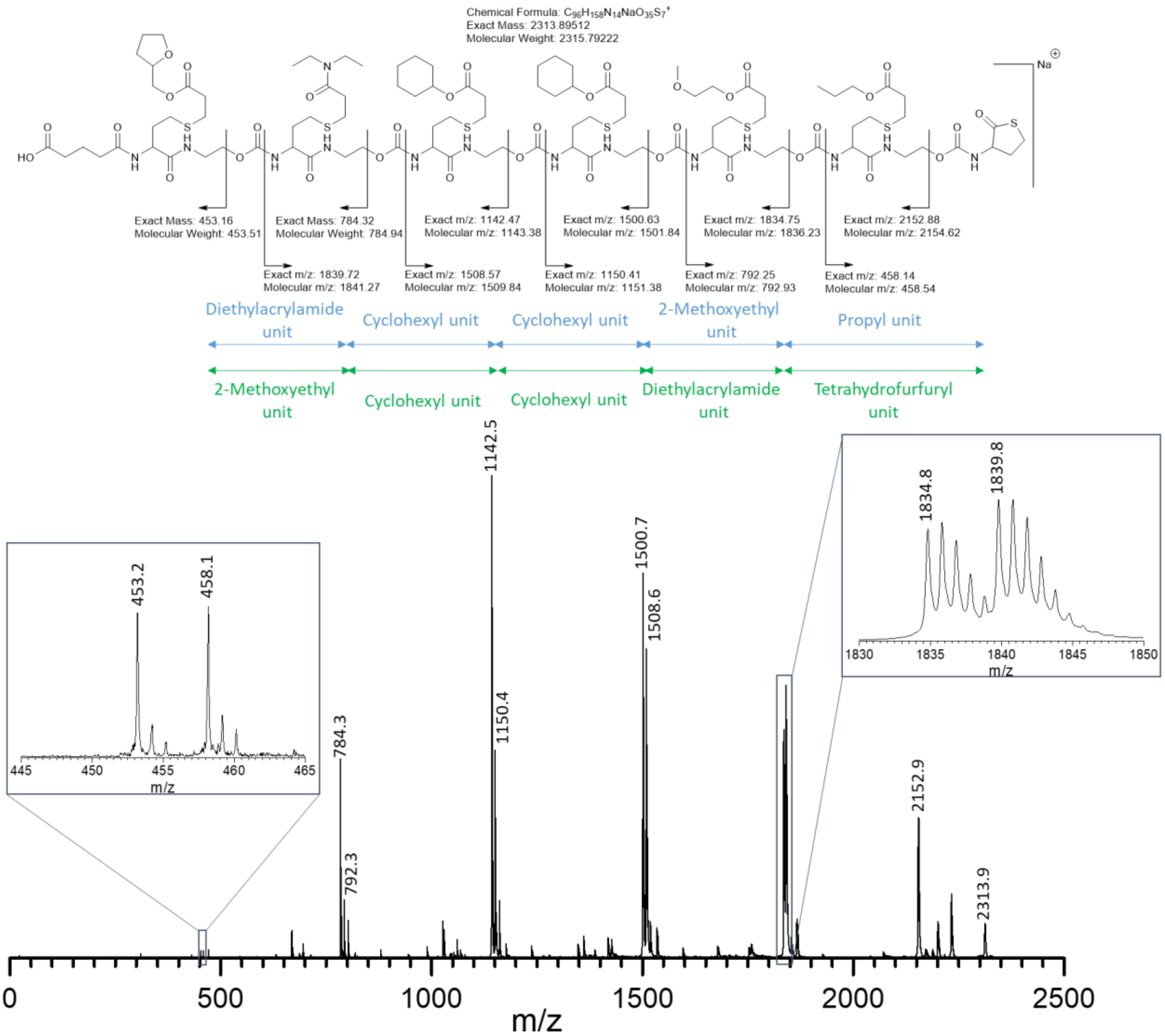

Supplementary Figure 141 | MALDI-MS/MS spectrum with peak assignment of **QR29**.

*Characterization of **QR30** using mass spectrometry (*Supplementary *Figure 142) and MALDI-MS/MS analysis (*Supplementary *Figure 143).*

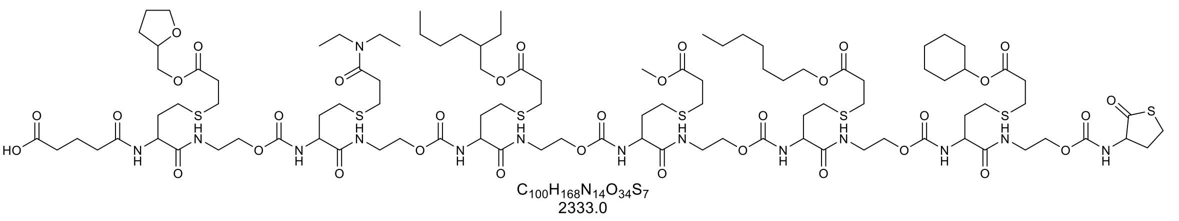

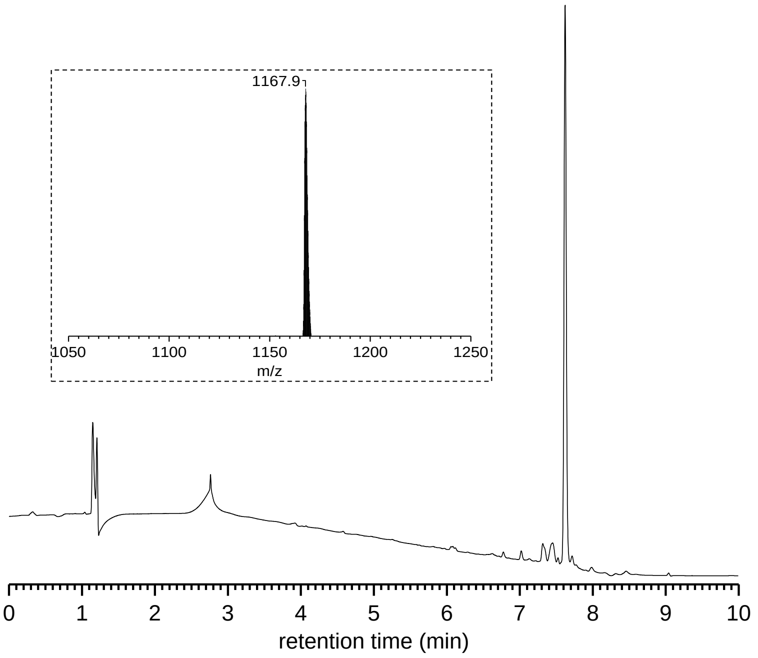

Supplementary Figure 142 | LC-ESI-MS analysis of **QR30**. Insert: ESI-MS-spectrum of dominant species (positive mode).

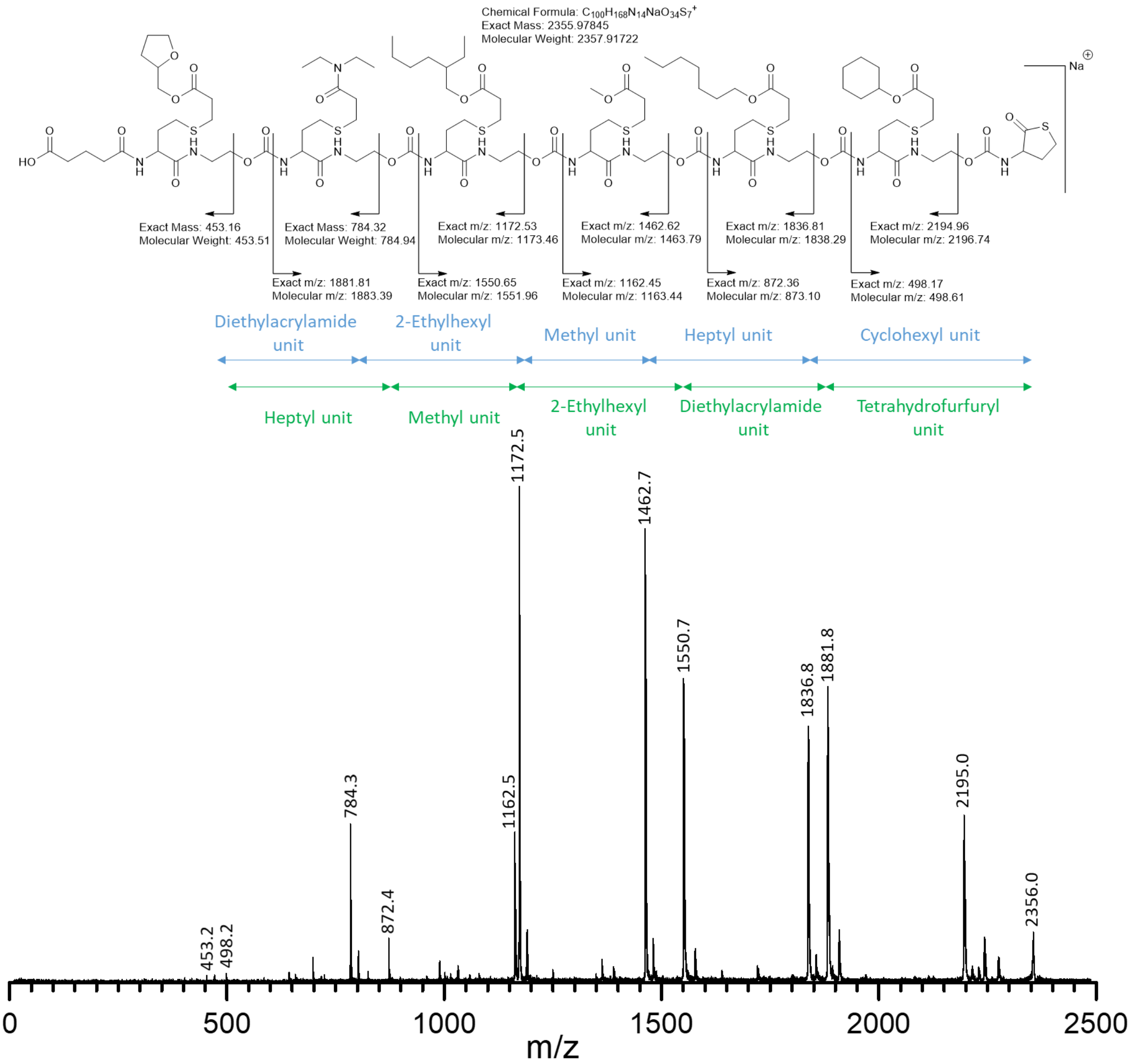

Supplementary Figure 143 | MALDI-MS/MS spectrum with peak assignment of **QR30**.

Characterization of ***QR31*** *using mass spectrometry (Supplementary Figure 144) and MALDI-MS/MS analysis (Supplementary Figure 145).*

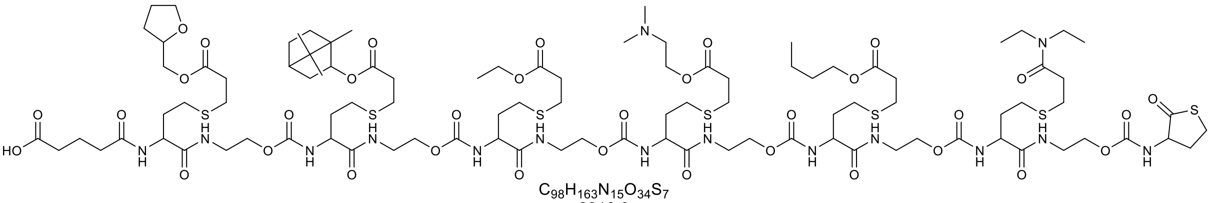

$C_{98}H_{163}N_{15}O_{34}S_7$
2318.0

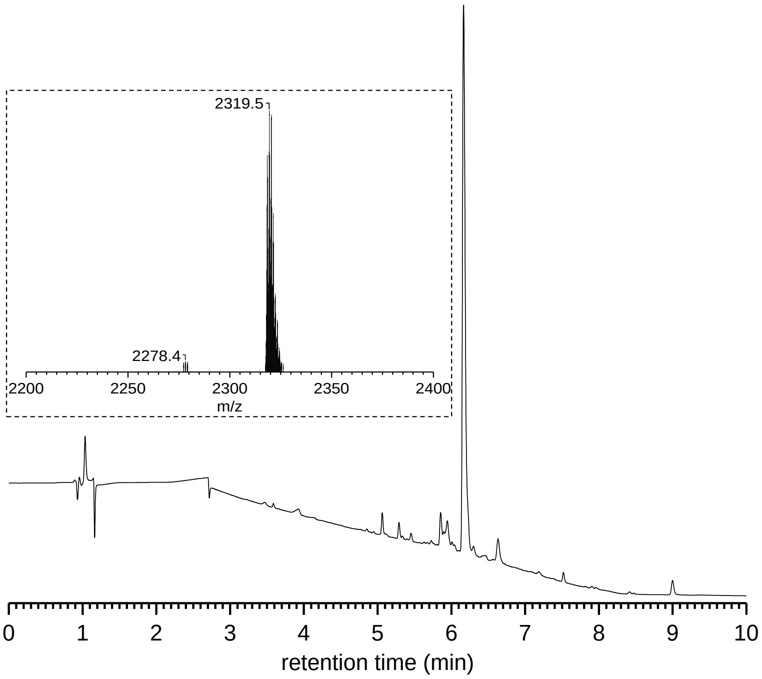

Supplementary Figure 144 | LC-ESI-MS analysis of **QR31**. Insert: ESI-MS-spectrum of dominant species (positive mode).

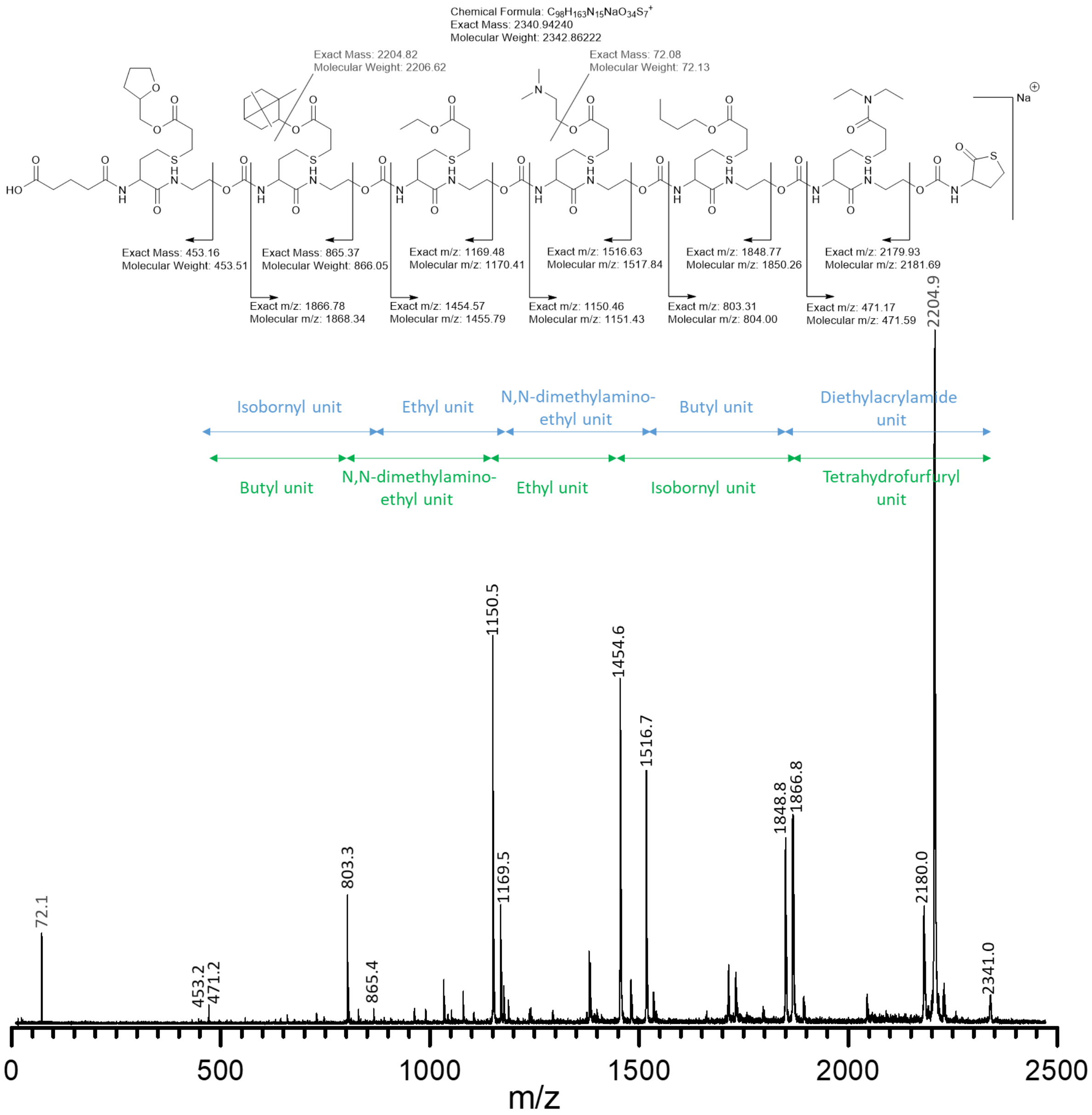

Supplementary Figure 145 | MALDI-MS/MS spectrum with peak assignment of **QR31**.

*Characterization of **QR32** using mass spectrometry (Supplementary Figure 146) and MALDI-MS/MS analysis (Supplementary Figure 147).*

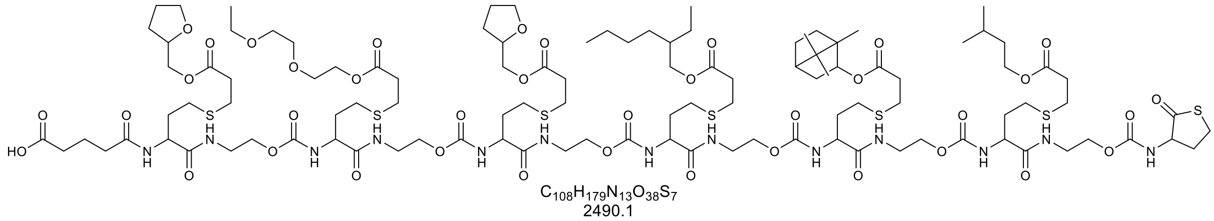

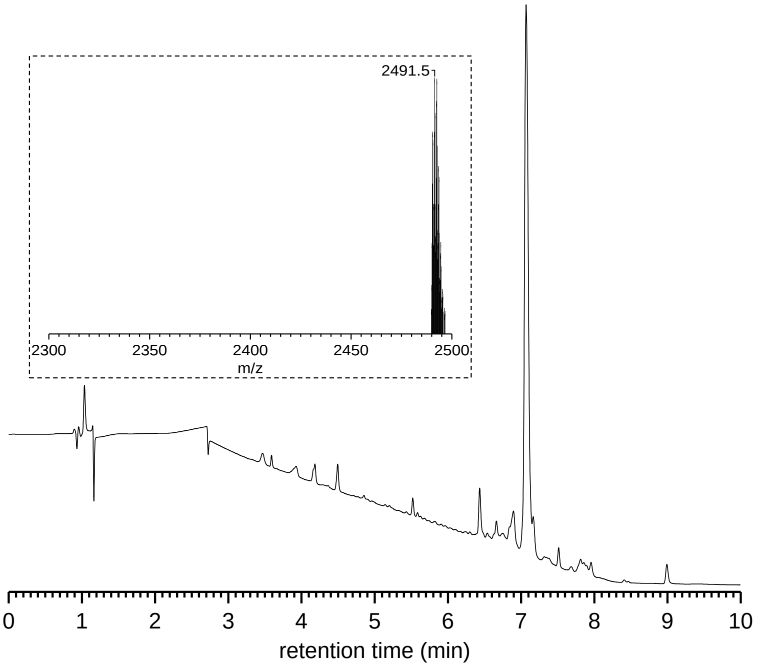

Supplementary Figure 146 | LC-ESI-MS analysis of **QR32**. Insert: ESI-MS-spectrum of dominant species (positive mode).

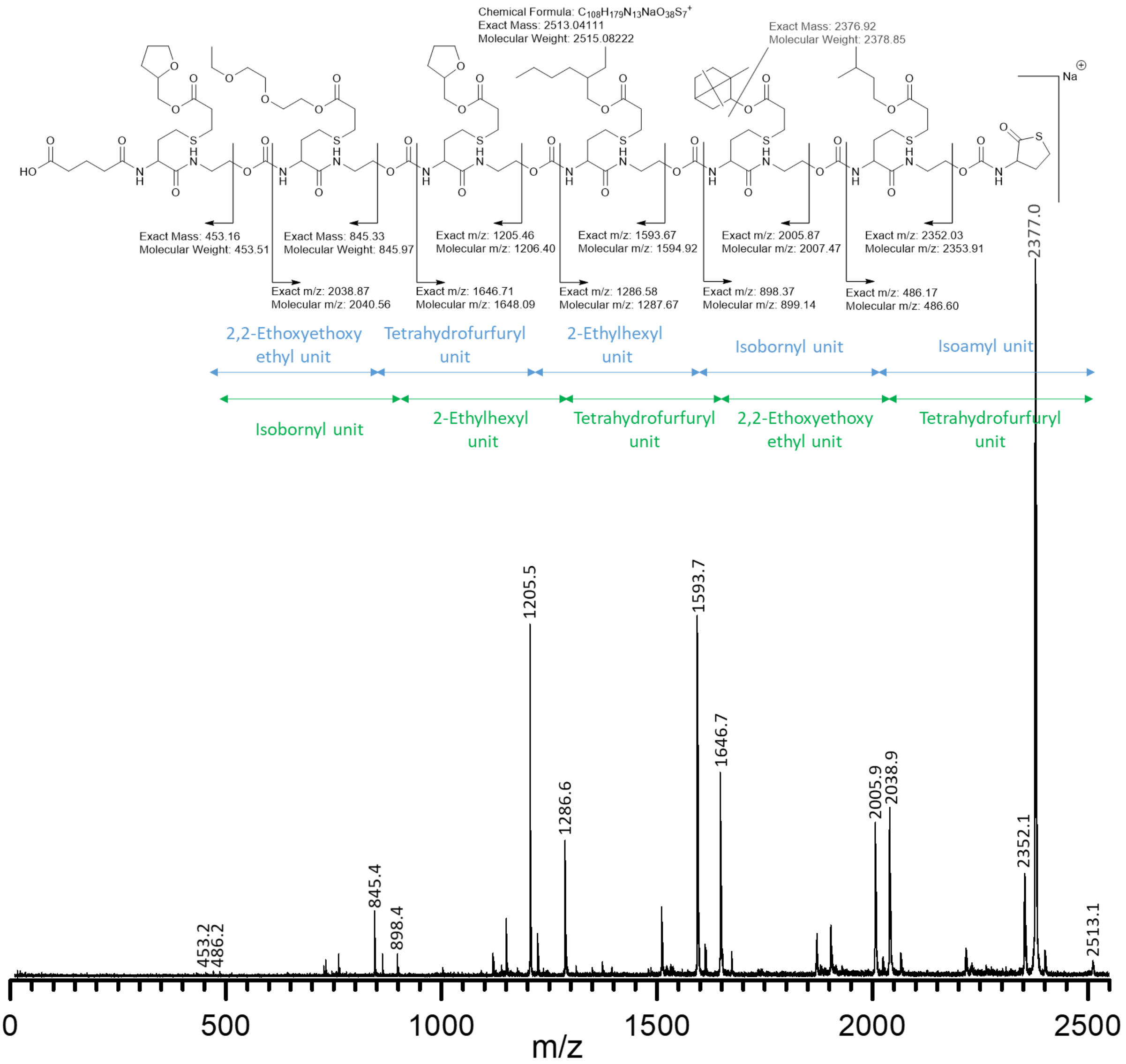

Supplementary Figure 147 | MALDI-MS/MS spectrum with peak assignment of **QR32**.

Characterization of ***QR33*** *using mass spectrometry (Supplementary Figure 148) and MALDI-MS/MS analysis (Supplementary Figure 149).*

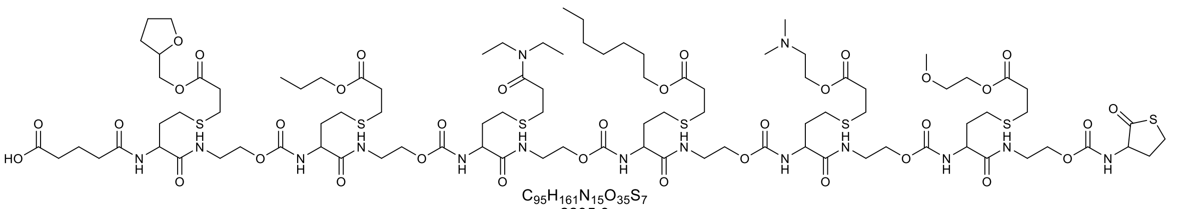

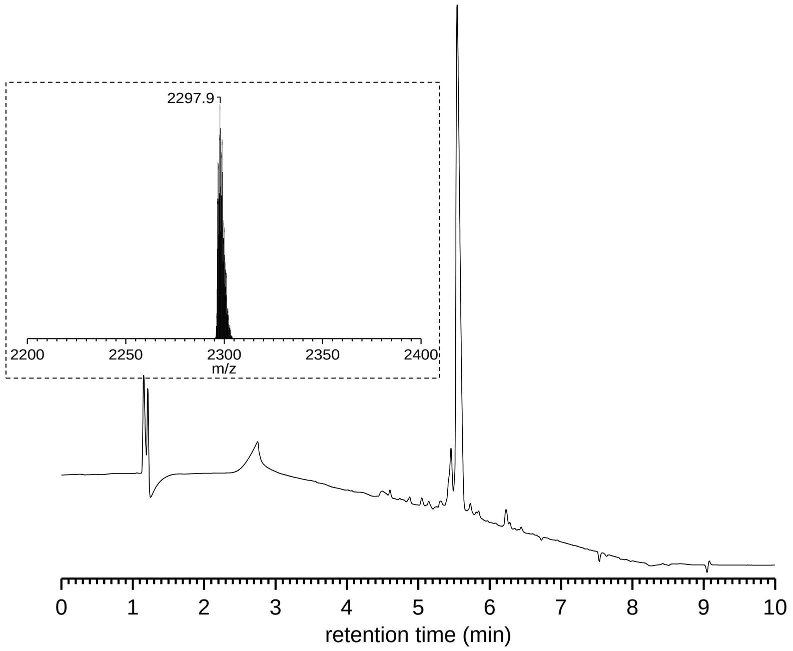

Supplementary Figure 148 | LC-ESI-MS analysis of **QR33**. Insert: ESI-MS-spectrum of dominant species (positive mode).

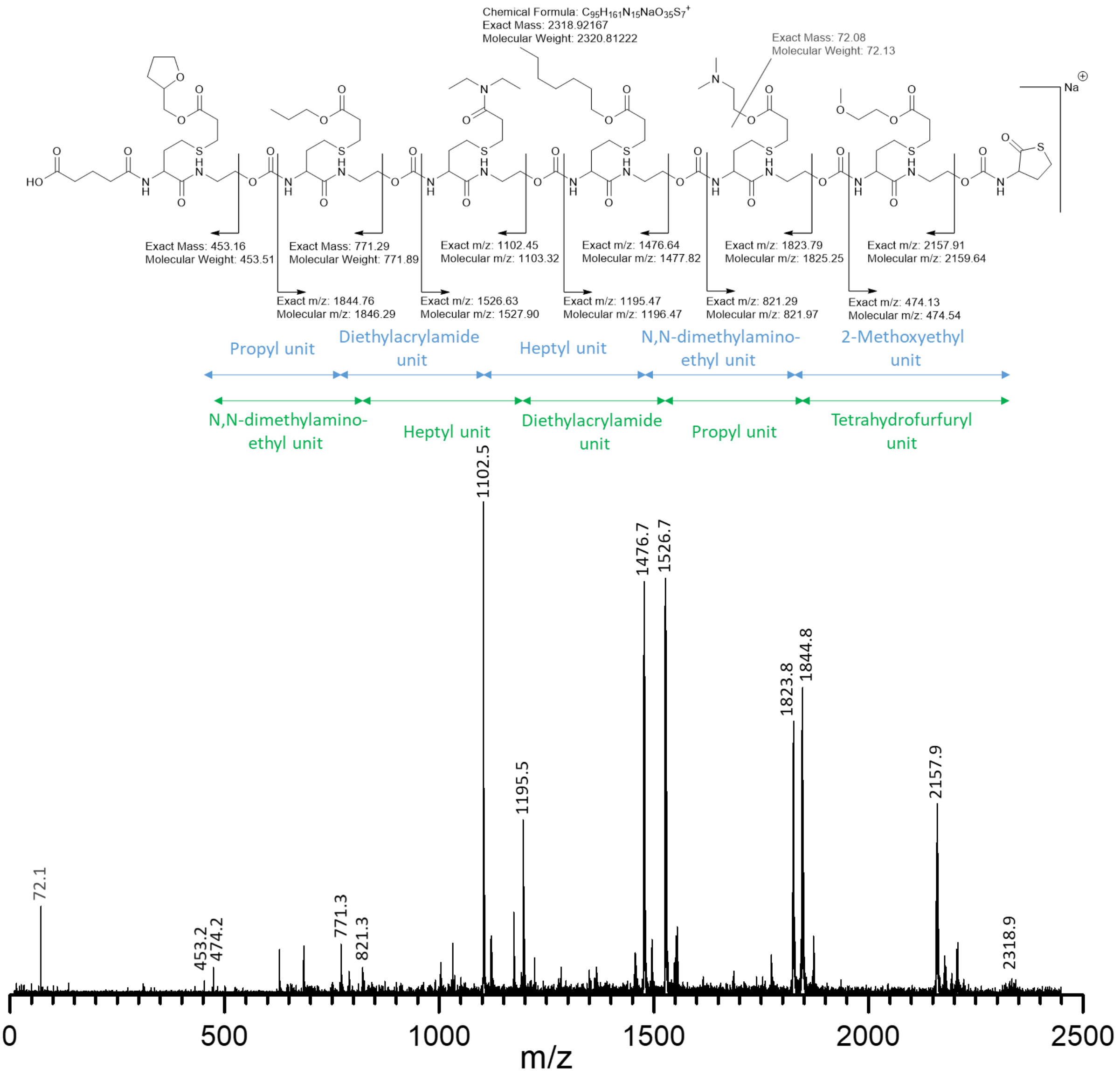

Supplementary Figure 149 | MALDI-MS/MS spectrum with peak assignment of **QR33**.

*Characterization of **QR34** using mass spectrometry (Supplementary Figure 150) and MALDI-MS/MS analysis (Supplementary Figure 151).*

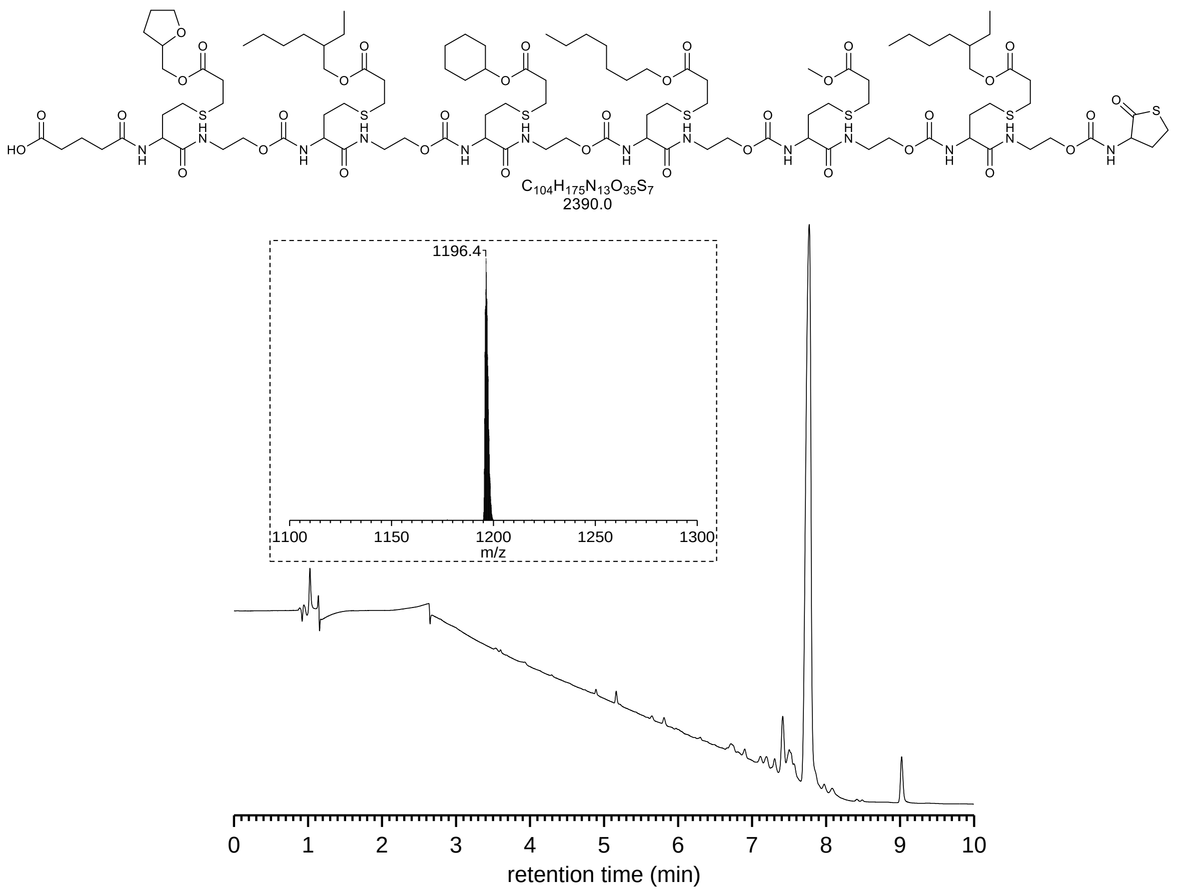

Supplementary Figure 150 | LC-ESI-MS analysis of **QR34**. Insert: ESI-MS-spectrum of dominant species (positive mode).

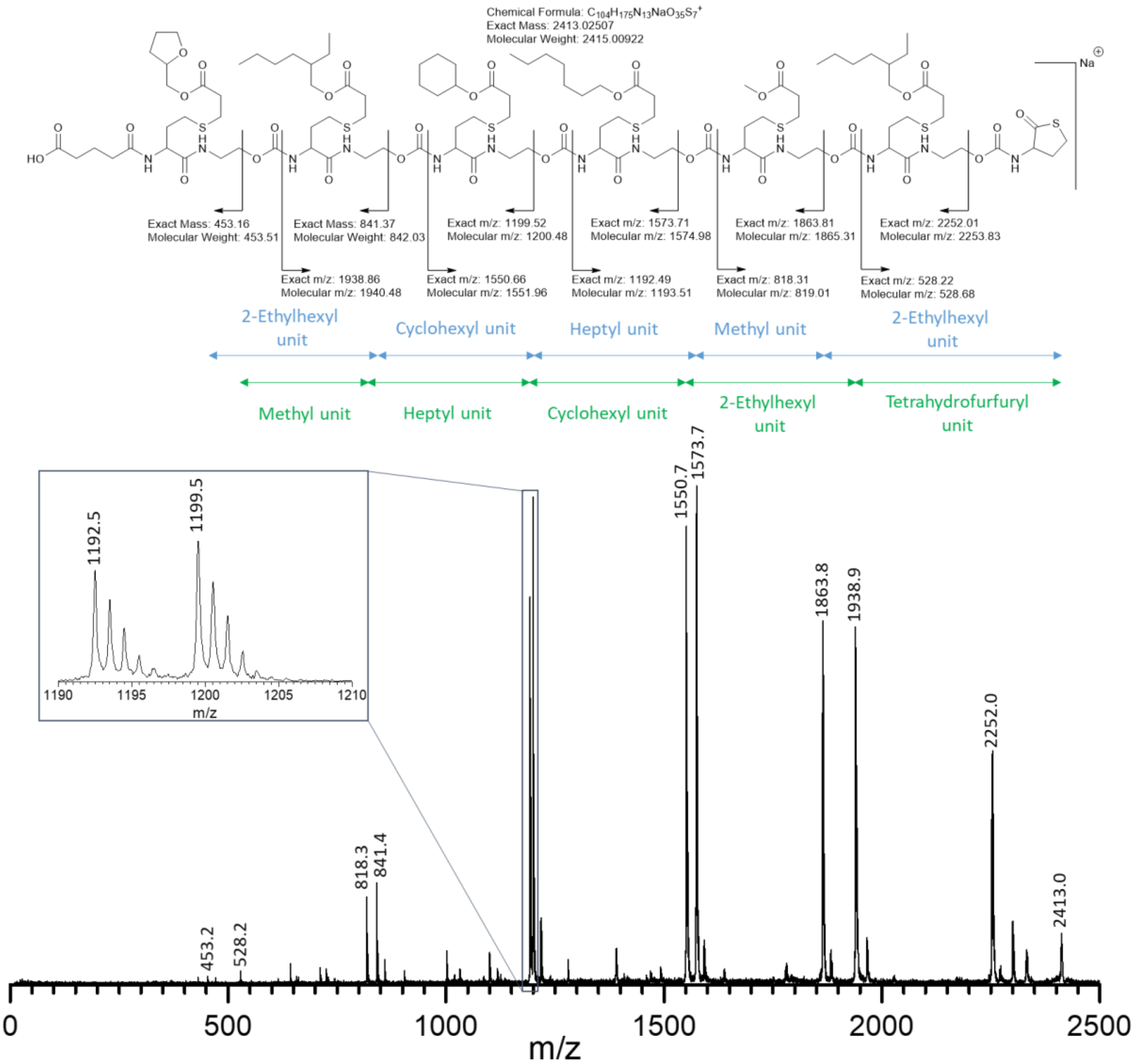

Supplementary Figure 151 | MALDI-MS/MS spectrum with peak assignment of **QR34**.

*Characterization of **QR35** using mass spectrometry (Supplementary Figure 152) and MALDI-MS/MS analysis (Supplementary Figure 153).*

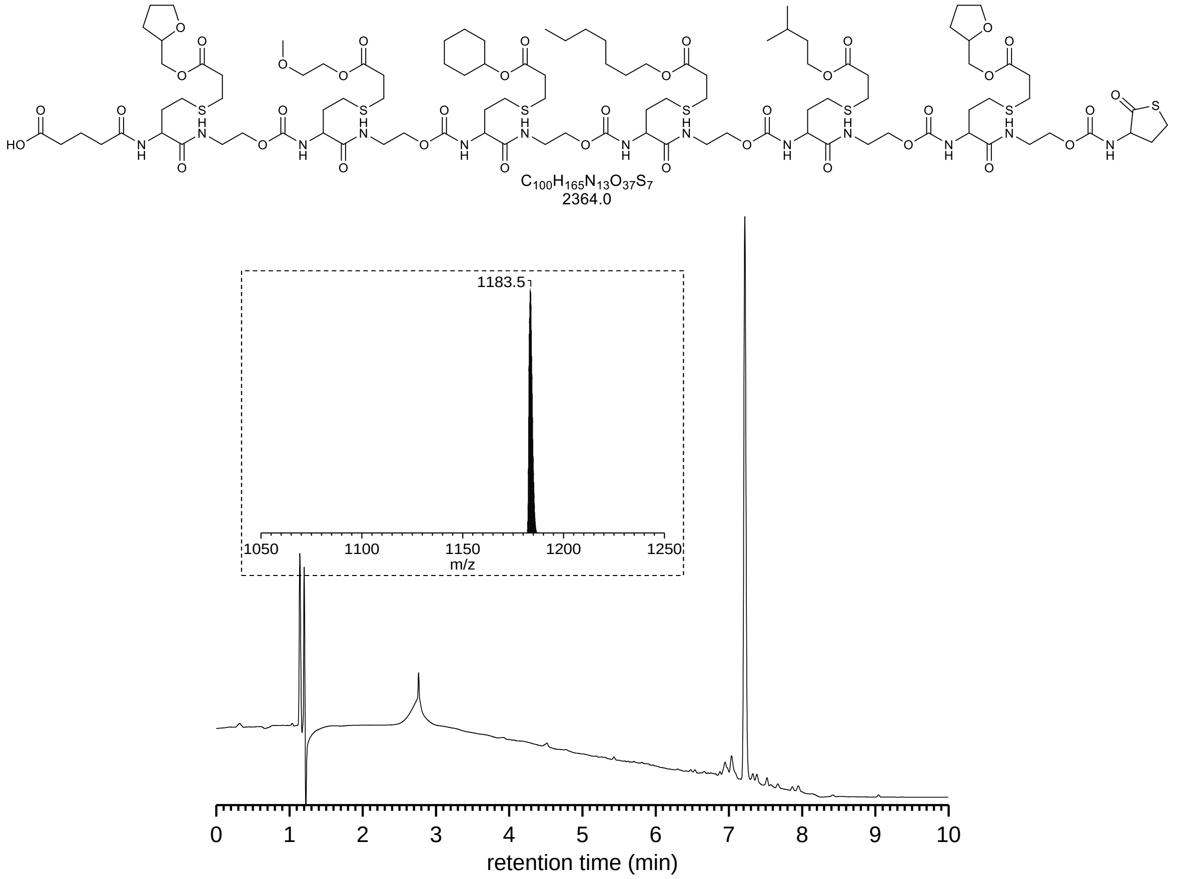

Supplementary Figure 152 | LC-ESI-MS analysis of **QR35**. Insert: ESI-MS-spectrum of dominant species (positive mode).

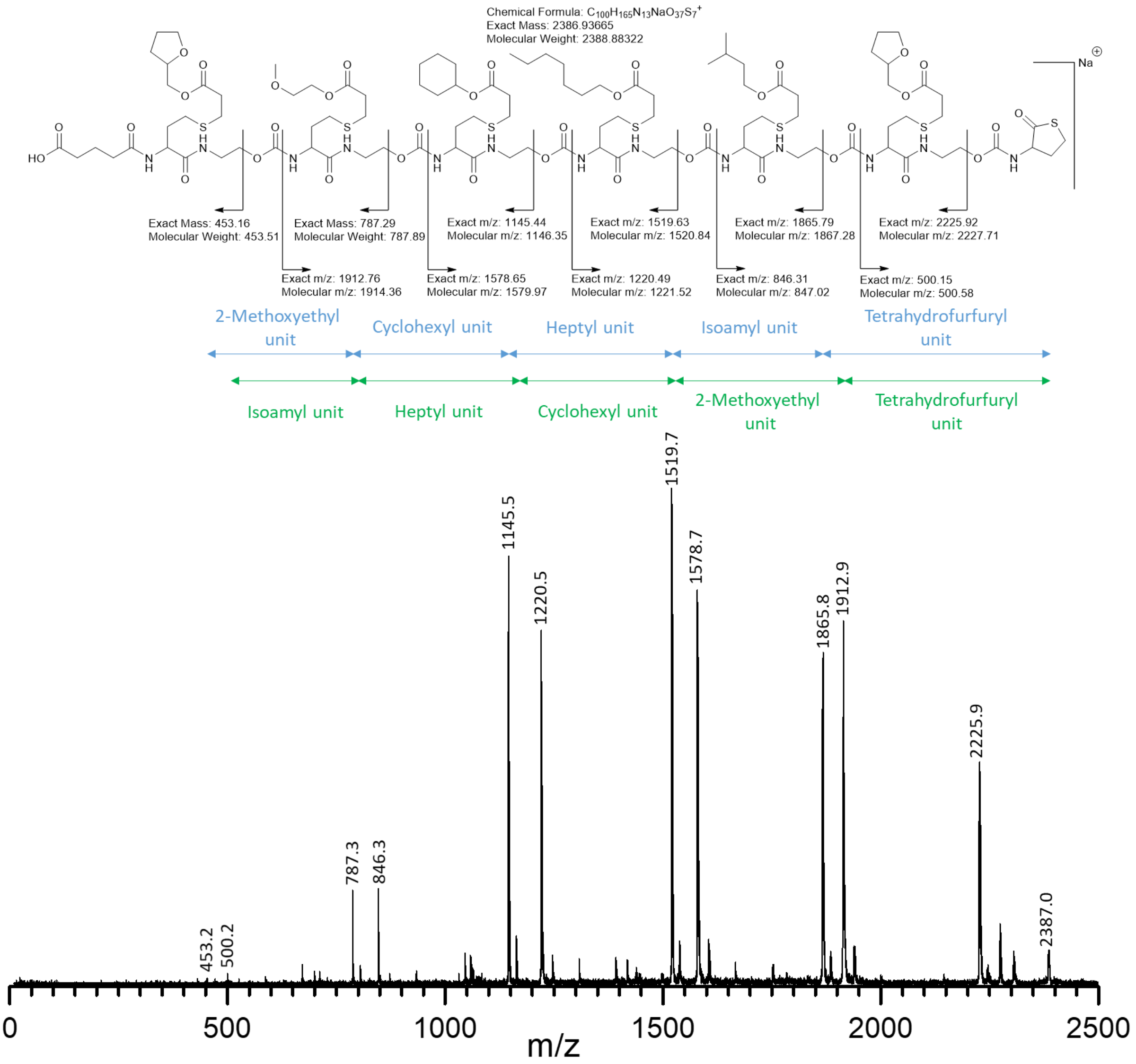

Supplementary Figure 153 | MALDI-MS/MS spectrum with peak assignment of **QR35**.

*Characterization of **QR36** using mass spectrometry (Supplementary Figure 154) and MALDI-MS/MS analysis (Supplementary Figure 155).*

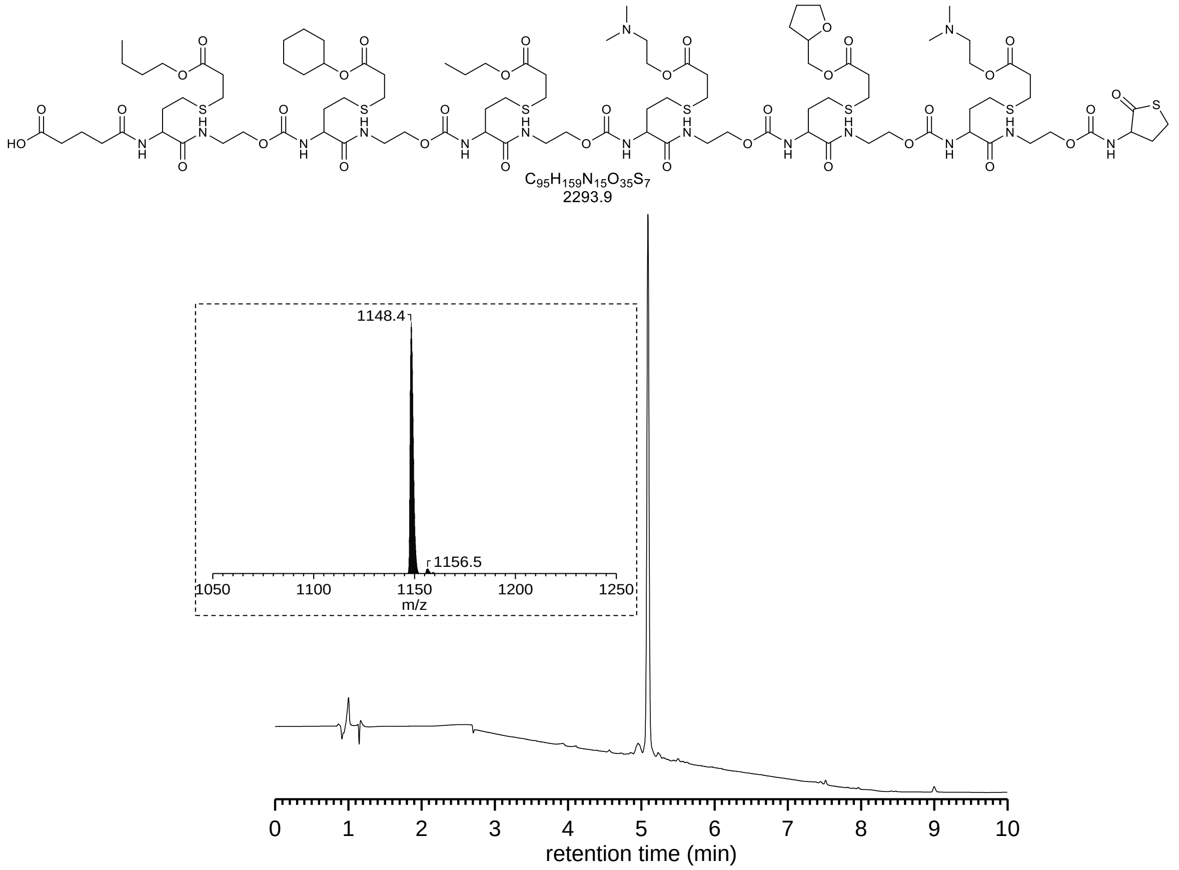

Supplementary Figure 154 | LC-ESI-MS analysis of **QR36**. Insert: ESI-MS-spectrum of dominant species (positive mode).

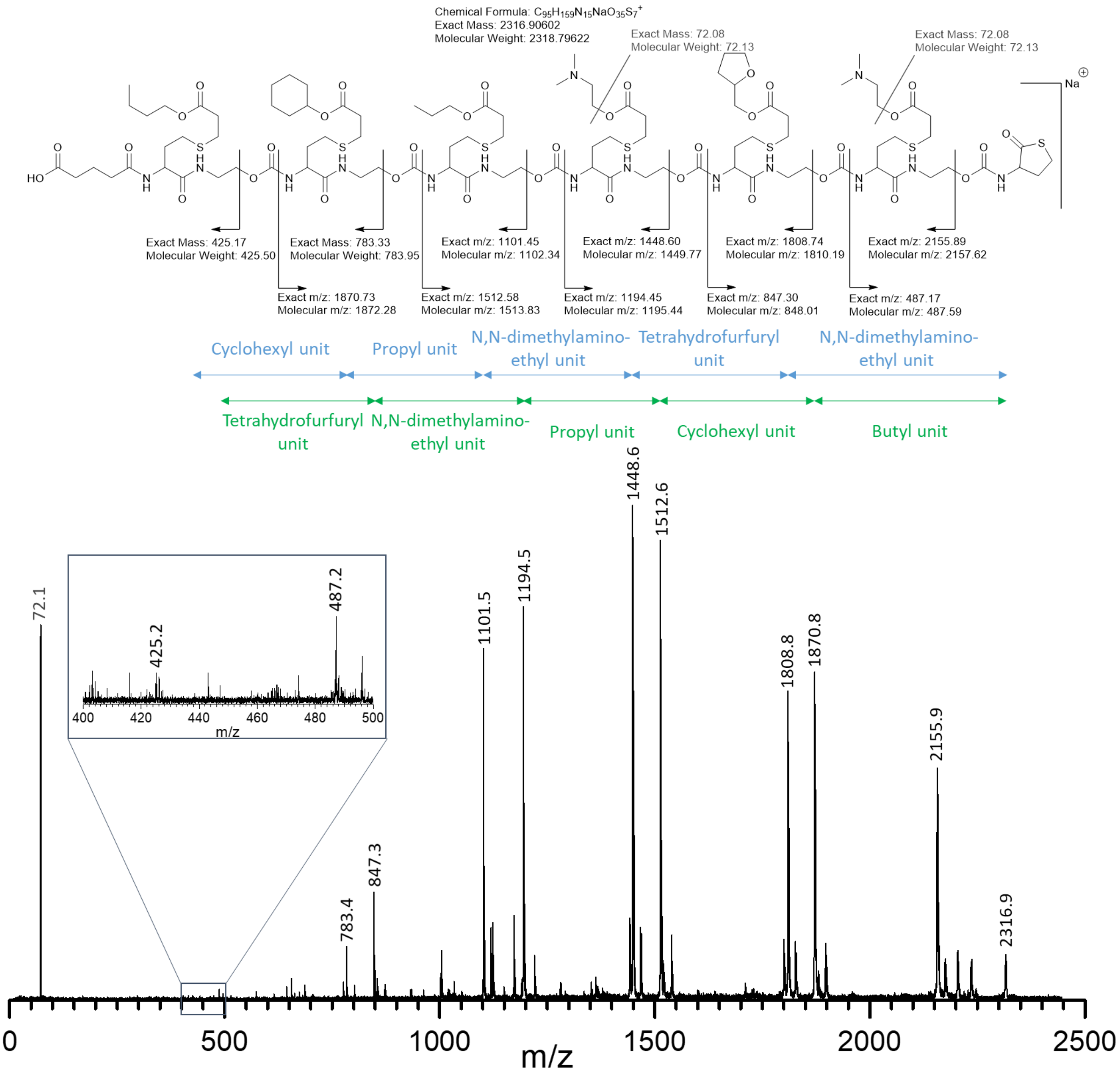

Supplementary Figure 155 | MALDI-MS/MS spectrum with peak assignment of **QR36**.

*Characterization of **QR37** using mass spectrometry (Supplementary Figure 156) and MALDI-MS/MS analysis (Supplementary Figure 157).*

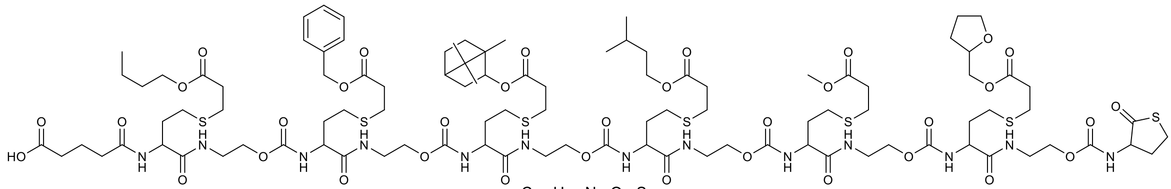

$C_{101}H_{159}N_{13}O_{35}S_7$
2337.9

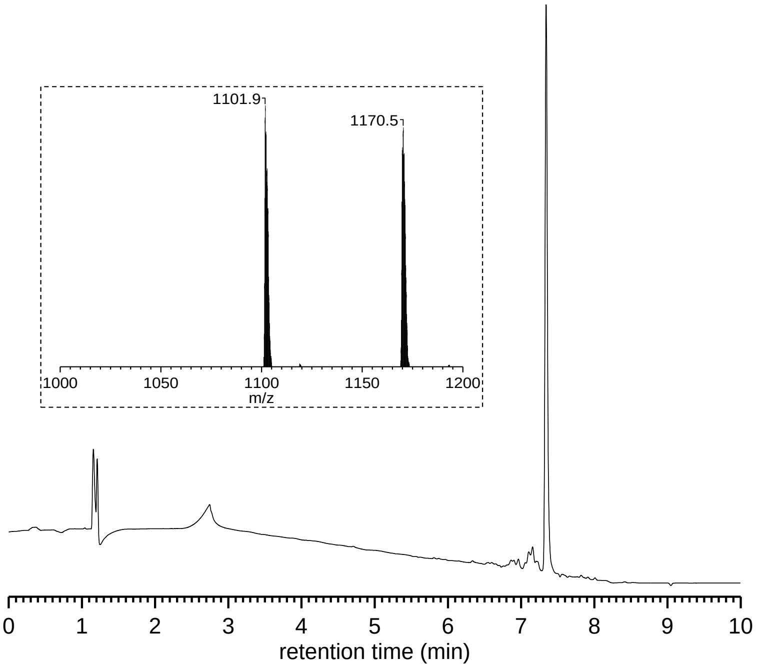

Supplementary Figure 156 | LC-ESI-MS analysis of **QR37**. Insert: ESI-MS-spectrum of dominant species (positive mode).

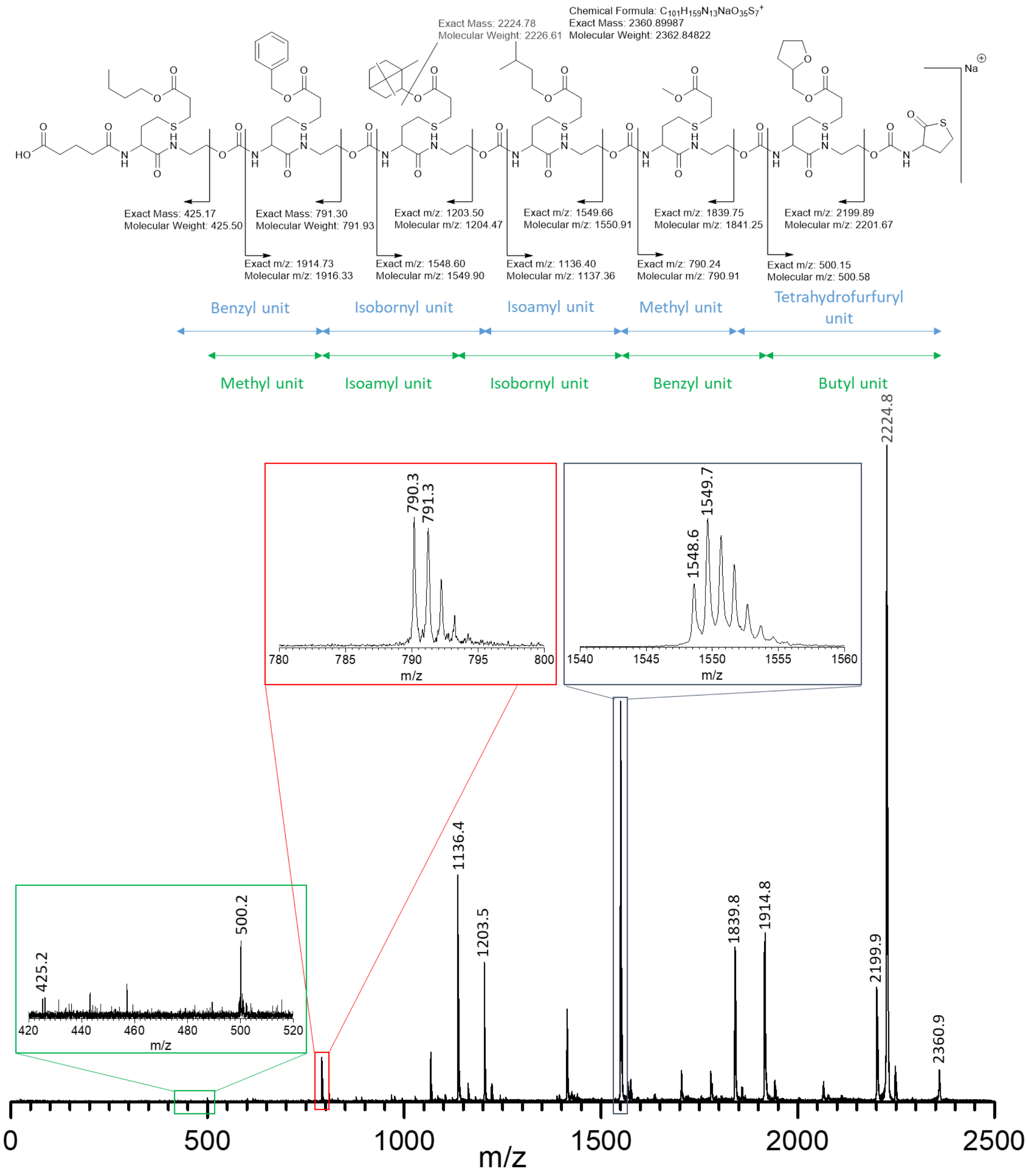

Supplementary Figure 157 | MALDI-MS/MS spectrum with peak assignment of **QR37**.

*Characterization of **QR38** using mass spectrometry (Supplementary Figure 158) and MALDI-MS/MS analysis (Supplementary Figure 159).*

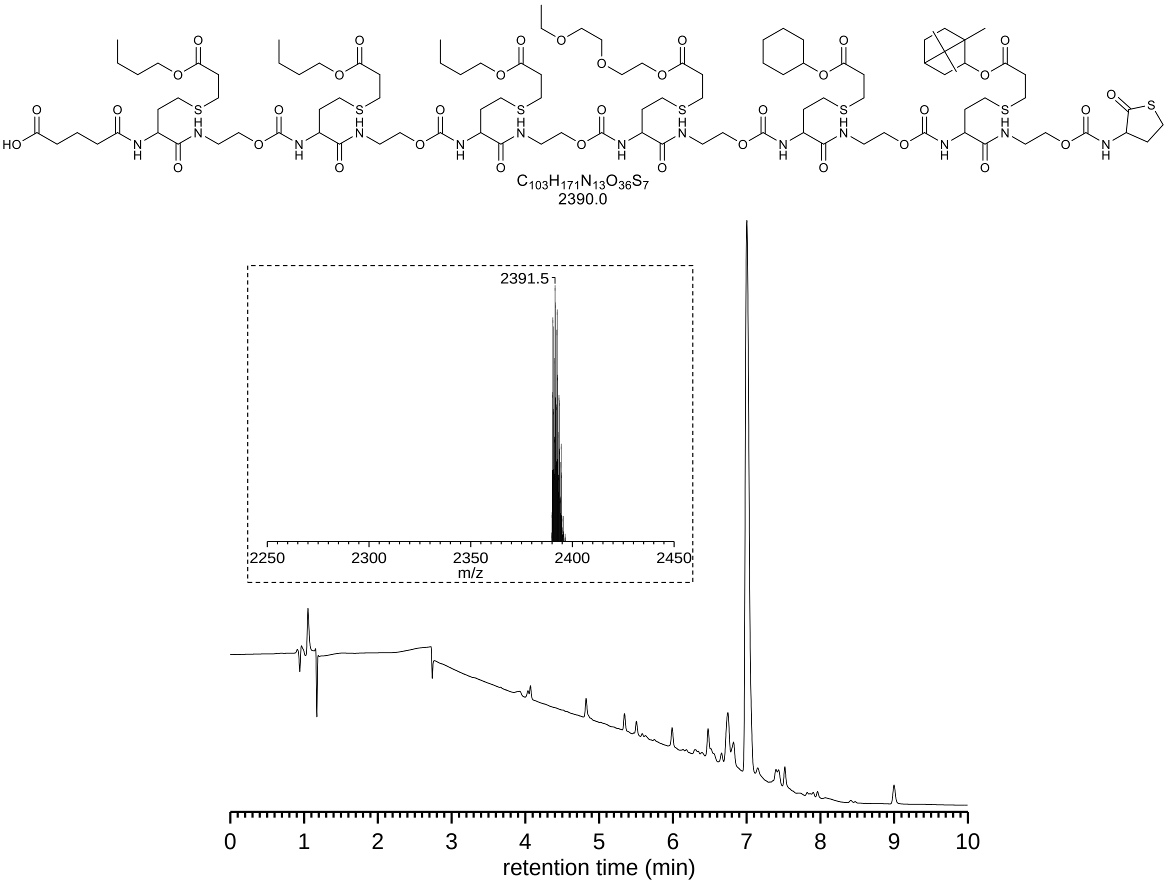

Supplementary Figure 158 | LC-ESI-MS analysis of **QR38**. Insert: ESI-MS-spectrum of dominant species (positive mode).

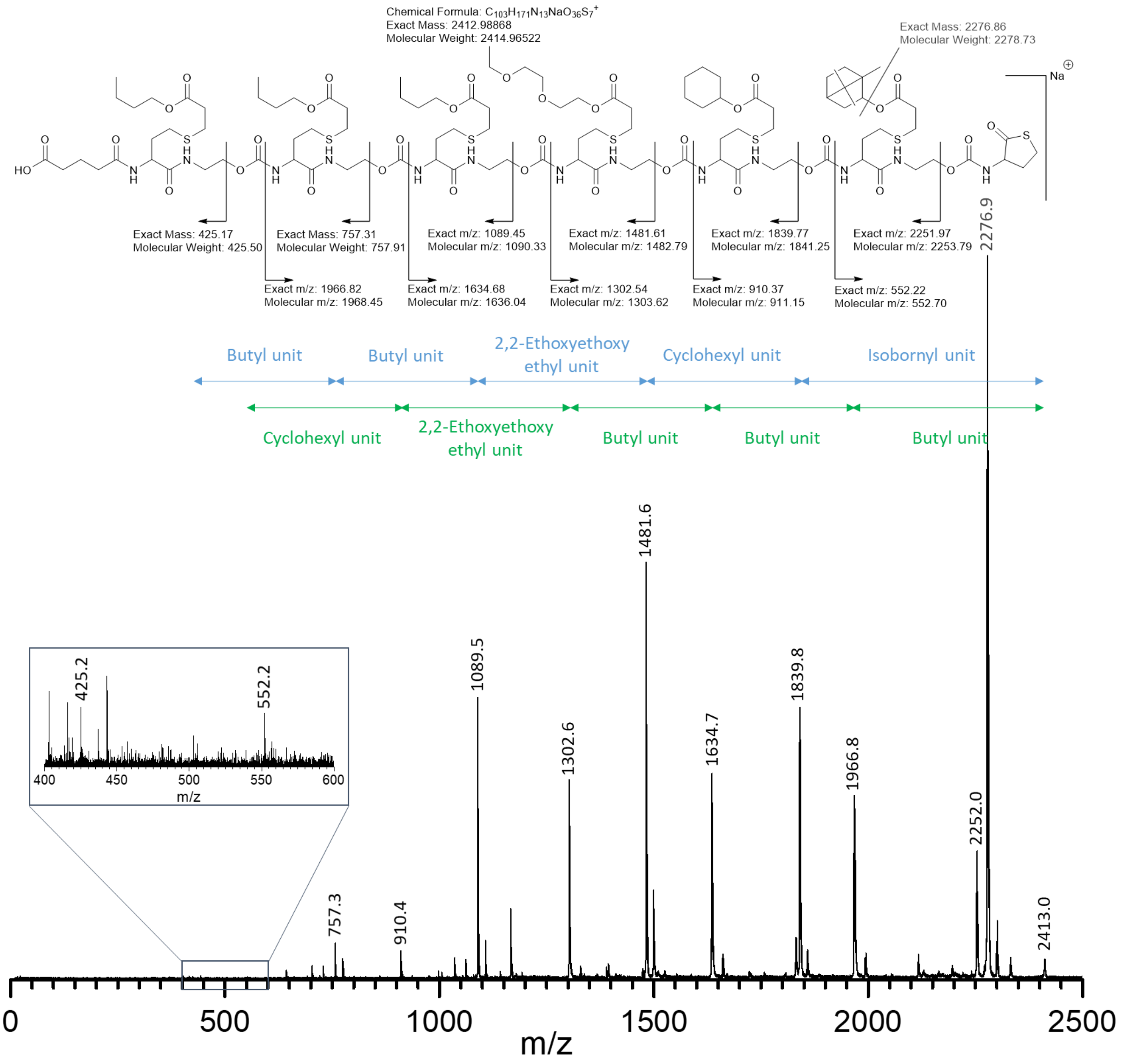

Supplementary Figure 159 | MALDI-MS/MS spectrum with peak assignment of **QR38**.

*Characterization of **QR39** using mass spectrometry (Supplementary Figure 160) and MALDI-MS/MS analysis (Supplementary Figure 161).*

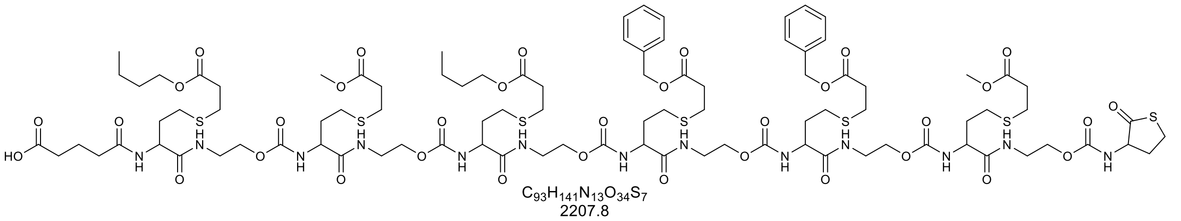

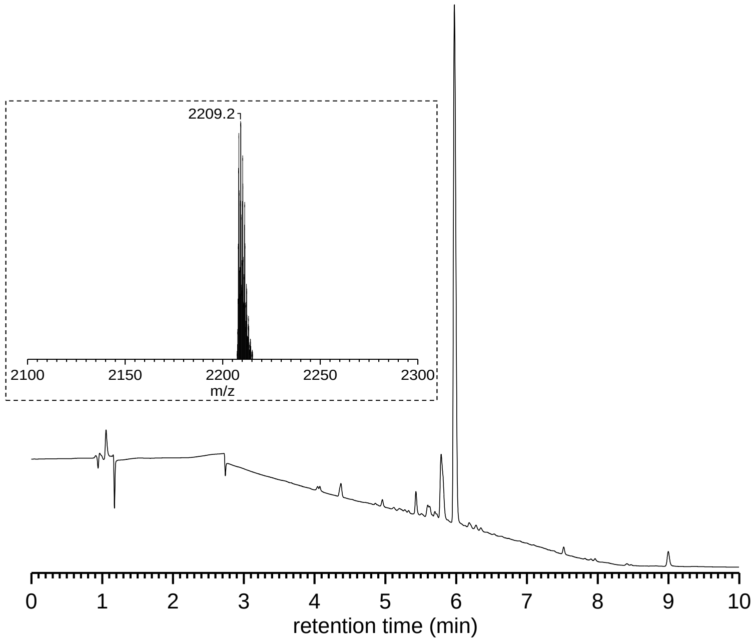

Supplementary Figure 160 | LC-ESI-MS analysis of **QR39**. Insert: ESI-MS-spectrum of dominant species (positive mode).

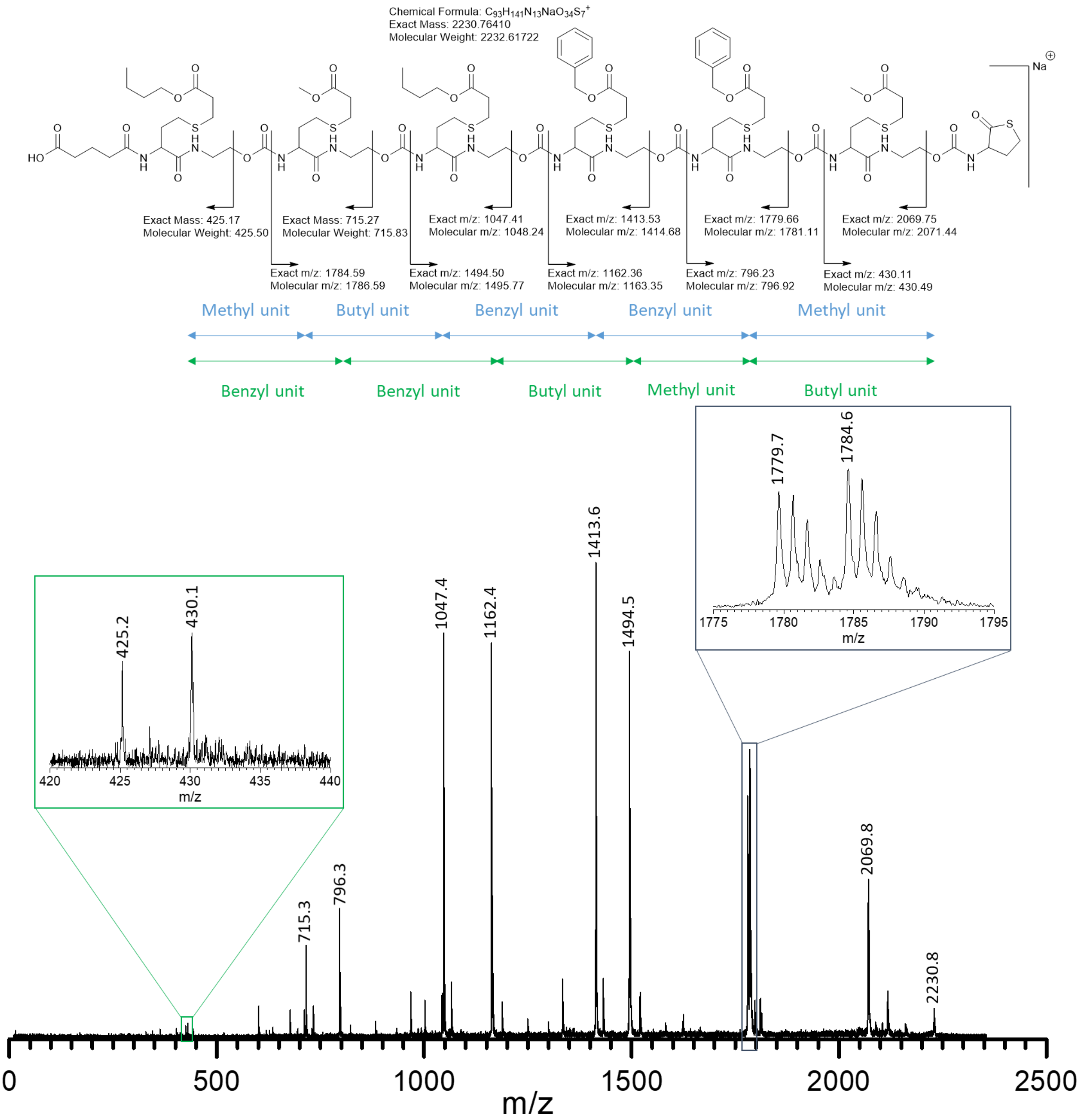

Supplementary Figure 161 | MALDI-MS/MS spectrum with peak assignment of **QR39**.

*Characterization of **QR40** using mass spectrometry (Supplementary Figure 162) and MALDI-MS/MS analysis (Supplementary Figure 163).*

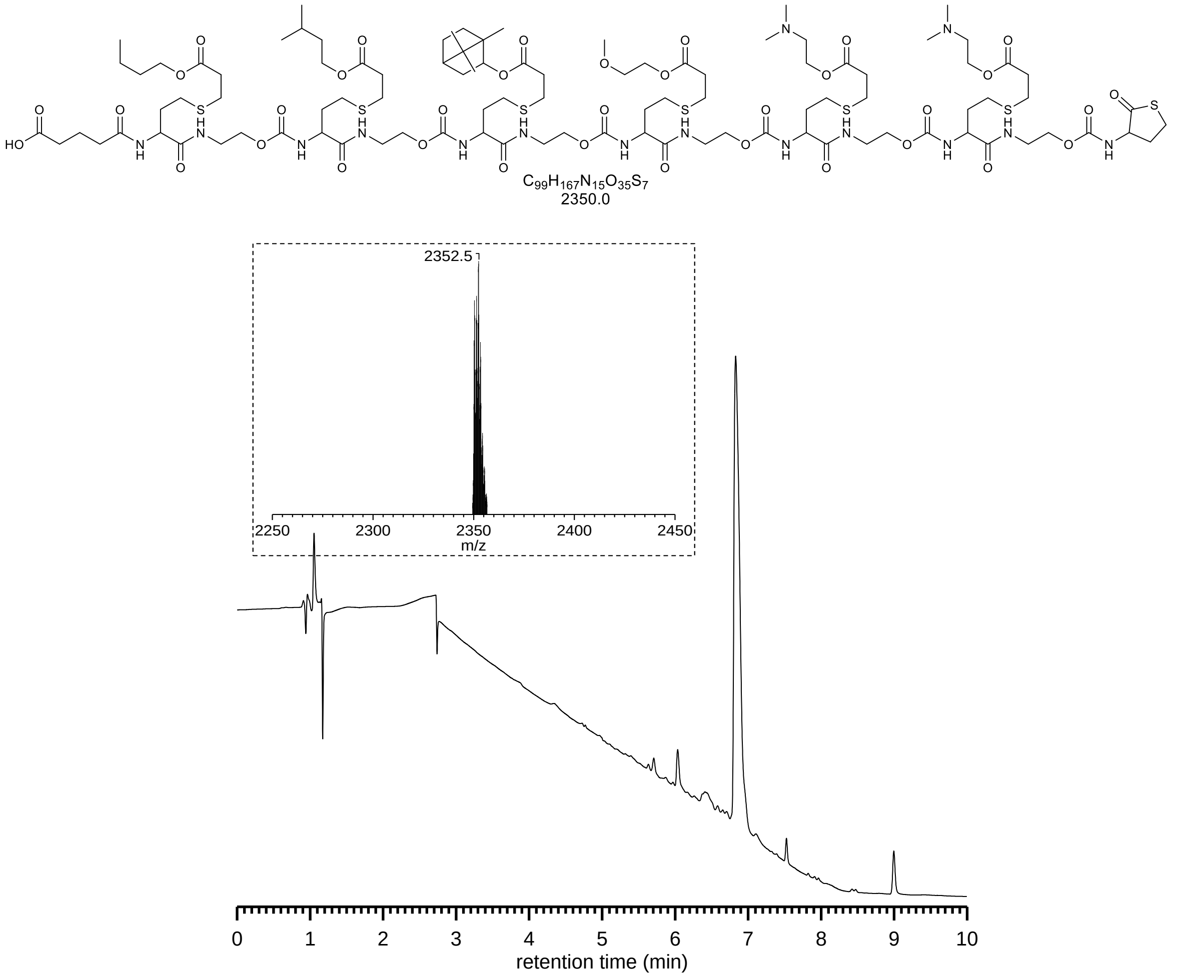

Supplementary Figure 162 | LC-ESI-MS analysis of **QR40**. Insert: ESI-MS-spectrum of dominant species (positive mode).

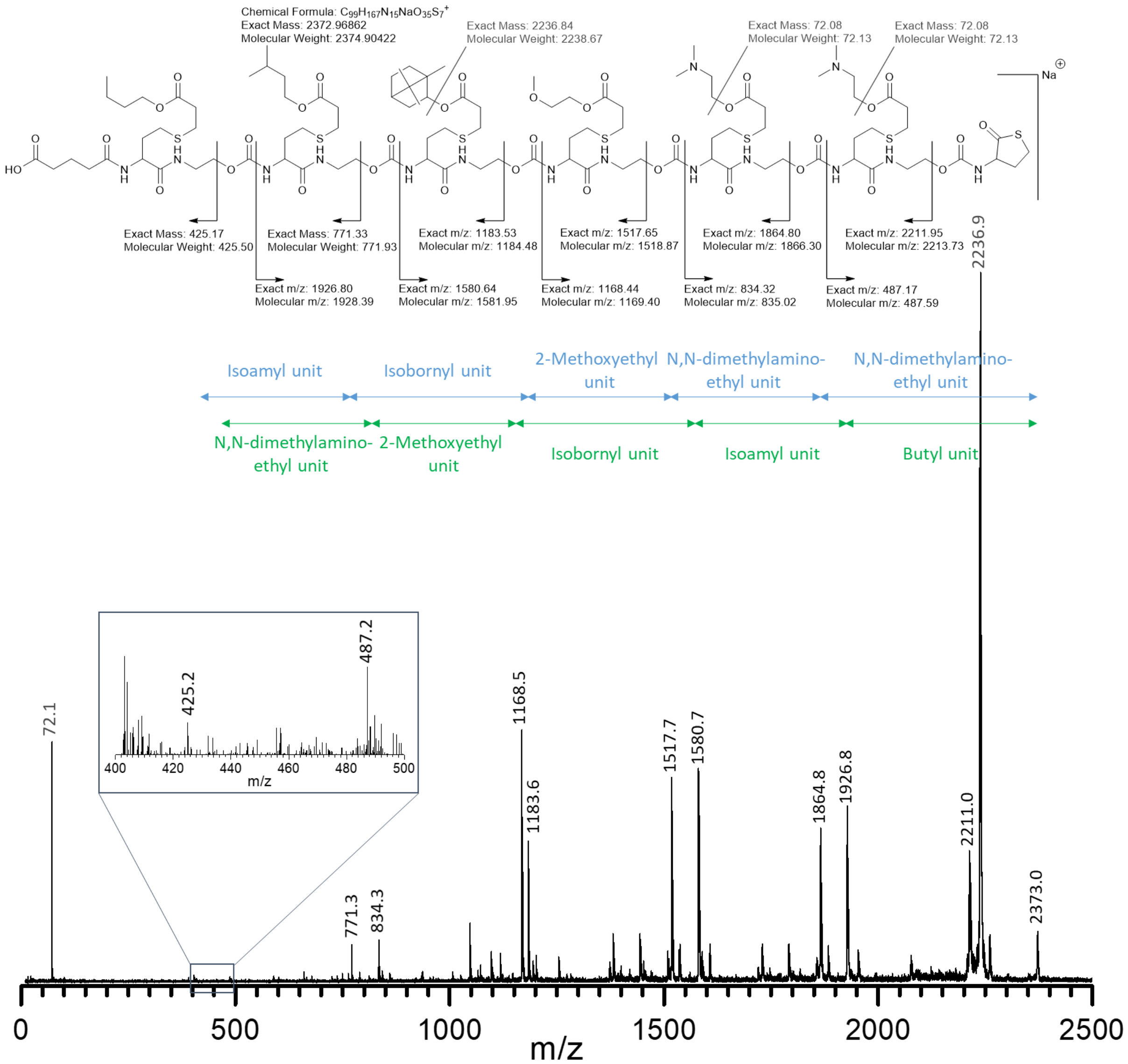

Supplementary Figure 163 | MALDI-MS/MS spectrum with peak assignment of **QR40**.

*Characterization of **QR41** using mass spectrometry (Supplementary Figure 164) and MALDI-MS/MS analysis (Supplementary Figure 165).*

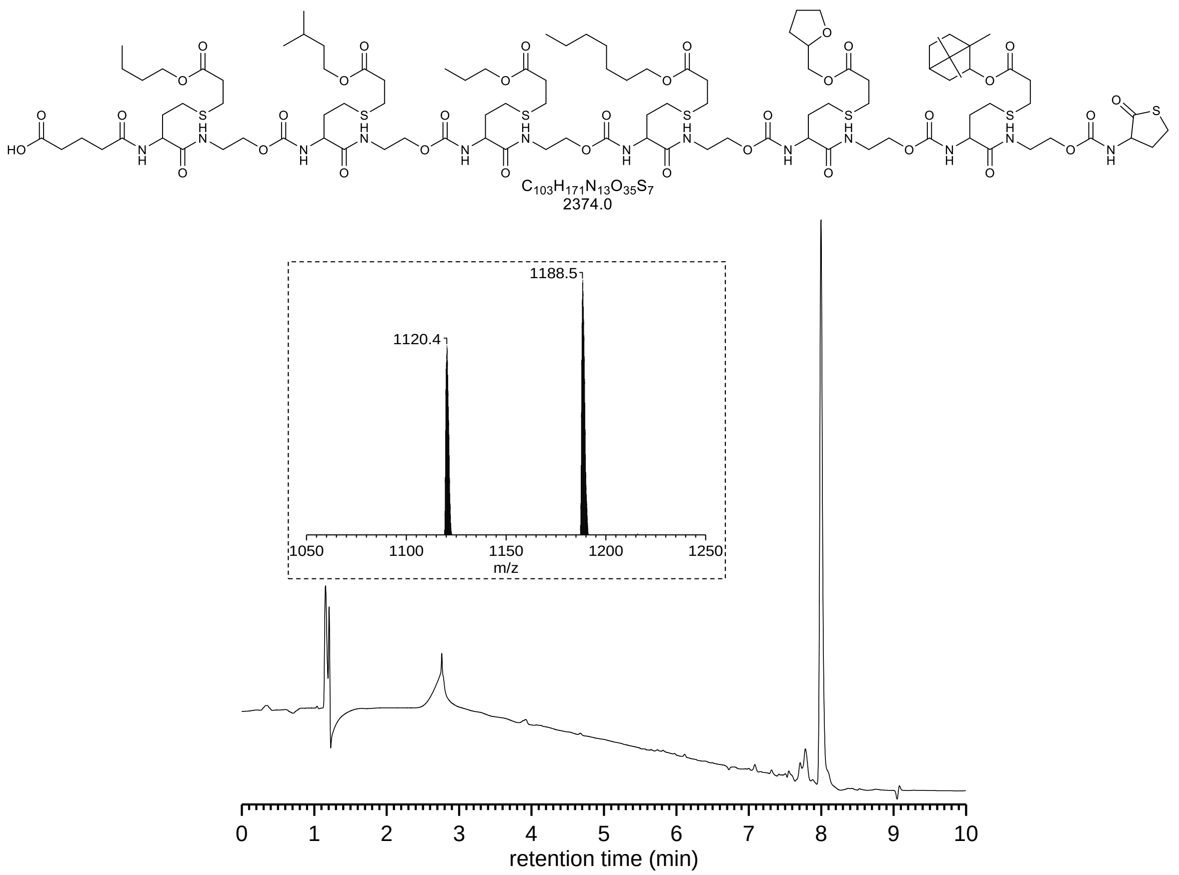

Supplementary Figure 164 | LC-ESI-MS analysis of **QR41**. Insert: ESI-MS-spectrum of dominant species (positive mode).

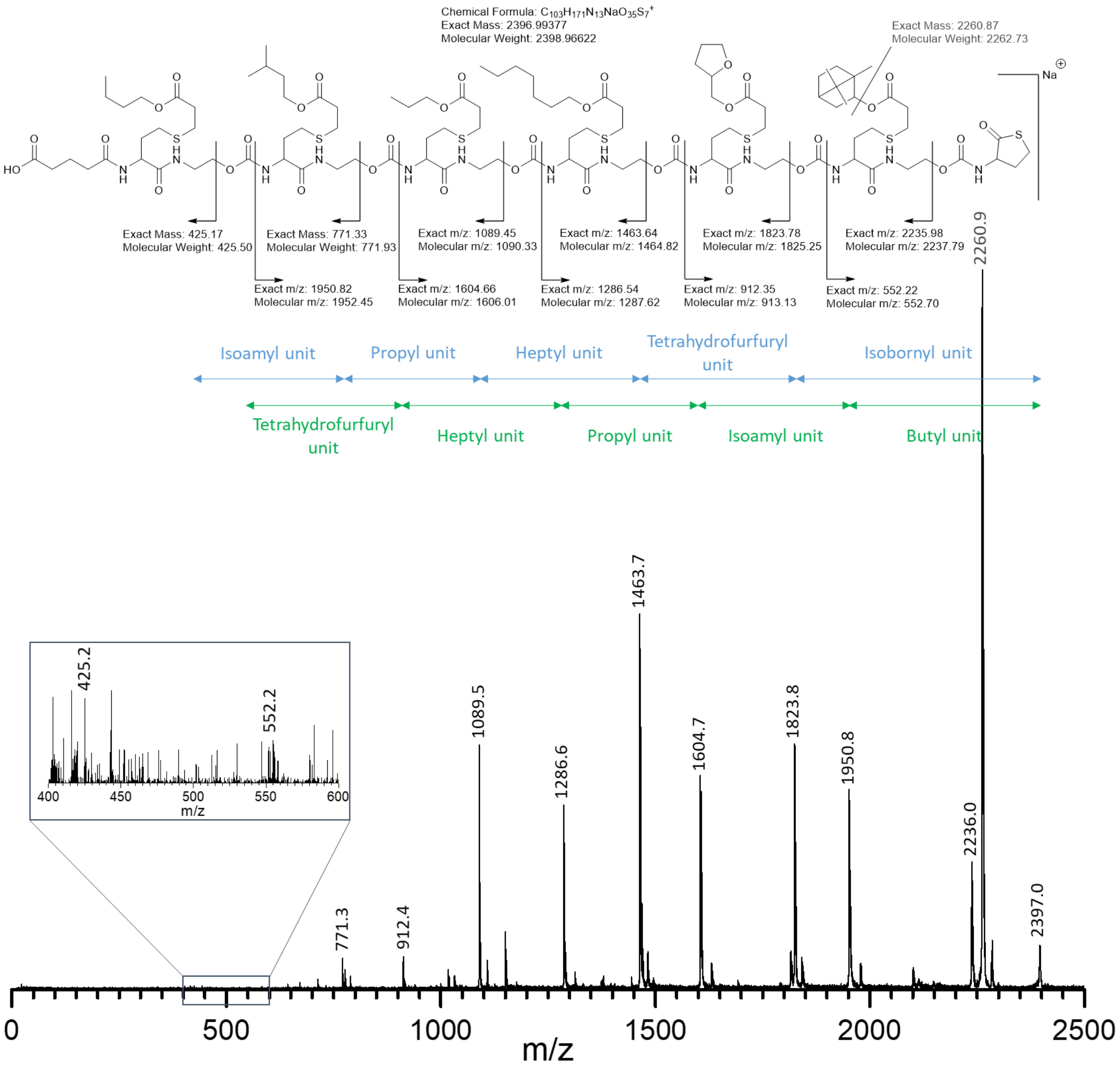

Supplementary Figure 165 | MALDI-MS/MS spectrum with peak assignment of **QR41**.

*Characterization of **QR42** using mass spectrometry (Supplementary Figure 166) and MALDI-MS/MS analysis (Supplementary Figure 167).*

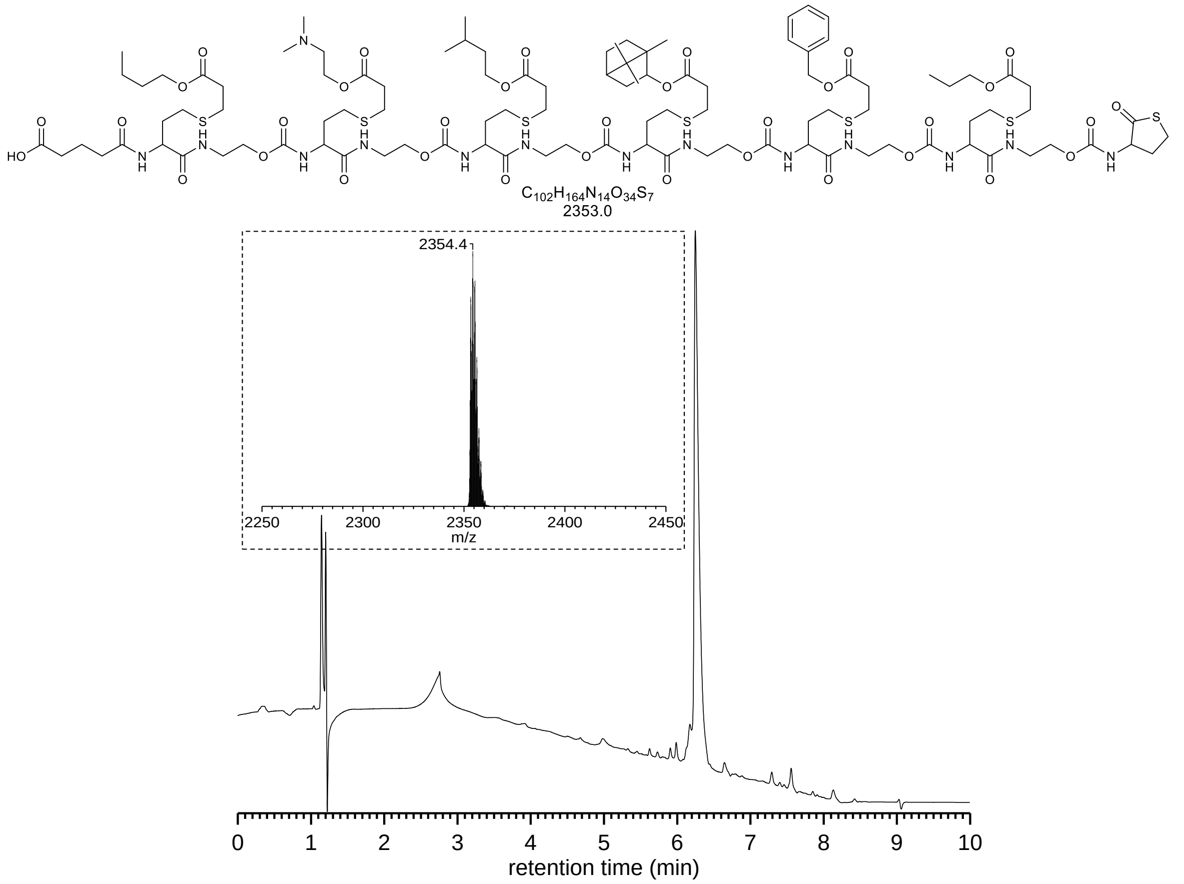

Supplementary Figure 166 | LC-ESI-MS analysis of **QR42**. Insert: ESI-MS-spectrum of dominant species (positive mode).

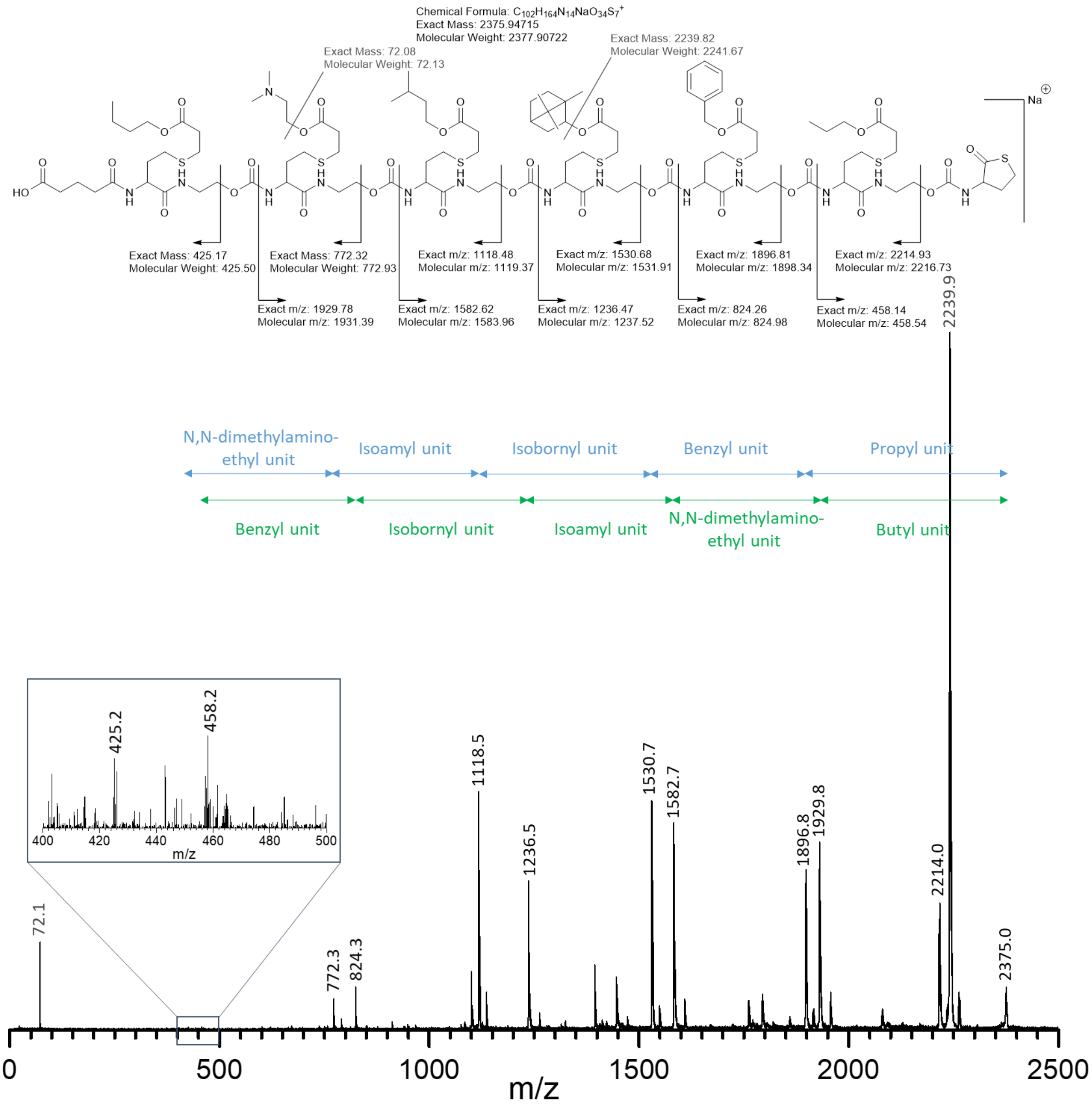

Supplementary Figure 167 | MALDI-MS/MS spectrum with peak assignment of **QR42**.

*Characterization of **QR43** using mass spectrometry (Supplementary Figure 168) and MALDI-MS/MS analysis (Supplementary Figure 169).*

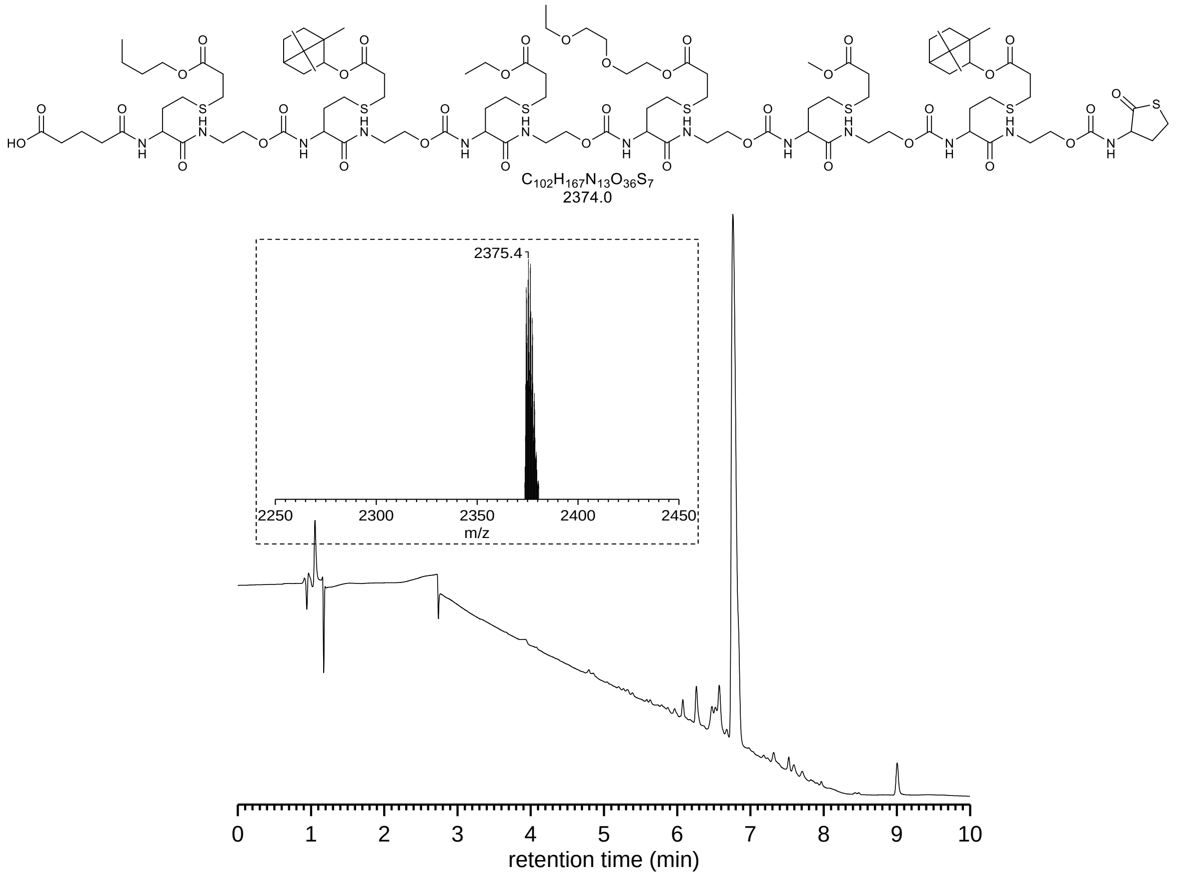

Supplementary Figure 168 | LC-ESI-MS analysis of **QR43**. Insert: ESI-MS-spectrum of dominant species (positive mode).

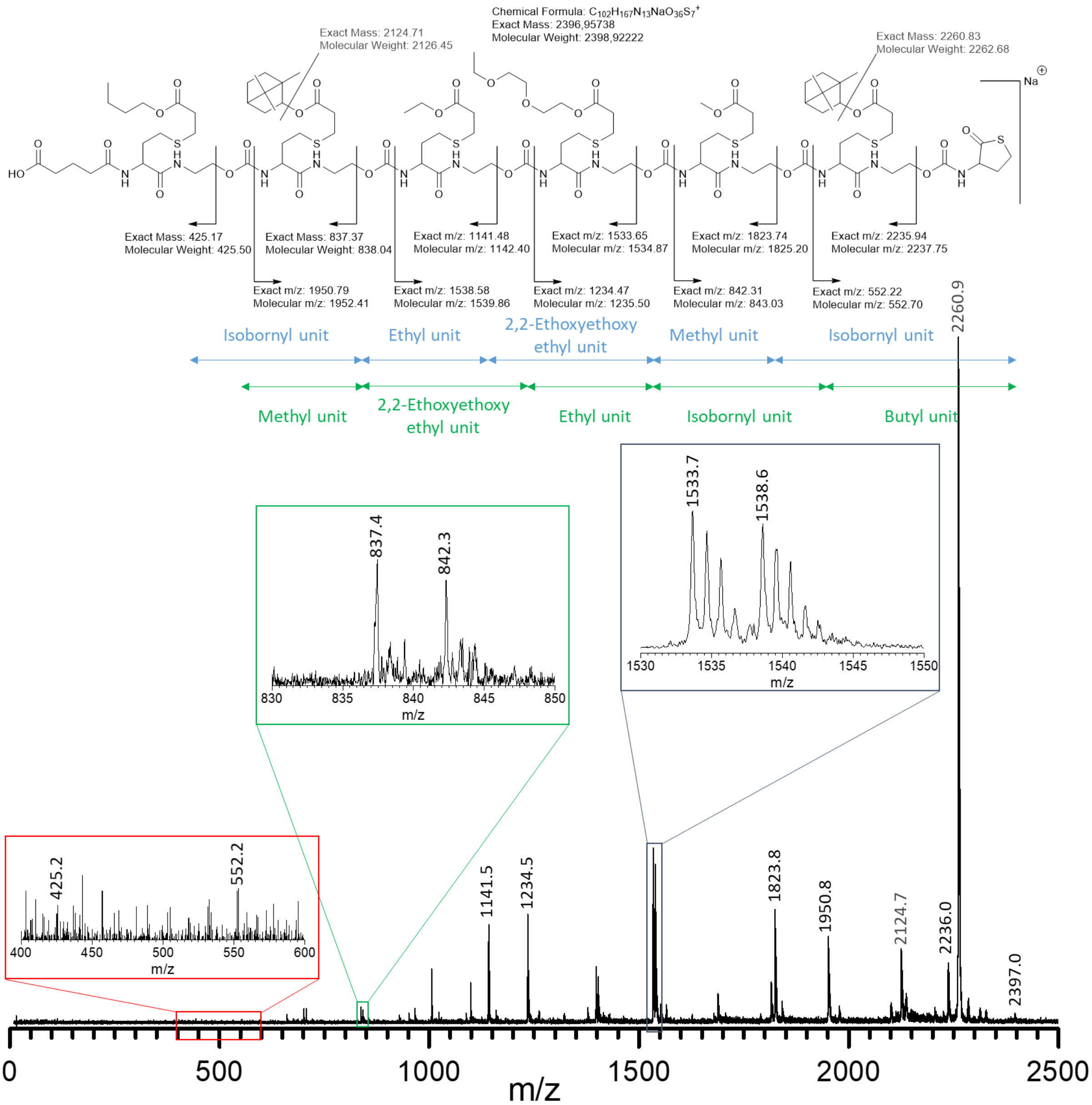

Supplementary Figure 169 | MALDI-MS/MS spectrum with peak assignment of **QR43**.

*Characterization of **QR44** using mass spectrometry (Supplementary Figure 170) and MALDI-MS/MS analysis (Supplementary Figure 171).*

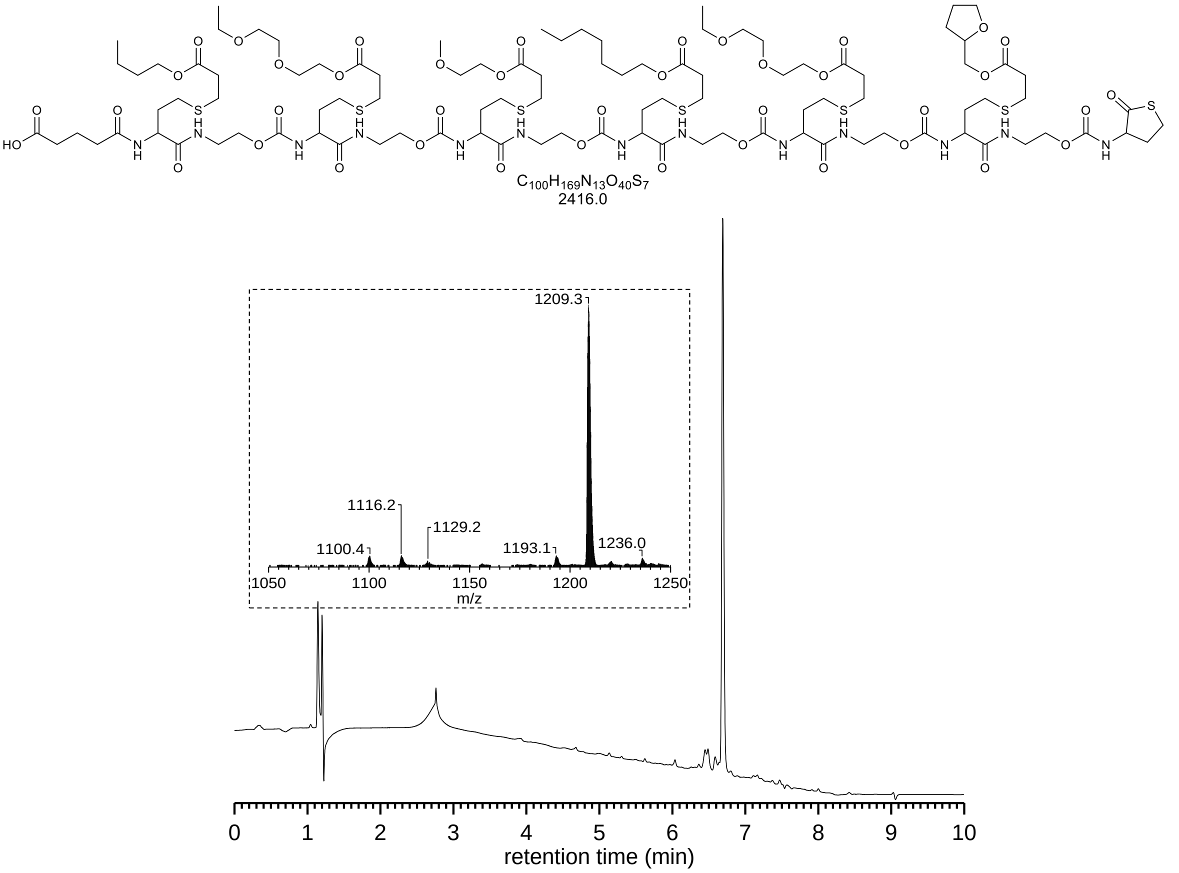

Supplementary Figure 170 | LC-ESI-MS analysis of **QR44**. Insert: ESI-MS-spectrum of dominant species (positive mode).

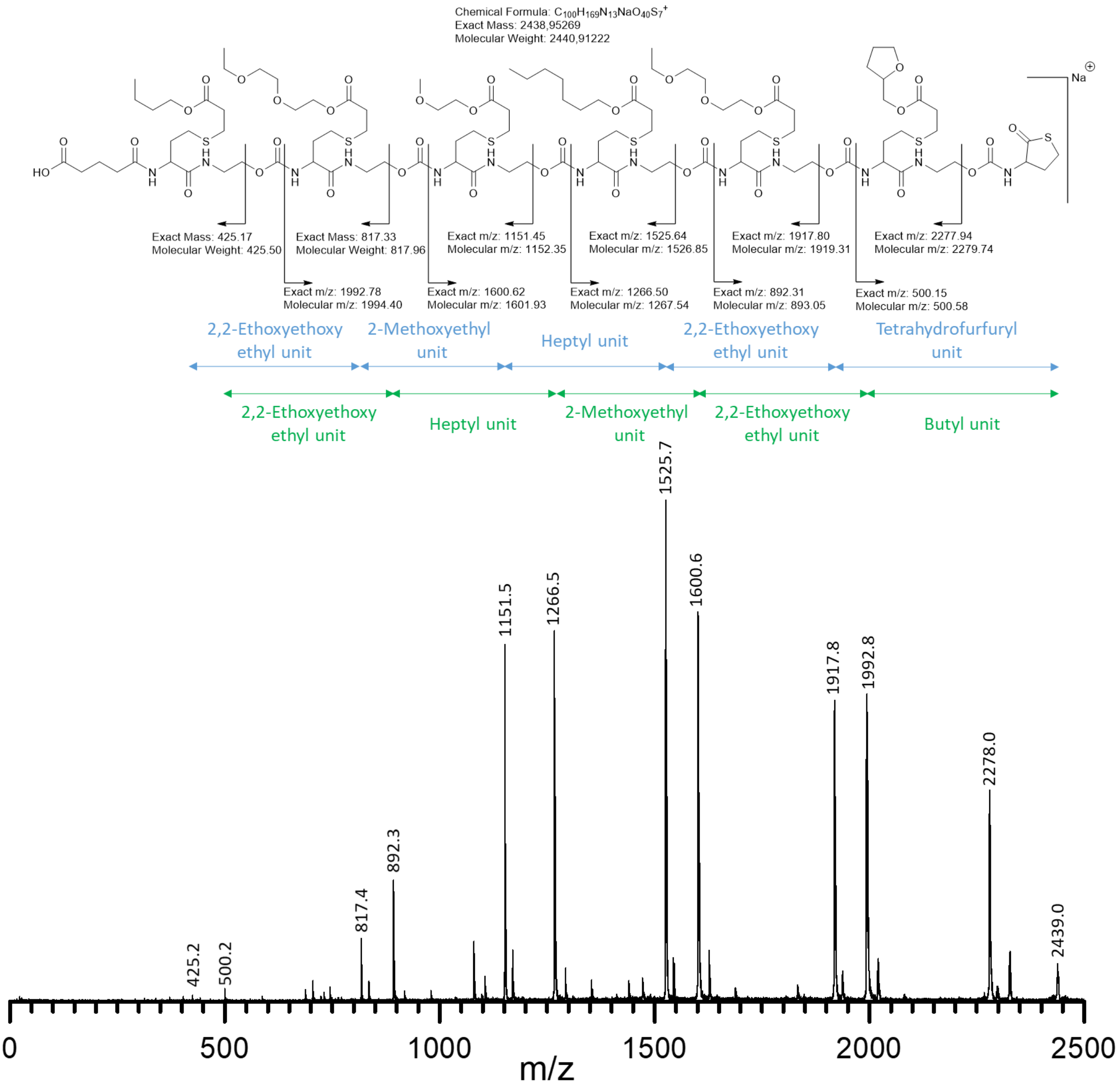

Supplementary Figure 171 | MALDI-MS/MS spectrum with peak assignment of **QR44**.

*Characterization of **QR45** using mass spectrometry (Supplementary Figure 172) and MALDI-MS/MS analysis (Supplementary Figure 173).*

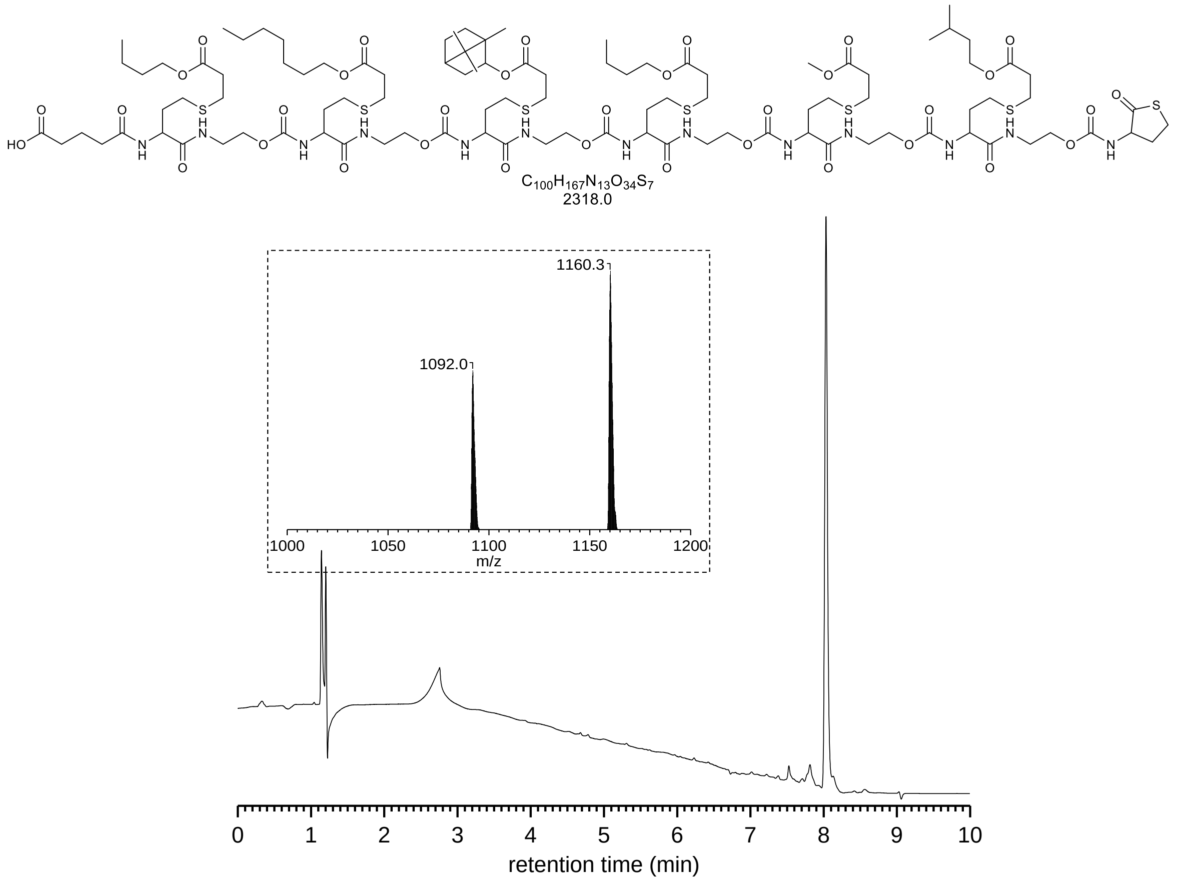

Supplementary Figure 172 | LC-ESI-MS analysis of **QR45**. Insert: ESI-MS-spectrum of dominant species (positive mode).

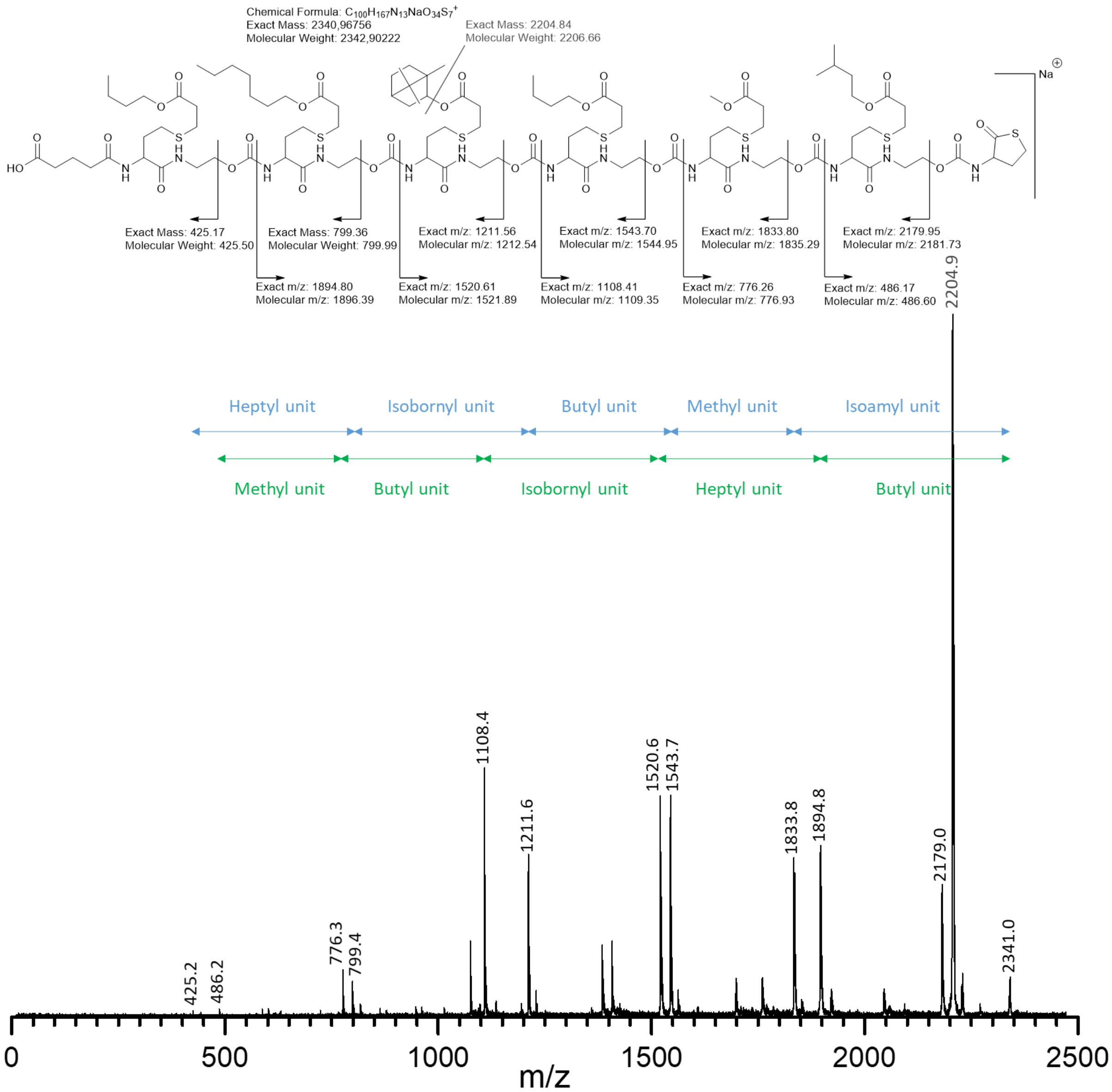

Supplementary Figure 173 | MALDI-MS/MS spectrum with peak assignment of **QR45**.

*Characterization of **QR46** using mass spectrometry (Supplementary Figure 174) and MALDI-MS/MS analysis (Supplementary Figure 175).*

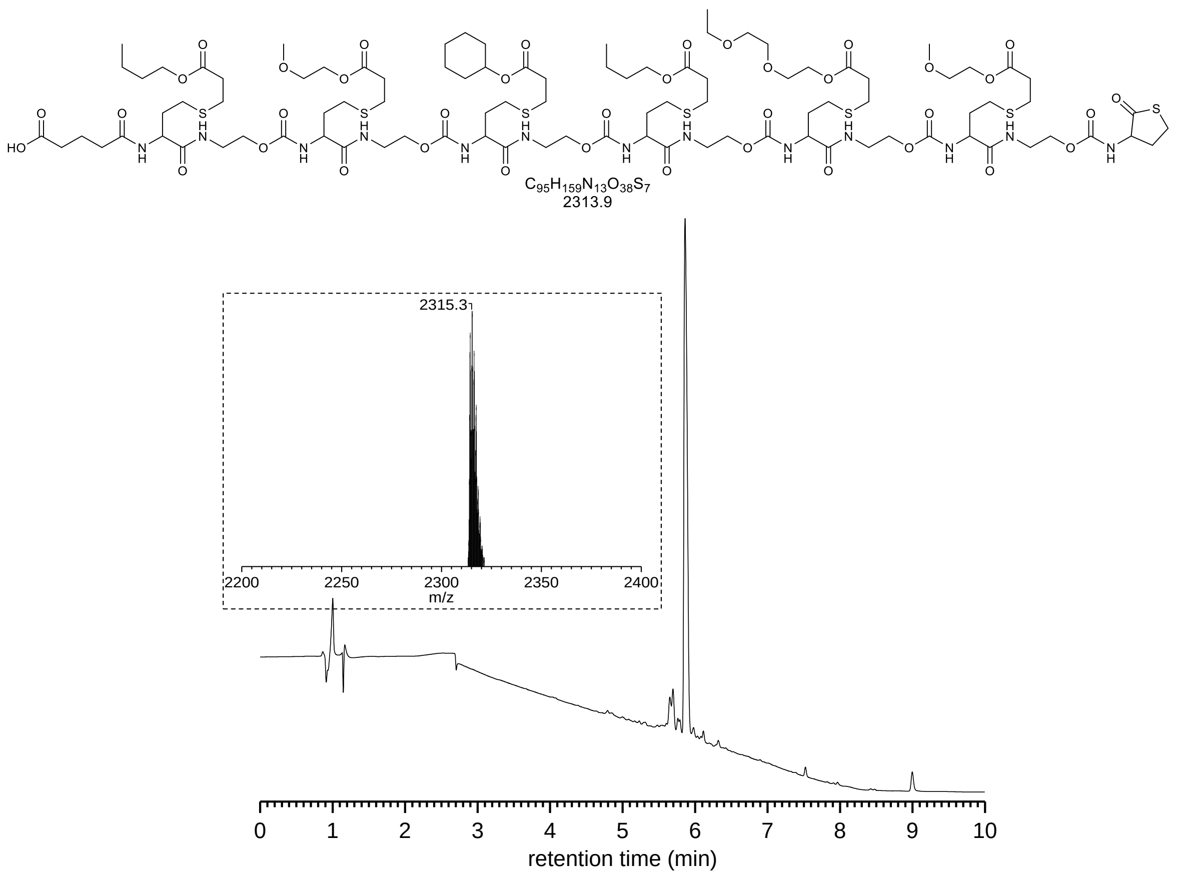

Supplementary Figure 174 | LC-ESI-MS analysis of **QR46**. Insert: ESI-MS-spectrum of dominant species (positive mode).

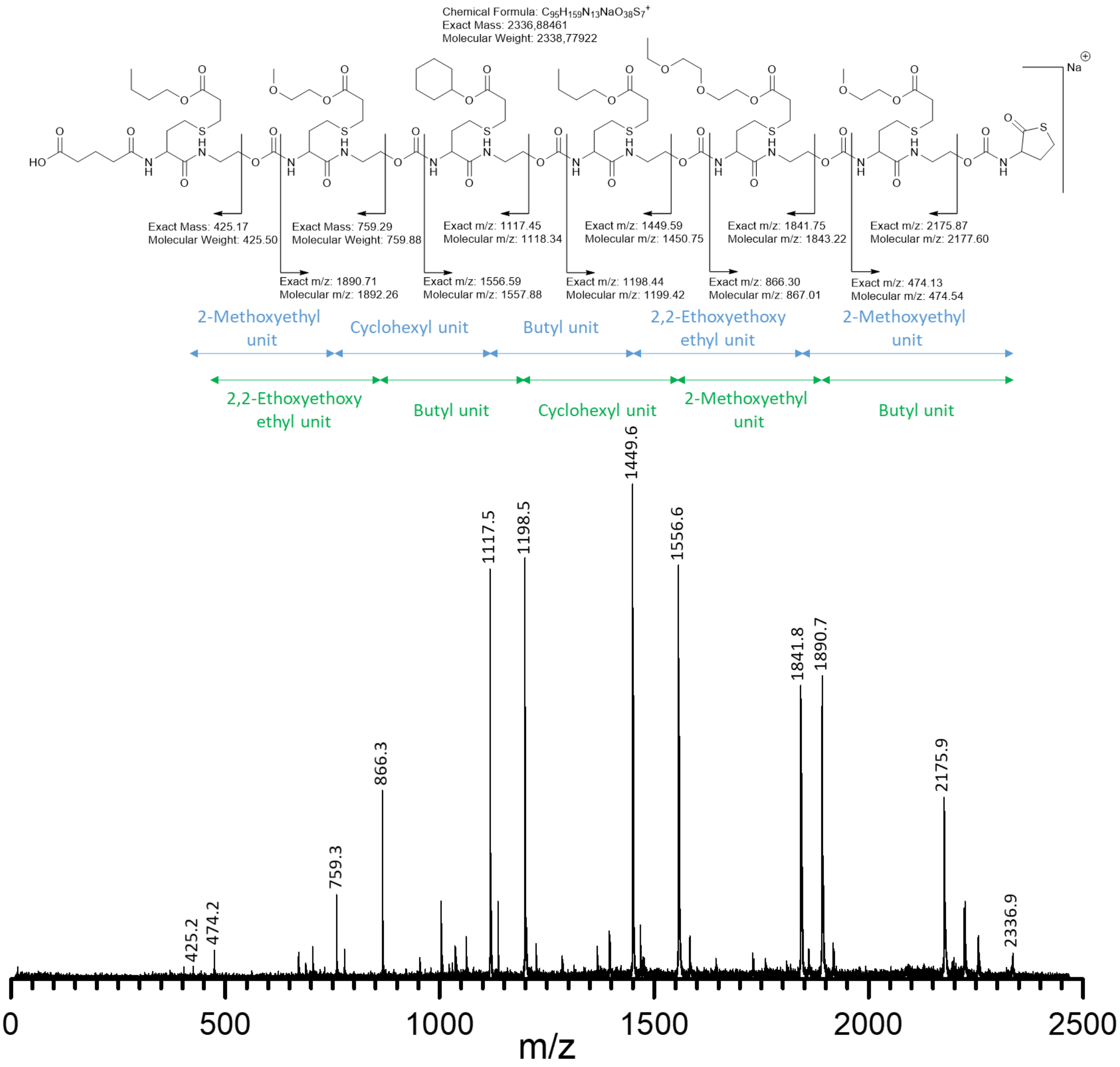

Supplementary Figure 175 | MALDI-MS/MS spectrum with peak assignment of **QR46**.

*Characterization of **QR47** using mass spectrometry (Supplementary Figure 176) and MALDI-MS/MS analysis (Supplementary Figure 177).*

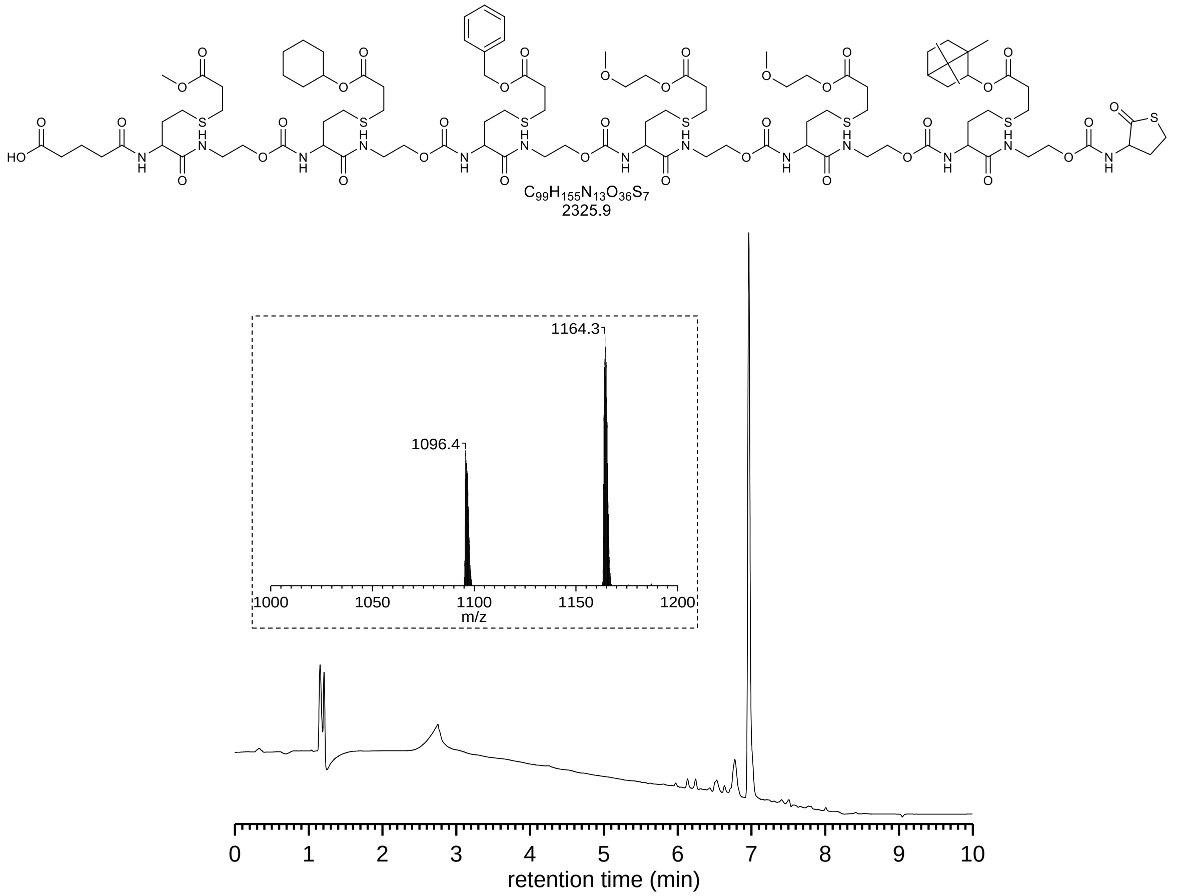

Supplementary Figure 176 | LC-ESI-MS analysis of **QR47**. Insert: ESI-MS-spectrum of dominant species (positive mode).

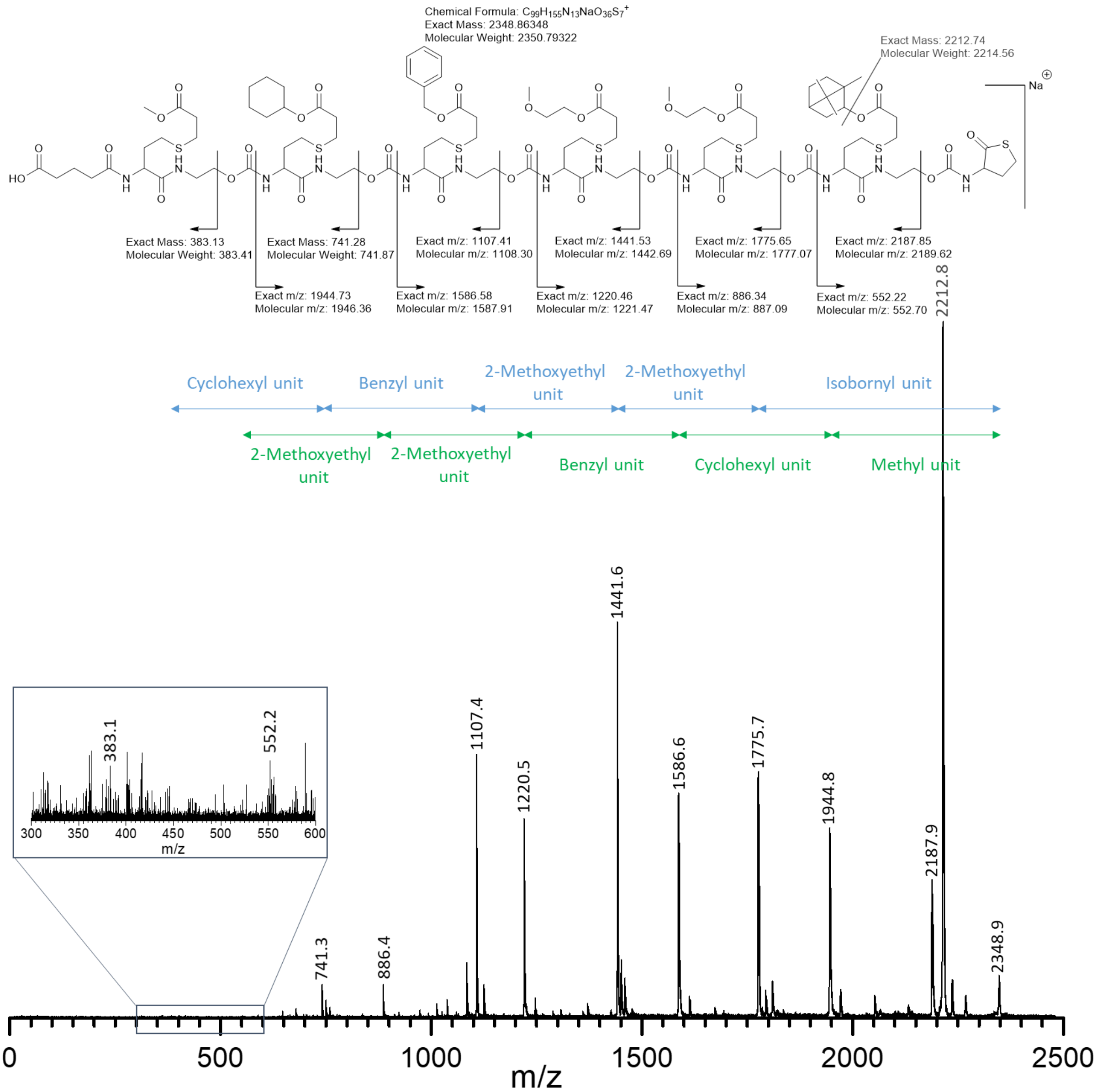

Supplementary Figure 177 | MALDI-MS/MS spectrum with peak assignment of **QR47**.

*Characterization of **QR48** using mass spectrometry (Supplementary Figure 178) and MALDI-MS/MS analysis (Supplementary Figure 179).*

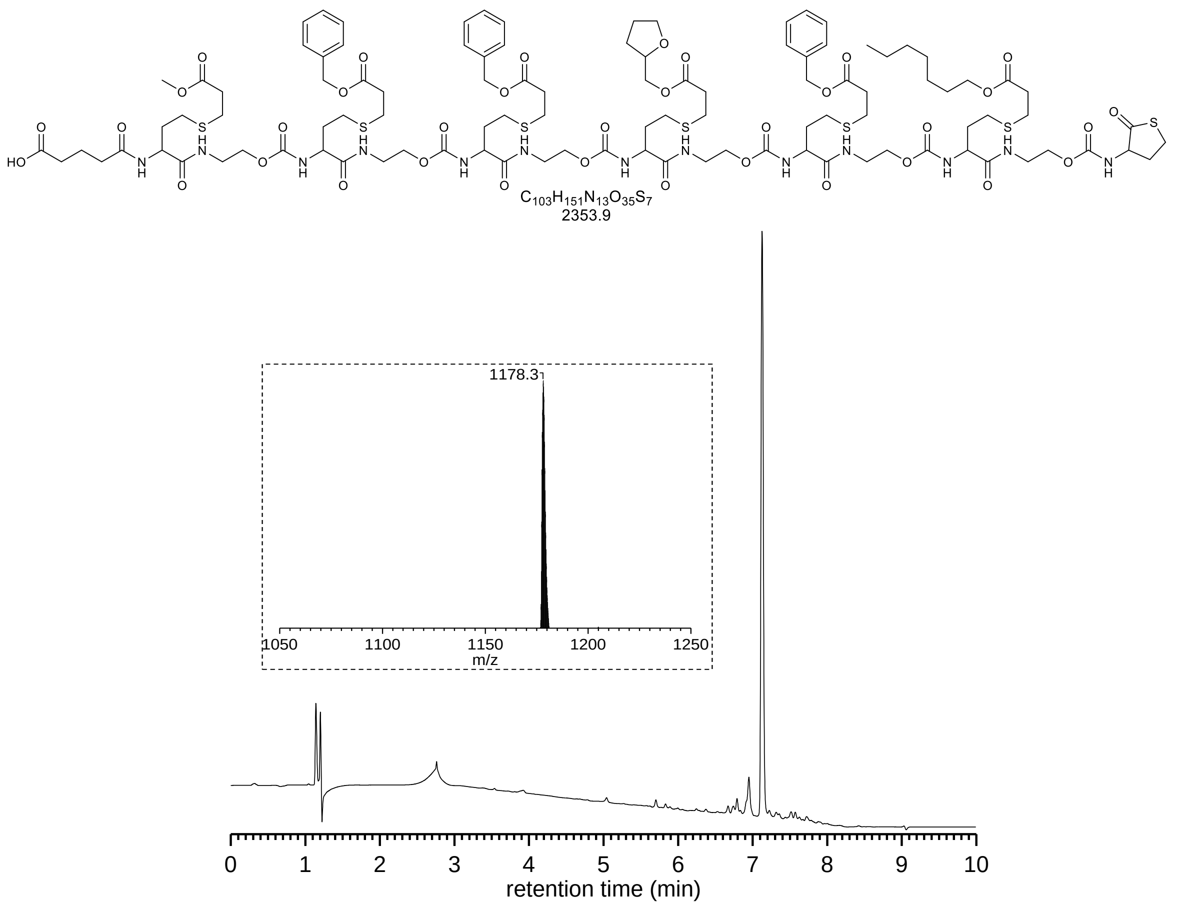

Supplementary Figure 178 | LC-ESI-MS analysis of **QR48**. Insert: ESI-MS-spectrum of dominant species (positive mode).

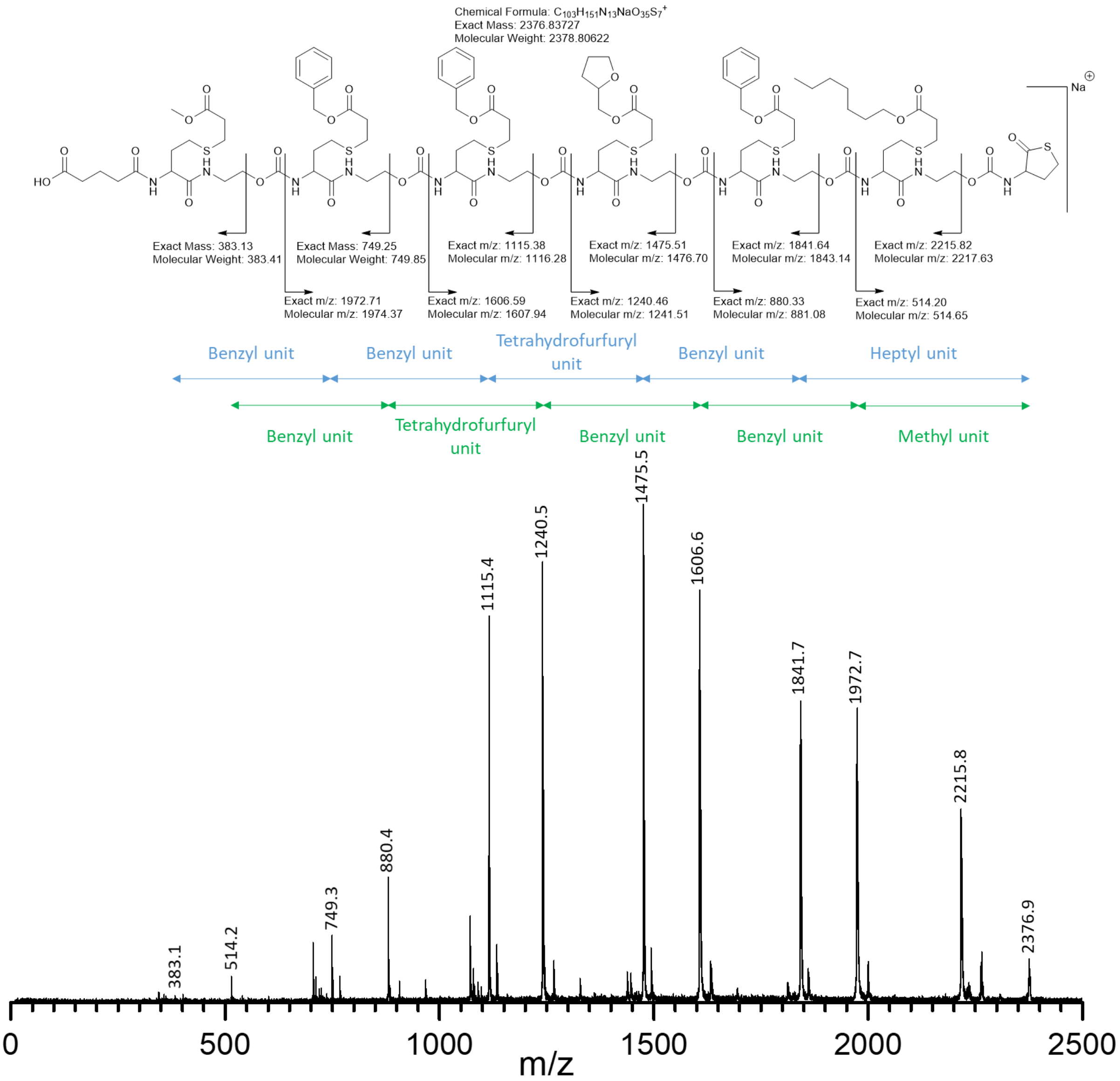

Supplementary Figure 179 | MALDI-MS/MS spectrum with peak assignment of **QR48**.

*Characterization of **QR49** using mass spectrometry (Supplementary Figure 180) and MALDI-MS/MS analysis (Supplementary Figure 181).*

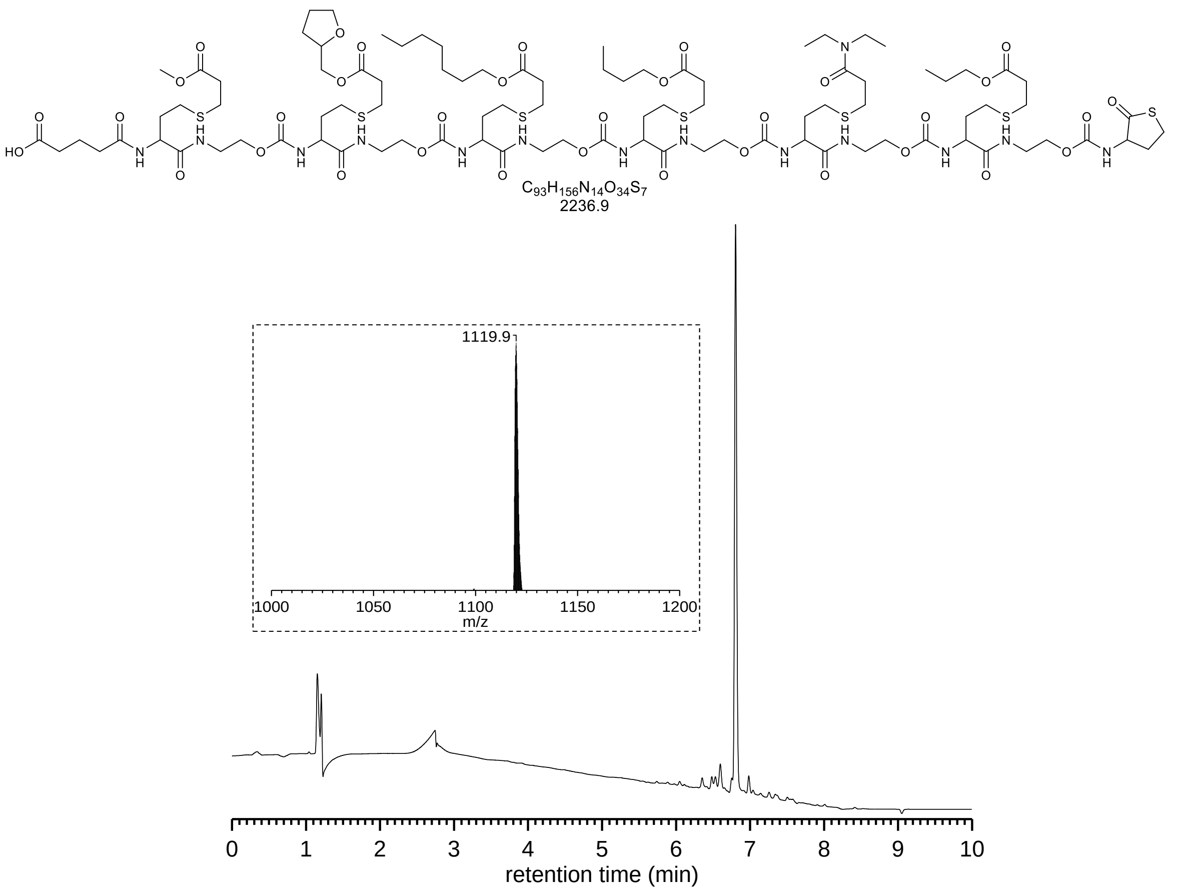

Supplementary Figure 180 | LC-ESI-MS analysis of **QR49**. Insert: ESI-MS-spectrum of dominant species (positive mode).

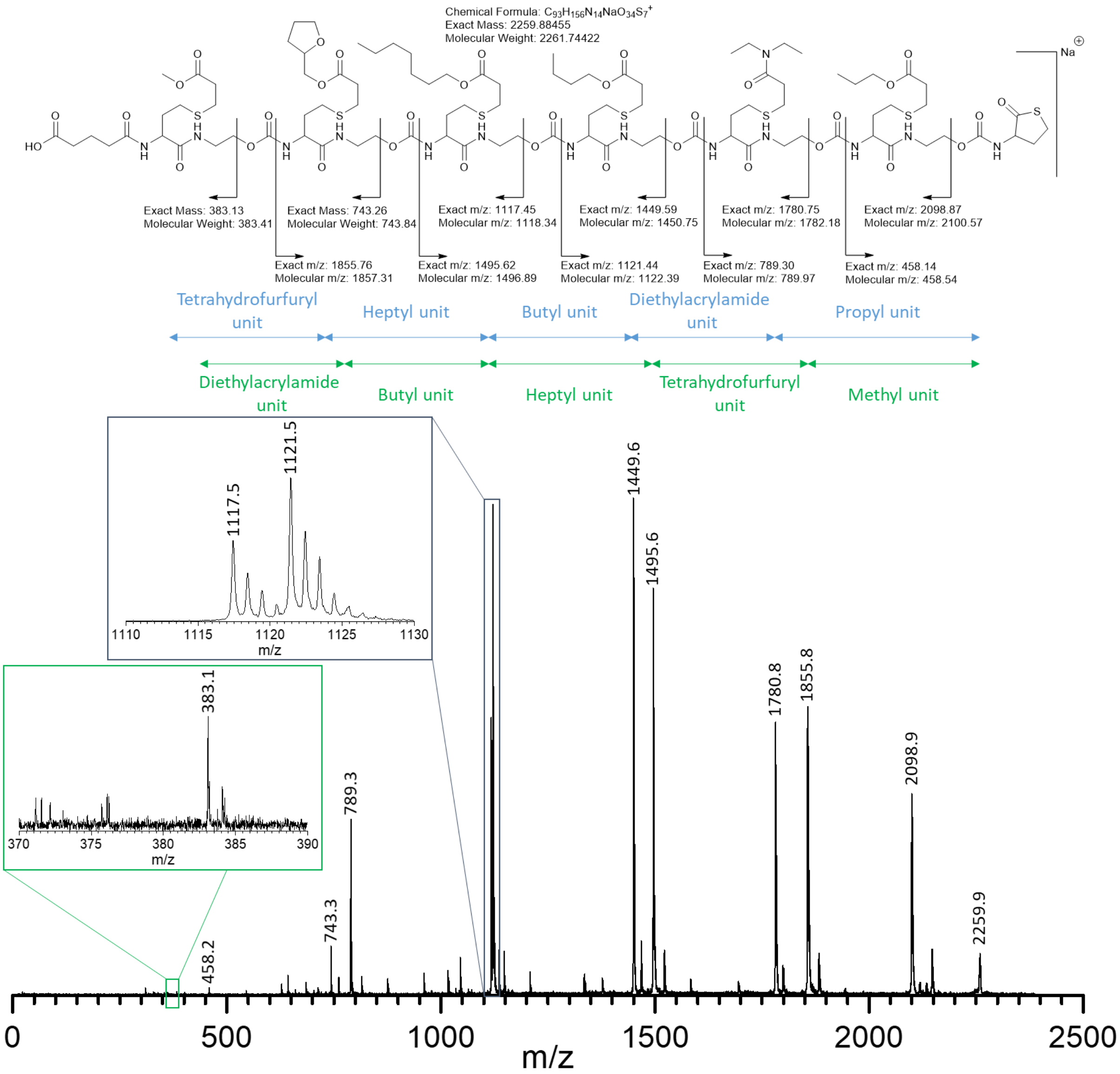

Supplementary Figure 181 | MALDI-MS/MS spectrum with peak assignment of **QR49**.

*Characterization of **QR50** using mass spectrometry (Supplementary Figure 182) and MALDI-MS/MS analysis (Supplementary Figure 183).*

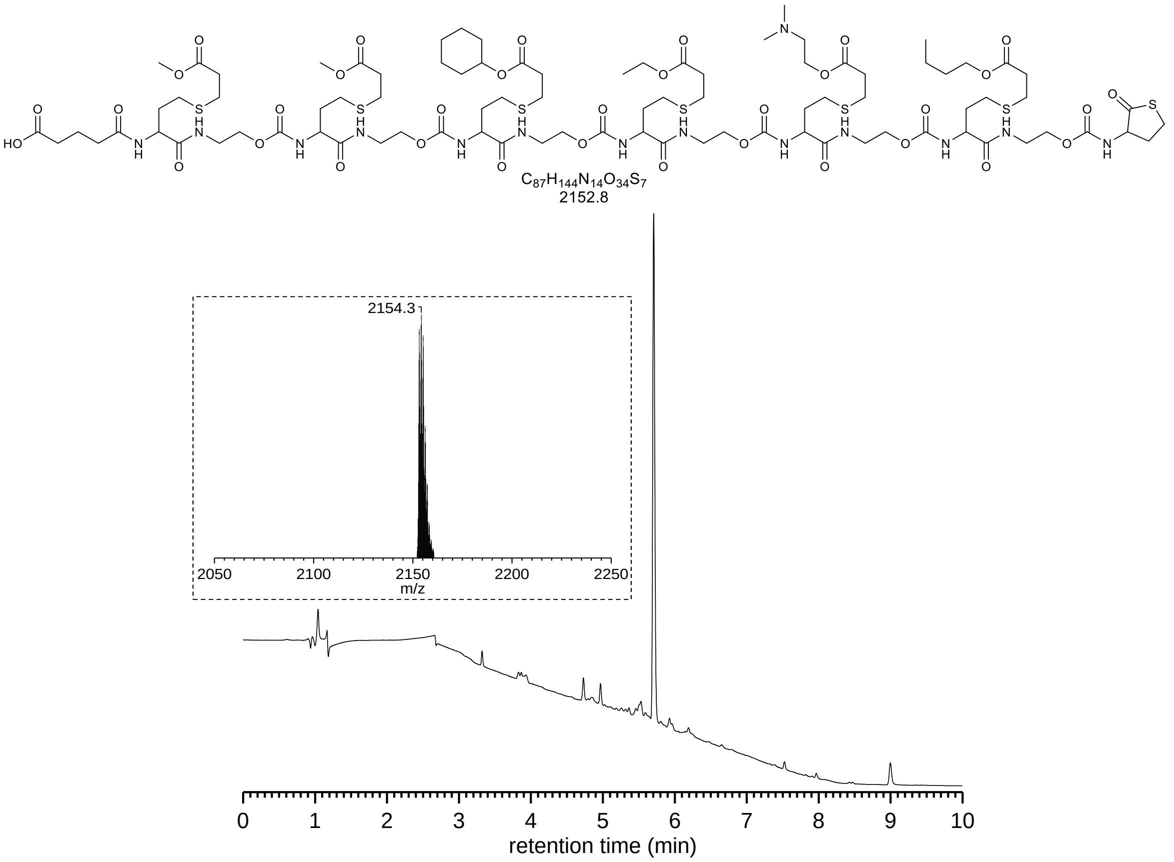

Supplementary Figure 182 | LC-ESI-MS analysis of **QR50**. Insert: ESI-MS-spectrum of dominant species (positive mode).

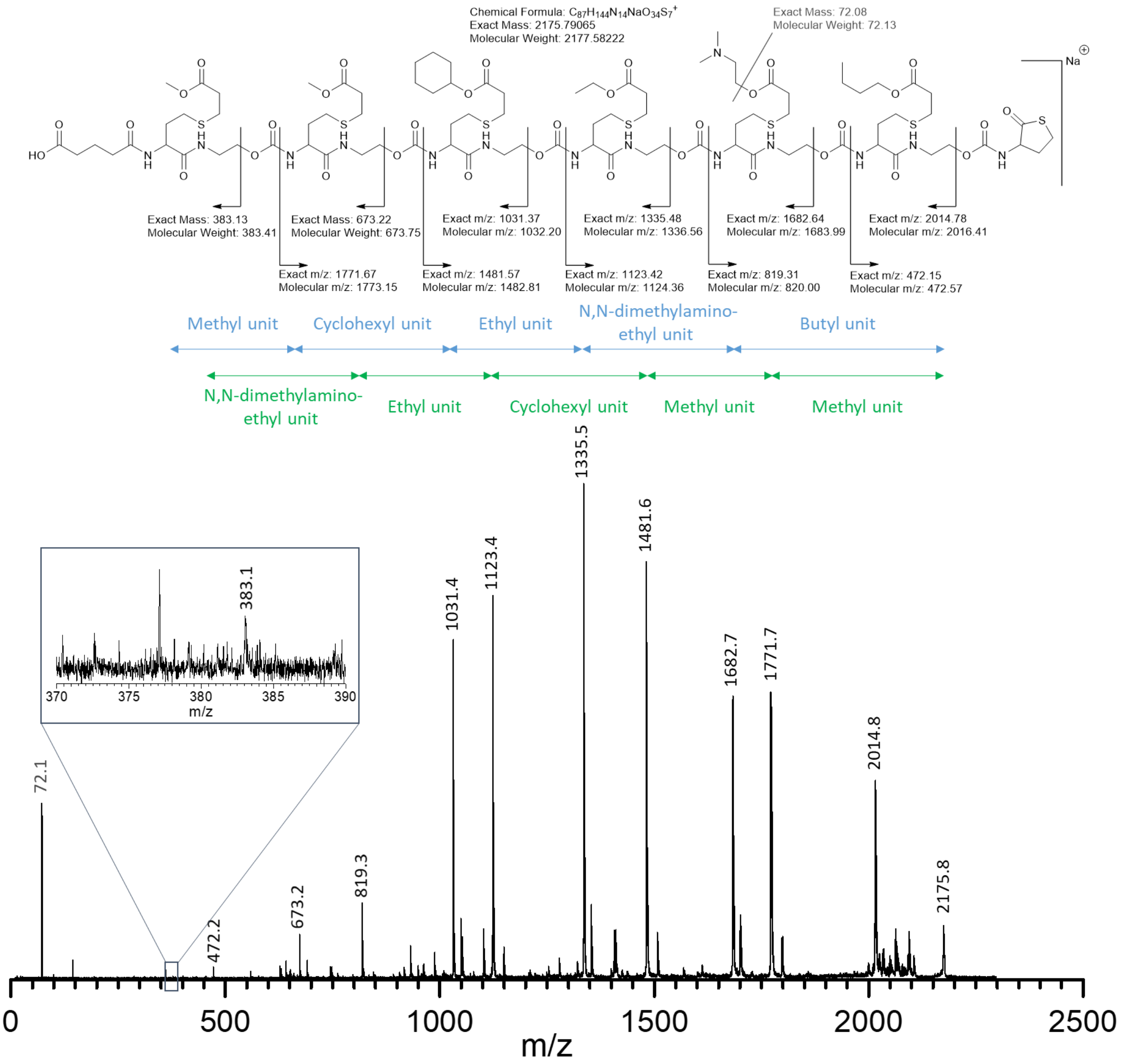

Supplementary Figure 183 | MALDI-MS/MS spectrum with peak assignment of **QR50**.

Characterization of ***QR51*** *using mass spectrometry (Supplementary Figure 184) and MALDI-MS/MS analysis (Supplementary Figure 185).*

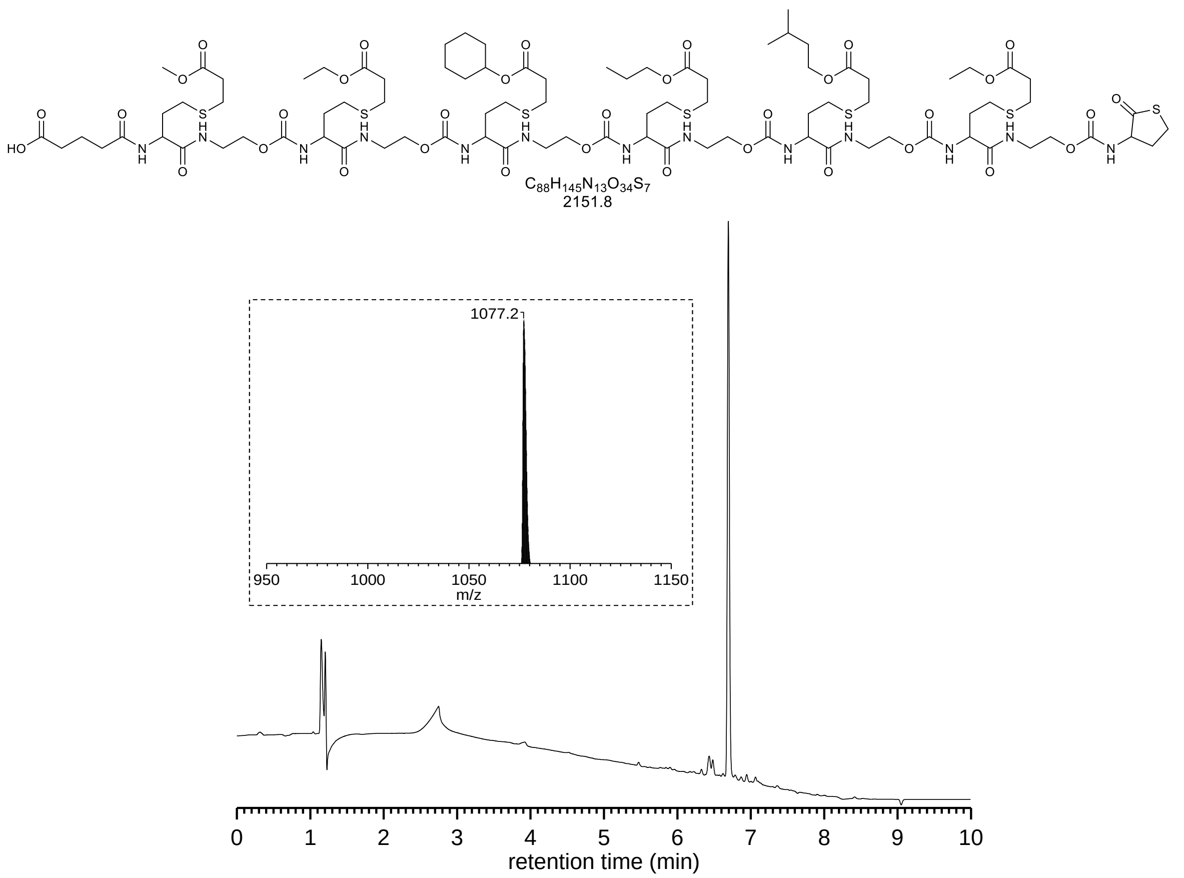

Supplementary Figure 184 | LC-ESI-MS analysis of **QR51**. Insert: ESI-MS-spectrum of dominant species (positive mode).

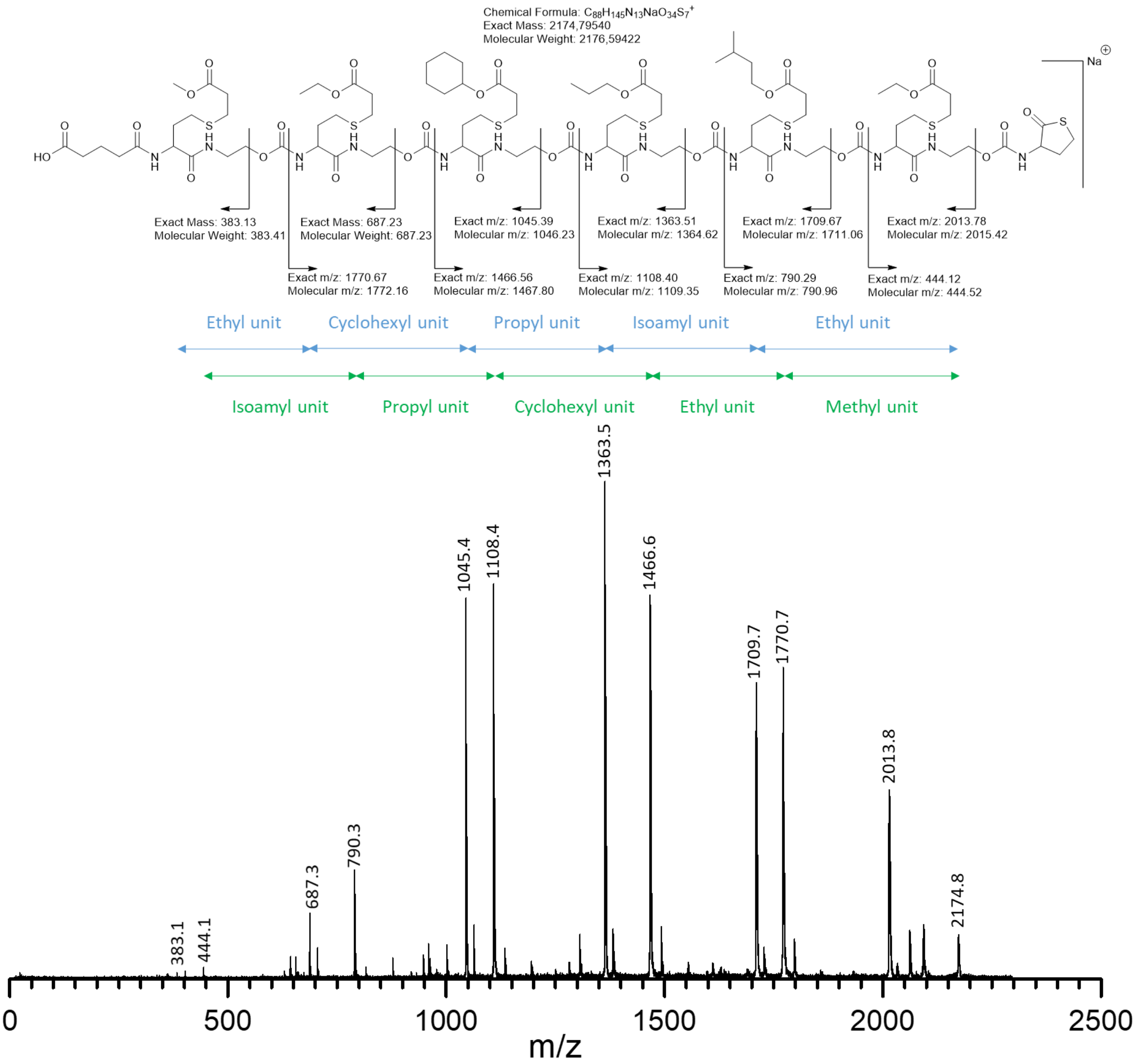

Supplementary Figure 185 | MALDI-MS/MS spectrum with peak assignment of **QR51**.

*Characterization of **QR52** using mass spectrometry (Supplementary Figure 186) and MALDI-MS/MS analysis (Supplementary Figure 187).*

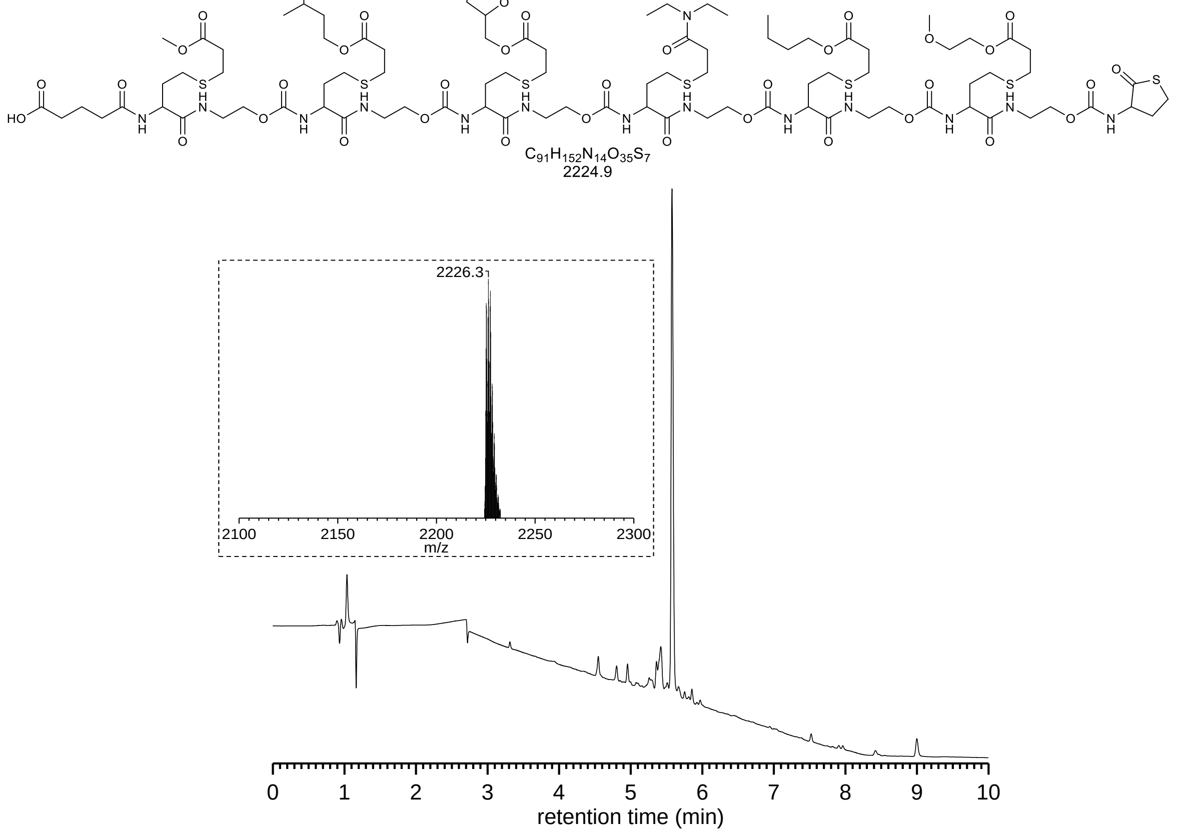

Supplementary Figure 186 | LC-ESI-MS analysis of **QR52**. Insert: ESI-MS-spectrum of dominant species (positive mode).

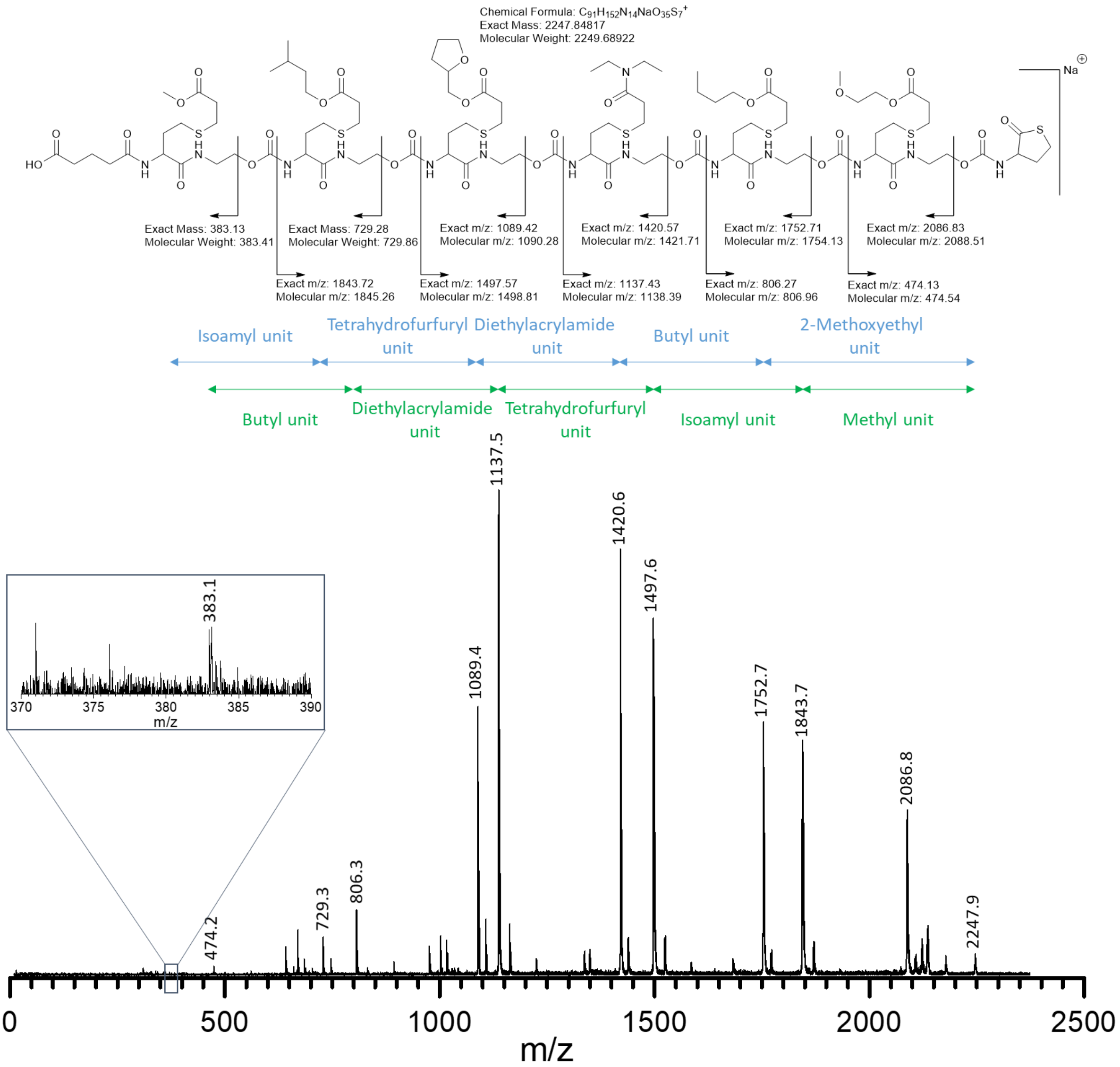

Supplementary Figure 187 | MALDI-MS/MS spectrum with peak assignment of **QR52**.

*Characterization of **QR53** using mass spectrometry (Supplementary Figure 188) and MALDI-MS/MS analysis (Supplementary Figure 189).*

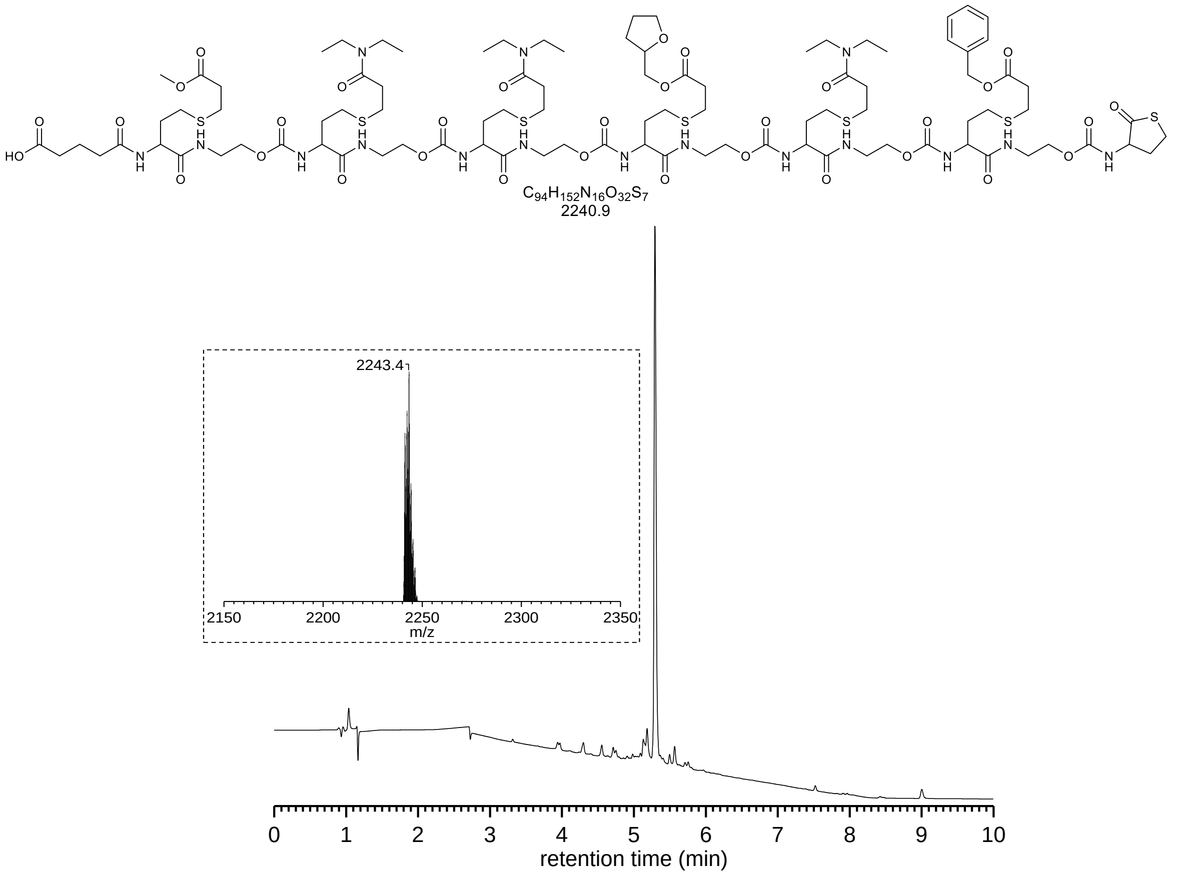

Supplementary Figure 188 | LC-ESI-MS analysis of **QR53**. Insert: ESI-MS-spectrum of dominant species (positive mode).

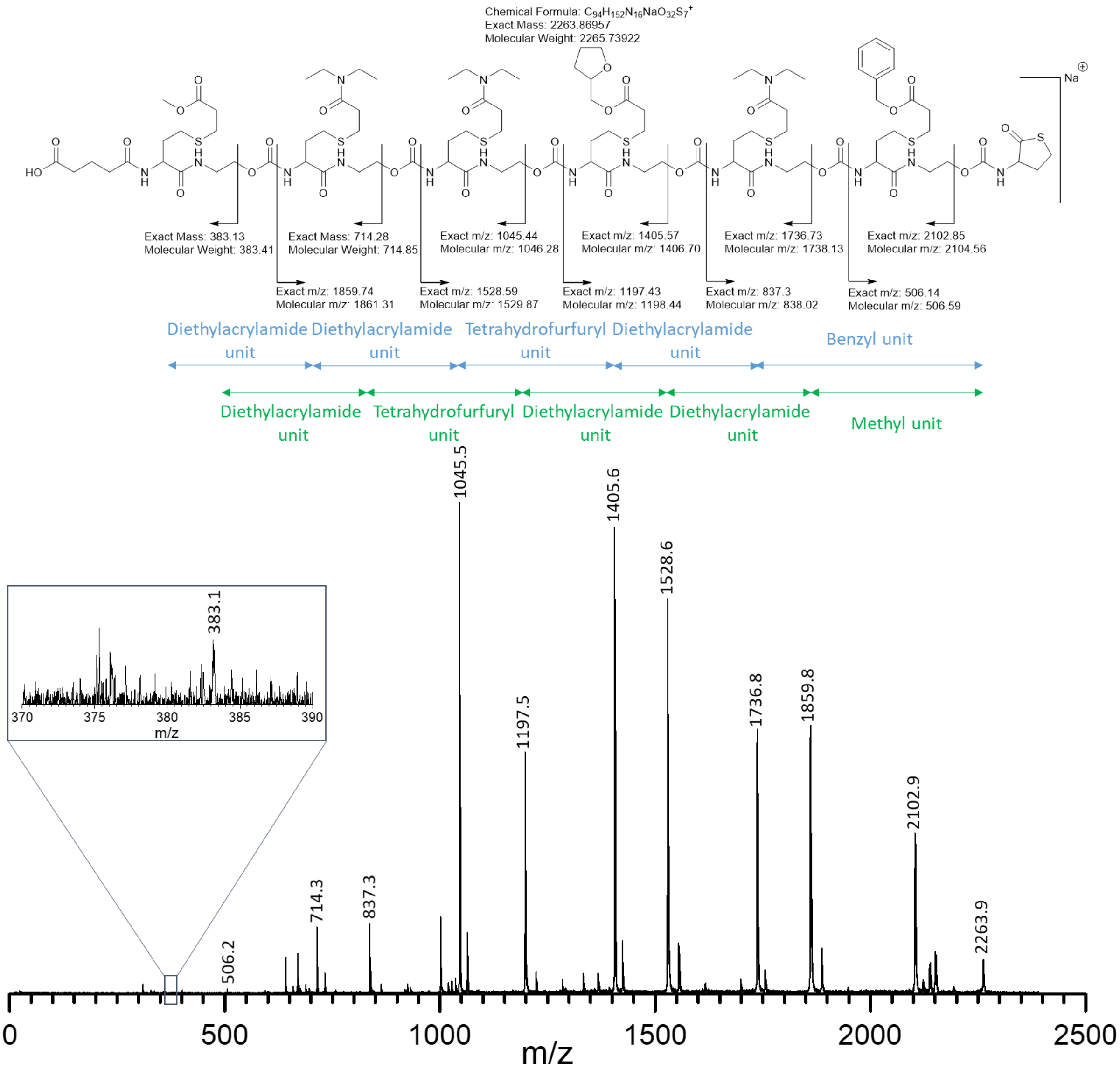

Supplementary Figure 189 | MALDI-MS/MS spectrum with peak assignment of **QR53**.

*Characterization of **QR54** using mass spectrometry (Supplementary Figure 190) and MALDI-MS/MS analysis* (Supplementary *Figure 191).*

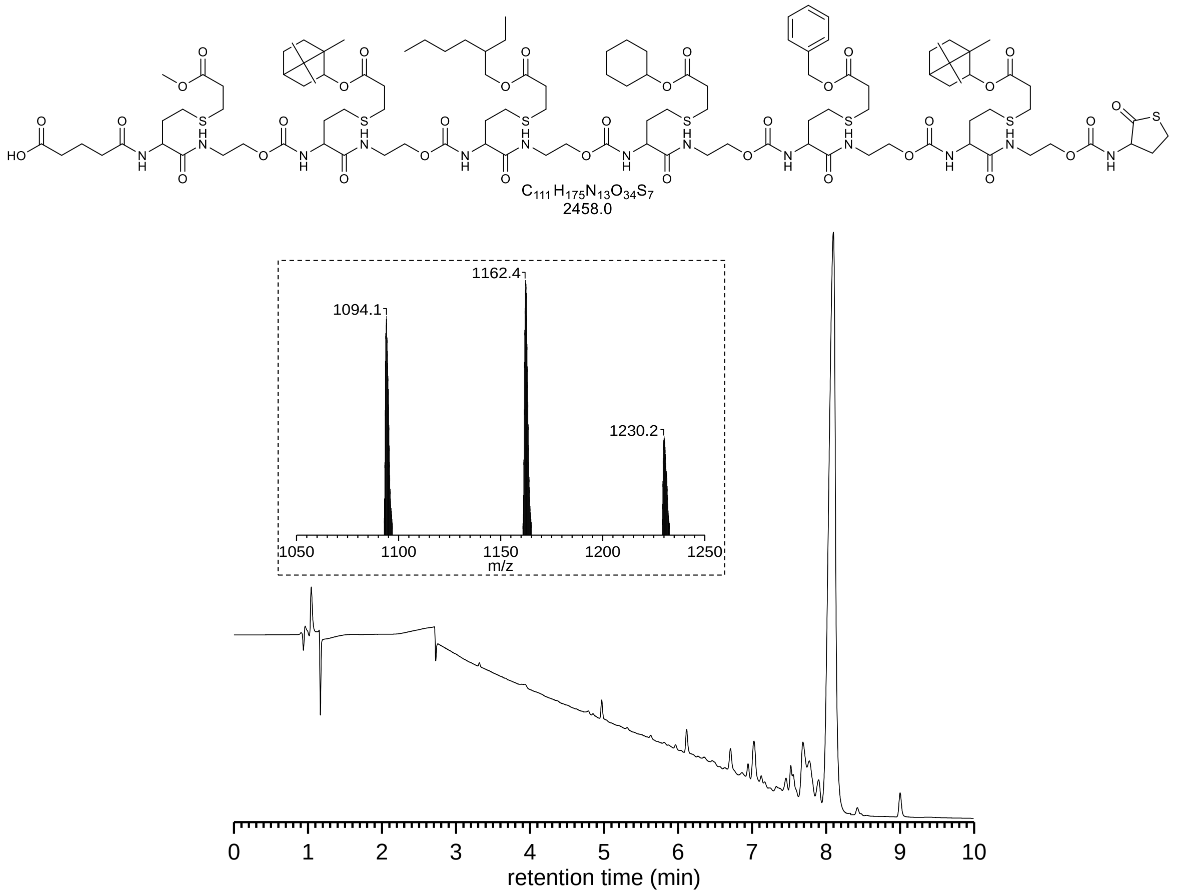

Supplementary Figure 190 | LC-ESI-MS analysis of **QR54**. Insert: ESI-MS-spectrum of dominant species (positive mode).

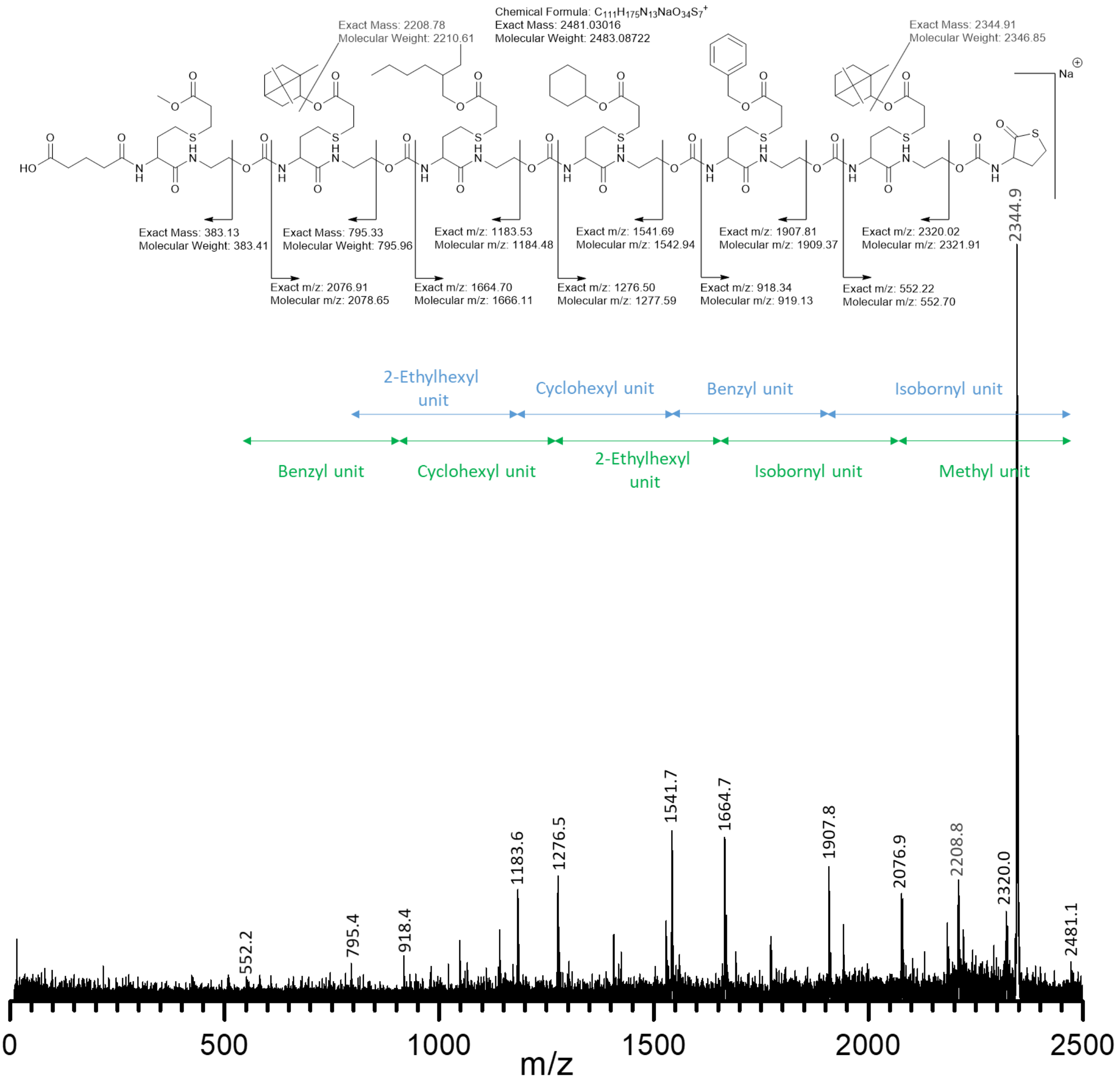

Supplementary Figure 191 | MALDI-MS/MS spectrum with peak assignment of **QR54**.

*Characterization of **QR55** using mass spectrometry (Supplementary Figure 192) and MALDI-MS/MS analysis (Supplementary Figure 193).*

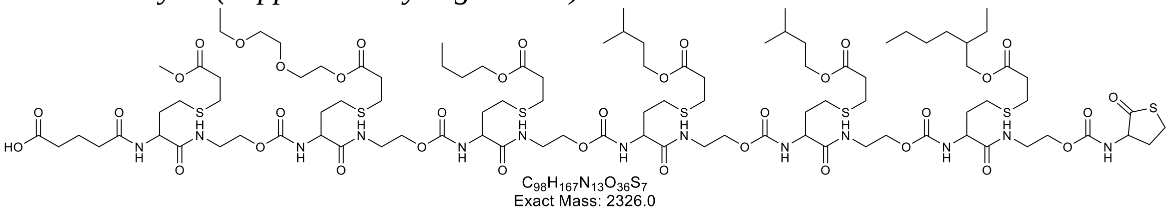

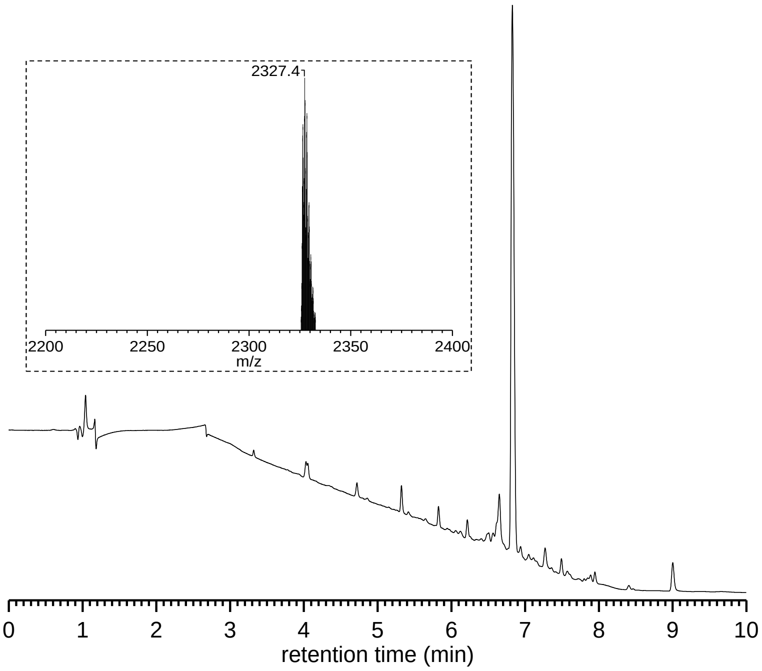

Supplementary Figure 192 | LC-ESI-MS analysis of **QR55**. Insert: ESI-MS-spectrum of dominant species (positive mode).

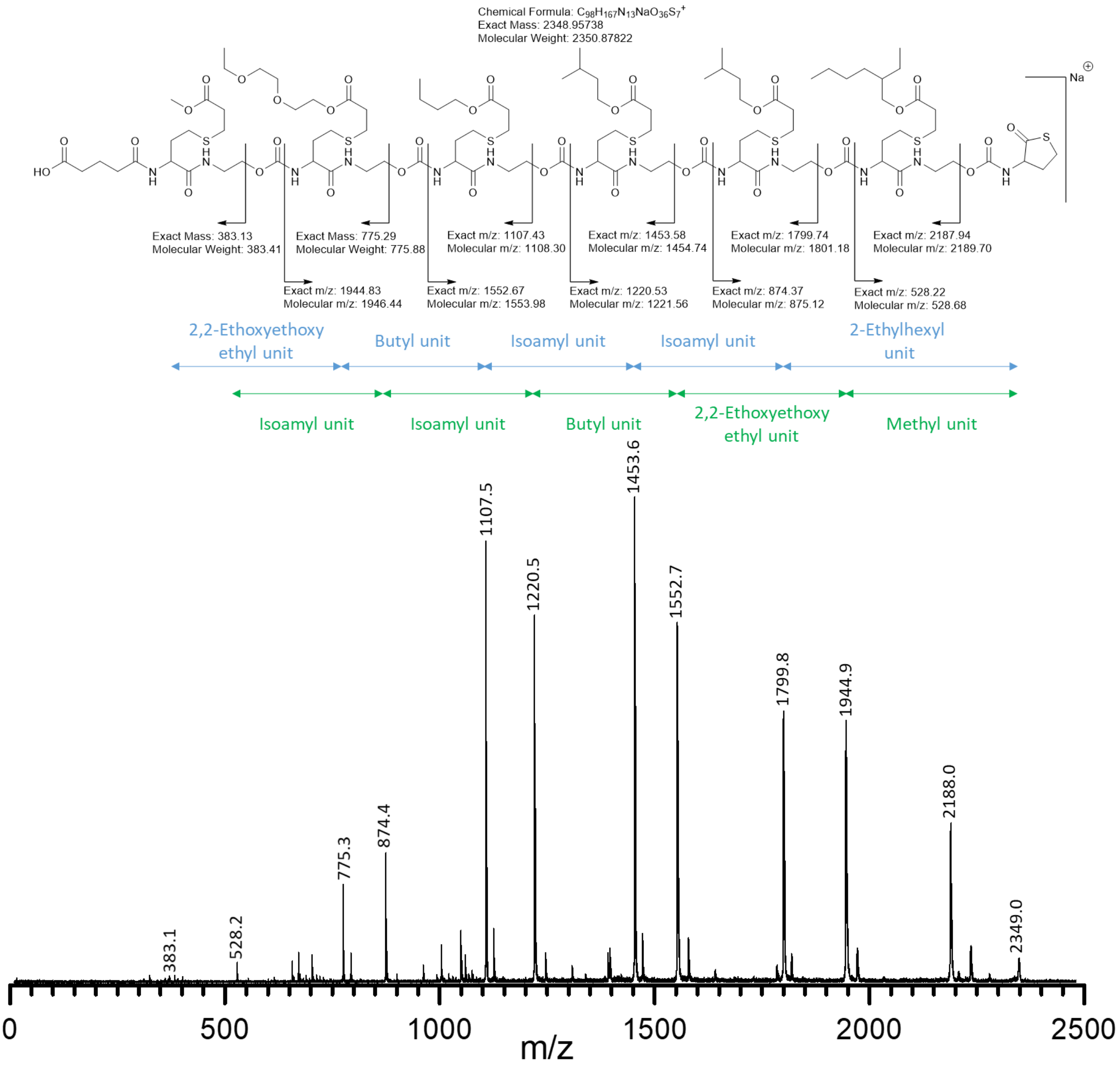

Supplementary Figure 193 | MALDI-MS/MS spectrum with peak assignment of **QR55**.

*Characterization of **QR56** using mass spectrometry (Supplementary Figure 194) and MALDI-MS/MS analysis (Supplementary Figure 195).*

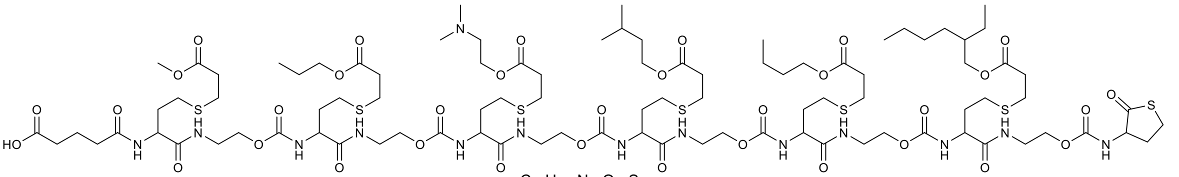

$C_{94}H_{160}N_{14}O_{34}S_7$
2252.9

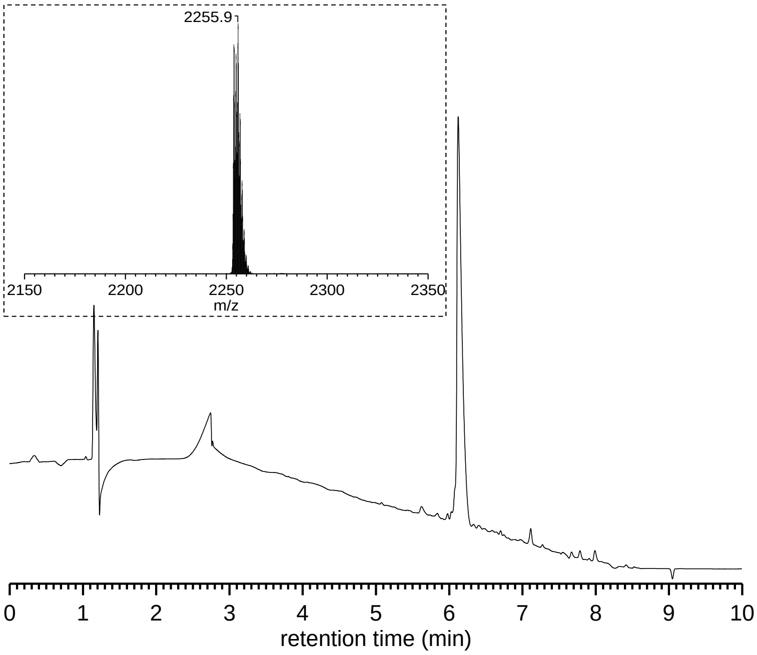

Supplementary Figure 194 | LC-ESI-MS analysis of **QR56**. Insert: ESI-MS-spectrum of dominant species (positive mode).

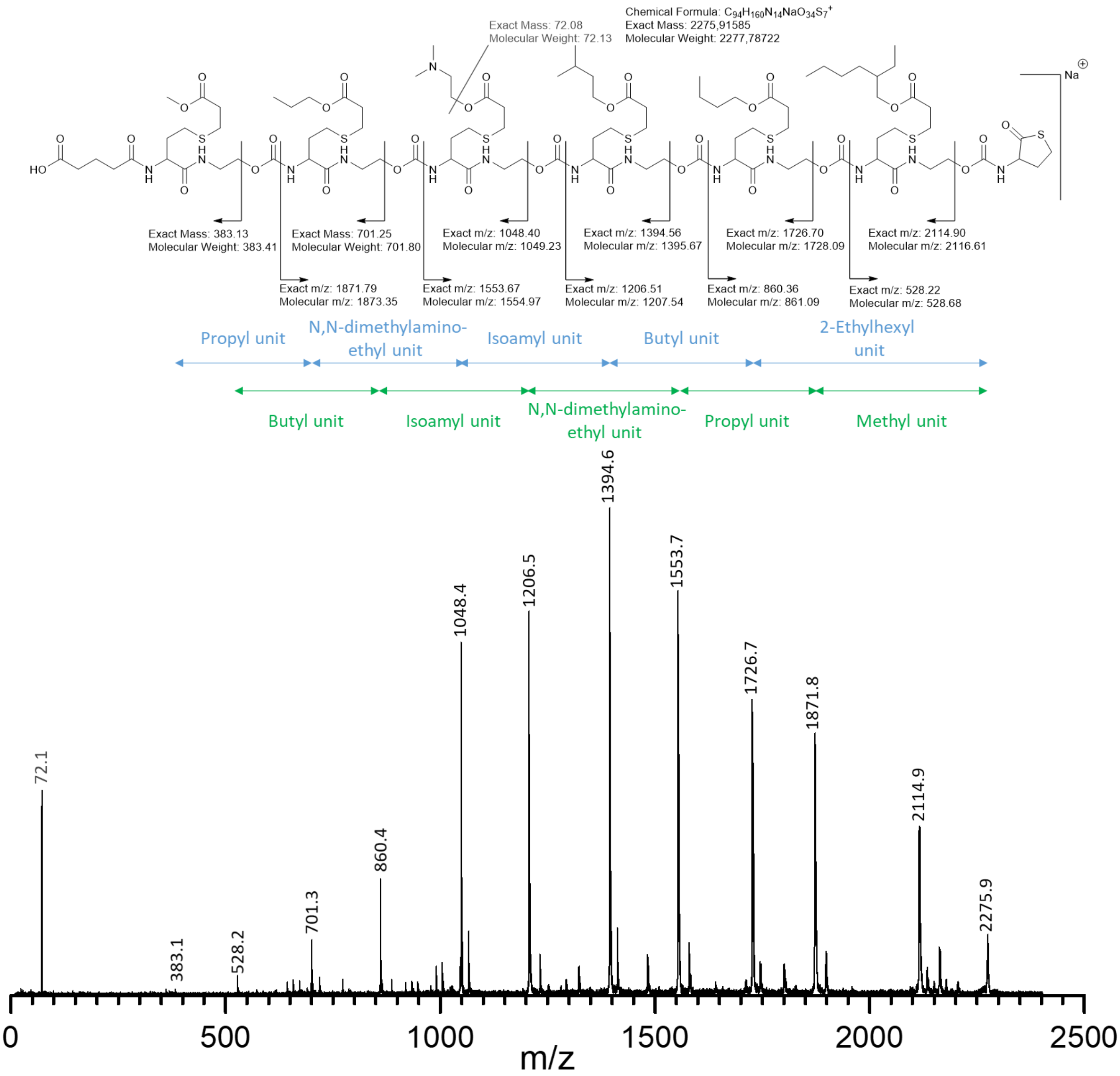

Supplementary Figure 195 | MALDI-MS/MS spectrum with peak assignment of **QR56**.

Characterization of ***QR57*** *using mass spectrometry (*Supplementary *Figure 196) and MALDI-MS/MS analysis (*Supplementary *Figure 197).*

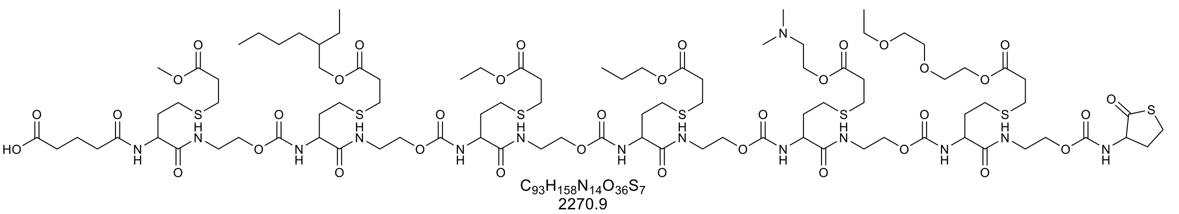

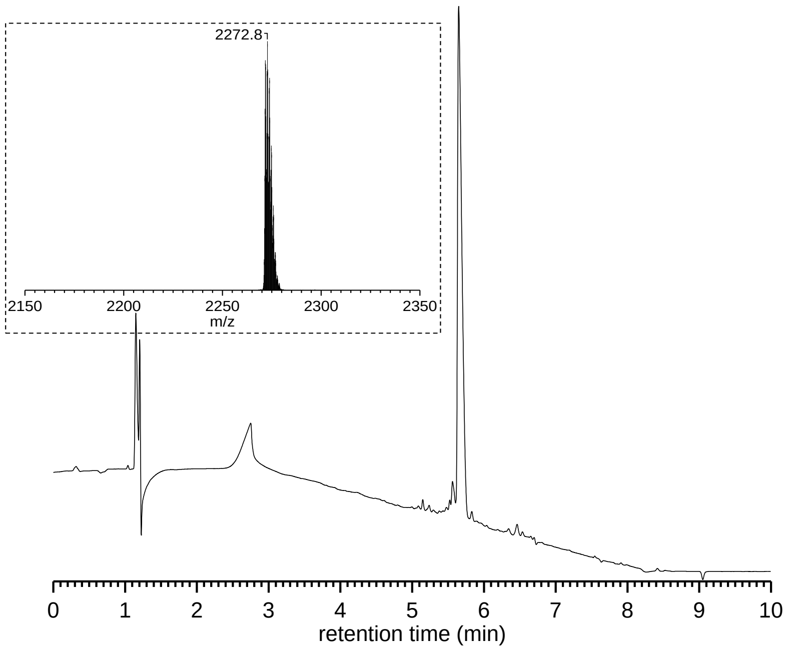

Supplementary Figure 196 | LC-ESI-MS analysis of **QR57**. Insert: ESI-MS-spectrum of dominant species (positive mode).

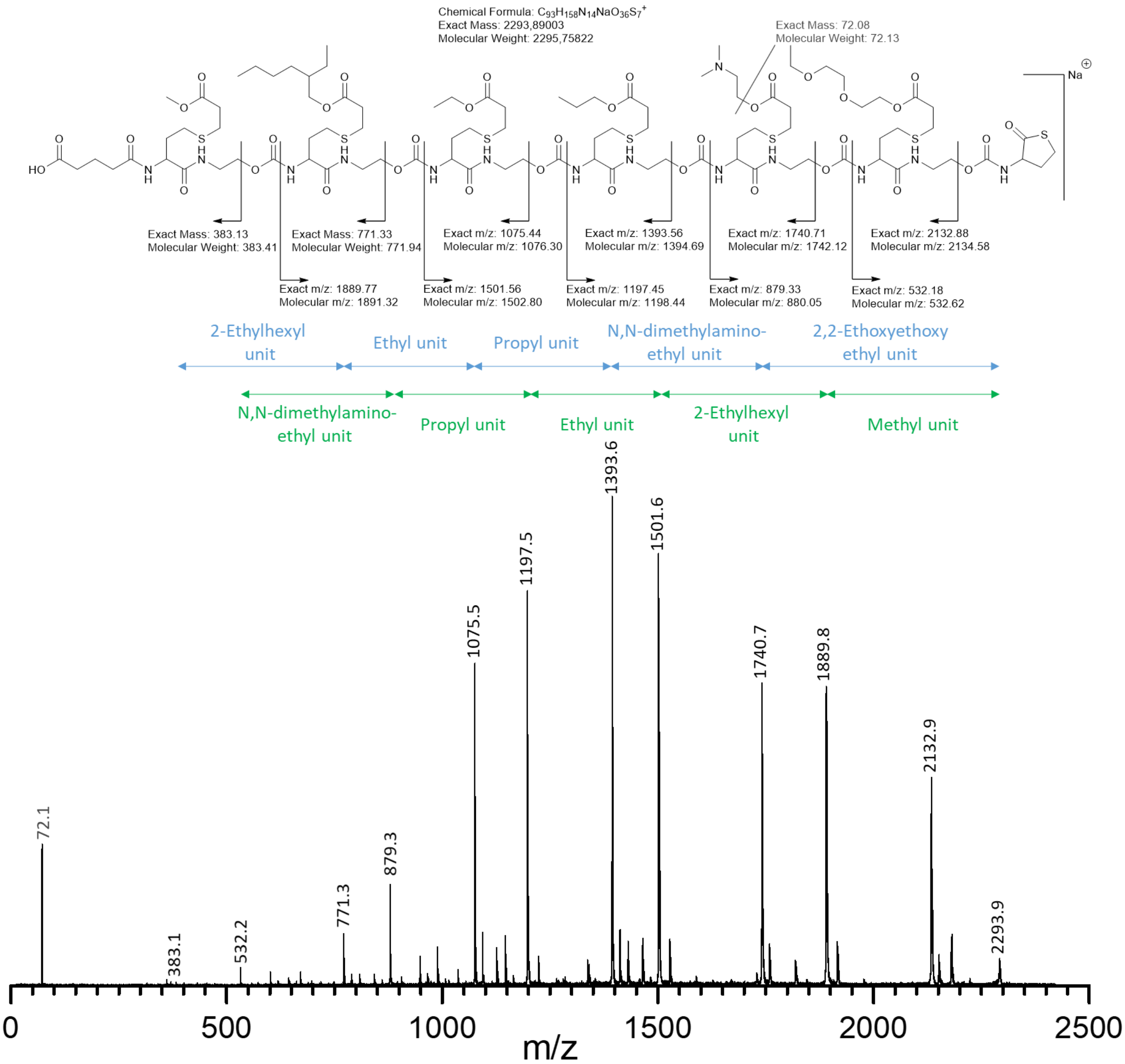

Supplementary Figure 197 | MALDI-MS/MS spectrum with peak assignment of **QR57**.

Characterization of ***QR58*** *using mass spectrometry (*Supplementary *Figure 198) and MALDI-MS/MS analysis (*Supplementary *Figure 199).*

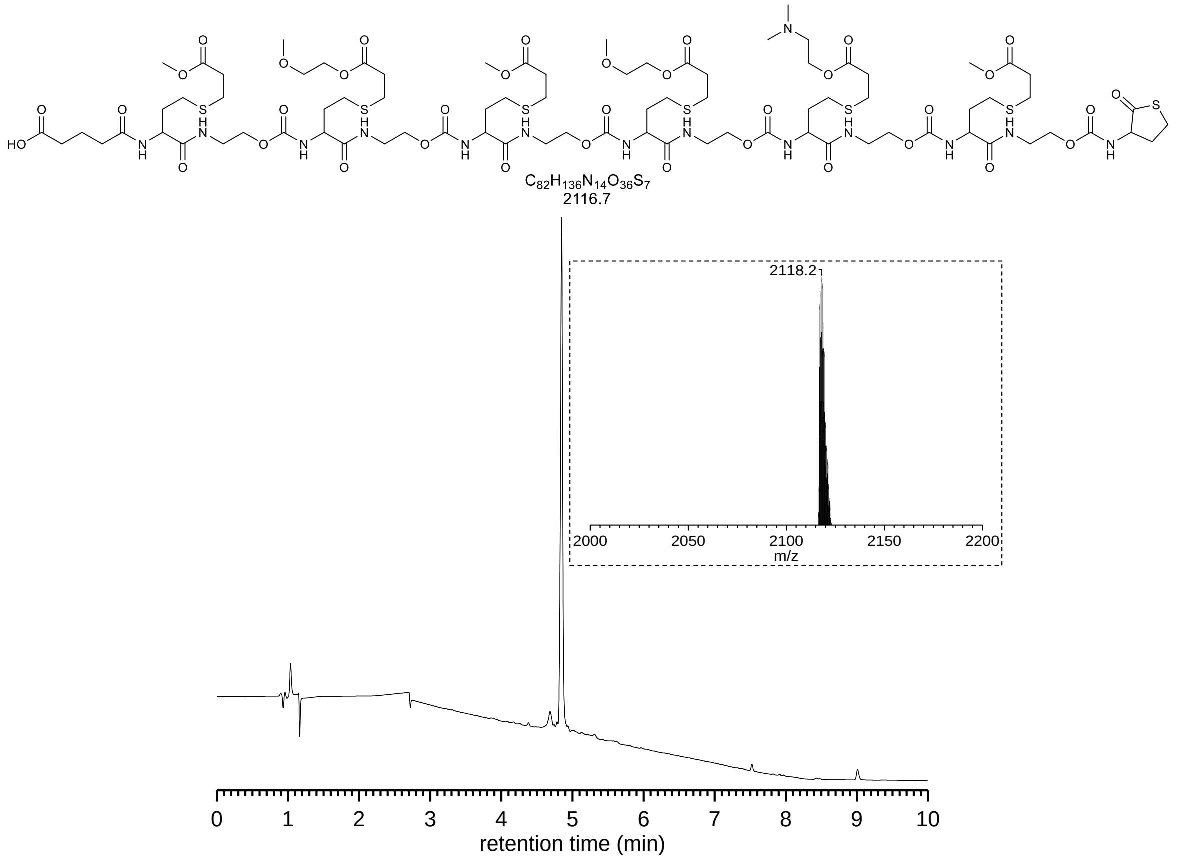

Supplementary Figure 198 | LC-ESI-MS analysis of **QR58**. Insert: ESI-MS-spectrum of dominant species (positive mode).

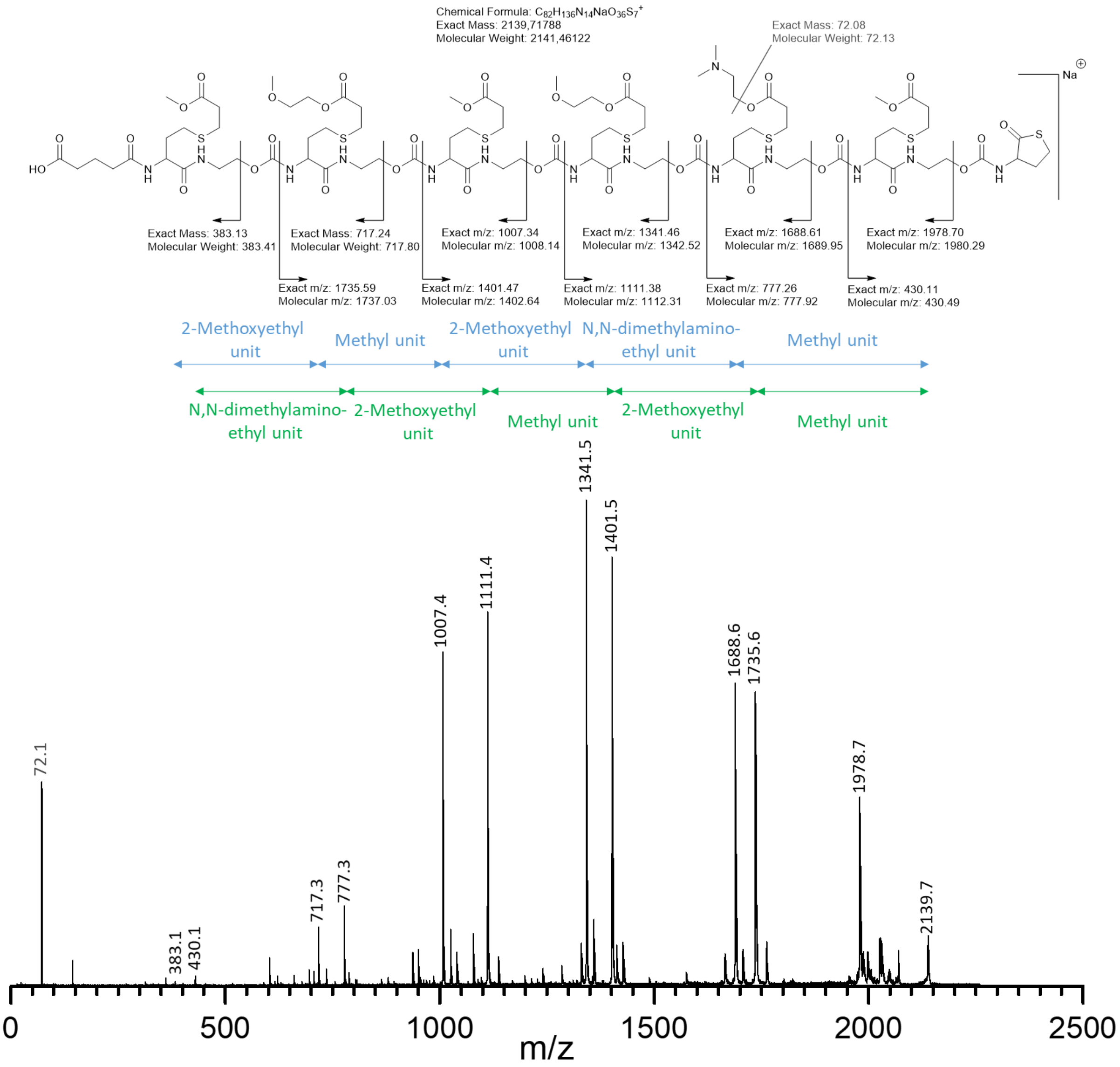

Supplementary Figure 199 | MALDI-MS/MS spectrum with peak assignment of **QR58**.

Characterization of ***QR59*** *using mass spectrometry (*Supplementary *Figure 200) and MALDI-MS/MS analysis (*Supplementary *Figure 201).*

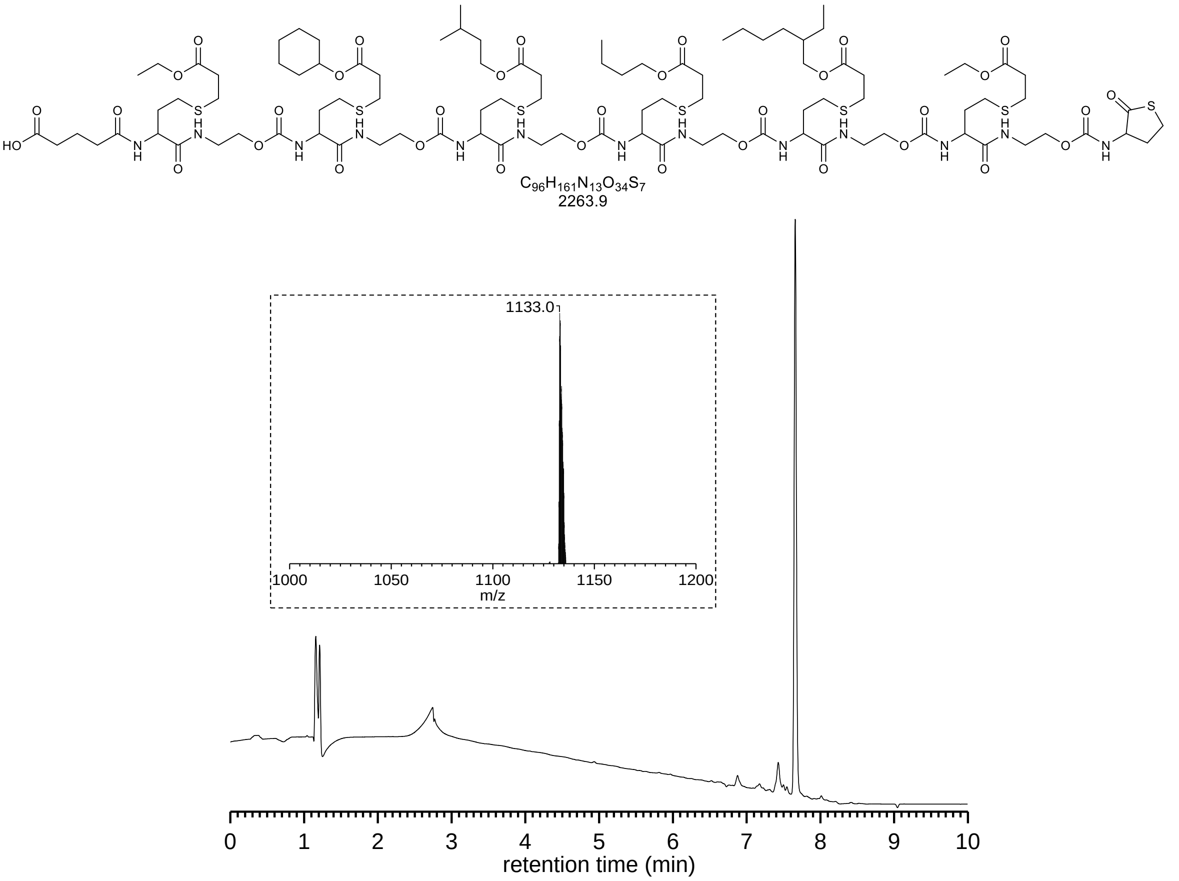

Supplementary Figure 200 | LC-ESI-MS analysis of **QR59**. Insert: ESI-MS-spectrum of dominant species (positive mode).

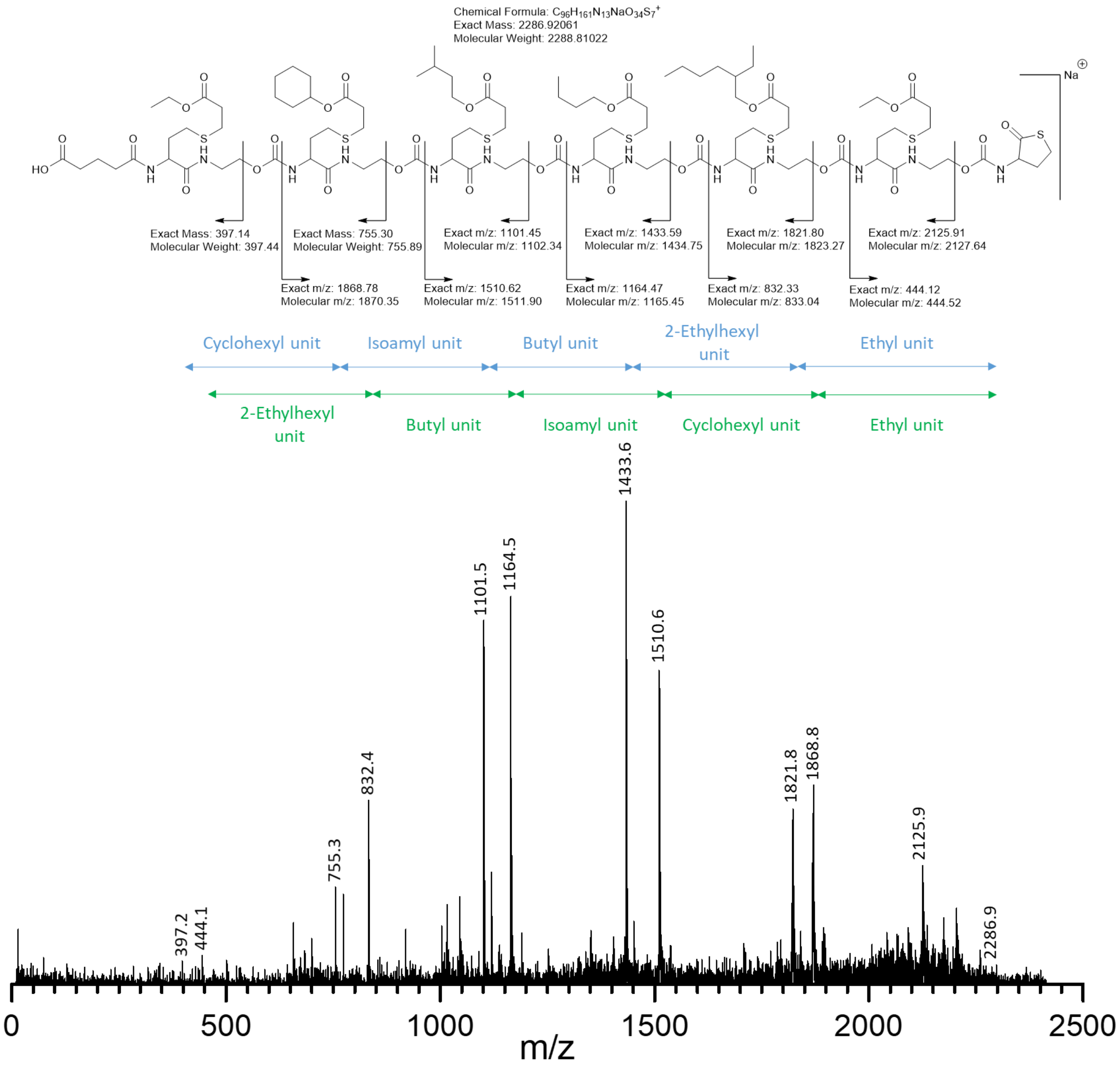

Supplementary Figure 201 | MALDI-MS/MS spectrum with peak assignment of **QR59**.

*Characterization of **QR60** using mass spectrometry (Supplementary Figure 202) and MALDI-MS/MS analysis (Supplementary Figure 203).*

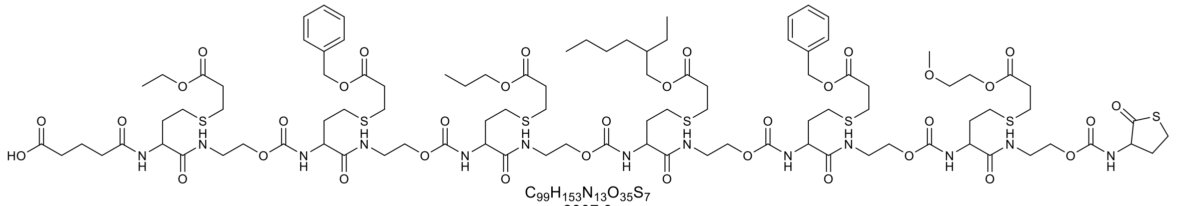

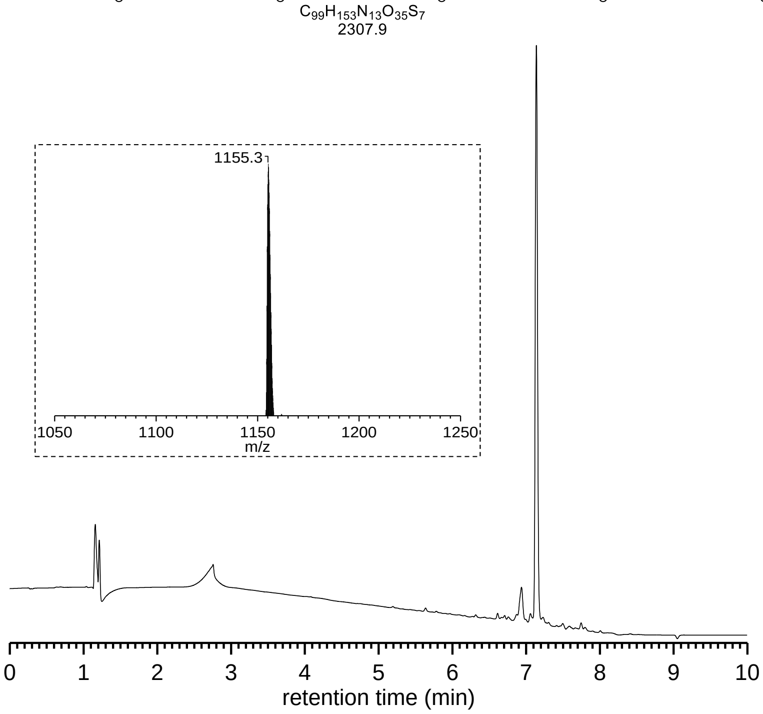

Supplementary Figure 202 | LC-ESI-MS analysis of **QR60**. Insert: ESI-MS-spectrum of dominant species (positive mode).

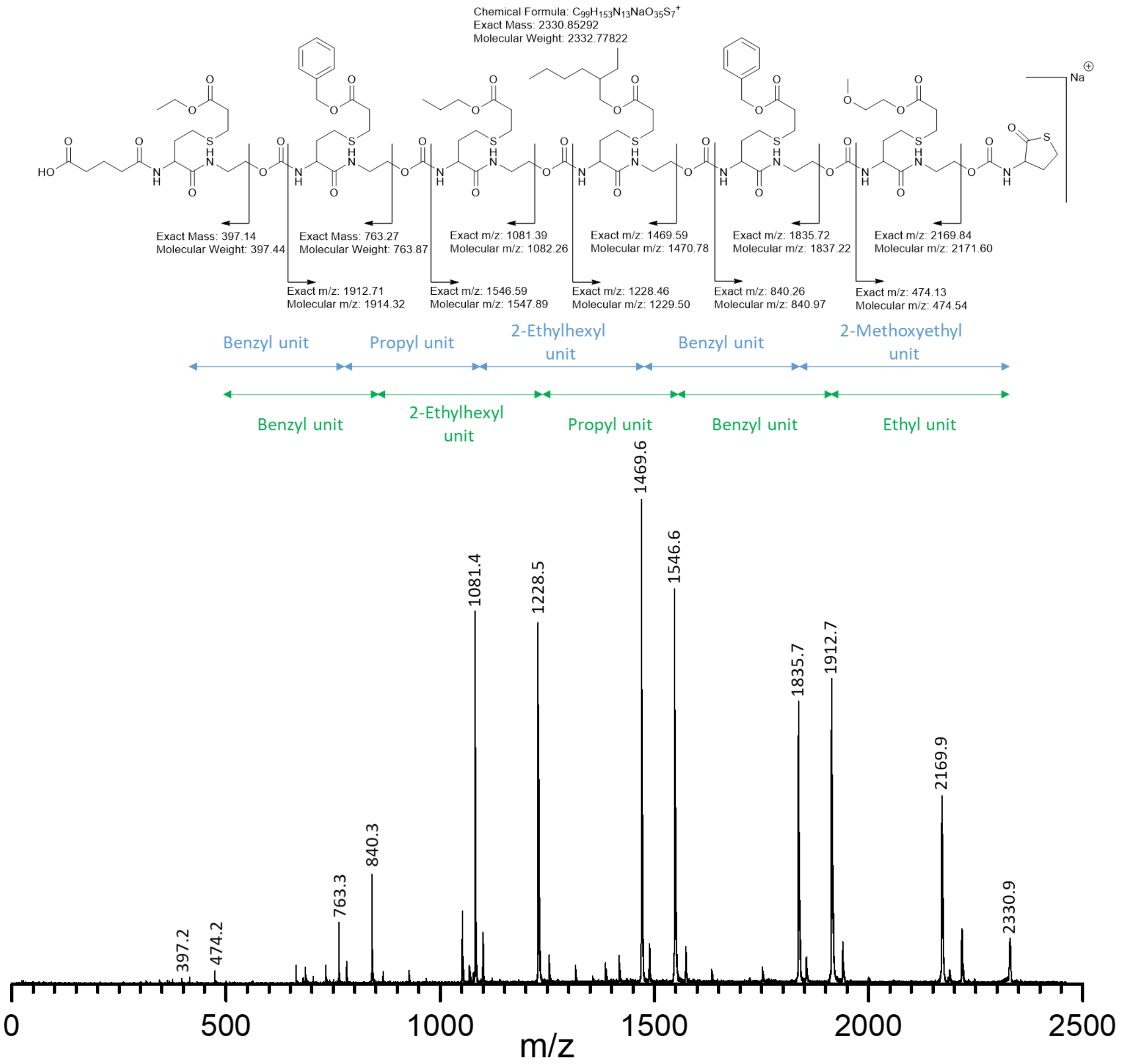

Supplementary Figure 203 | MALDI-MS/MS spectrum with peak assignment of **QR60**.

*Characterization of **QR61** using mass spectrometry (Supplementary Figure 204) and MALDI-MS/MS analysis (Supplementary Figure 205).*

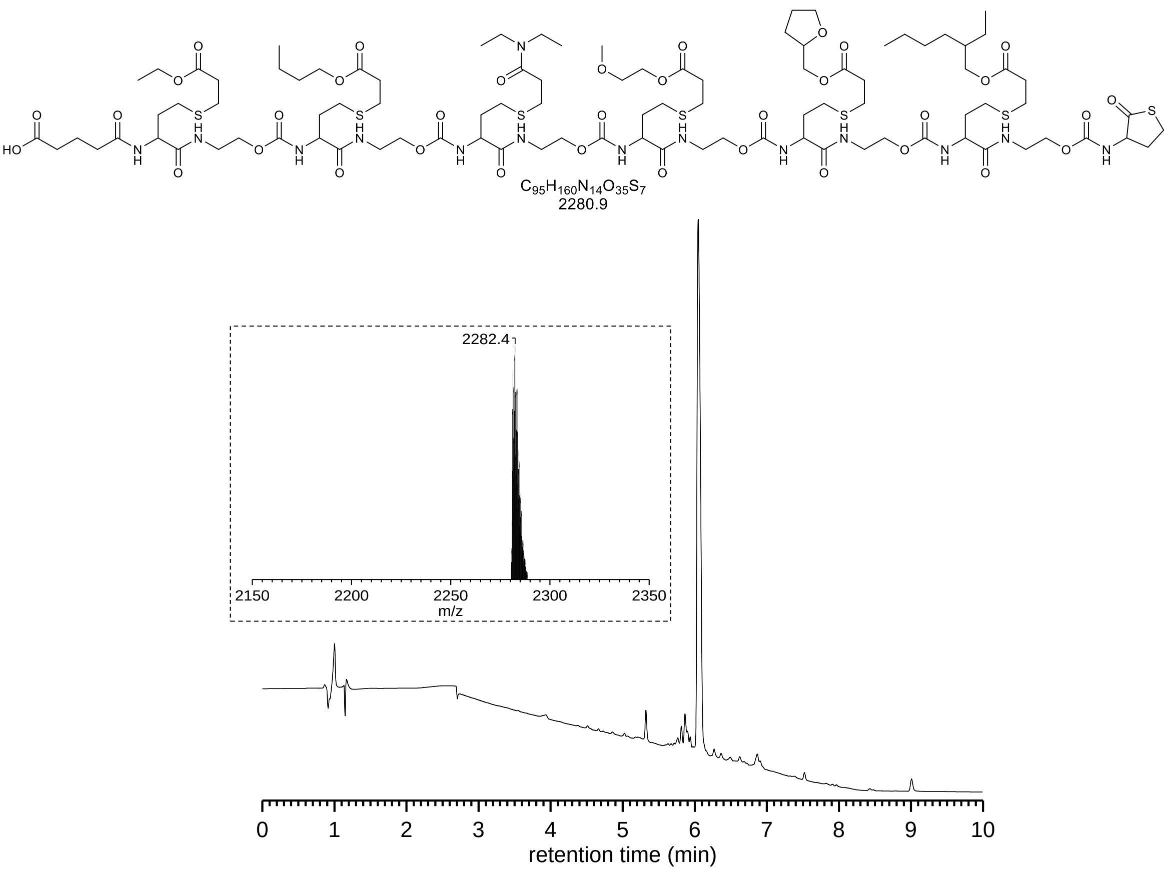

Supplementary Figure 204 | LC-ESI-MS analysis of **QR61**. Insert: ESI-MS-spectrum of dominant species (positive mode).

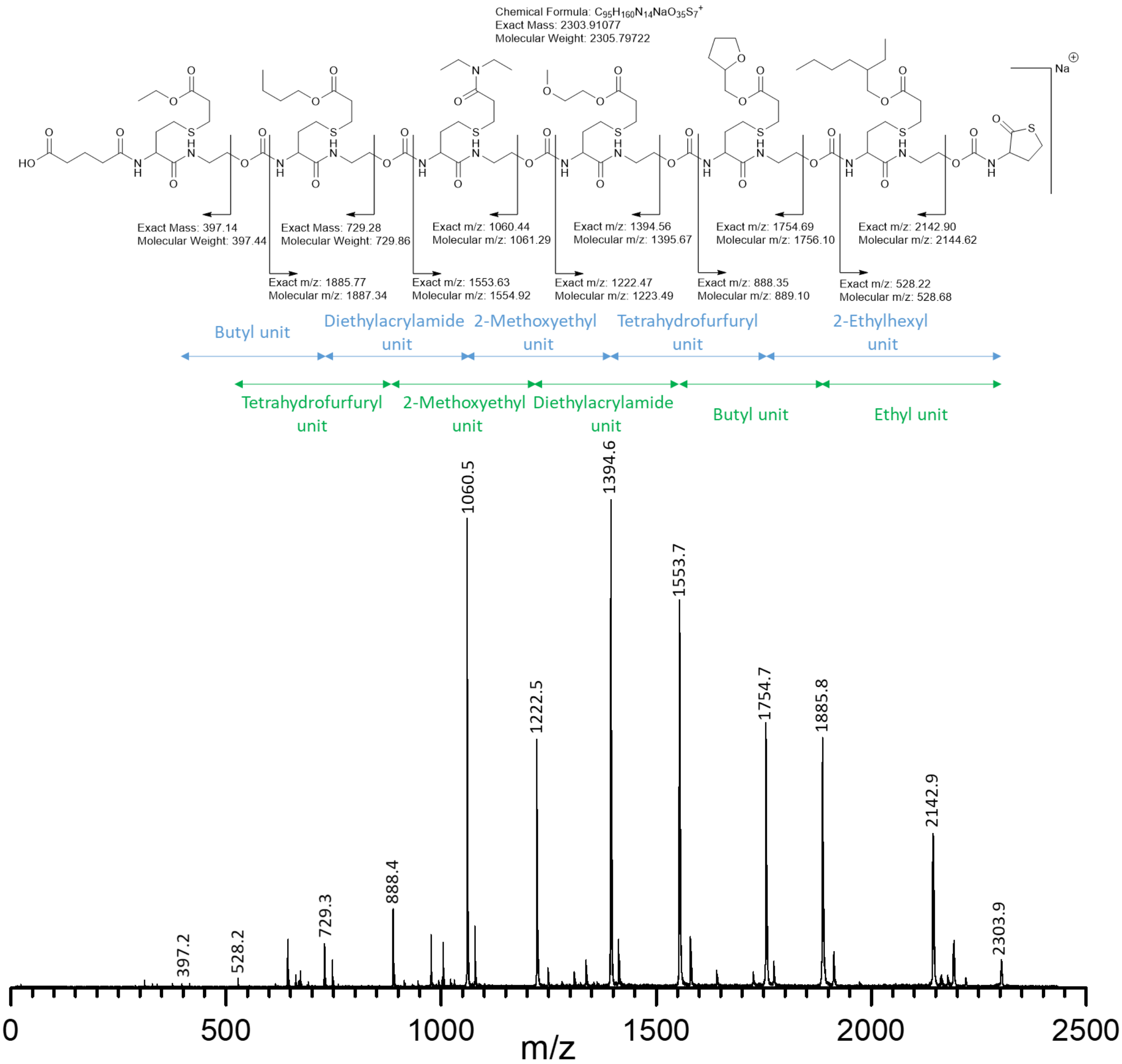

Supplementary Figure 205 | MALDI-MS/MS spectrum with peak assignment of **QR61**.

*Characterization of **QR62** using mass spectrometry (Supplementary Figure 206) and MALDI-MS/MS analysis (Supplementary Figure 207).*

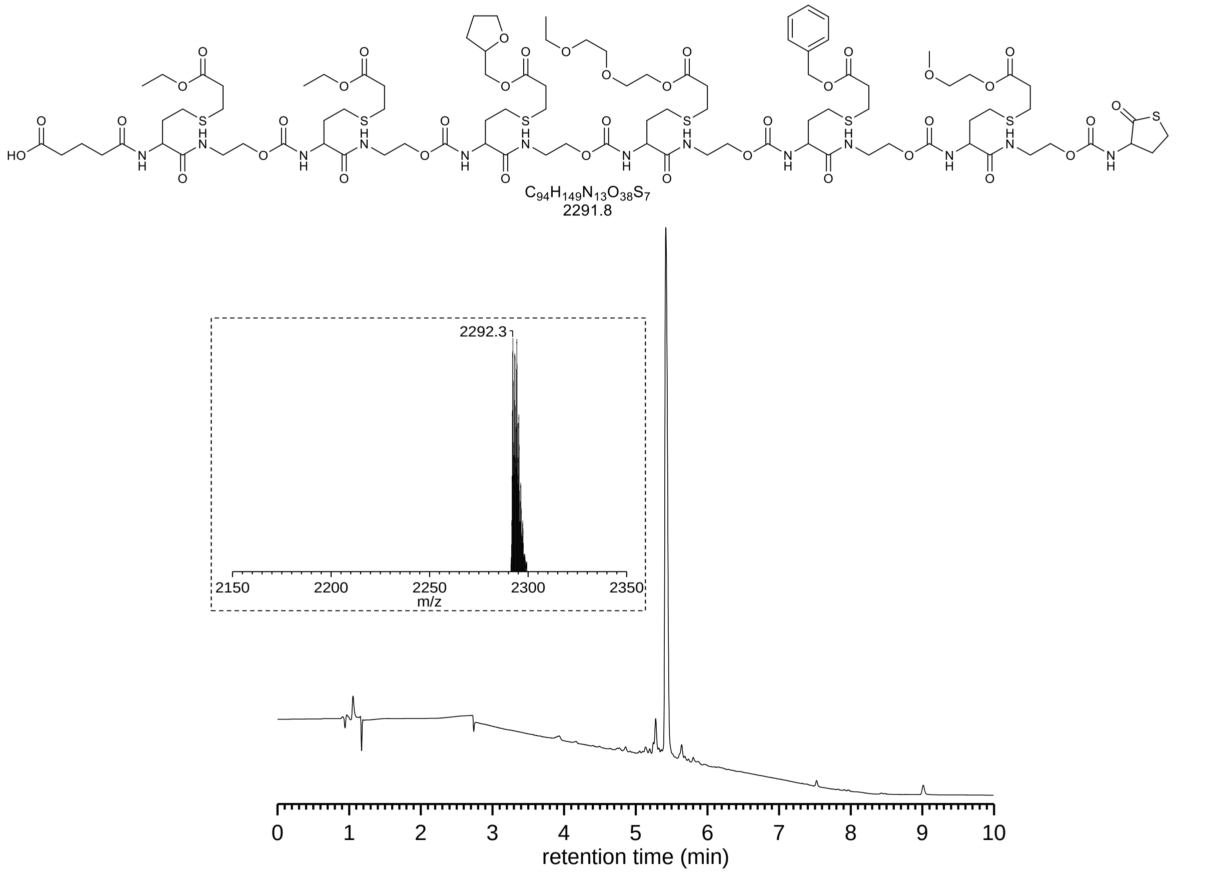

Supplementary Figure 206 | LC-ESI-MS analysis of **QR62**. Insert: ESI-MS-spectrum of dominant species (positive mode).

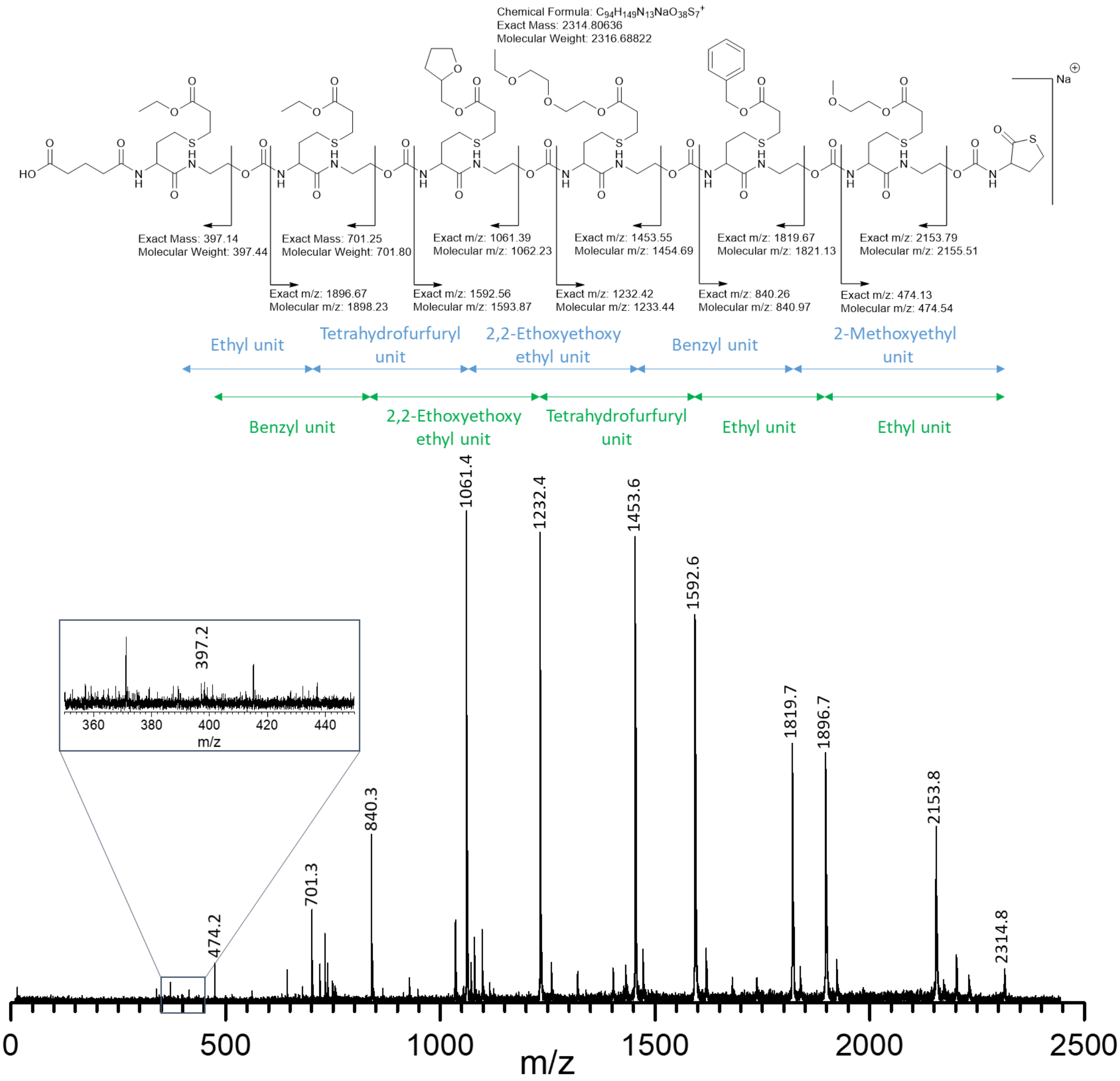

Supplementary Figure 207 | MALDI-MS/MS spectrum with peak assignment of **QR62**.

*Characterization of **QR63** using mass spectrometry (Supplementary Figure 208) and MALDI-MS/MS analysis (Supplementary Figure 209).*

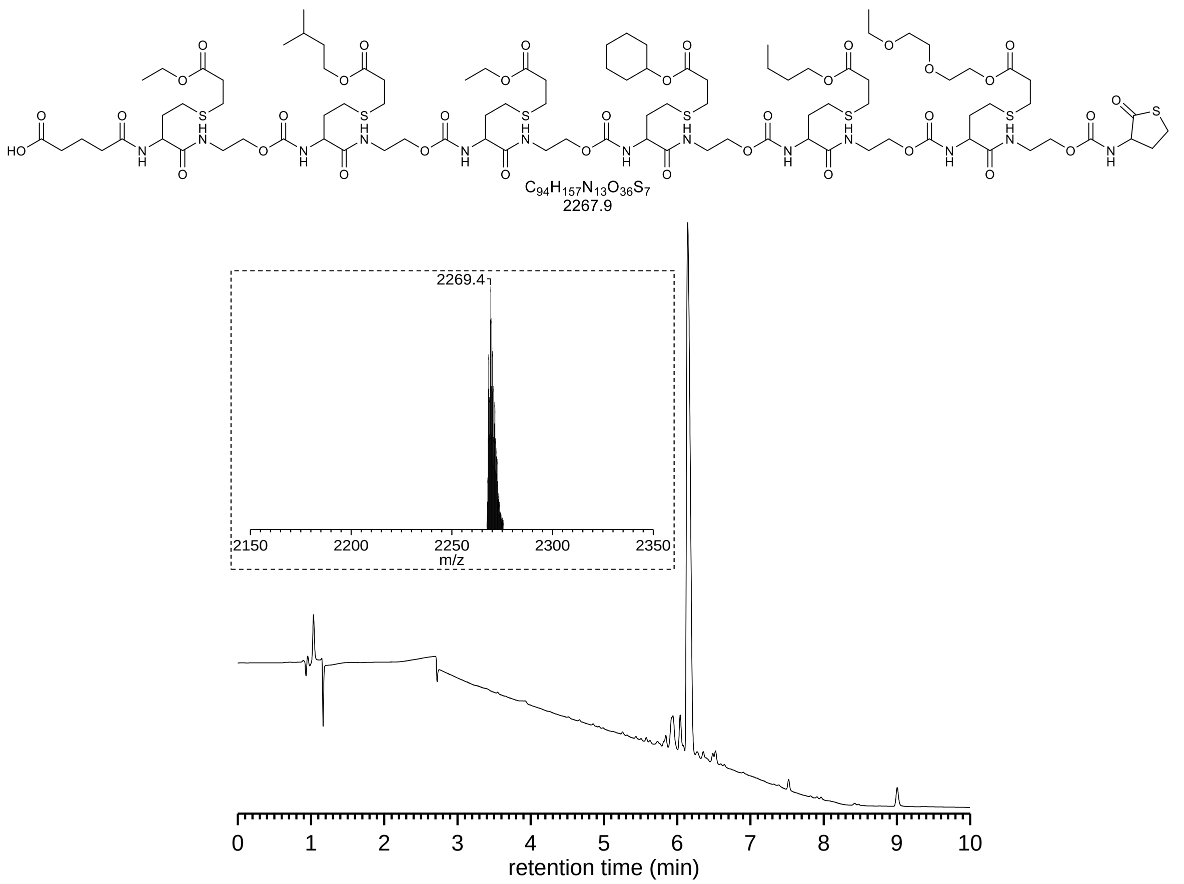

Supplementary Figure 208 | LC-ESI-MS analysis of **QR63**. Insert: ESI-MS-spectrum of dominant species (positive mode).

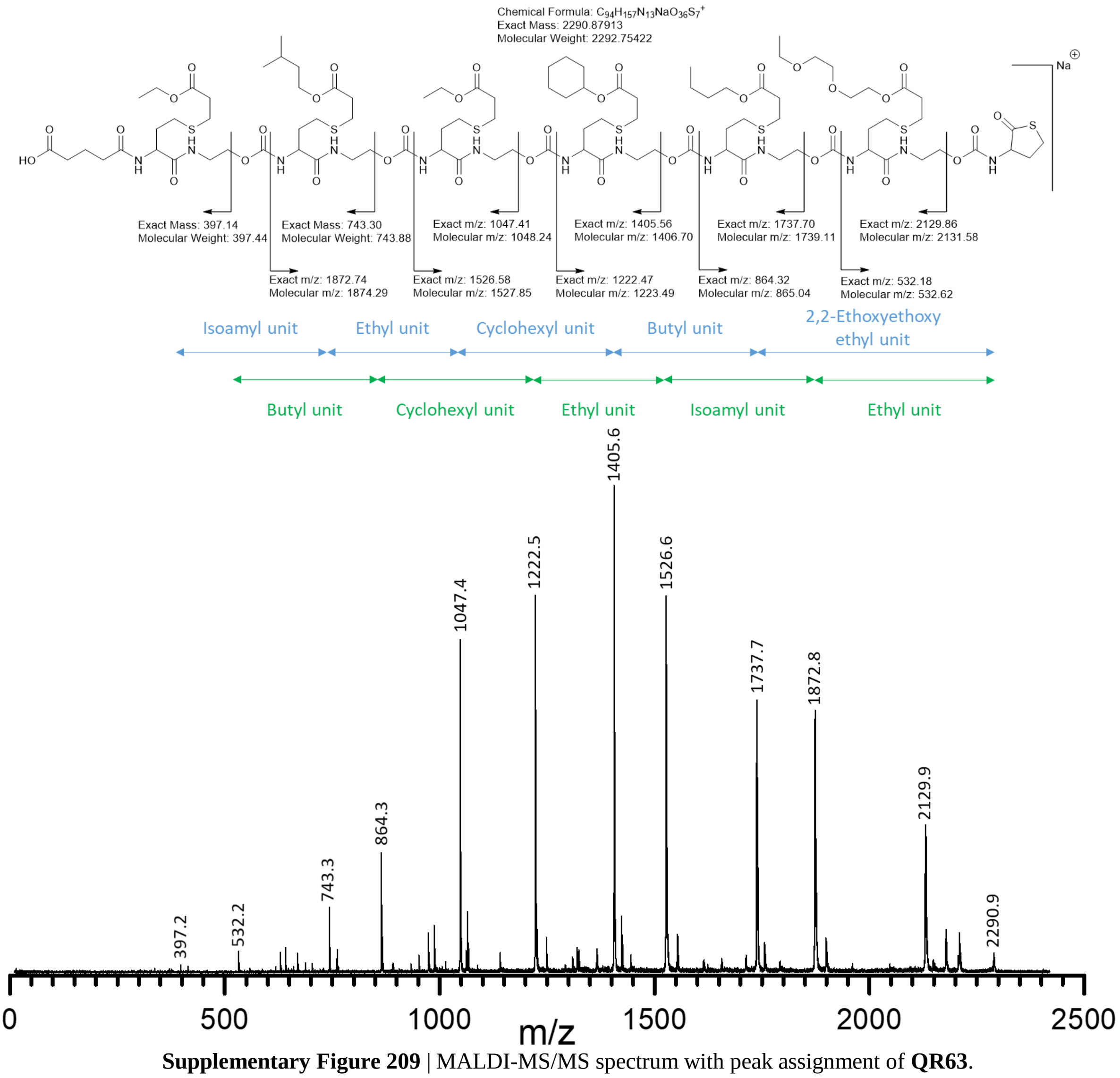

Supplementary Figure 209 | MALDI-MS/MS spectrum with peak assignment of **QR63**.

*Characterization of **QR64** using mass spectrometry (Supplementary Figure 210) and MALDI-MS/MS analysis (Supplementary Figure 211).*

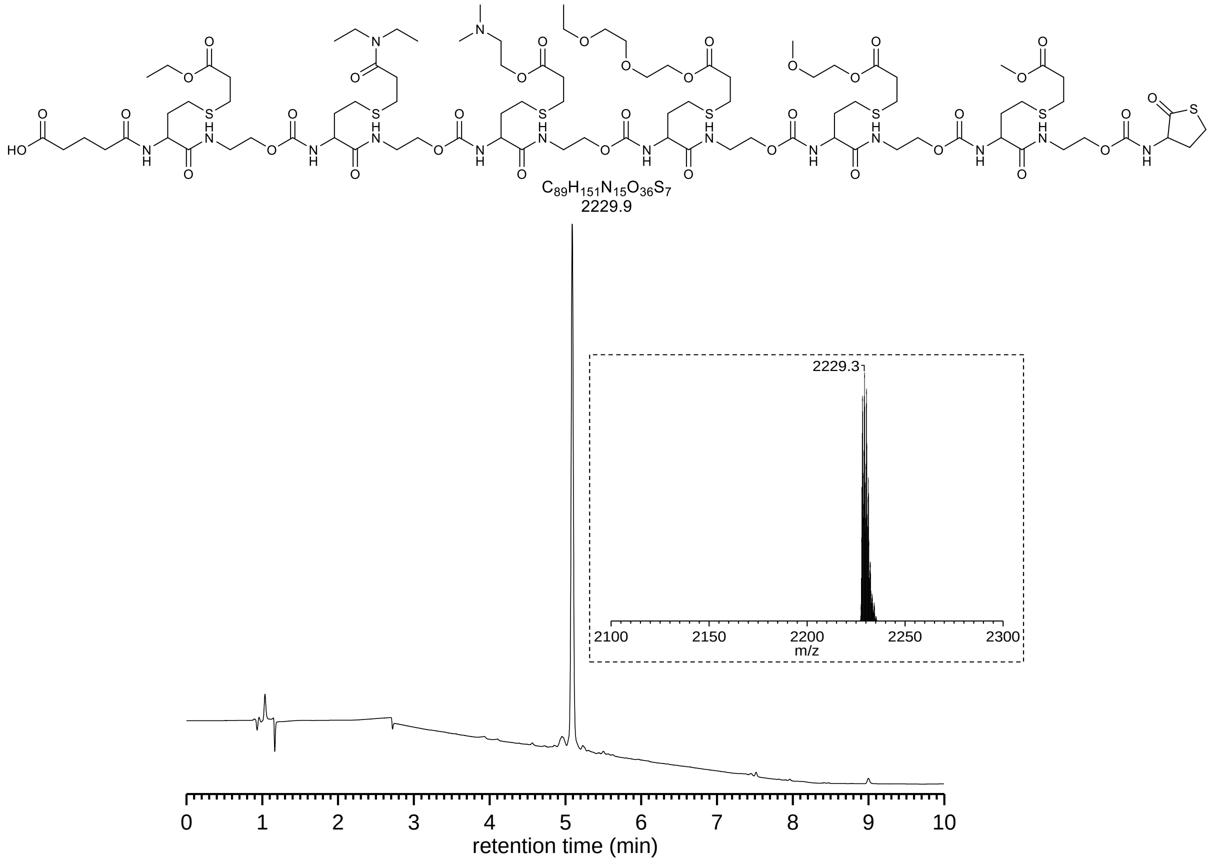

Supplementary Figure 210 | LC-ESI-MS analysis of **QR64**. Insert: ESI-MS-spectrum of dominant species (negative mode).

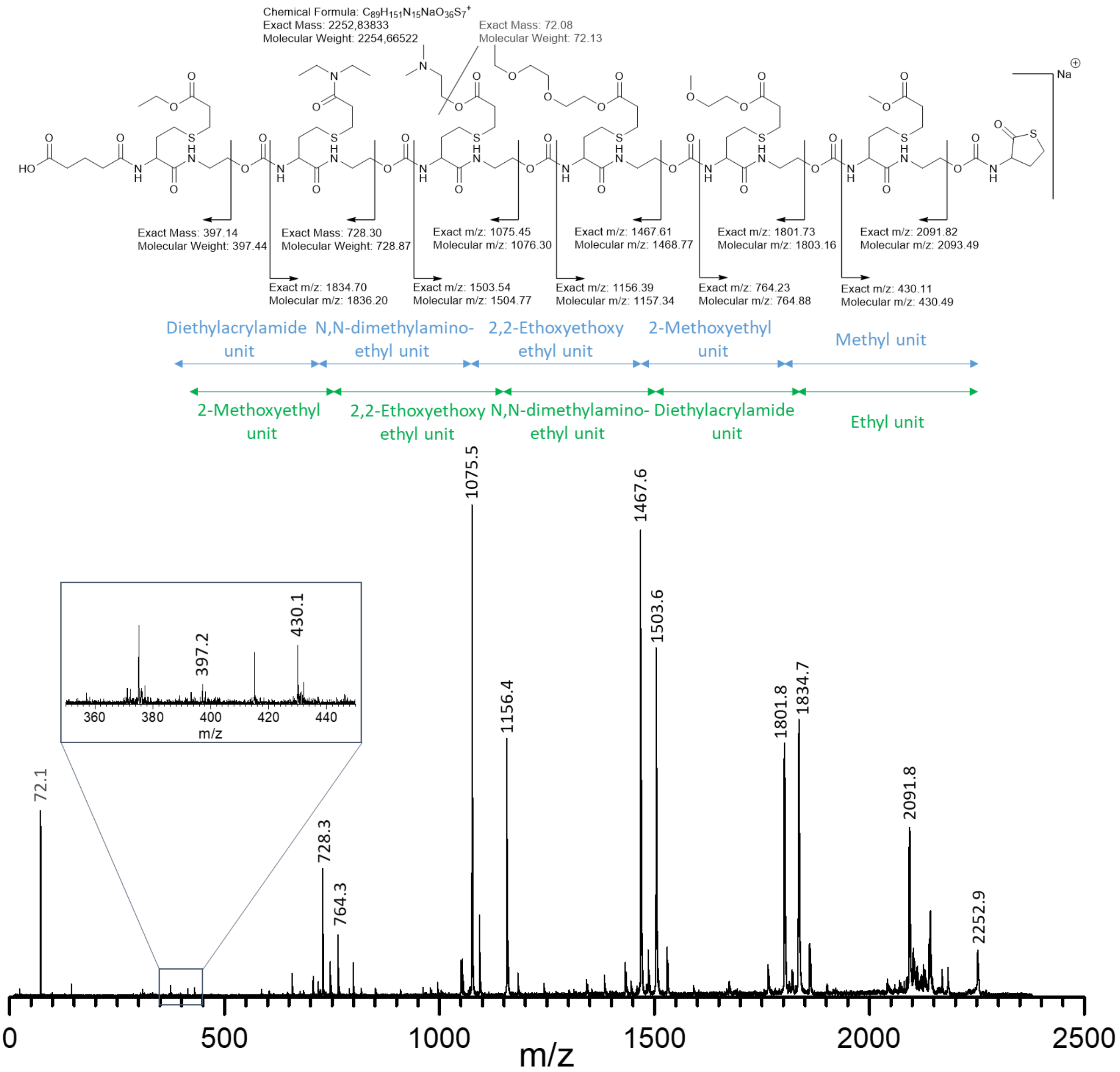

Supplementary Figure 211 | MALDI-MS/MS spectrum with peak assignment of **QR64**.

*Characterization of **QR65** using mass spectrometry (Supplementary Figure 212) and MALDI-MS/MS analysis (Supplementary Figure 213).*

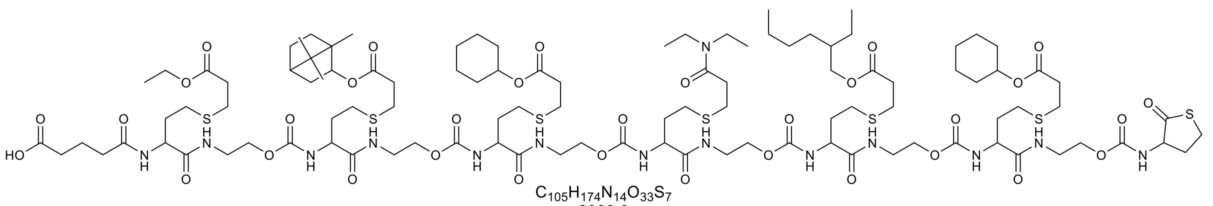

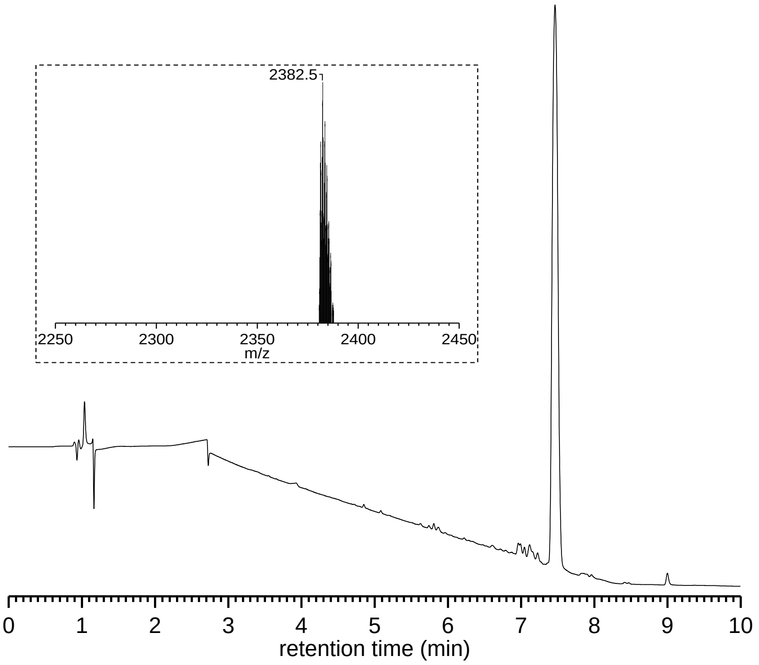

Supplementary Figure 212 | LC-ESI-MS analysis of **QR65**. Insert: ESI-MS-spectrum of dominant species (negative mode).

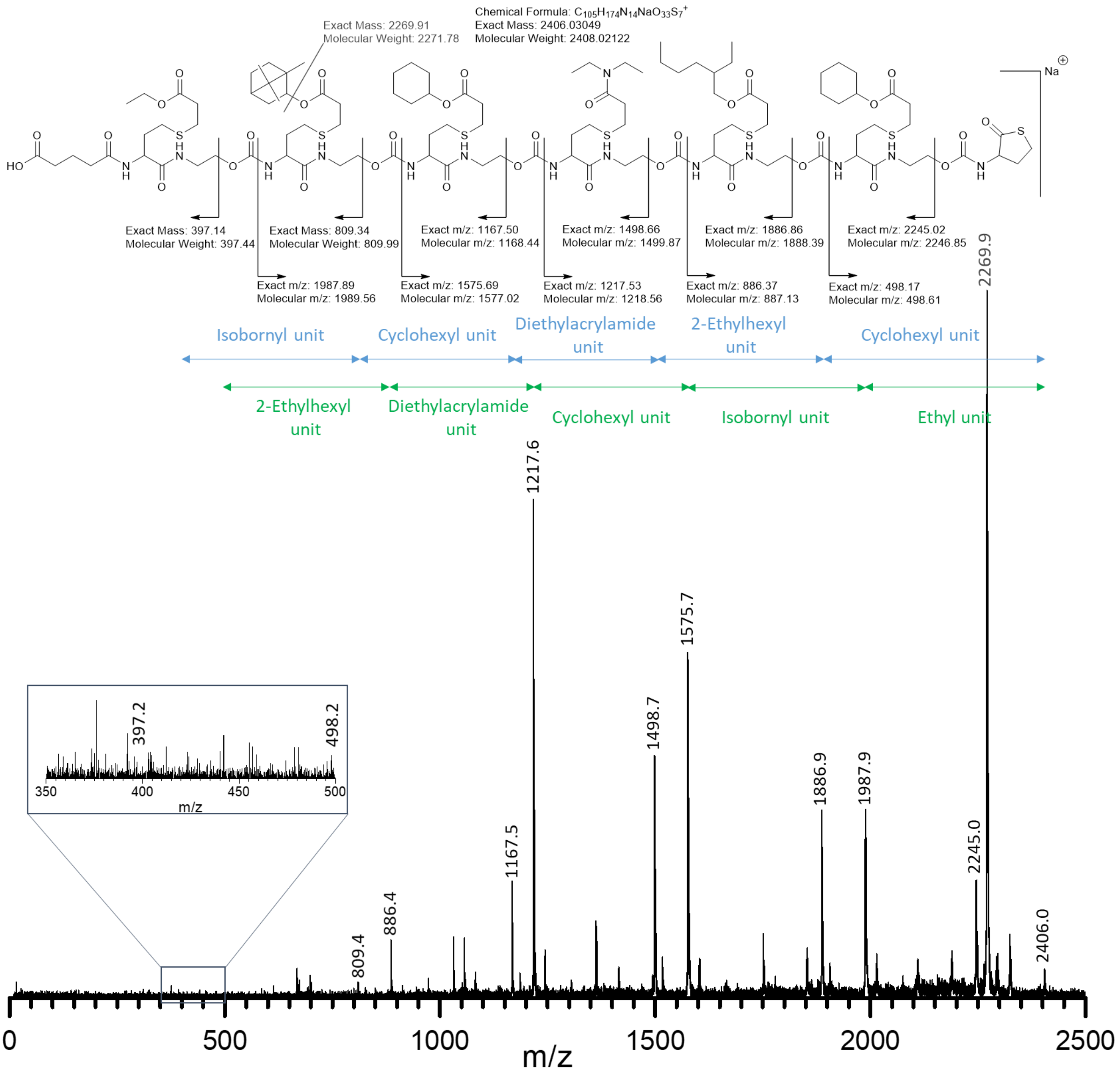

Supplementary Figure 213 | MALDI-MS/MS spectrum with peak assignment of **QR65**.

Characterization of ***QR66*** *using mass spectrometry (Supplementary Figure 214) and MALDI-MS/MS analysis (Supplementary Figure 215).*

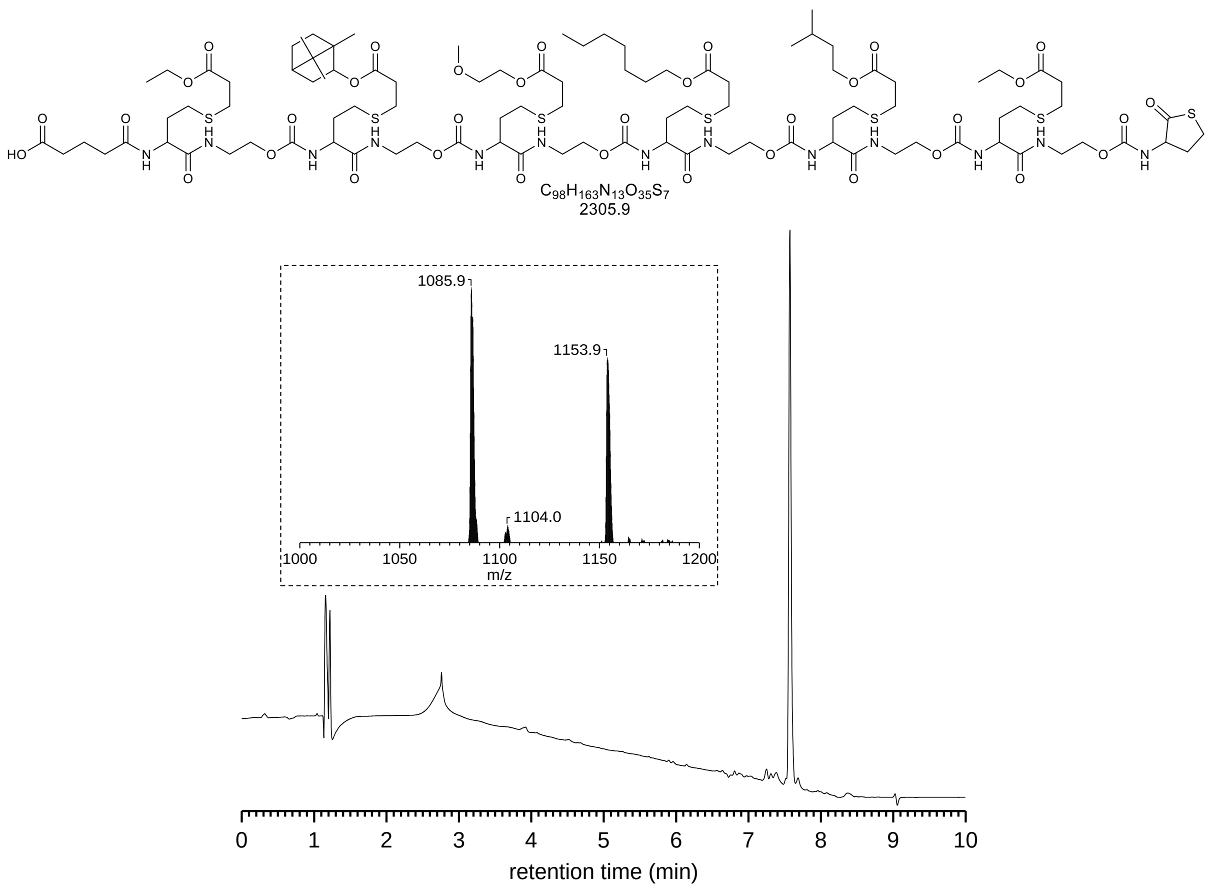

Supplementary Figure 214 | LC-ESI-MS analysis of **QR66**. Insert: ESI-MS-spectrum of dominant species (positive mode).

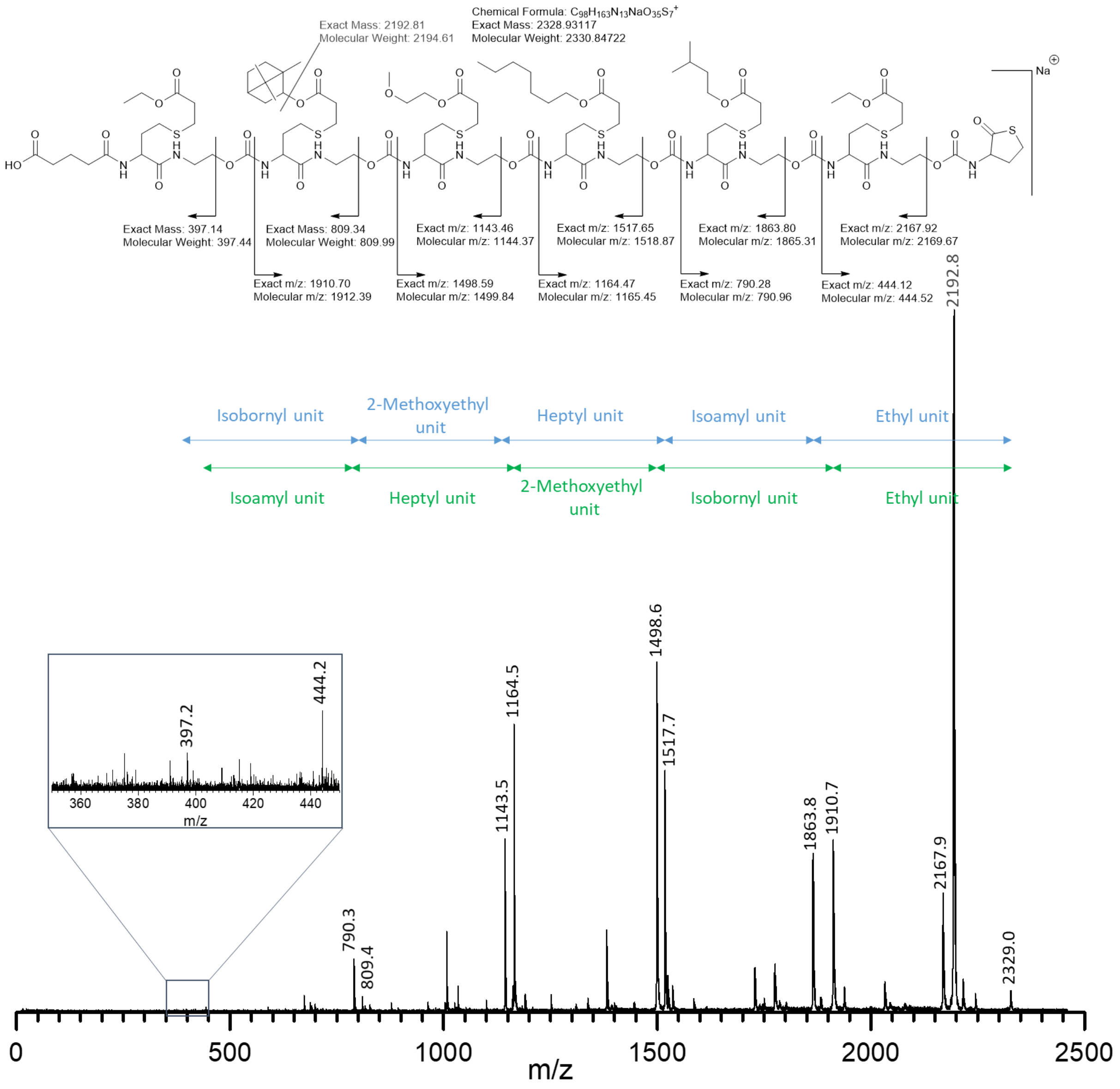

Supplementary Figure 215 | MALDI-MS/MS spectrum with peak assignment of **QR66**.

*Characterization of **QR67** using mass spectrometry (Supplementary Figure 216) and MALDI-MS/MS analysis (Supplementary Figure 217).*

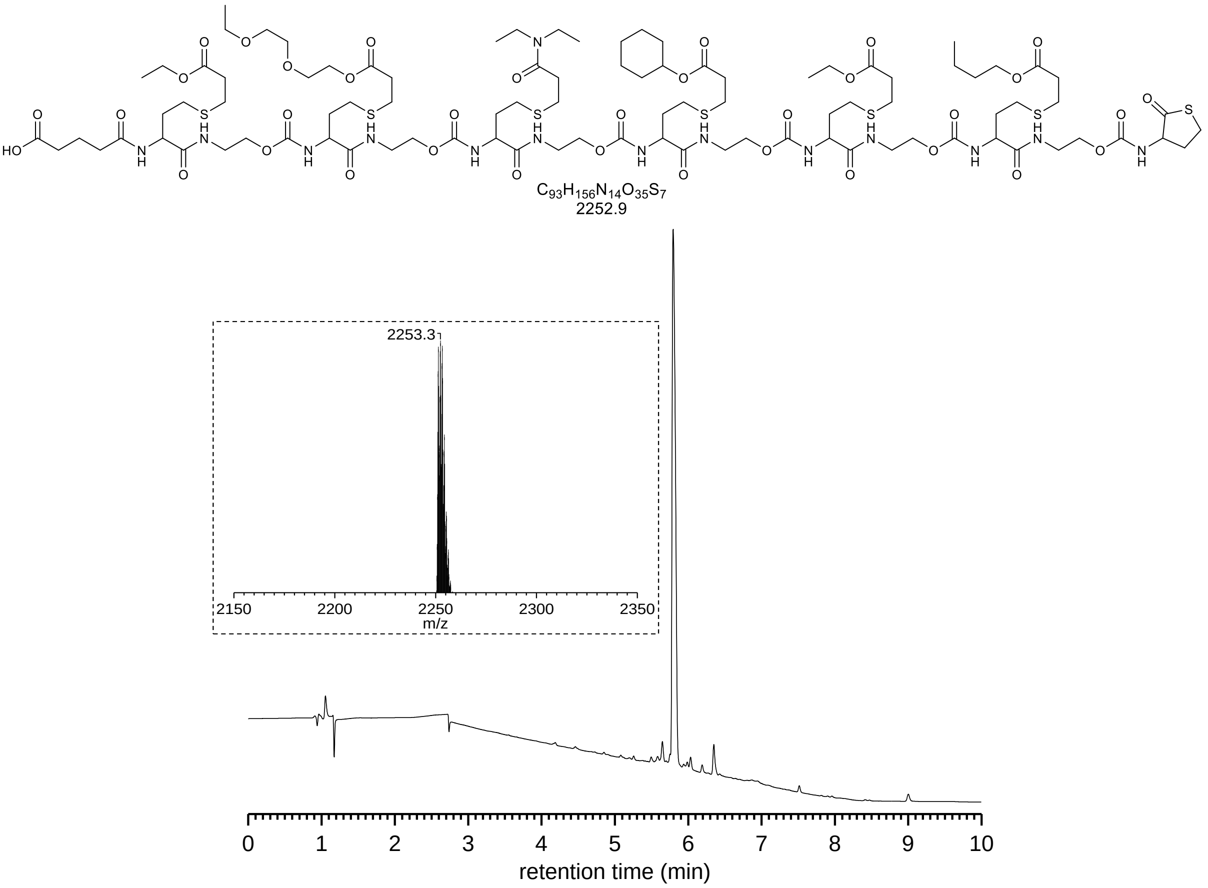

Supplementary Figure 216 | LC-ESI-MS analysis of **QR67**. Insert: ESI-MS-spectrum of dominant species (positive mode).

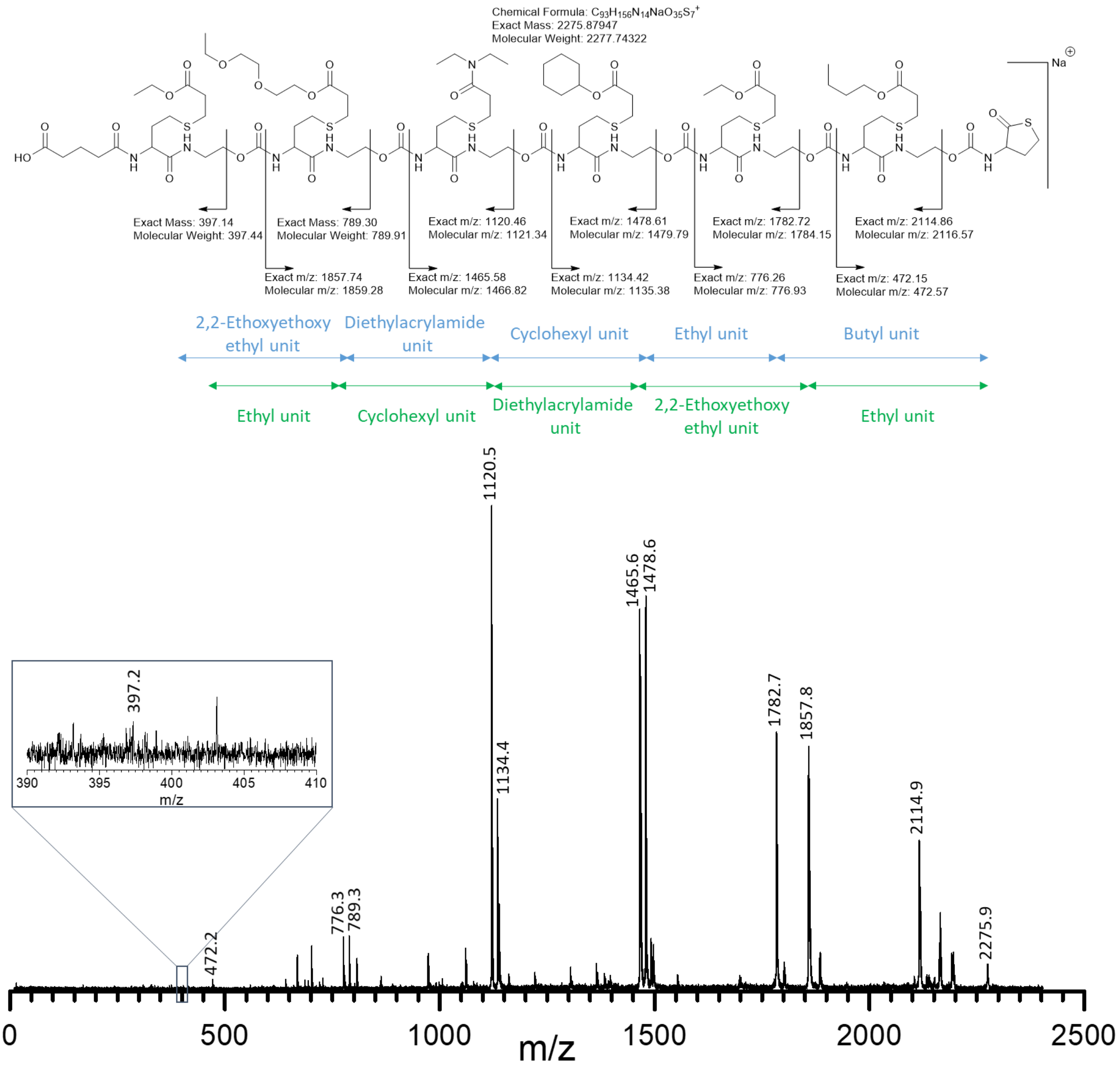

Supplementary Figure 217 | MALDI-MS/MS spectrum with peak assignment of **QR67**.

*Characterization of **QR68** using mass spectrometry (Supplementary Figure 218) and MALDI-MS/MS analysis (Supplementary Figure 219).*

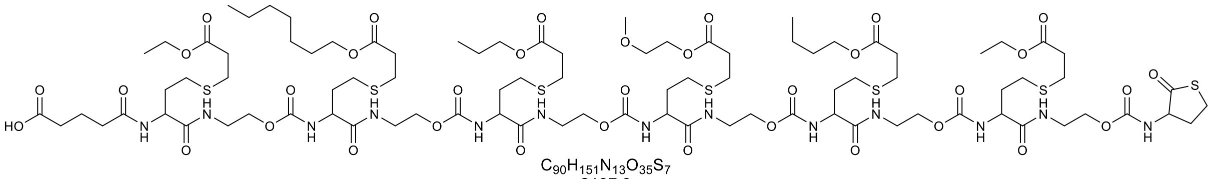

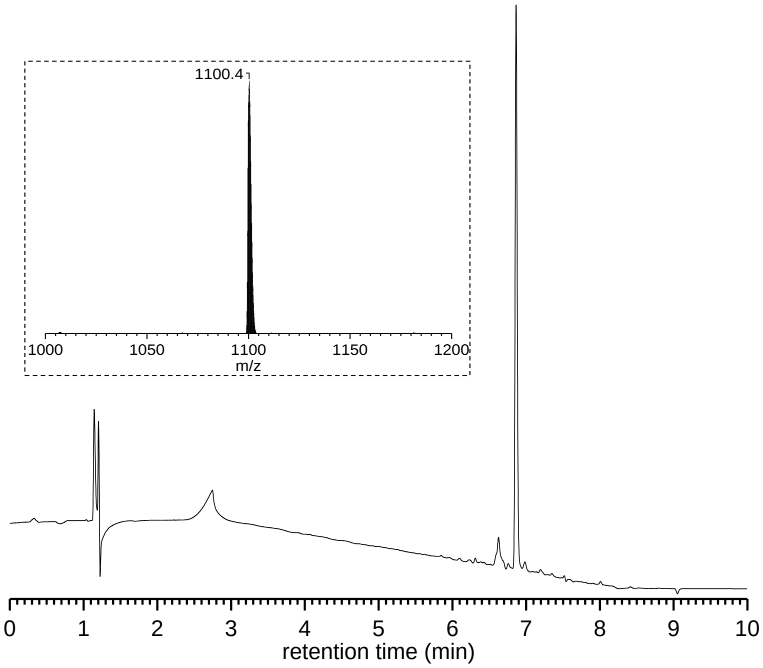

Supplementary Figure 218 | LC-ESI-MS analysis of **QR68**. Insert: ESI-MS-spectrum of dominant species (positive mode).

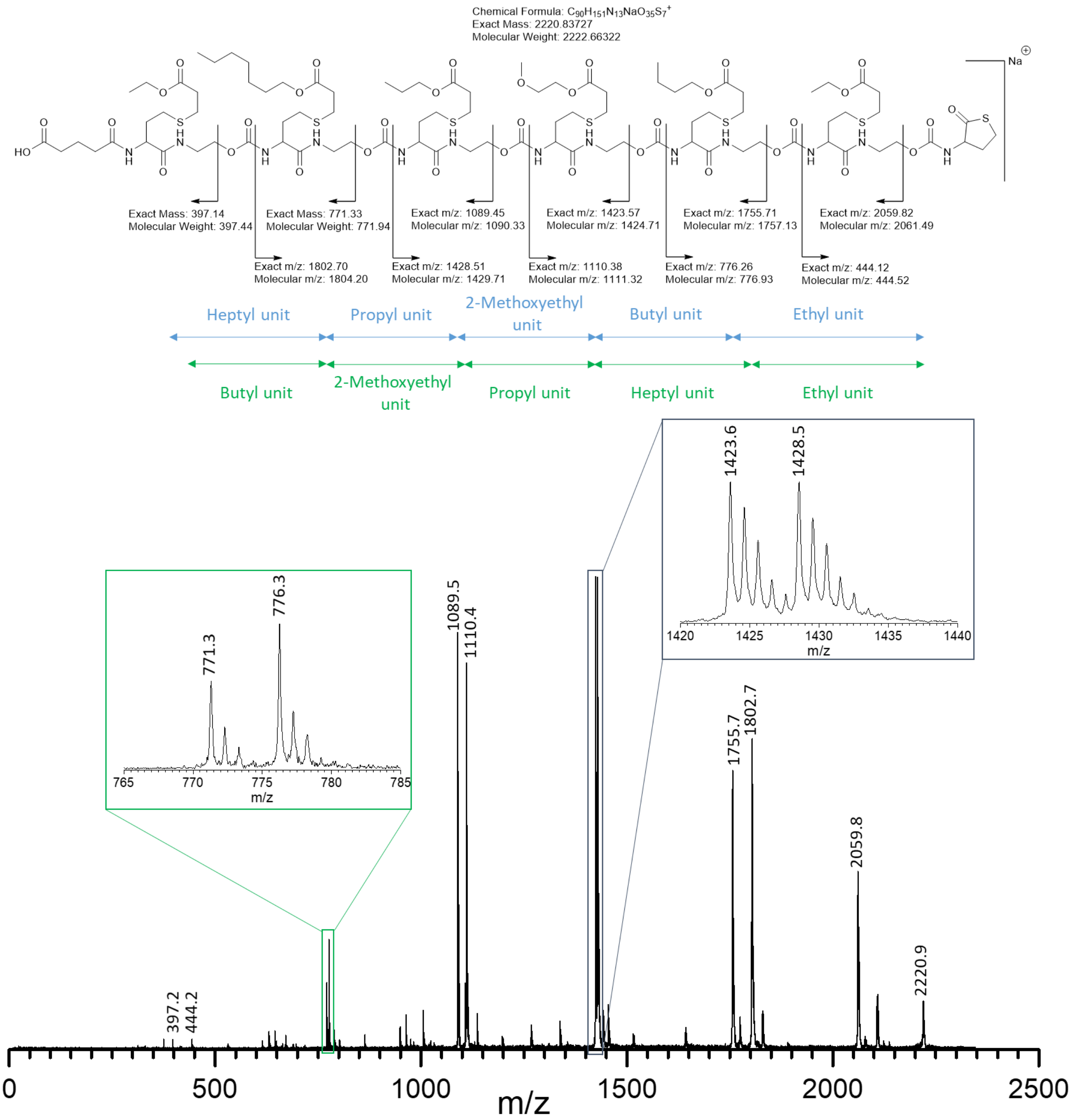

Supplementary Figure 219 | MALDI-MS/MS spectrum with peak assignment of **QR68**.

*Characterization of **QR69** using mass spectrometry (Supplementary Figure 220) and MALDI-MS/MS analysis (Supplementary Figure 221).*

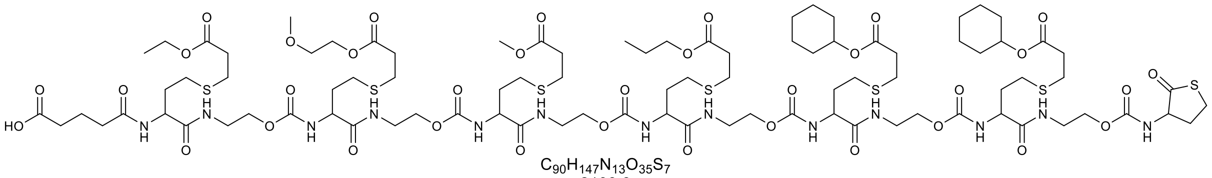

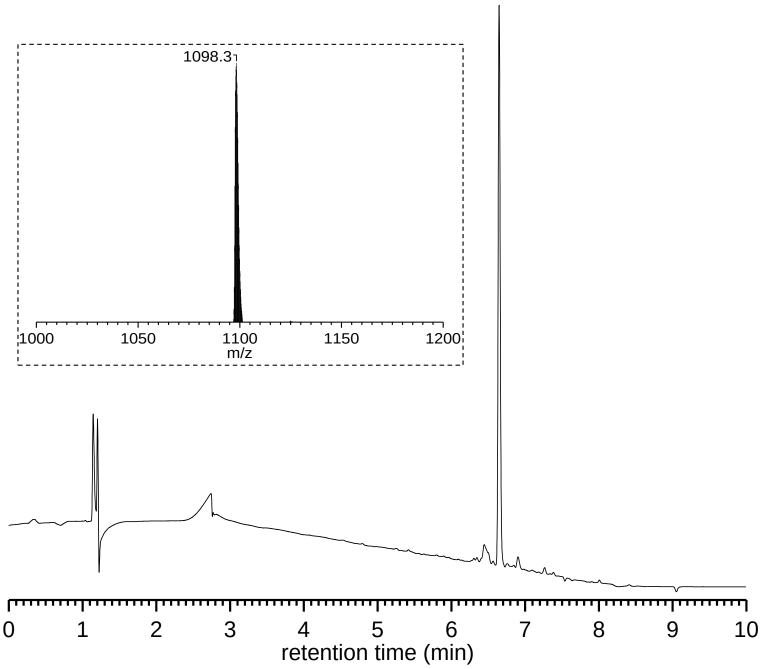

Supplementary Figure 220 | LC-ESI-MS analysis of **QR69**. Insert: ESI-MS-spectrum of dominant species (positive mode).

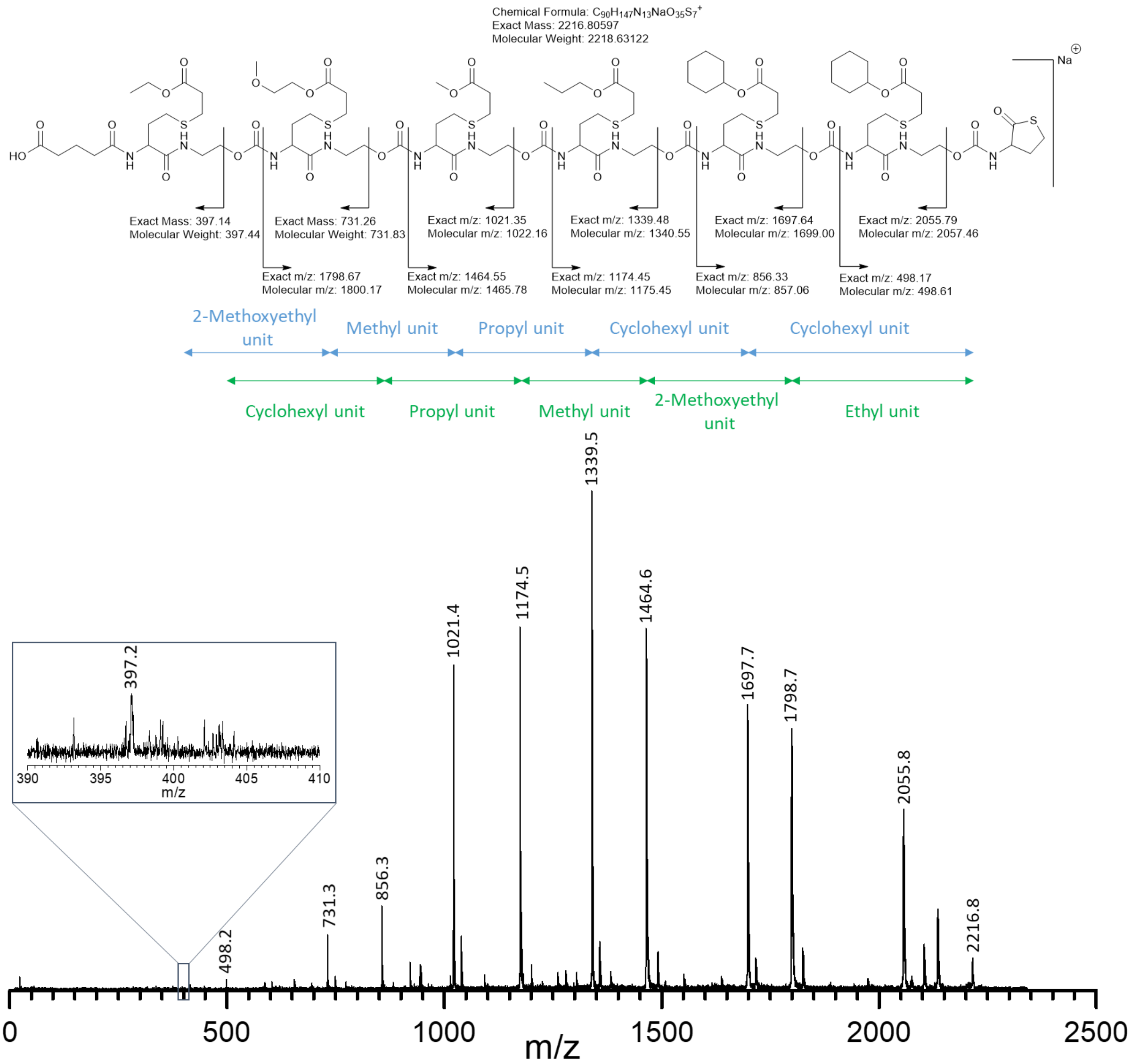

Supplementary Figure 221 | MALDI-MS/MS spectrum with peak assignment of **QR69**.

*Characterization of **QR70** using mass spectrometry (Supplementary Figure 222) and MALDI-MS/MS analysis (Supplementary Figure 223).*

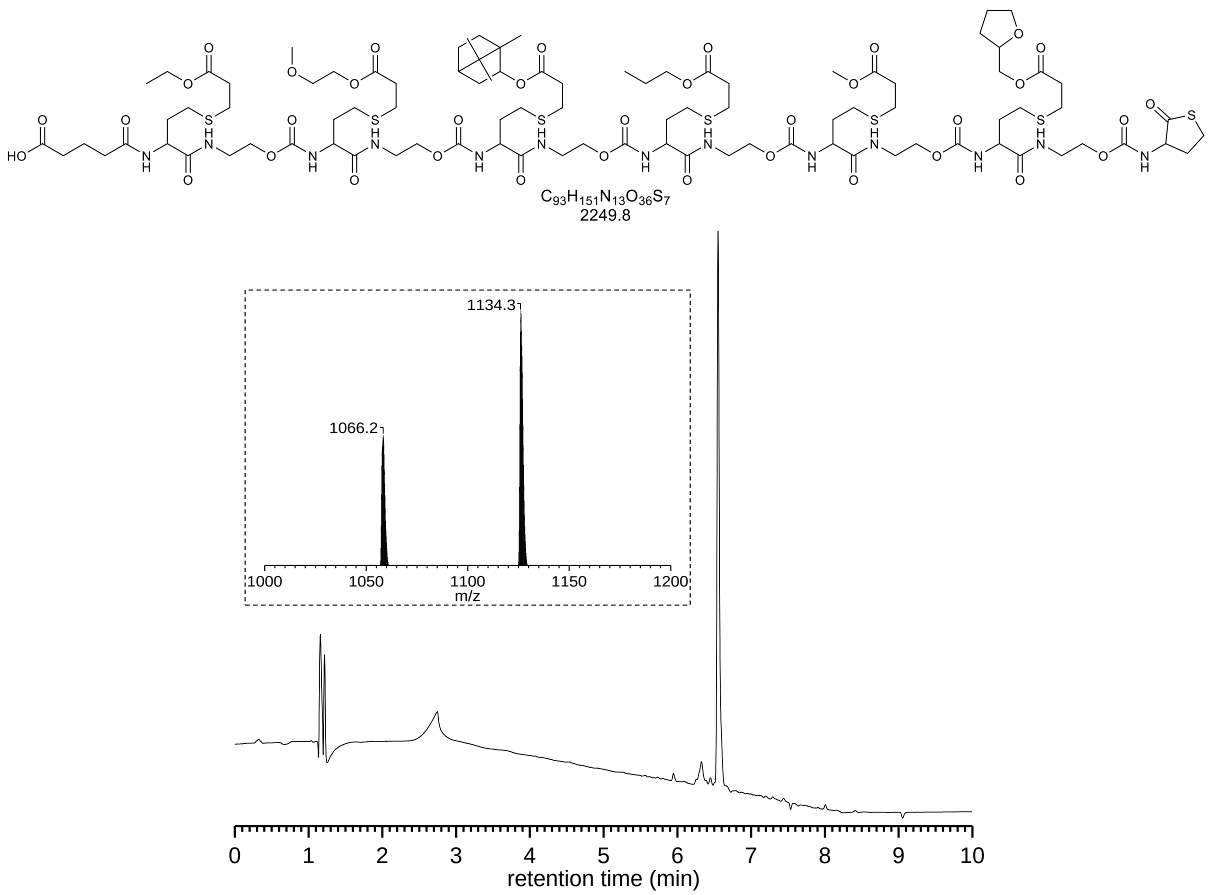

Supplementary Figure 222 | LC-ESI-MS analysis of **QR70**. Insert: ESI-MS-spectrum of dominant species (positive mode).

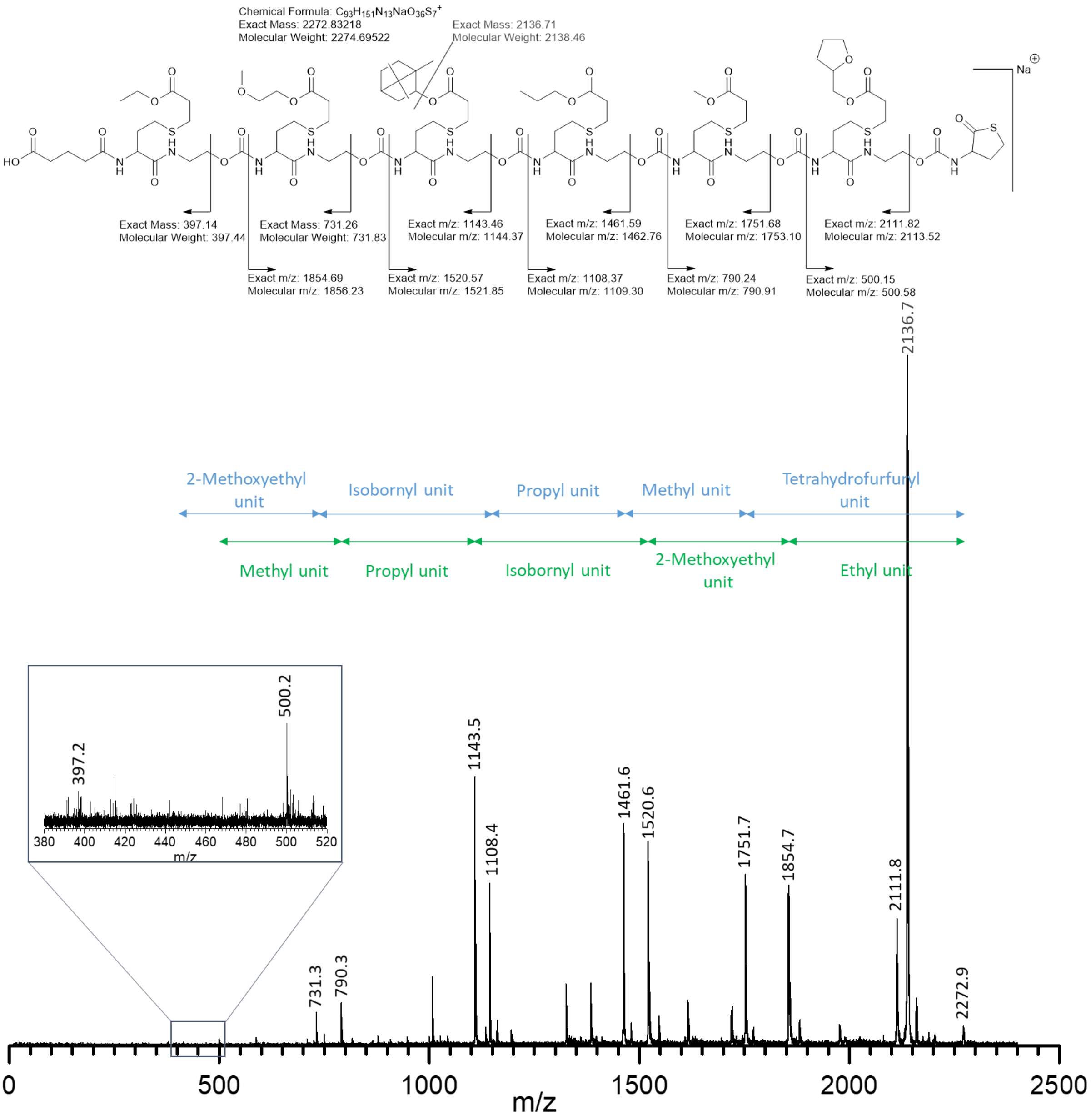

Supplementary Figure 223 | MALDI-MS/MS spectrum with peak assignment of **QR70**.

*Characterization of **QR71** using mass spectrometry (Supplementary Figure 224) and MALDI-MS/MS analysis (Supplementary Figure 225).*

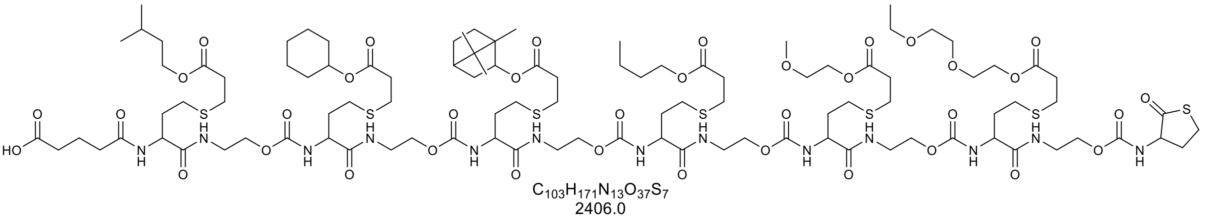

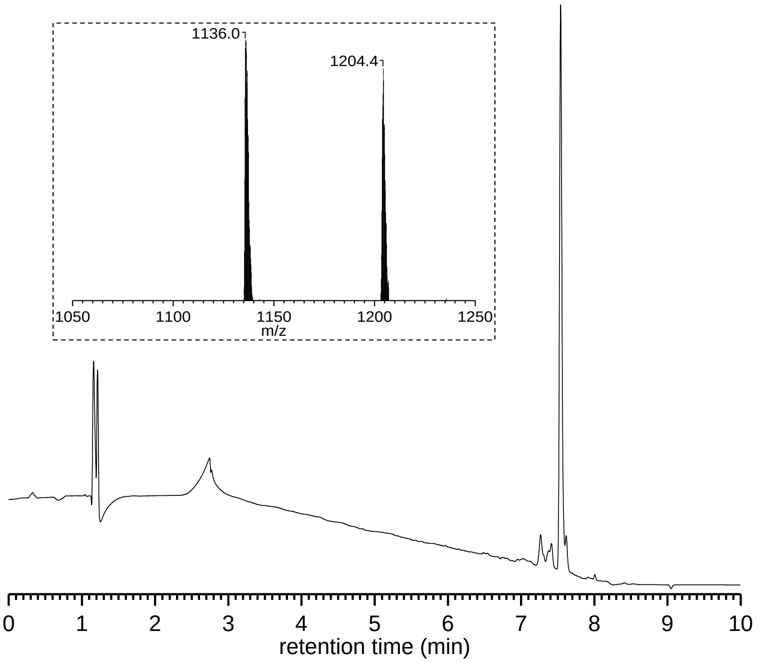

Supplementary Figure 224 | LC-ESI-MS analysis of **QR71**. Insert: ESI-MS-spectrum of dominant species (positive mode).

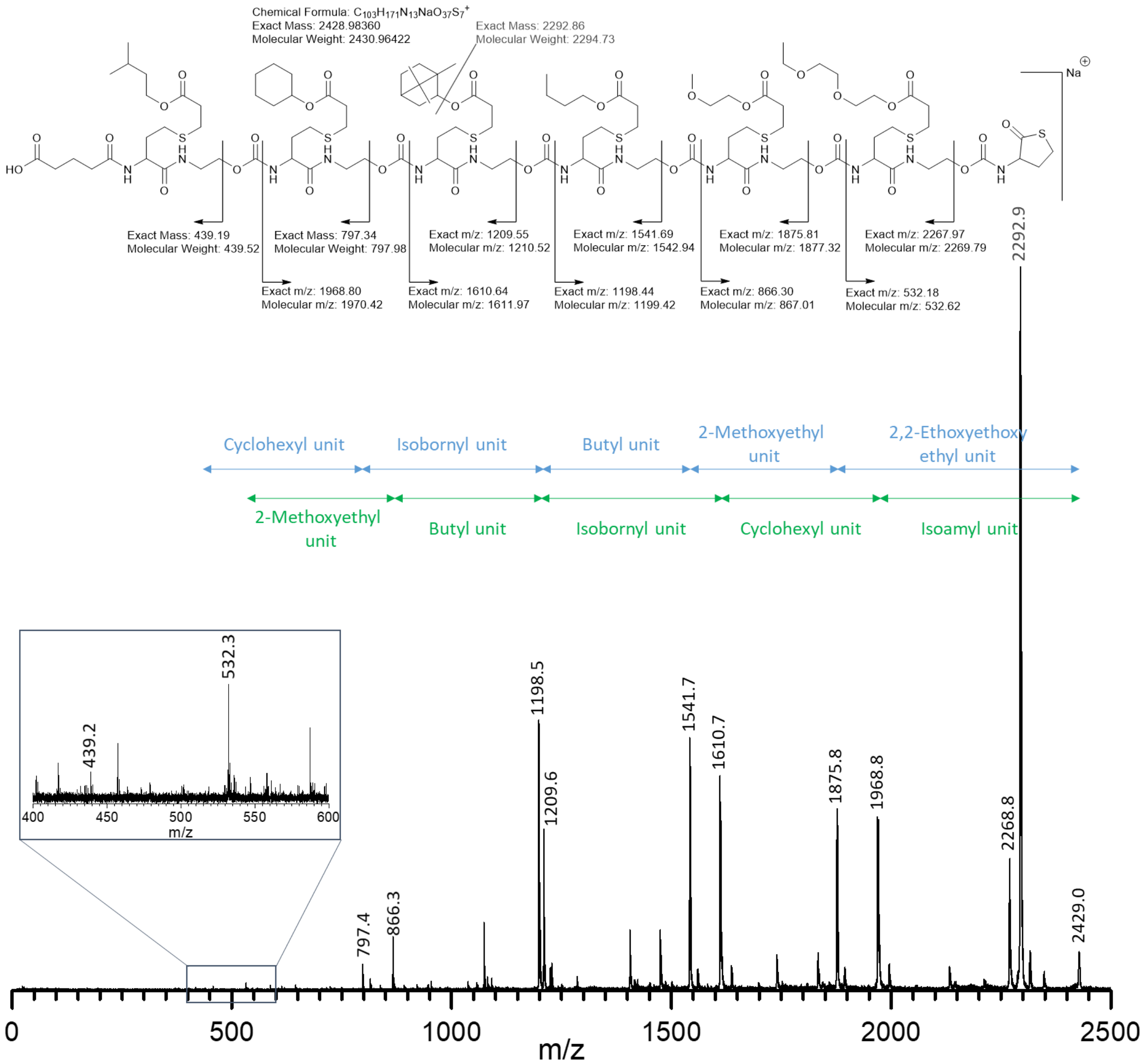

Supplementary Figure 225 | MALDI-MS/MS spectrum with peak assignment of **QR71**.

Supplementary References

1. Espeel P, *et al.* Multifunctionalized Sequence-Defined Oligomers from a Single Building Block. *Angew Chem, Int Ed* **52**, 13261-13264 (2013).

2. Van Camp W, Du Prez FE, Bon SAF. Atom Transfer Radical Polymerization of 1-Ethoxyethyl (Meth)acrylate: Facile Route toward Near-Monodisperse Poly((meth)acrylic acid). *Macromolecules* **37**, 6673-6675 (2004).

3. Martens S, Van den Begin J, Madder A, Du Prez FE, Espeel P. Automated Synthesis of Monodisperse Oligomers, Featuring Sequence Control and Tailored Functionalization. *J Am Chem Soc* **138**, 14182-14185 (2016).

4. Richter C, Schaepe K, Glorius F, Ravoo BJ. Tailor-made N-heterocyclic carbenes for nanoparticle stabilization. *Chem Commun* **50**, 3204-3207 (2014).

5. Dijkstra EW. A note on two problems in connexion with graphs. *Numer Math* **1**, 269-271 (1959).
